# Integrated Proteomics Analysis of Baseline Protein Expression in Pig Tissues

**DOI:** 10.1021/acs.jproteome.3c00741

**Published:** 2024-05-08

**Authors:** Shengbo Wang, Andrew Collins, Ananth Prakash, Silvie Fexova, Irene Papatheodorou, Andrew R. Jones, Juan Antonio Vizcaíno

**Affiliations:** † European Molecular Biology Laboratory-European Bioinformatics Institute (EMBL-EBI), Wellcome Genome Campus, Hinxton, Cambridge CB10 1SD, United Kingdom; ‡ Open Targets, Wellcome Genome Campus, Hinxton, Cambridge CB10 1SD, United Kingdom; § Institute of Systems, Molecular and Integrative Biology, University of Liverpool, Liverpool L69 7ZB, United Kingdom

**Keywords:** proteomics, meta-analysis study, protein abundance, pig organs, human−pig comparison, data integration

## Abstract

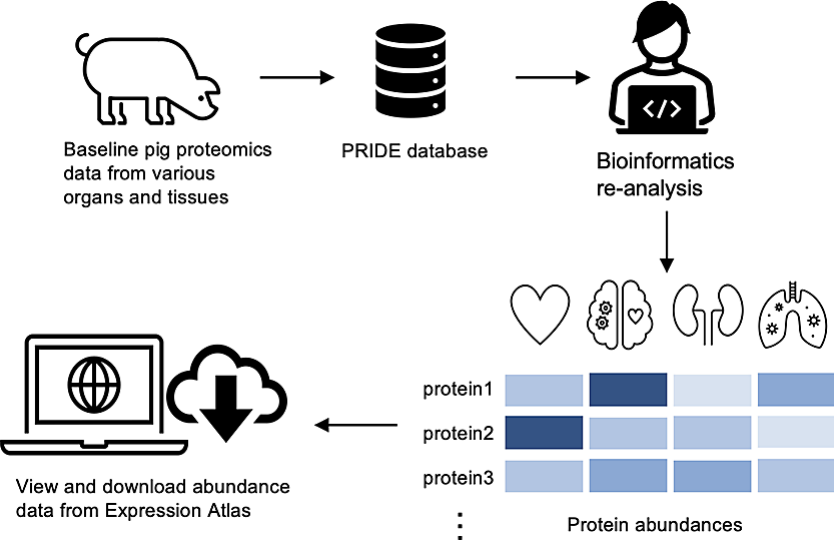

The availability of an increasingly large amount of public proteomics data sets presents an opportunity for performing combined analyses to generate comprehensive organism-wide protein expression maps across different organisms and biological conditions. *Sus scrofa*, a domestic pig, is a model organism relevant for food production and for human biomedical research. Here, we reanalyzed 14 public proteomics data sets from the PRIDE database coming from pig tissues to assess baseline (without any biological perturbation) protein abundance in 14 organs, encompassing a total of 20 healthy tissues from 128 samples. The analysis involved the quantification of protein abundance in 599 mass spectrometry runs. We compared protein expression patterns among different pig organs and examined the distribution of proteins across these organs. Then, we studied how protein abundances were compared across different data sets and studied the tissue specificity of the detected proteins. Of particular interest, we conducted a comparative analysis of protein expression between pig and human tissues, revealing a high degree of correlation in protein expression among orthologs, particularly in brain, kidney, heart, and liver samples. We have integrated the protein expression results into the Expression Atlas resource for easy access and visualization of the protein expression data individually or alongside gene expression data.

## 1 Introduction

In recent years, high-throughput mass spectrometry (MS)-based proteomics methods have made significant advances and have become essential tools in biological research.^1^ These improvements are the result of significant developments in MS instrumentation, chromatographic methods, sample preparation automation, and computational analysis.^2^ The dominant experimental technique for MS-based proteomics has historically been data-dependent acquisition (DDA) bottom-up proteomics.^3^ Among the quantitative techniques, label-free DDA approaches are well accepted. However, data-independent acquisition (DIA) approaches are currently becoming increasingly popular.

In parallel to the technical developments, in recent years, the proteomics community has embraced open data practices, leading to a substantial increase in the availability of shared data sets in the public domain. The field has mirrored the progress witnessed in genomics and transcriptomics. The PRIDE database,^4^ as part of the ProteomeXchange consortium,^5^ is the most used proteomics data repository worldwide. The availability of extensive public proteomics data sets has paved the way for various applications, including meta-analysis studies involving the reanalysis and integration of quantitative proteomics data sets.^6−9^ By systematically reanalyzing these data sets, original findings can be updated, confirmed, and/or strengthened. Moreover, novel insights beyond the scope of the original studies can be obtained through alternative reanalysis strategies to those used in the original studies.^10^

To enable access to proteomics data by the wider scientific community, PRIDE is developing data dissemination and integration pipelines with existing popular resources at the European Bioinformatics Institute (EBI). Expression Atlas^11^ (https://www.ebi.ac.uk/gxa/home) is a well-established database for gene expression data and has more recently incorporated protein expression information derived from reanalyzed data sets into its ‘bulk’ section. As a result, the integration of proteomics expression/protein abundance data with transcriptomics information, primarily from RNA-Seq experiments, enhances the comprehensive understanding of molecular expression across various biological contexts. This approach ensures the long-term accessibility and integration of proteomics data, benefiting researchers, including those without expertise in proteomics, in their exploration of multiomics information.

We have already performed combined analyses of baseline (without any perturbation) protein expression for human,^7^ mouse, and rat tissues.^6^ Here, we are reporting an analogous study of baseline protein expression in the model organism *Sus scrofa*, the domestic pig. The study of pig proteomics data sets is crucial for advancing food production, animal welfare, and human biomedical research, as it offers insights into genetic and environmental factors affecting farm animal production and leverages the close genetic and proteomic similarities between pigs and humans.^12,13^ The resource PeptideAtlas provided a few years ago a build for pigs, including extensive peptide and protein identification data, but is not providing protein abundance information.^8^ Additionally, PaxDB recently released a new version 5.0,^14^ providing expression data coming from different vertebrates, including *Sus scrofa*, but quantitative data is based on spectral counting, a semiquantitative technique. Also, no tissue-specific information is provided there, apart from the liver. To the best of our knowledge, we are providing the first combined quantitative analysis of label-free DDA data sets in pigs.

Here, we report the reanalysis and integration of 14 public label-free pig baseline tissue data sets, including 14 organs and a total of 20 healthy tissues from 128 samples. The results were incorporated into Expression Atlas as baseline studies. Additionally, we report a comparative analysis of protein expression across pig and human tissues among other analyses.

## 2 Methods

### 2.1 Data Sets

The PRIDE database hosted 165 publicly available MS proteomics data sets of *Sus scrofa* as of October 2022. For this study, we manually selected data sets based on several predefined criteria, which included (i) label-free DDA studies from baseline tissues (without any perturbation) and without enrichment for post-translational modifications; (ii) data sets generated using Thermo Fisher Scientific instruments to avoid the heterogeneity introduced by data generated by other platforms; and (iii) data sets with sufficient sample metadata, manually curated from the original publication. This resulted in the identification of 14 pig data sets for further analysis.

Sample and experimental metadata were manually curated using Annotare,^15^ and adhering to the Investigation Description Format (IDF) and Sample-Data Relationship Format (SDRF) files,^16^ which are needed for integration of the data into Expression Atlas. The IDF file contains an overview of the experimental design, including details on experimental factors, protocols, publication information, and contact information. The SDRF file contains complementary information: the sample metadata that describes the relationships between various sample characteristics and the associated data files within the data set.

### 2.2 Proteomics Raw Data Processing

All data sets underwent analysis using MaxQuant version 2.0.3.0^17^ in multithreaded mode on a Linux high-performance computing cluster for peptide/protein identification and protein quantification. Input parameters for each data set, including MS1 and MS2 tolerances, digesting enzymes, and fixed and variable modifications, were set according to the specifications provided in their respective publications and accounting for two missed cleavage sites. The false discovery rate (FDR) at both the peptide spectrum match (PSM) and protein levels was set to 1%. The remaining parameters of MaxQuant were set to the default values: a maximum of 5 modifications per peptide, a minimum peptide length of 7 amino acids, and a maximum peptide mass of 4600 Da. For the “match between runs” option, a minimum match time window of 0.7 s and a minimum retention time alignment window of 20 s were applied. MaxQuant parameter files can be downloaded from the Expression Atlas. The *Sus scrofa* UniProt^18^ Reference proteome release-2021_04 (including isoforms, 49,865 sequences) was used as the target sequence database for the pig data sets. MaxQuant uses a built-in database of contaminants, and a decoy database was generated by reversing the input database sequences following the respective enzymatic digestion.

### 2.3 Postprocessing

The postprocessing of MaxQuant results followed the methodology detailed in previous publications.^6^ In short, after removing the protein groups labeled as potential contaminants, decoys, and those with less than 2 PSMs, the protein intensities in each sample were normalized by scaling the iBAQ intensity values with the total signal in each MS run and converting to parts per billion (ppb).
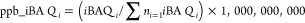


UniProt protein accessions, from the MaxQuant output- proteinGroups.txt file, were mapped to their Ensembl gene identifiers (ENSSSCG) using the ID mapping data set (Release 2022/03) at the UniProt Web site (https://www.uniprot.org/id-mapping).^19^ The resulting id mapping data file (idmapping_selected.tab), by default, maps UniProt protein accessions to Ensembl gene identifiers of all pig breeds (i.e., Landrace, Pietrain, etc.) rather than to the reference breed, thus leading to multiple Ensembl gene identifiers being returned per UniProt protein identifier. To resolve this, we downloaded the *Sus scrofa* model Ensembl fasta peptide dump (Release 11.1 gene set, https://ftp.ensembl.org/pub/release-110/fasta/sus_scrofa/pep/Sus_scrofa.Sscrofa11.1.pep.all.fa.gz) and used this as a filter to keep only gene-mappings specific to the reference pig breed. We used the reference pig Ensembl gene identifiers for further downstream analysis.

During downstream postprocessing, we removed protein groups which mapped to more than one gene identifier, and for cases where two or more protein groups mapped to the same gene identifier, protein intensities were aggregated using the median value. The parent genes to which the different protein groups were mapped to are equivalent to “canonical proteins” in UniProt (https://www.uniprot.org/help/canonical_and_isoforms), and therefore the term protein abundance is used to describe the protein abundance of the canonical protein throughout the article.

### 2.4 Integration into Expression Atlas

The normalized protein abundances along with the validated SDRF files, summary files detailing the postprocessing quality assessment, and MaxQuant parameter files (mqpar.xml) are available to download from Expression Atlas. Table 1 describes the data sets and their corresponding E-PROT identifiers.

**Table 1:** List of Pig Proteomics Datasets and Their Main Characteristics

**expression Atlas accession number**	proteomics data set identifier*	**breed**	**tissues**	**organs**	**mass spectrometer**	**number of MS runs**	**number of samples**	**fraction**	**number of protein groups**^**†**^	**number of peptides**^**†**^	**number of unique peptides**^**†**^	**number of unique genes (canonical proteins) mapped**^**†**^
E-PROT-111	PXD001800^27^	landrace	retina	eye	Q exactive Plus	48	16	yes	4579	36,992	28,055	3643
E-PROT-113	PXD002918^28^	NA	biceps femoris	muscle	LTQ orbitrap XL	6	6	no	1272	9148	6163	841
E-PROT-114	PXD003204^29^	landrace	bile duct, heart, spleen, lung, brain, liver, diaphragm, pancreas, kidney	biliary system, heart, spleen, lung, brain, liver, diaphragm, pancreas, kidney	LTQ orbitrap Elite	108	9	Yes	5969	85,655	57,338	5065
E-PROT-115	PXD009577^30^	NA	heart	heart	Q exactive HF	30	6	yes	4258	47,975	32,956	3582
E-PROT-117	PXD011360^31^	landrace × piétrain	cecum, colon, Ileum, Jejunum	intestine	Q exactive Plus	16	16	no	4716	55,392	41,184	3981
E-PROT-118	PXD011536^32^	NA	liver	liver	Q exactive HF-X	5	5	no	2356	18,816	15,078	1773
E-PROT-119	PXD011755^33^	House swine	eye	eye	LTQ orbitrap XL	204	12	yes	1337	7039	5660	888
E-PROT-120	PXD012636^34^	NA	heart	heart	Q exactive HF	120	4	yes	7747	151,177	94,341	6982
E-PROT-122	PXD014893^35^	NA	biceps femoris, triceps muscles	muscle	Q exactive HF-X	8	4	yes	1287	13,065	8527	1004
E-PROT-124	PXD016003^36^	NA	biceps femoris, triceps muscles	muscle	Q exactive HF-X	8	4	yes	2379	22,409	15,650	1885
E-PROT-125	PXD017671^37^	NA	liver	liver	Q exactive HF-X	8	8	no	3128	32,215	24,585	2576
E-PROT-126	PXD019852^38^	NA	heart	heart	Q exactive HF-X	8	8	no	1527	13,511	10,085	1143
E-PROT-130	PXD026910^39^	NA	subcutaneous adipose tissue, mesenteric adipose tissue	adipose tissue	Q exactive HF	10	10	No	2586	23,718	17,676	2107
E-PROT-131	PXD027772^40^	landrace × swabian-hall	skeletal muscle, heart	skeletal muscle, heart	Q exactive HF	20	20	no	3242	39,140	25,993	2797
TOTAL	14 data sets		20 tissues	14 organs		599 MS runs	128 samples					

### 2.5 Protein Abundance Comparison across Data Sets

The normalized protein abundances (in ppb values) within each data set were transformed into ranked bins as described in.^7^ Briefly, the normalized protein abundance (ppb) of each MS run was sorted from lowest to highest and binned into 5 equal length bins. Proteins ranked in the lowest bin (bin 1) represent the lowest abundance, and correspondingly, proteins ranked in bin 5 have the highest abundance. To analyze and compare the data effectively, protein abundances from ‘tissues’ were grouped into ‘organs’. For example, the Ileum and jejunum “tissues” were grouped as “small intestine”. Similarly, triceps, biceps femoris, diaphragm, and skeletal muscle were grouped as “muscle”. When tissues were combined into organs, median bin values were used.

Proteins of all the samples were selected for uniform manifold approximation and projection (UMAP)^20^ representation and analyzed for binned abundance values using the R programming language (https://www.R-project.org/). Pearson correlation coefficients (rp) were calculated for all samples based on paired complete observations and used to generate a heatmap. Missing values were marked as NA (not available). For each organ, the median rp was calculated from all paired rp values of the respective sample. Columns and rows of the samples were clustered hierarchically by using Euclidean distances.

### 2.6 Organ-Specific Expression Profile Analysis

To make comparisons of protein expression across organs based on organ specificity, we grouped the proteins into three categories based on the classification scheme of Uhlen et al.^21^: (1) “Organ-enriched”: present in one unique organ with bin values 2-fold higher than the mean bin value across all organs; (2) “group-enriched”: present in at least 7 organs, with bin values 2-fold higher than the mean bin value across all organs; and (3) “mixed”: the remaining canonical proteins that are not part of the above two categories.

We then performed Gene Ontology (GO) term enrichment analysis through an over-representation test on the “organ-enriched” and “group-enriched” using the mapped gene lists for each organ. The computational analysis was carried out in the R programming language with the package clusterProfiler^22^ version 3.16.1 by using the function enrichGO() for the GO term over-representation test. The p value cutoff was set to 0.05, and the q value cutoff was set to 0.05.

### 2.7 Comparison of Protein Expression Values between Pig and Human Tissues

The orthologous genes for pigs and humans were obtained following the procedure described in the Ensembl BioMart.^23^ Briefly, we first selected the “Ensembl genes 110″, then chose “Human genes (GRCh38.p14)”, clicked on “Filters” in the left menu, then unfolded the “MULTI SPECIES COMPARISONS” box, ticked the “Homolog filters” option, and chose “Orthologous Pig Genes” from the drop-down menu. Then, we clicked on “Attributes” in the left menu, unfolded the “Pig ORTHOLOGS” box, and selected the pig gene ID and pig gene name. Finally, we clicked on the “Results” button (top left) to download the list of orthologous genes between humans and pigs. The orthologous gene list was filtered to include only parent gene identifiers from pig samples in this study and the parent genes of human samples described in our previous study using human baseline tissue samples.^7^

We used the calculation of “edit distance”^7^ of a protein, which was computed as the difference between two pairs of protein abundance bins in pigs and humans. The following categories were used to classify their groups of protein expression samples: (1) “Group A″: protein abundance is similar between human and pig tissues; (2) “Group B″: protein abundance is higher in human tissues when compared to pig tissues; and (3) “Group C ″: protein abundance is higher in pig tissues compared to human tissues.

A GO term enrichment analysis was performed using the mapped gene lists for each organ in each group (“Group A″, “Group B″, or “Group C″) as the foreground and the gene list of all three groups as the background. The settings were the same as those used for organ-specific expression profile analysis in the previous section.

The one-to-one mapped orthologue identifiers were used to compare pig and human protein intensities. Additionally, their normalized protein abundance (using parts per billion values) in 10 organs (adipose tissue, brain, colon, heart, kidney, liver, lung, pancreas, small intestine, and spleen) was used to assess pairwise correlations. Linear regression was calculated using the linear fit “lm” method in the R programming language.

### 2.8 Correlation between Gene (RNA-seq) and Protein Expression

One pig RNA-seq experimental tissue baseline data set (the only one available) was obtained from Expression Atlas (data set E-MTAB-5895). The data set was composed of pig samples from a Duroc breed.^24^ Transcriptomics data had been previously collated in Expression Atlas, and FPKMS (Fragments per Kilobase of transcript per Million mapped reads) data were computed by iRAP (https://github.com/nunofonseca/irap) based on the raw data, which were first averaged based on technical replicates, then quantile normalized within each set of biological replicates using limma^25^ and finally averaged again over all biological replicates. Biological metadata were collected in the SDRF format, consistent with the proteomics data.

### 2.9 Comparison of Protein Abundance with Spectral Counting Values from PaxDB

We compared the protein abundances generated in our study with the protein abundance data from PaxDB version 5.0 (https://www.pax-db.org/)^26^ available for *Sus scrofa*. Normalized iBAQ abundances were compared with the spectral counting abundances for the liver, the only matching organ available. This comparison was not possible for other pig organs as other data in PaxDB are labeled as “whole organism”. Ensembl gene ids (ENSSSCG) were mapped to protein ids (ENSSSCP) in PaxDB using the Ensembl BioMart, as described in this tutorial.^23^

## 3 Results

### 3.1 Pig Proteomics Data Sets

In summary, we obtained protein expression data from 20 healthy tissues in 14 organs, coming from 14 public data sets. The analyses covered a total of 599 MS runs from 128 samples that were annotated as healthy/control/nontreated samples, thus representing baseline protein expression. Noncontrol/disease samples associated with these data sets were also analyzed but are not discussed here. Normalized protein abundance values (as ppb) from both control/healthy/nontreated and disease/treated tissue samples are available to view as heatmaps in Expression Atlas. The protein abundances along with sample annotations, the sample quality assessment summary, and experimental parameter inputs for MaxQuant can be downloaded from Expression Atlas as text files. The total number of proteins and peptides identified in these data sets is shown in Table 1.

### 3.2 Protein Coverage across Organs and Data Sets

A total of 7,767 protein groups were identified from the reanalysis of the 14 pig data sets, among which 2,164 protein groups (27.9%) were uniquely present in only one organ and 523 protein groups (6.7%) were ubiquitously observed (Table S1 in Supporting File 2). However, it should be emphasized that a specific list of typical proteins detected in only one organ should be treated with caution, as the FDR of this list will be amplified due to the accumulation of false positives when the data sets were analyzed individually. For proteins detected in four or more data sets, this should not be a problem, as from the common number of decoy protein hits across data sets, a protein FDR of less than 1% could be inferred for those proteins (Figure S1 in Supporting File 1).

**Figure 1:**
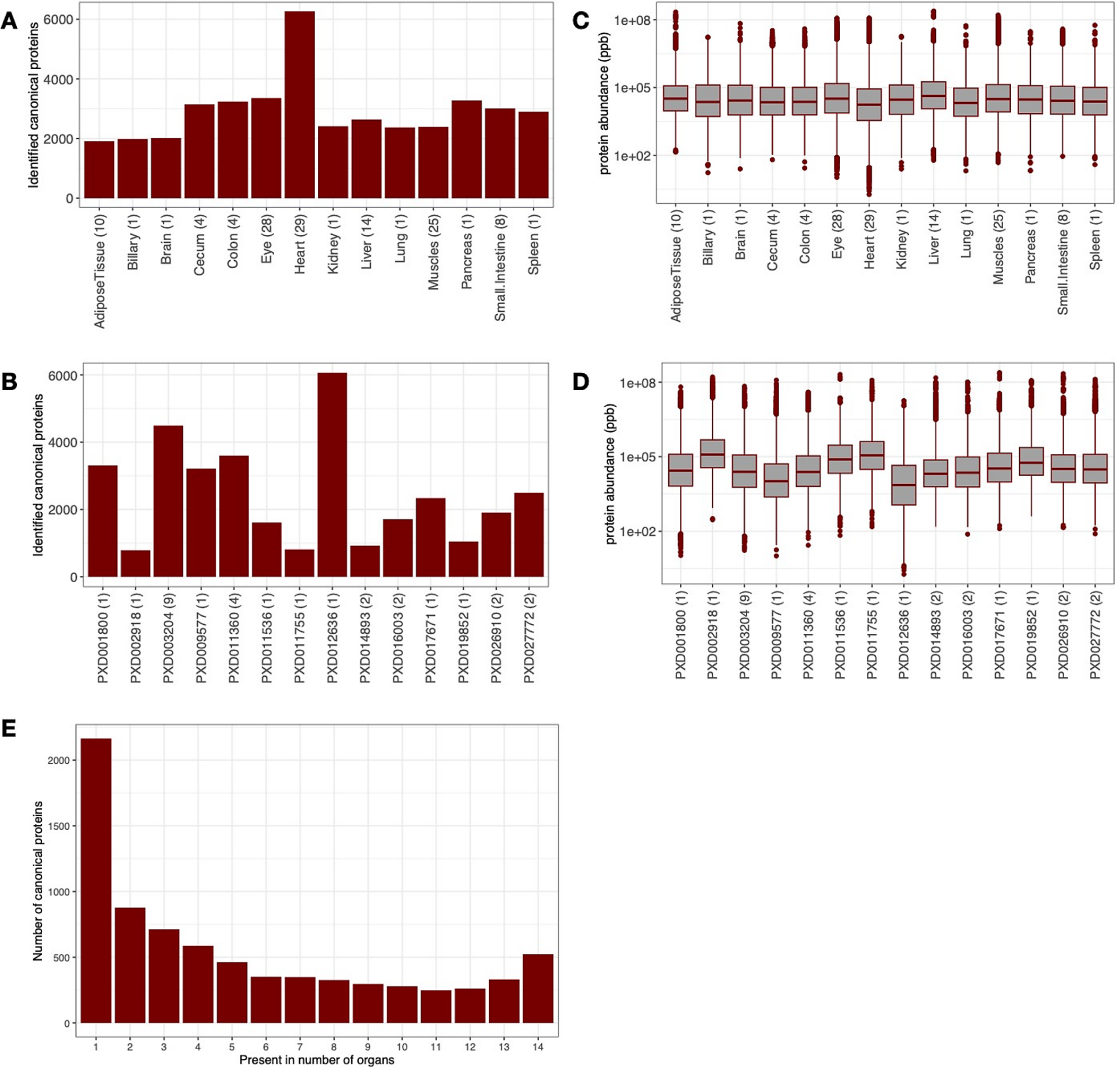
Distribution of canonical proteins detected per organ and data set. (A) Number of canonical proteins identified across different pig organs. The number in parentheses denotes the number of samples. (B) Number of canonical proteins identified in different data sets. The number in the brackets indicates the number of unique tissues in the data set. (C) Range of normalized iBAQ protein abundances across different organs. The number in brackets indicates the number of samples. (D) Range of normalized iBAQ protein abundances across different data sets. The number in brackets indicates the number of unique tissues in the data set. (E) Distribution of canonical proteins identified across different organs.

Protein groups were mapped to 7780 genes (which are equivalent to canonical proteins, the term that we will be using from now on in the article). The largest number of canonical proteins was detected in samples from the heart (6264, 80.5% of the total) and the lowest number in samples from adipose tissue (1913, 24.6%) and from the biliary system (1983, 25.5%) (Figure 1A). The lower number of proteins identified in the biliary system could be attributed to the smallest sample size (only one sample out of 128, 0.08%). Data set PXD012636, a data set containing pig heart samples, which was fractionated, provided the highest number of detected canonical proteins (6062, 77.9%), whereas the smallest number of proteins were detected in data set PXD002918 (biceps femoris, 789, 10.1%, nonfractionated data set) (Figure 1B).

We studied the normalized protein abundance distribution in organs (Figure 1C) and found that all organs had similar median abundances. However, one cannot attribute biological meaning to these observations, since the method of normalization by definition fixes each sample to have the same “total abundance”, which then gets shared out among all proteins. The normalized protein abundance distribution in data sets indicated a lower than median abundance detected in the data set PXD012636 (heart), as a direct result of more proteins being detected overall in this data set (Figure 1D). In terms of the distribution of proteins detected per organ, most proteins were found in just one organ (Figure 1E).

### 3.3 Protein Abundance Comparison across Organs and Data Sets

Next, we studied how protein abundances compared across different data sets and organs. To make protein abundance values more comparable between data sets, we transformed the normalized iBAQ intensities into ranked bins as explained in Section 2, i.e., proteins included in bin 5 are highly abundant, whereas proteins included in bin 1 are expressed in the lowest abundances (among the detected proteins). We found that 494 (6.3%) proteins were expressed in at least 3 organs, with a median bin value greater than 4 (not including 4). At the other end of the scale, 337 (4.3%) canonical proteins were expressed in at least 3 organs at a median bin value less than 2 (not including 2). The bin transformed abundances in all organs and in all data sets are provided in Tables S2 and S3 in Supporting File 2.

**Figure 2:**
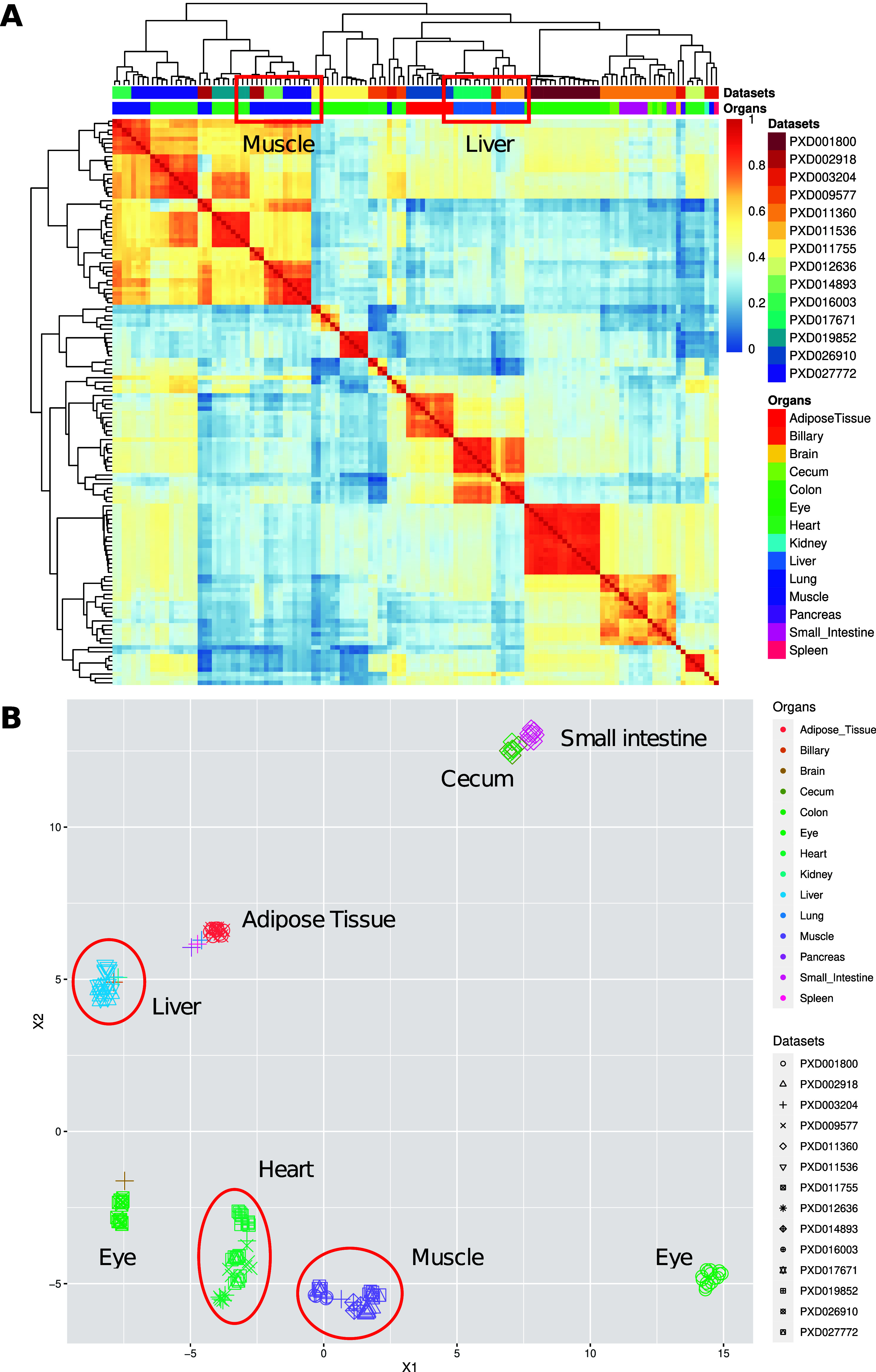
(A) Heatmap of pairwise Pearson correlation coefficients across all pig samples among data sets and organs. The colors on the heatmap represent the correlation coefficients calculated using the bin transformed values. Hierarchical clustering of the columns and rows of the samples was performed by using Euclidean distances. (B) UMAP representation among different data sets and organs. Groups of data sets coming from the same organ are highlighted.

To compare protein expression across all pig organs, we calculated pairwise Pearson correlation coefficients (rp) for the 128 samples (Figure 2A). We observed a good correlation of protein expression within the liver (median rp = 0.77) and muscle (median rp = 0.65) samples. We then performed a cluster analysis using UMAP^20^ on all samples to test the effectiveness of the bin-transformed method in reducing batch effects (Figure 2B). We observed that samples from different data sets belonging to the same organ were generally clustered together. For example, liver samples from data sets PXD003204, PXD011536, and PXD017671 clustered together (color blue in Figure 2B). Additionally, muscle samples from PXD002918 (tissue biceps femoris), PXD003204 (tissue diaphragm), PXD014893 (tissue biceps femoris and triceps muscle), and PXD016003 (tissue biceps femoris and triceps muscle) clustered together too (color purple). Similarly, heart samples from data sets PXD003204, PXD009577, PXD012636, and PXD019852 clustered together as well (color dark green).

### 3.4 Organ Elevated Proteome and the Over-Representative Biological Processes

To get more insights about organ expression specificity, proteins were classified into three different groups: “group-enriched”, “organ-enriched” and “mixed” (see Section 2 for details, Table S4 in Supporting File 2). The analysis (Figure S2 in Supporting File 1) showed that on average 26.8% of the total elevated canonical proteins were organ group-specific in pig. In addition, 4.2% were unique organ-enriched in pig. The highest ratio of group-specific proteins was found in the pancreas (36.8%), and the highest ratio of organ-enriched proteins was found in the heart (23.9%).

A GO enrichment analysis (see Section 2) was performed on those proteins that were “organ-enriched” and “group-enriched”. Overall, 310 GO terms were found to be statistically significant in all organs. The two most significant GO terms were the ‘organic acid metabolic process’ (GO:0006082) and the ‘small molecule catabolic process’ (GO:0044282), both in the liver and in the biliary system. These terms were followed by “RNA processing” (GO:0006396) in the pancreas. For the whole list of GO terms enriched for each organ, see Table S5 in Supporting File 2.

### 3.5 Comparative Analysis of Pig and Human Protein Expression

**Figure 3:**
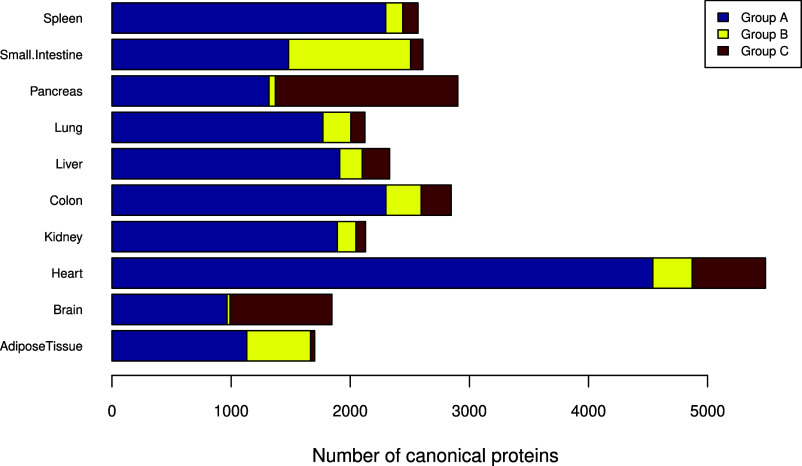
Organ specificity of canonical proteins based on edit distances between pig and human. The canonical proteins detected both in pig and human samples were classified into three groups: “Group A” (similar protein expression levels between human and pig), “Group B” (higher protein expression in human tissues), and “Group C” (higher protein expression in pig tissues).

We also performed a comparative analysis of protein abundances (in bins) between the pig baseline tissue data sets with the results obtained in our previous analogous study involving human data sets coming from baseline tissues, which was performed using the same overall methodology^7^ (Table S6 in Supporting File 2). Pig is often used as a model organism for human biomedical research, and then it is interesting to compare protein expression in both organisms. We calculated metrics to study the differences between the protein abundances for all organs found in the two studies (see ‘Material and Methods’ for full details), as shown in Figure 3 (also see Table S7 in Supporting File 2). Three groups of proteins were found according to their protein expression levels: (i) ″Group A″: protein expression is similar between human and pig tissues; (ii) ″Group B″: protein expression is higher in human tissues; and (iii) ″Group C ″: protein expression is higher in pig tissues.

We found that for pig, protein expression levels were higher in the pancreas, brain, and heart than in the corresponding human tissues, whereas protein expression levels were higher in the human’s small intestine and adipose tissue when compared to the corresponding pig tissues. For other organs, the number of proteins in “Group B” and “Group C” was quite similar. Since different sizes and counts of data sets have been used for the different organs in both species, the organ-level trends reflect the results found in our studies. In our view, they are not necessarily meaningful for understanding species level differences. Instead, for individual proteins or groups of related proteins, the comparison gives a potentially useful guide to relative protein abundance between orthologous pairs in individual organs.

We then performed a GO enrichment analysis^41^ per organ of the proteins included in the three groups using GO terms related to biological processes (see Section 2). We found 740 GO terms to be enriched in all organs overall (see all enriched GO terms in Table S8 in Supporting File 2), and in particular in the heart and kidney. For instance, in “Group A”, we found enrichment for “intracellular transport” (GO:0046907) in 9 organs (brain, colon, heart, kidney, liver, lung, pancreas, small intestine, and spleen). In “Group B”, we observed the ‘ribonucleoside monophosphate biosynthetic process’ (GO:0009156) enriched in adipose tissue and the ‘peptide biosynthetic process’ (GO:0043043) in the small intestine. Also, in “Group C”, we observed ‘vesicle-mediated transport’ (GO: 0016192) enriched in the brain and ‘carboxylic acid metabolic process’ (GO:0019752) in the pancreas. For the whole list, also including the GO terms enriched for other organs, see Table S8 in Supporting File 2.

### 3.6 Comparison of Protein Abundances across Orthologs between Pig and Human Data Sets

In a previous study, we compared the expression of canonical proteins found in three different species: human, mouse, and rat.^6^ Here, we used the same approach to compare canonical protein expression between human^7^ and pig organs. Overall, 13,248 detected human canonical proteins were compared with 7,800 detected pig canonical proteins (Table S9 in Supporting File 2). When comparing protein abundance (in ppb), we only considered the corresponding orthologous genes with unambiguous (one-to-one) mappings, which resulted in 6,811 common protein orthologs.

**Figure 4:**
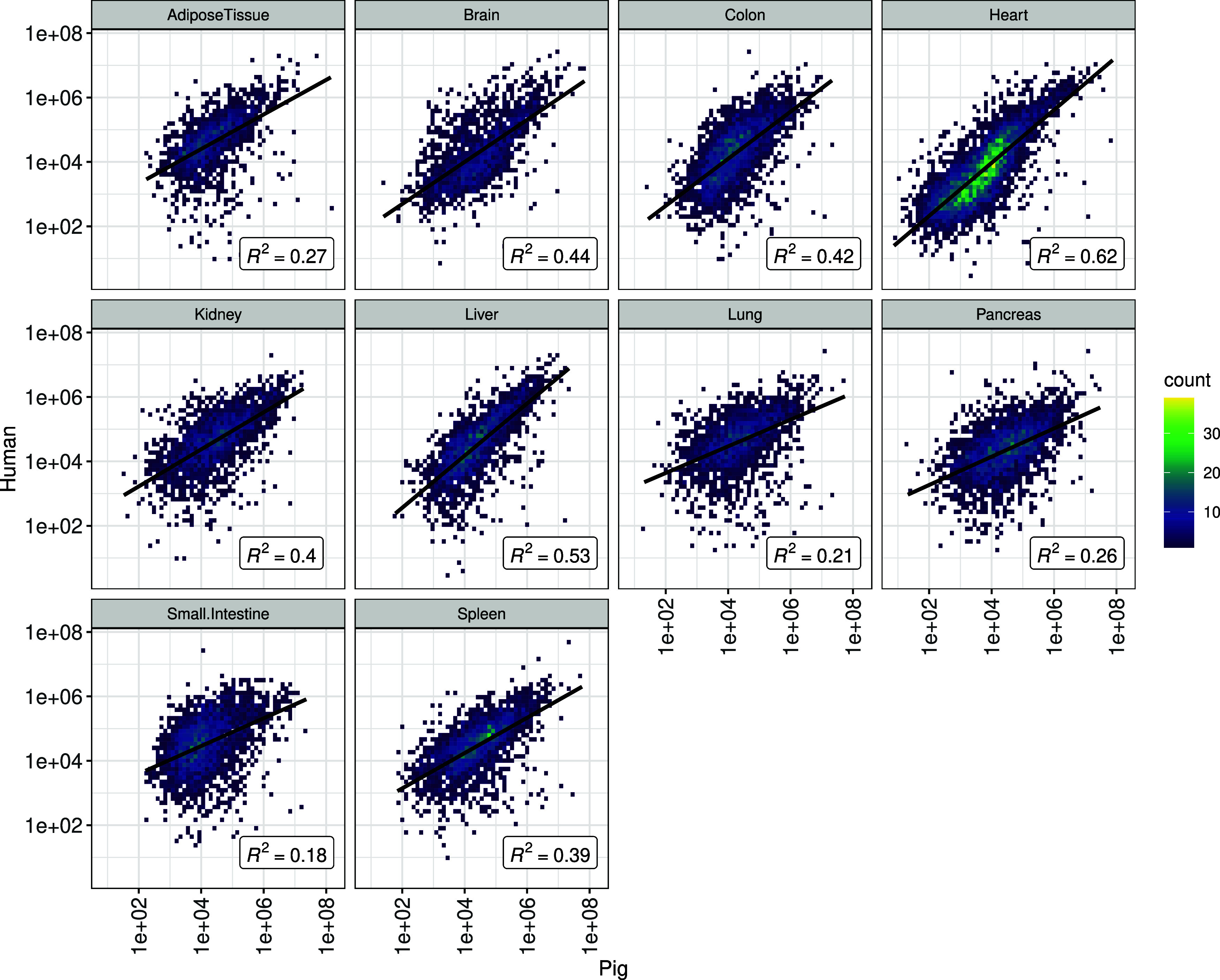
Comparison of protein abundance between orthologues of pigs and humans in various organs.

When comparing the protein expression of orthologues in humans and pigs, we observed a relatively overall high correlation in protein abundance in the heart (*R*^2^ = 0.62) and liver (*R*^2^ = 0.53), medium correlation for the brain (*R*^2^ = 0.44), colon (*R*^2^ = 0.42), kidney (*R*^2^ = 0.40), and spleen (*R*^2^ = 0.39), and low correlation in the small intestine (*R*^2^ = 0.18), lung (*R*^2^ = 0.21), pancreas (*R*^2^ = 0.26), and adipose tissue (*R*^2^ = 0.27) (Figure 4).

**Figure 5:**
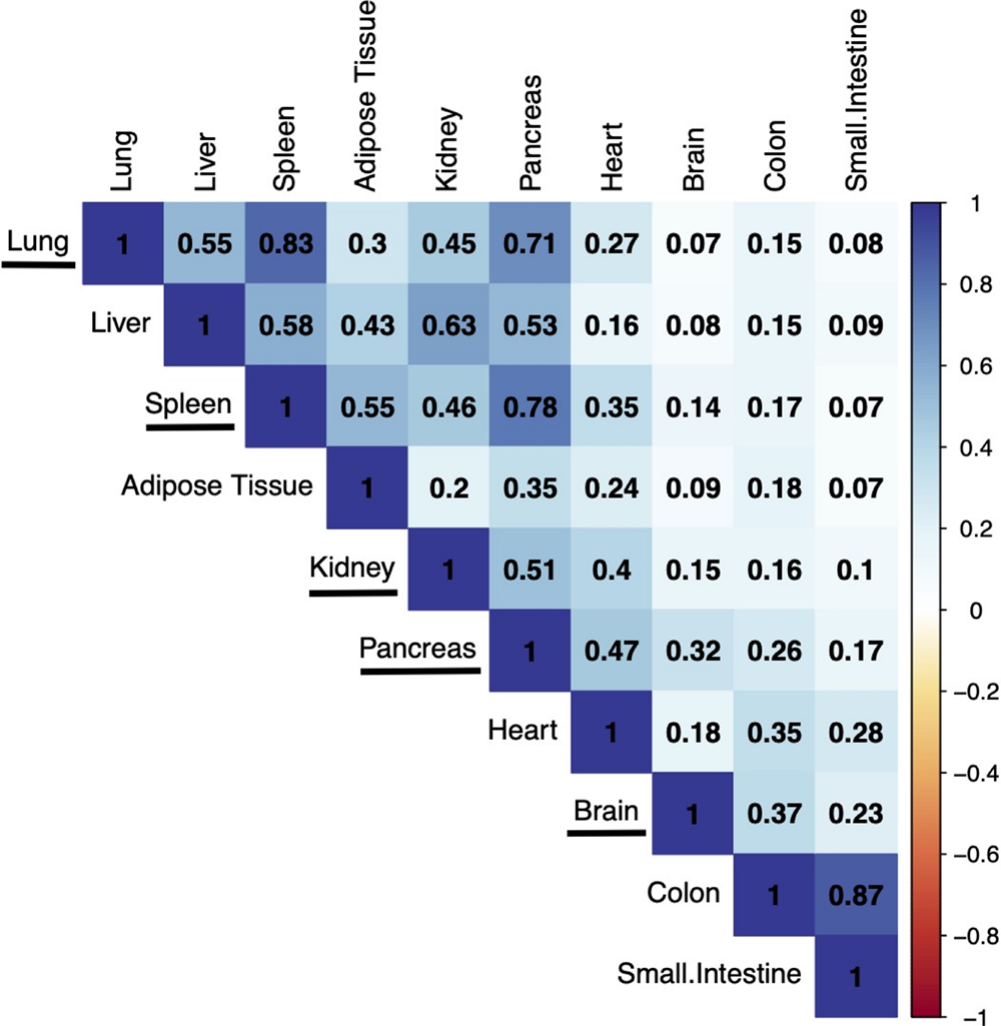
Protein expression correlation among all pig organs across the data sets included in this study. The organs underlined all come from a single data set (PXD003204).

We also investigated the correlation of protein expression across different organs in pigs across the data sets used in this study (Figure 5). The data sometimes followed expected distributions whereby tissues predicted to be more similar to each other in terms of biological function have higher pairwise correlations, e.g., small intestine and colon (*R*^2^ = 0.87) and spleen and pancreas (*R*^2^ = 0.78). However, other high correlations were found between lung and spleen (*R*^2^ = 0.83) and lung and pancreas (*R*^2^ = 0.71), likely due to these data originating from a single study. The correlation between the brain and the remaining organs was generally low, as would be expected. A plotted representation of the abundance of all sorted proteins for both species is provided in Figure S5 in Supporting File 3.

### 3.7 Comparison between Gene (RNA-seq) and Protein Expression, and Comparison with Protein Expression Data in PaxDB

We also investigated the correlation between gene (RNA-seq based) and protein expression in baseline tissue pig data sets. For that, we used the only suitable data set available in Expression Atlas (data set E-MTAB-5895).^24^ We compared the normalized iBAQ protein abundances (ppb) with the baseline RNA-seq expression (FPKM). To compare expression across different organs, we grouped the RNA-seq expression from various tissues into their respective organs by using their median values. We did not observe a strong correlation between protein and RNA expression across various organs (Figure S3 in Supporting File 1). The lowest correlation between protein and RNA expression was observed in the lung (*R*^2^ = 0.06) and the highest was observed in the heart (*R*^2^ = 0.20). In our view, these low correlations could be due to the inherent limitations in this comparison, i.e., (i) samples are not paired and (ii) the different pig breeds used (Duroc for the RNA-seq study and several others for the protein expression studies, see details in Table 1).

In addition, we compared the protein abundance of organ liver generated in this study with data from the PaxDB resource generated by using a spectral counting method. We observed that protein abundance values (fraction of total (FOT) normalized ppb values) calculated using iBAQ in this study correlated until a limited extent (*R*^2^ = 0.47) with the semiquantitative values available in PaxDB (Figure S4 in Supporting File 1). Unfortunately, it was not possible to perform this correlation for other organs (see details in Section 2).

## 4 Discussion

We have previously performed three meta-analysis studies involving the reanalysis and integration in Expression Atlas of public quantitative proteomics data sets coming from cell lines and human tumor samples,^8^ from human baseline tissues,^7^ from mouse and rat baseline tissues.^6^ Here, we have reanalyzed 14 public proteomics data sets coming from pig tissues in baseline conditions. Our overall aim was to provide a system-wide baseline protein expression catalogue across various pig organs. We used the same methodology as in the study involving baseline human tissues (and in the mouse/rat study), which enabled a comparison of protein expression levels across human and pig organs. To the best of our knowledge, this is the first metanalysis study for pig at a protein expression level, in this case using label-free DDA data. The resource PaxDB version 5.0^14^ includes pig data generated using spectral counting, but its granularity is limited, providing only organ-specific expression for the liver.

As done before, we reanalyzed each data set separately using MaxQuant and the same protein sequence database. The disadvantage of this approach is that the FDR statistical thresholds are applied at a data set level and not to all data sets together as a whole. However, as also explained before,^6,7^ using a data set per data set analysis approach is in our view the only sustainable manner to reanalyze and integrate quantitative proteomics data sets in resources such as Expression Atlas, where gene expression data sets are stored following the same data set per data set approach. It is also important to highlight that the number of commonly detected protein false positives is reduced in parallel with the increase in the number of common data sets where a given protein is detected. In this case, for proteins detected in four or more data sets, a protein FDR of less than 1% can be inferred (Figure S1 in Supporting File 1).

This overall study of protein expression in pigs and its comparison with human protein expression are relevant in different contexts. First of all, systems biology research on the domestic pig is of immediate relevance for food production and animal welfare. Additionally, domestic pigs are a model organism for human biomedical research. Furthermore, the diversity of the available pig models is rapidly expanding. Some possible applications of these models are research in nutrition, inflammation, and host–microbial crosstalk. In this context, pig models present great opportunities because the pig, like humans, is an omnivore with very similar nutritional requirements, digestive and immune systems, and gut microbial components.^12^ It is also important to highlight that minipig models are being increasingly used in drug development. Animals are still requested in the safety testing of new drug candidates, and minipigs are a potential nonrodent alternative to the use of nonhuman primates (NHP) due to ethical considerations.^42^ The use of alternative in vitro models is still challenging due to complex biological responses in various organ systems following drug treatment. Therefore, it is important to have access to protein expression information in pig organs (and also ideally in mini-pig; there are still very few mini-pig proteomics studies in the public domain) so that comparisons in protein abundance across different organs and species (especially between pig, mini-pig, and human) can be performed.

Future directions in analogous studies will involve (i) the inclusion of additional species, e.g., other model organisms or other species of economic importance; (ii) studies focused on particular diseases or physiological states; (iii) the inclusion of differential proteomics data sets in addition to baseline studies; and (iv) reanalysis of DIA data sets (e.g., ref (43)). In conclusion, we present here a meta-analysis study of public pig baseline proteomics data sets from the PRIDE database. We also performed a comparative analysis across human and pig protein abundances. The resulting protein expression data has been made available via Expression Atlas.

## 5 Data Availability

Expression Atlas E-PROT identifiers and the PRIDE original data set identifiers used for the reanalysis are included in Table 1.

## Supporting Information Available

Distribution of common reverse decoy hits across the number of data sets; organ specificity of canonical proteins in pig; correlation between gene (RNA-seq based) and protein expression in baseline tissue pig data sets; correlation of protein abundances across organ liver compared between PaxDB and the results found in this study; median protein abundances (in ppb) for each protein group across various tissue samples included in each organ; median binned protein abundances across various tissue samples in each pig organ; median binned protein abundances across various pig data sets; organ distribution of canonical proteins in pig; gene ontology enrichment analysis of “organ-enriched” and “group-enriched” proteins; median binned protein abundances of human and pig orthologs across all organs; elevated proteomes of three different groups in various organs after applying edit distance between human and pig homologous genes; gene ontology enrichment analysis of the three different groups of proteins considering their level of expression in pig and human tissues; protein abundances (ppb) considering only one-to-one mapping between human and pig orthologs across all organs; and the binned protein abundances of all one-to-one mapped orthologs across ten common organs in human and pig (ZIP)

## Abbreviations

DDA: data-dependent acquisition
DIA: data-independent acquisition
FDR: false discovery rate
GO: gene ontology
iBAQ: intensity-based absolute quantification
IDF: investigation description format
MS: mass spectrometry
NHP: non-human primate
ppb: parts per billion
PCA: principal component analysis
SDRF: sample and data relationship format
UMAP: uniform manifold approximation and projection
