# Supplementary material for: Integrated Proteomics Analysis of Baseline Protein Expression in Pig Tissues
Source: J Proteome Res. 2024 May 8;23(6):1948–59. doi: 10.1021/acs.jproteome.3c00741 (PMC11165573; doi:10.1021/acs.jproteome.3c00741)
Supplement: Supplementary file 1 — pr3c00741_si_001.zip [file pr3c00741_si_001.zip › Supporting File 1.pdf]

Integrated Proteomics analysis of baseline protein expression in pig tissues

Shengbo Wang[1#], Andrew Collins[3#], Ananth Prakash[1,2#], Silvie Fexova[1], Irene Papatheodorou[1,2], Andrew R. Jones[3*], Juan Antonio Vizcaíno[1,2*]

[1] European Molecular Biology Laboratory - European Bioinformatics Institute (EMBL-EBI), Wellcome Genome Campus, Hinxton, Cambridge, CB10 1SD. United Kingdom.

[2] Open Targets, Wellcome Genome Campus, Hinxton, Cambridge, CB10 1SD. United Kingdom.

[3] Institute of Systems, Molecular and Integrative Biology, University of Liverpool, Liverpool L69 7ZB, United Kingdom.

*Corresponding authors.

#All three authors have contributed equally, and they wish to be considered as joint first authors.

Prof. Andrew R. Jones. Institute of Systems, Molecular and Integrative Biology, University of Liverpool, Liverpool L69 7ZB, United Kingdom. Email: Andrew.Jones@liverpool.ac.uk.

Dr. Juan Antonio Vizcaíno. European Molecular Biology Laboratory, European Bioinformatics Institute (EMBL-EBI), Wellcome Trust Genome Campus, Hinxton, Cambridge, CB10 1SD, UK. Email: juan@ebi.ac.uk.

TABLE OF CONTENT

- Supporting File 1:
    - Figure S1: Distribution of common reverse decoy hits across the number of datasets.
    - Figure S2: Organ specificity of canonical proteins in pig.
    - Figure S3: Correlation between gene (RNA-seq based) and protein expression in baseline tissue pig datasets.
    - Figure S4: Correlation of protein abundances across organ liver compared between PaxDB and the results found in this study.
- Supporting File 2:
    - Table S1: Median protein abundances (in ppb) for each protein group across various tissue samples included in each organ.
    - Table S2: Median binned protein abundances across various tissue samples in each pig organ.
    - Table S3: Median binned protein abundances across various pig datasets.
    - Table S4: Organ distribution of canonical proteins in pig.
    - Table S5: Gene Ontology enrichment analysis of 'organ-enriched' and 'group-enriched' proteins.
    - Table S6: Median binned protein abundances of human and pig orthologs across all organs.
    - Table S7: Elevated proteomes of three different groups in various organs after applying edit distance between human and pig homologous genes.
    - Table S8: Gene Ontology enrichment analysis of the three different groups of proteins, considering their level of expression in pig and human tissues.

- Table S9: Protein abundances (ppb) considering only one to one mapping between human and pig orthologs across all organs.

- Supporting File 3:
  - Figure S5: Figure illustrating the binned protein abundances of all one-to-one mapped orthologs across ten common organs in human and pig.

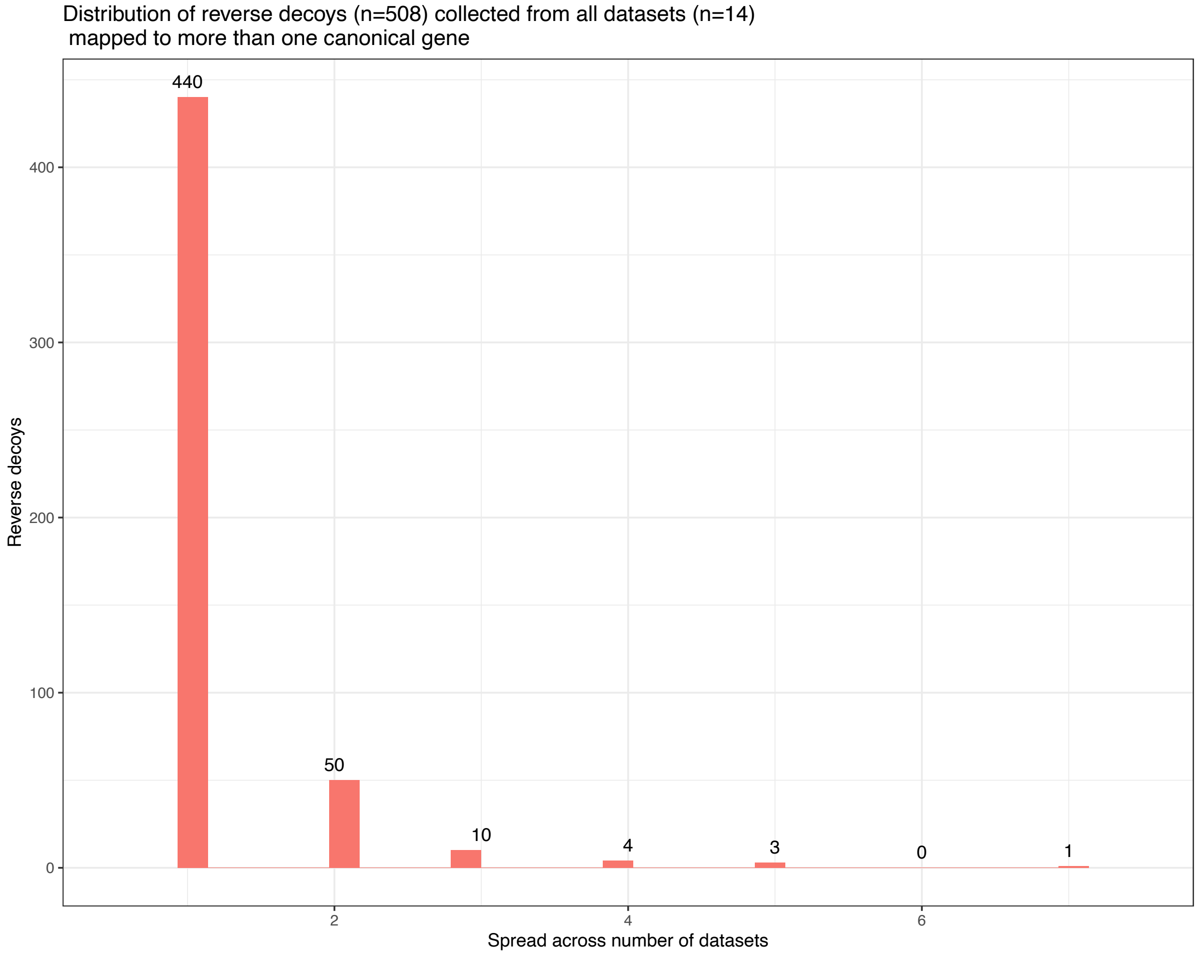

Figure S1. Distribution of common reverse decoy hits across the number of datasets.

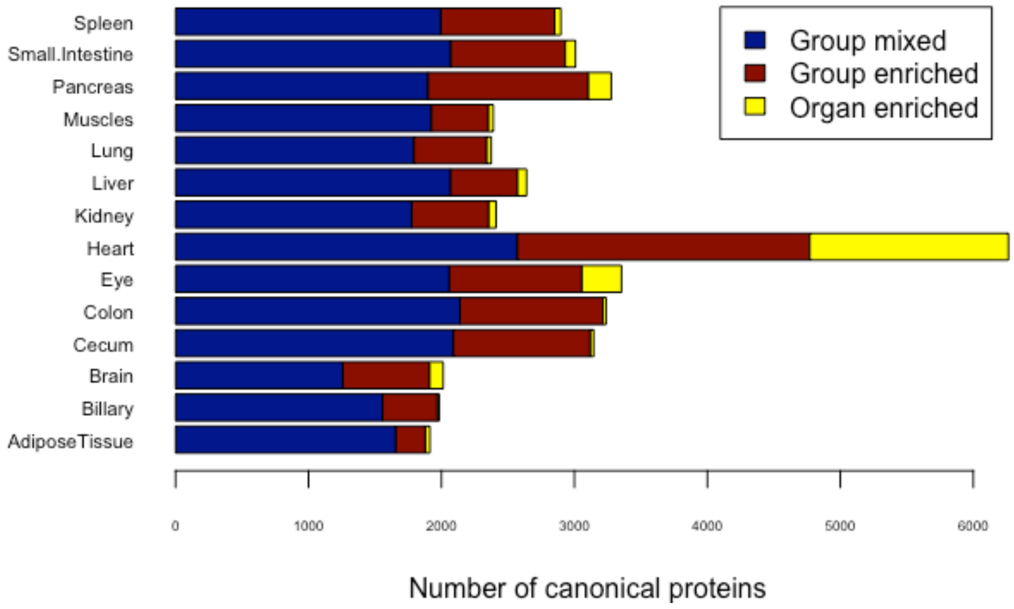

Figure S2. Organ specificity of canonical proteins in pig.

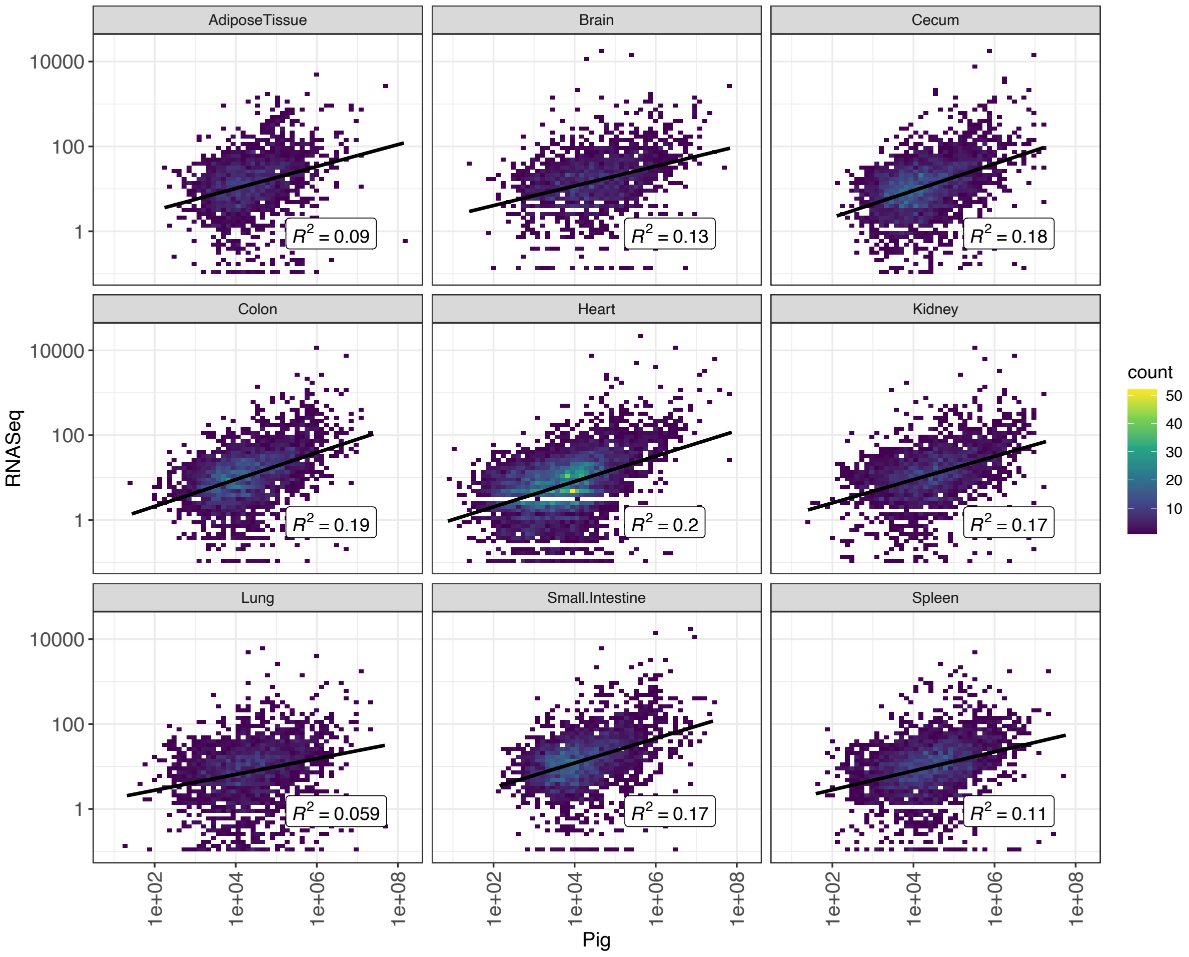

Figure S3. Correlation between gene (RNA-seq based) and protein expression in baseline tissue pig datasets.

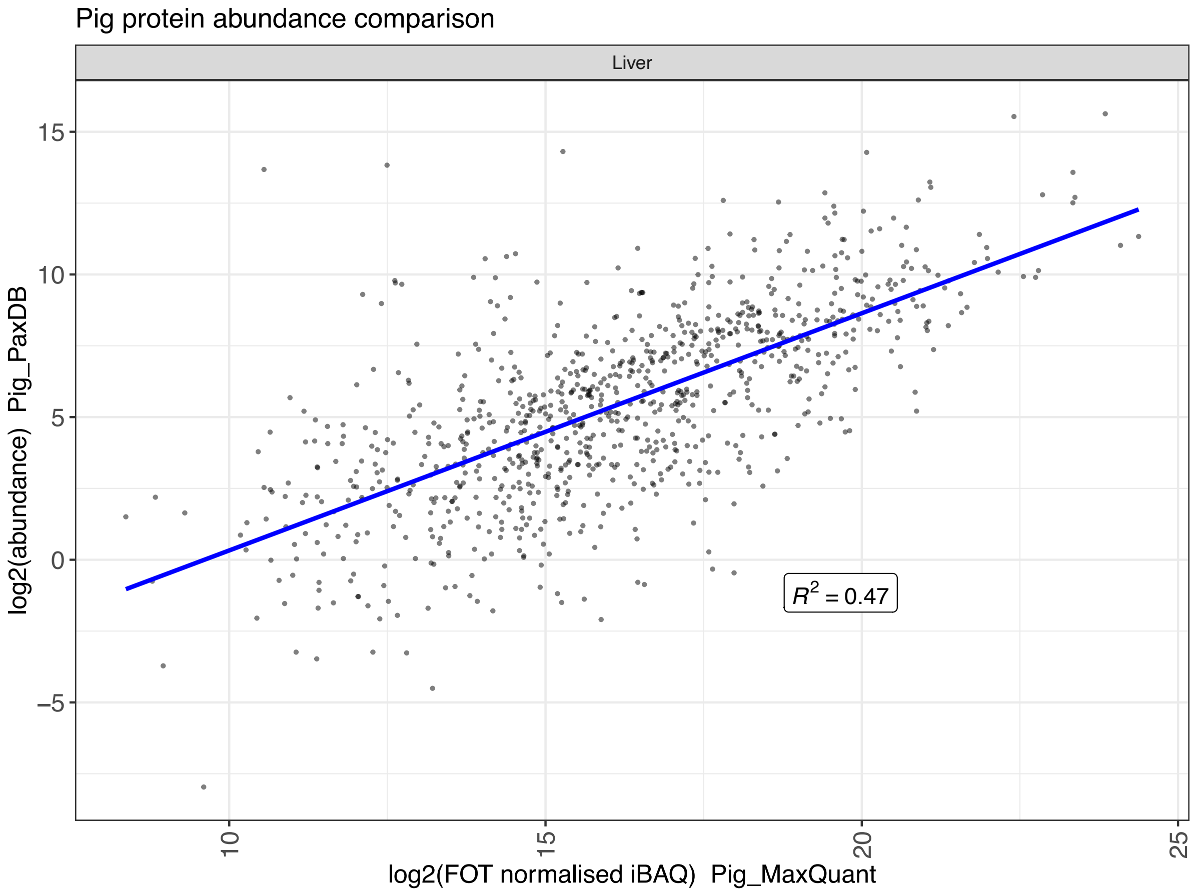

Figure S4. Correlation of protein abundances across organ liver compared between PaxDB and this study.
