# Supplementary material for: Integrated Proteomics Analysis of Baseline Protein Expression in Pig Tissues
Source: J Proteome Res. 2024 May 8;23(6):1948–59. doi: 10.1021/acs.jproteome.3c00741 (PMC11165573; doi:10.1021/acs.jproteome.3c00741)
Supplement: Supplementary file 1 — pr3c00741_si_001.zip [file pr3c00741_si_001.zip › Supporting File 3.pdf]

Integrated Proteomics analysis of baseline protein expression in pig tissues

Shengbo Wang1#, Andrew Collins3#, Ananth Prakash1,2#, Silvie Fexova1, Irene Papatheodorou1,2, Andrew R. Jones3*, Juan Antonio Vizcaíno1,2*

1 European Molecular Biology Laboratory - European Bioinformatics Institute (EMBL-EBI), Wellcome Genome Campus, Hinxton, Cambridge, CB10 1SD. United Kingdom.

2 Open Targets, Wellcome Genome Campus, Hinxton, Cambridge, CB10 1SD. United Kingdom.

3 Institute of Systems, Molecular and Integrative Biology, University of Liverpool, Liverpool L69 7ZB, United Kingdom.

*Corresponding authors.

#All three authors have contributed equally, and they wish to be considered as joint first authors.

Prof. Andrew R. Jones. Institute of Systems, Molecular and Integrative Biology, University of Liverpool, Liverpool L69 7ZB, United Kingdom. Email: Andrew.Jones@liverpool.ac.uk.

Dr. Juan Antonio Vizcaíno. European Molecular Biology Laboratory, European Bioinformatics Institute (EMBL-EBI), Wellcome Trust Genome Campus, Hinxton, Cambridge, CB10 1SD, UK. Email: juan@ebi.ac.uk.

Figure S5. Figure illustrating the binned protein abundances of all one-to-one mapped orthologs across ten common organs in human and pig.

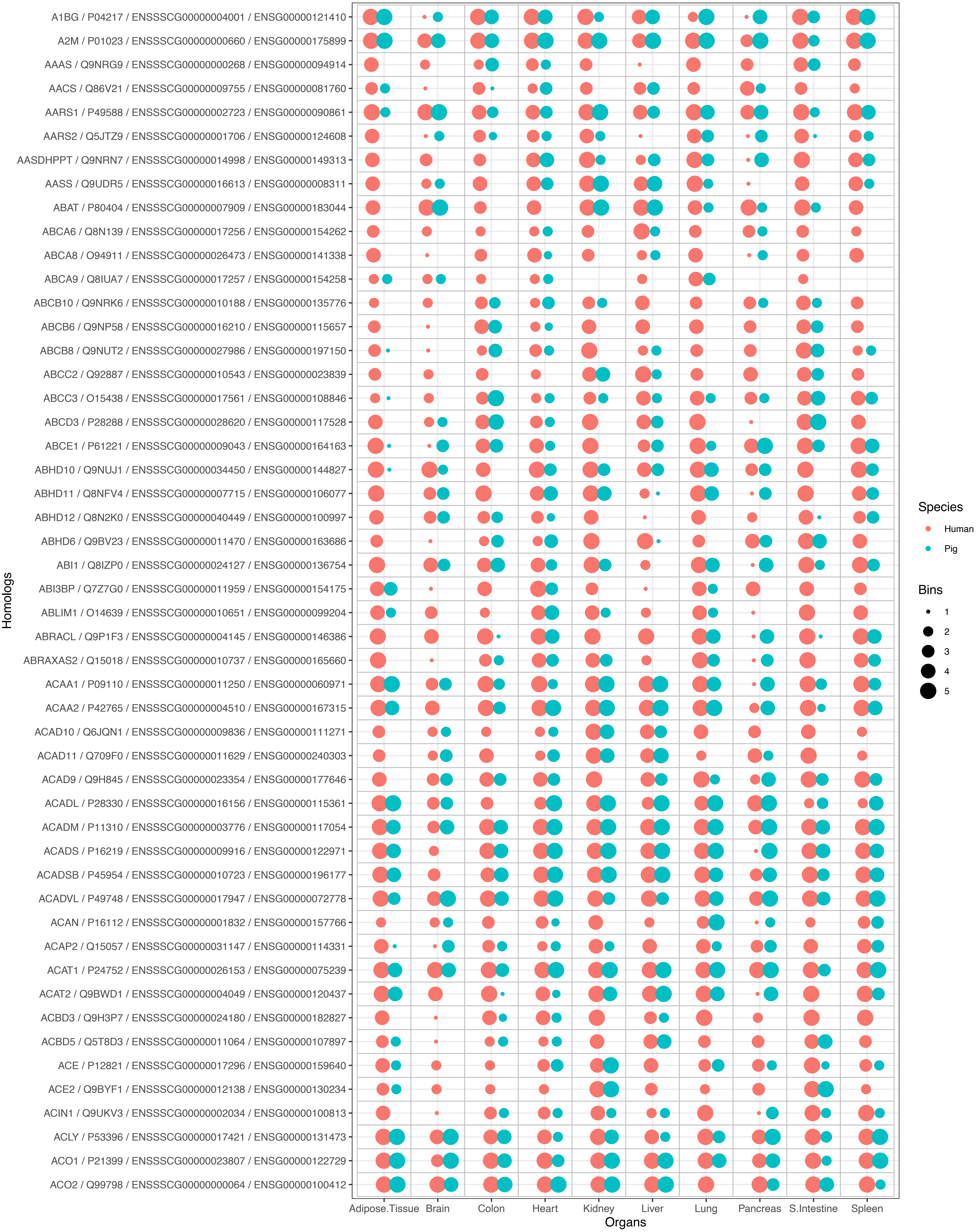
A1BG / P04217 / ENSSSCG00000004001 / ENSG00000121410
A2M / P01023 / ENSSSCG00000000660 / ENSG00000175899
AAAS / Q9NRG9 / ENSSSCG00000000268 / ENSG00000094914
AACS / Q86V21 / ENSSSCG00000009755 / ENSG00000081760
AARS1 / P49588 / ENSSSCG00000002723 / ENSG00000090861
AARS2 / Q5JTZ9 / ENSSSCG00000001706 / ENSG00000124608
AASDHPPT / Q9NRN7 / ENSSSCG00000014998 / ENSG00000149313
AASS / Q9UDR5 / ENSSSCG00000016613 / ENSG00000008311
ABAT / P80404 / ENSSSCG00000007909 / ENSG00000183044
ABCA6 / Q8N139 / ENSSSCG00000017256 / ENSG00000154262
ABCA8 / O94911 / ENSSSCG00000026473 / ENSG00000141338
ABCA9 / Q8IUA7 / ENSSSCG00000017257 / ENSG00000154258
ABCB10 / Q9NRK6 / ENSSSCG00000010188 / ENSG00000135776
ABCB6 / Q9NP58 / ENSSSCG00000016210 / ENSG00000115657
ABCB8 / Q9NUT2 / ENSSSCG00000027986 / ENSG00000197150
ABCC2 / Q92887 / ENSSSCG00000010543 / ENSG00000023839
ABCC3 / O15438 / ENSSSCG00000017561 / ENSG00000108846
ABCD3 / P28288 / ENSSSCG00000028620 / ENSG00000117528
ABCE1 / P61221 / ENSSSCG00000009043 / ENSG00000164163
ABHD10 / Q9NUJ1 / ENSSSCG00000034450 / ENSG00000144827
ABHD11 / Q8NFV4 / ENSSSCG00000007715 / ENSG00000106077
ABHD12 / Q8N2K0 / ENSSSCG00000040449 / ENSG00000100997
ABHD6 / Q9BV23 / ENSSSCG00000011470 / ENSG00000163686
ABI1 / Q8IZP0 / ENSSSCG00000024127 / ENSG00000136754
ABI3BP / Q7Z7G0 / ENSSSCG00000011959 / ENSG00000154175
ABLIM1 / O14639 / ENSSSCG00000010651 / ENSG00000099204
ABRACL / Q9P1F3 / ENSSSCG00000004145 / ENSG00000146386
ABRAXAS2 / Q15018 / ENSSSCG00000010737 / ENSG00000165660
ACAA1 / P09110 / ENSSSCG00000011250 / ENSG00000060971
ACAA2 / P42765 / ENSSSCG00000004510 / ENSG00000167315
ACAD10 / Q6JQN1 / ENSSSCG00000009836 / ENSG00000111271
ACAD11 / Q709F0 / ENSSSCG00000011629 / ENSG00000240303
ACAD9 / Q9H845 / ENSSSCG00000023354 / ENSG00000177646
ACADL / P28330 / ENSSSCG00000016156 / ENSG00000115361
ACADM / P11310 / ENSSSCG00000003776 / ENSG00000117054
ACADS / P16219 / ENSSSCG00000009916 / ENSG00000122971
ACADSB / P45954 / ENSSSCG00000010723 / ENSG00000196177
ACADVL / P49748 / ENSSSCG00000017947 / ENSG00000072778
ACAN / P16112 / ENSSSCG00000001832 / ENSG00000157766
ACAP2 / Q15057 / ENSSSCG00000031147 / ENSG00000114331
ACAT1 / P24752 / ENSSSCG00000026153 / ENSG00000075239
ACAT2 / Q9BWD1 / ENSSSCG00000004049 / ENSG00000120437
ACBD3 / Q9H3P7 / ENSSSCG00000024180 / ENSG00000182827
ACBD5 / Q5T8D3 / ENSSSCG00000011064 / ENSG00000107897
ACE / P12821 / ENSSSCG00000017296 / ENSG00000159640
ACE2 / Q9BYF1 / ENSSSCG00000012138 / ENSG00000130234
ACIN1 / Q9UKV3 / ENSSSCG00000002034 / ENSG00000100813
ACLY / P53396 / ENSSSCG00000017421 / ENSG00000131473
ACO1 / P21399 / ENSSSCG00000023807 / ENSG00000122729
ACO2 / Q99798 / ENSSSCG00000000064 / ENSG00000100412
Homologs
Adipose.Tissue
Brain
Colon
Heart
Kidney
Liver
Lung
Pancreas
S.Intestine
Spleen
Organs
Species
Human
Pig
Bins
1
2
3
4
5

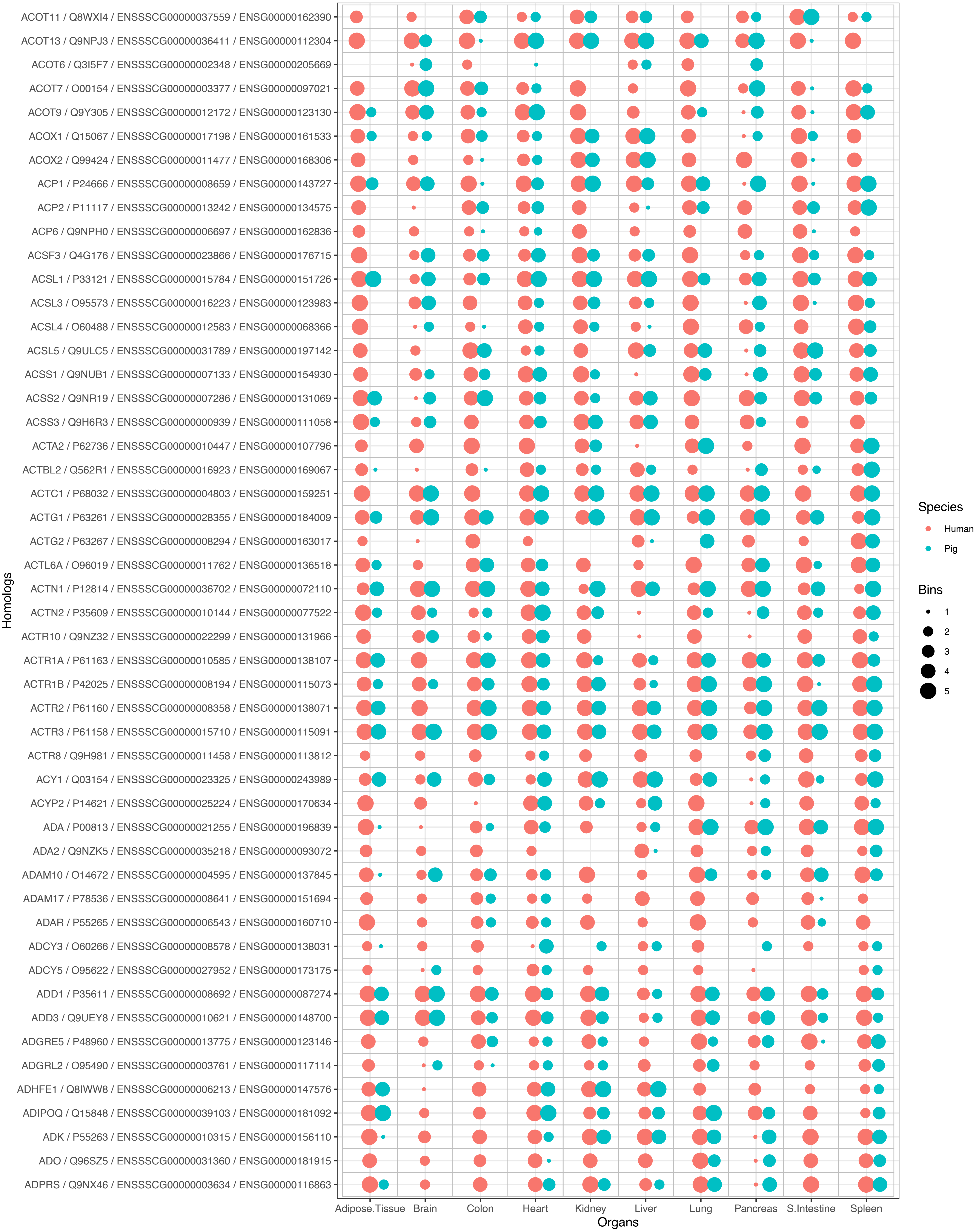
ACOT11 / Q8WXI4 / ENSSSCG00000037559 / ENSG00000162390
ACOT13 / Q9NPJ3 / ENSSSCG00000036411 / ENSG00000112304
ACOT6 / Q3I5F7 / ENSSSCG00000002348 / ENSG00000205669
ACOT7 / O00154 / ENSSSCG00000003377 / ENSG00000097021
ACOT9 / Q9Y305 / ENSSSCG00000012172 / ENSG00000123130
ACOX1 / Q15067 / ENSSSCG00000017198 / ENSG00000161533
ACOX2 / Q99424 / ENSSSCG00000011477 / ENSG00000168306
ACP1 / P24666 / ENSSSCG00000008659 / ENSG00000143727
ACP2 / P11117 / ENSSSCG00000013242 / ENSG00000134575
ACP6 / Q9NPH0 / ENSSSCG00000006697 / ENSG00000162836
ACSF3 / Q4G176 / ENSSSCG00000023866 / ENSG00000176715
ACSL1 / P33121 / ENSSSCG00000015784 / ENSG00000151726
ACSL3 / O95573 / ENSSSCG00000016223 / ENSG00000123983
ACSL4 / O60488 / ENSSSCG00000012583 / ENSG00000068366
ACSL5 / Q9ULC5 / ENSSSCG00000031789 / ENSG00000197142
ACSS1 / Q9NUB1 / ENSSSCG00000007133 / ENSG00000154930
ACSS2 / Q9NR19 / ENSSSCG00000007286 / ENSG00000131069
ACSS3 / Q9H6R3 / ENSSSCG00000000939 / ENSG00000111058
ACTA2 / P62736 / ENSSSCG00000010447 / ENSG00000107796
ACTBL2 / Q562R1 / ENSSSCG00000016923 / ENSG00000169067
ACTC1 / P68032 / ENSSSCG00000004803 / ENSG00000159251
ACTG1 / P63261 / ENSSSCG00000028355 / ENSG00000184009
ACTG2 / P63267 / ENSSSCG00000008294 / ENSG00000163017
ACTL6A / O96019 / ENSSSCG00000011762 / ENSG00000136518
ACTN1 / P12814 / ENSSSCG00000036702 / ENSG00000072110
ACTN2 / P35609 / ENSSSCG00000010144 / ENSG00000077522
ACTR10 / Q9NZ32 / ENSSSCG00000022299 / ENSG00000131966
ACTR1A / P61163 / ENSSSCG00000010585 / ENSG00000138107
ACTR1B / P42025 / ENSSSCG00000008194 / ENSG00000115073
ACTR2 / P61160 / ENSSSCG00000008358 / ENSG00000138071
ACTR3 / P61158 / ENSSSCG00000015710 / ENSG00000115091
ACTR8 / Q9H981 / ENSSSCG00000011458 / ENSG00000113812
ACY1 / Q03154 / ENSSSCG00000023325 / ENSG00000243989
ACYP2 / P14621 / ENSSSCG00000025224 / ENSG00000170634
ADA / P00813 / ENSSSCG00000021255 / ENSG00000196839
ADA2 / Q9NZK5 / ENSSSCG00000035218 / ENSG00000093072
ADAM10 / O14672 / ENSSSCG00000004595 / ENSG00000137845
ADAM17 / P78536 / ENSSSCG00000008641 / ENSG00000151694
ADAR / P55265 / ENSSSCG00000006543 / ENSG00000160710
ADCY3 / O60266 / ENSSSCG00000008578 / ENSG00000138031
ADCY5 / O95622 / ENSSSCG00000027952 / ENSG00000173175
ADD1 / P35611 / ENSSSCG00000008692 / ENSG00000087274
ADD3 / Q9UEY8 / ENSSSCG00000010621 / ENSG00000148700
ADGRE5 / P48960 / ENSSSCG00000013775 / ENSG00000123146
ADGRL2 / O95490 / ENSSSCG00000003761 / ENSG00000117114
ADHFE1 / Q8IWW8 / ENSSSCG00000006213 / ENSG00000147576
ADIPOQ / Q15848 / ENSSSCG00000039103 / ENSG00000181092
ADK / P55263 / ENSSSCG00000010315 / ENSG00000156110
ADO / Q96SZ5 / ENSSSCG00000031360 / ENSG00000181915
ADPRS / Q9NX46 / ENSSSCG00000003634 / ENSG00000116863
Homologs
Adipose.Tissue
Brain
Colon
Heart
Kidney
Liver
Lung
Pancreas
S.Intestine
Spleen
Organs
Species
Human
Pig
Bins
1
2
3
4
5

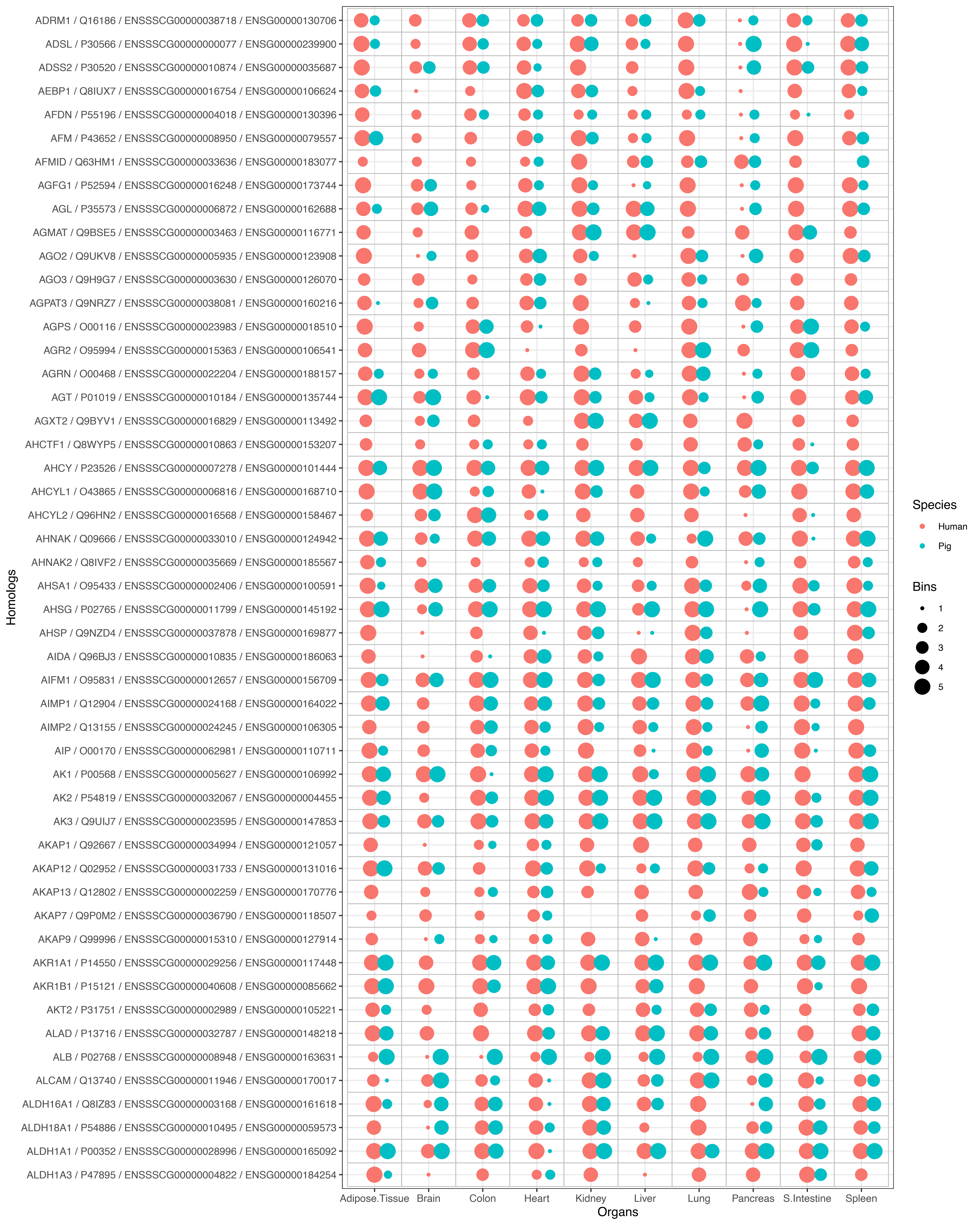
ADRM1 / Q16186 / ENSSSCG00000038718 / ENSG00000130706
ADSL / P30566 / ENSSSCG00000000077 / ENSG00000239900
ADSS2 / P30520 / ENSSSCG00000010874 / ENSG00000035687
AEBP1 / Q8IUX7 / ENSSSCG00000016754 / ENSG00000106624
AFDN / P55196 / ENSSSCG00000004018 / ENSG00000130396
AFM / P43652 / ENSSSCG00000008950 / ENSG00000079557
AFMID / Q63HM1 / ENSSSCG00000033636 / ENSG00000183077
AGFG1 / P52594 / ENSSSCG00000016248 / ENSG00000173744
AGL / P35573 / ENSSSCG00000006872 / ENSG00000162688
AGMAT / Q9BSE5 / ENSSSCG00000003463 / ENSG00000116771
AGO2 / Q9UKV8 / ENSSSCG00000005935 / ENSG00000123908
AGO3 / Q9H9G7 / ENSSSCG00000003630 / ENSG00000126070
AGPAT3 / Q9NRZ7 / ENSSSCG00000038081 / ENSG00000160216
AGPS / O00116 / ENSSSCG00000023983 / ENSG00000018510
AGR2 / O95994 / ENSSSCG00000015363 / ENSG00000106541
AGRN / O00468 / ENSSSCG00000022204 / ENSG00000188157
AGT / P01019 / ENSSSCG00000010184 / ENSG00000135744
AGXT2 / Q9BYV1 / ENSSSCG00000016829 / ENSG00000113492
AHCTF1 / Q8WYP5 / ENSSSCG00000010863 / ENSG00000153207
AHCY / P23526 / ENSSSCG00000007278 / ENSG00000101444
AHCYL1 / O43865 / ENSSSCG00000006816 / ENSG00000168710
AHCYL2 / Q96HN2 / ENSSSCG00000016568 / ENSG00000158467
AHNAK / Q09666 / ENSSSCG00000033010 / ENSG00000124942
AHNAK2 / Q8IVF2 / ENSSSCG00000035669 / ENSG00000185567
AHSA1 / O95433 / ENSSSCG00000002406 / ENSG00000100591
AHSG / P02765 / ENSSSCG00000011799 / ENSG00000145192
AHSP / Q9NZD4 / ENSSSCG00000037878 / ENSG00000169877
AIDA / Q96BJ3 / ENSSSCG00000010835 / ENSG00000186063
AIFM1 / O95831 / ENSSSCG00000012657 / ENSG00000156709
AIMP1 / Q12904 / ENSSSCG00000024168 / ENSG00000164022
AIMP2 / Q13155 / ENSSSCG00000024245 / ENSG00000106305
AIP / O00170 / ENSSSCG00000062981 / ENSG00000110711
AK1 / P00568 / ENSSSCG00000005627 / ENSG00000106992
AK2 / P54819 / ENSSSCG00000032067 / ENSG00000004455
AK3 / Q9UIJ7 / ENSSSCG00000023595 / ENSG00000147853
AKAP1 / Q92667 / ENSSSCG00000034994 / ENSG00000121057
AKAP12 / Q02952 / ENSSSCG00000031733 / ENSG00000131016
AKAP13 / Q12802 / ENSSSCG00000002259 / ENSG00000170776
AKAP7 / Q9P0M2 / ENSSSCG00000036790 / ENSG00000118507
AKAP9 / Q99996 / ENSSSCG00000015310 / ENSG00000127914
AKR1A1 / P14550 / ENSSSCG00000029256 / ENSG00000117448
AKR1B1 / P15121 / ENSSSCG00000040608 / ENSG00000085662
AKT2 / P31751 / ENSSSCG00000002989 / ENSG00000105221
ALAD / P13716 / ENSSSCG00000032787 / ENSG00000148218
ALB / P02768 / ENSSSCG00000008948 / ENSG00000163631
ALCAM / Q13740 / ENSSSCG00000011946 / ENSG00000170017
ALDH16A1 / Q8IZ83 / ENSSSCG00000003168 / ENSG00000161618
ALDH18A1 / P54886 / ENSSSCG00000010495 / ENSG00000059573
ALDH1A1 / P00352 / ENSSSCG00000028996 / ENSG00000165092
ALDH1A3 / P47895 / ENSSSCG00000004822 / ENSG00000184254
Homologs
Adipose.Tissue
Brain
Colon
Heart
Kidney
Liver
Lung
Pancreas
S.Intestine
Spleen
Organs
Species
Human
Pig
Bins
1
2
3
4
5

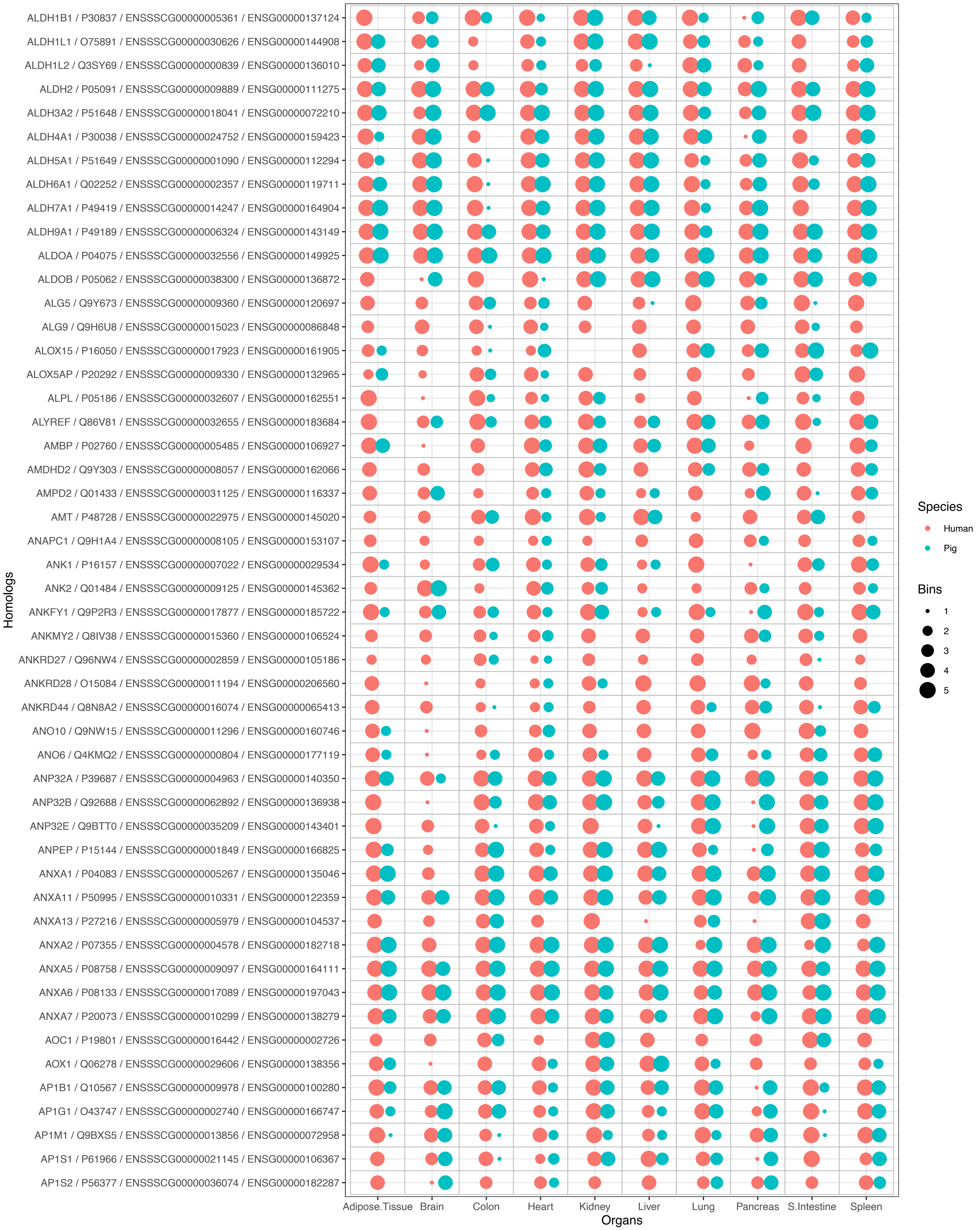

ALDH1B1 / P30837 / ENSSSCG00000005361 / ENSG00000137124
ALDH1L1 / O75891 / ENSSSCG00000030626 / ENSG00000144908
ALDH1L2 / Q3SY69 / ENSSSCG00000000839 / ENSG00000136010
ALDH2 / P05091 / ENSSSCG00000009889 / ENSG00000111275
ALDH3A2 / P51648 / ENSSSCG00000018041 / ENSG00000072210
ALDH4A1 / P30038 / ENSSSCG00000024752 / ENSG00000159423
ALDH5A1 / P51649 / ENSSSCG00000001090 / ENSG00000112294
ALDH6A1 / Q02252 / ENSSSCG00000002357 / ENSG00000119711
ALDH7A1 / P49419 / ENSSSCG00000014247 / ENSG00000164904
ALDH9A1 / P49189 / ENSSSCG00000006324 / ENSG00000143149
ALDOA / P04075 / ENSSSCG00000032556 / ENSG00000149925
ALDOB / P05062 / ENSSSCG00000038300 / ENSG00000136872
ALG5 / Q9Y673 / ENSSSCG00000009360 / ENSG00000120697
ALG9 / Q9H6U8 / ENSSSCG00000015023 / ENSG00000086848
ALOX15 / P16050 / ENSSSCG00000017923 / ENSG00000161905
ALOX5AP / P20292 / ENSSSCG00000009330 / ENSG00000132965
ALPL / P05186 / ENSSSCG00000032607 / ENSG00000162551
ALYREF / Q86V81 / ENSSSCG00000032655 / ENSG00000183684
AMBP / P02760 / ENSSSCG00000005485 / ENSG00000106927
AMDHD2 / Q9Y303 / ENSSSCG00000008057 / ENSG00000162066
AMPD2 / Q01433 / ENSSSCG00000031125 / ENSG00000116337
AMT / P48728 / ENSSSCG00000022975 / ENSG00000145020
ANAPC1 / Q9H1A4 / ENSSSCG00000008105 / ENSG00000153107
ANK1 / P16157 / ENSSSCG00000007022 / ENSG00000029534
ANK2 / Q01484 / ENSSSCG00000009125 / ENSG00000145362
ANKFY1 / Q9P2R3 / ENSSSCG00000017877 / ENSG00000185722
ANKMY2 / Q8IV38 / ENSSSCG00000015360 / ENSG00000106524
ANKRD27 / Q96NW4 / ENSSSCG00000002859 / ENSG00000105186
ANKRD28 / O15084 / ENSSSCG00000011194 / ENSG00000206560
ANKRD44 / Q8N8A2 / ENSSSCG00000016074 / ENSG00000065413
ANO10 / Q9NW15 / ENSSSCG00000011296 / ENSG00000160746
ANO6 / Q4KMQ2 / ENSSSCG00000000804 / ENSG00000177119
ANP32A / P39687 / ENSSSCG00000004963 / ENSG00000140350
ANP32B / Q92688 / ENSSSCG00000062892 / ENSG00000136938
ANP32E / Q9BTT0 / ENSSSCG00000035209 / ENSG00000143401
ANPEP / P15144 / ENSSSCG00000001849 / ENSG00000166825
ANXA1 / P04083 / ENSSSCG00000005267 / ENSG00000135046
ANXA11 / P50995 / ENSSSCG00000010331 / ENSG00000122359
ANXA13 / P27216 / ENSSSCG00000005979 / ENSG00000104537
ANXA2 / P07355 / ENSSSCG00000004578 / ENSG00000182718
ANXA5 / P08758 / ENSSSCG00000009097 / ENSG00000164111
ANXA6 / P08133 / ENSSSCG00000017089 / ENSG00000197043
ANXA7 / P20073 / ENSSSCG00000010299 / ENSG00000138279
AOC1 / P19801 / ENSSSCG00000016442 / ENSG00000002726
AOX1 / Q06278 / ENSSSCG00000029606 / ENSG00000138356
AP1B1 / Q10567 / ENSSSCG00000009978 / ENSG00000100280
AP1G1 / O43747 / ENSSSCG00000002740 / ENSG00000166747
AP1M1 / Q9BXS5 / ENSSSCG00000013856 / ENSG00000072958
AP1S1 / P61966 / ENSSSCG00000021145 / ENSG00000106367
AP1S2 / P56377 / ENSSSCG00000036074 / ENSG00000182287
Homologs
Adipose.Tissue
Brain
Colon
Heart
Kidney
Liver
Lung
Pancreas
S.Intestine
Spleen
Organs
Species
Human
Pig
Bins
1
2
3
4
5

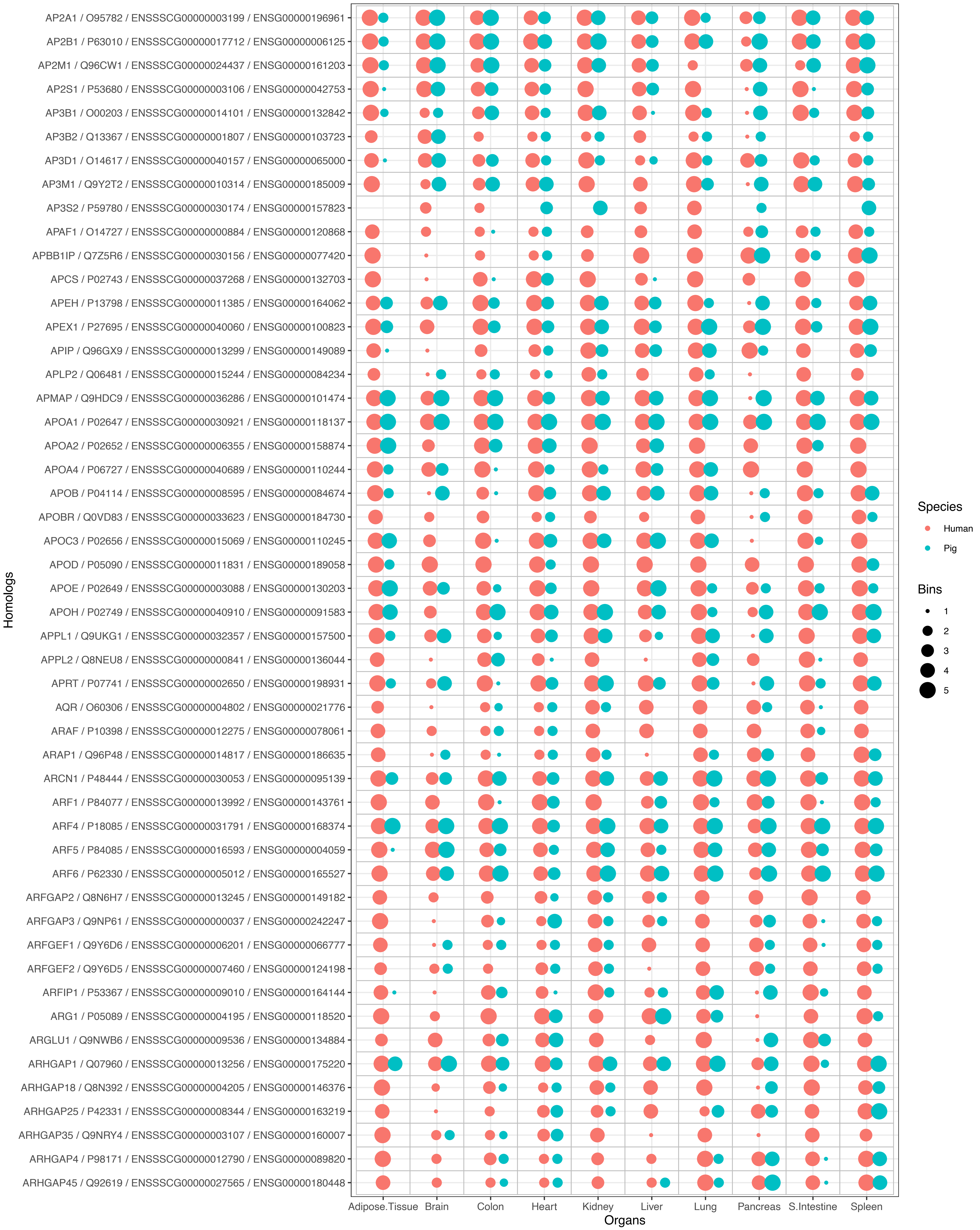
AP2A1 / O95782 / ENSSSCG00000003199 / ENSG00000196961
AP2B1 / P63010 / ENSSSCG00000017712 / ENSG00000006125
AP2M1 / Q96CW1 / ENSSSCG00000024437 / ENSG00000161203
AP2S1 / P53680 / ENSSSCG00000003106 / ENSG00000042753
AP3B1 / O00203 / ENSSSCG00000014101 / ENSG00000132842
AP3B2 / Q13367 / ENSSSCG00000001807 / ENSG00000103723
AP3D1 / O14617 / ENSSSCG00000040157 / ENSG00000065000
AP3M1 / Q9Y2T2 / ENSSSCG00000010314 / ENSG00000185009
AP3S2 / P59780 / ENSSSCG00000030174 / ENSG00000157823
APAF1 / O14727 / ENSSSCG00000000884 / ENSG00000120868
APBB1IP / Q7Z5R6 / ENSSSCG00000030156 / ENSG00000077420
APCS / P02743 / ENSSSCG00000037268 / ENSG00000132703
APEH / P13798 / ENSSSCG00000011385 / ENSG00000164062
APEX1 / P27695 / ENSSSCG00000040060 / ENSG00000100823
APIP / Q96GX9 / ENSSSCG00000013299 / ENSG00000149089
APLP2 / Q06481 / ENSSSCG00000015244 / ENSG00000084234
APMAP / Q9HDC9 / ENSSSCG00000036286 / ENSG00000101474
APOA1 / P02647 / ENSSSCG00000030921 / ENSG00000118137
APOA2 / P02652 / ENSSSCG00000006355 / ENSG00000158874
APOA4 / P06727 / ENSSSCG00000040689 / ENSG00000110244
APOB / P04114 / ENSSSCG00000008595 / ENSG00000084674
APOBR / Q0VD83 / ENSSSCG00000033623 / ENSG00000184730
APOC3 / P02656 / ENSSSCG00000015069 / ENSG00000110245
APOD / P05090 / ENSSSCG00000011831 / ENSG00000189058
APOE / P02649 / ENSSSCG00000003088 / ENSG00000130203
APOH / P02749 / ENSSSCG00000040910 / ENSG00000091583
APPL1 / Q9UKG1 / ENSSSCG00000032357 / ENSG00000157500
APPL2 / Q8NEU8 / ENSSSCG00000000841 / ENSG00000136044
APRT / P07741 / ENSSSCG00000002650 / ENSG00000198931
AQR / O60306 / ENSSSCG00000004802 / ENSG00000021776
ARAF / P10398 / ENSSSCG00000012275 / ENSG00000078061
ARAP1 / Q96P48 / ENSSSCG00000014817 / ENSG00000186635
ARCN1 / P48444 / ENSSSCG00000030053 / ENSG00000095139
ARF1 / P84077 / ENSSSCG00000013992 / ENSG00000143761
ARF4 / P18085 / ENSSSCG00000031791 / ENSG00000168374
ARF5 / P84085 / ENSSSCG00000016593 / ENSG00000004059
ARF6 / P62330 / ENSSSCG00000005012 / ENSG00000165527
ARFGAP2 / Q8N6H7 / ENSSSCG00000013245 / ENSG00000149182
ARFGAP3 / Q9NP61 / ENSSSCG00000000037 / ENSG00000242247
ARFGEF1 / Q9Y6D6 / ENSSSCG00000006201 / ENSG00000066777
ARFGEF2 / Q9Y6D5 / ENSSSCG00000007460 / ENSG00000124198
ARFIP1 / P53367 / ENSSSCG00000009010 / ENSG00000164144
ARG1 / P05089 / ENSSSCG00000004195 / ENSG00000118520
ARGLU1 / Q9NWB6 / ENSSSCG00000009536 / ENSG00000134884
ARHGAP1 / Q07960 / ENSSSCG00000013256 / ENSG00000175220
ARHGAP18 / Q8N392 / ENSSSCG00000004205 / ENSG00000146376
ARHGAP25 / P42331 / ENSSSCG00000008344 / ENSG00000163219
ARHGAP35 / Q9NRY4 / ENSSSCG00000003107 / ENSG00000160007
ARHGAP4 / P98171 / ENSSSCG00000012790 / ENSG00000089820
ARHGAP45 / Q92619 / ENSSSCG00000027565 / ENSG00000180448
Homologs
Adipose.Tissue
Brain
Colon
Heart
Kidney
Liver
Lung
Pancreas
S.Intestine
Spleen
Organs
Species
Human
Pig
Bins
1
2
3
4
5

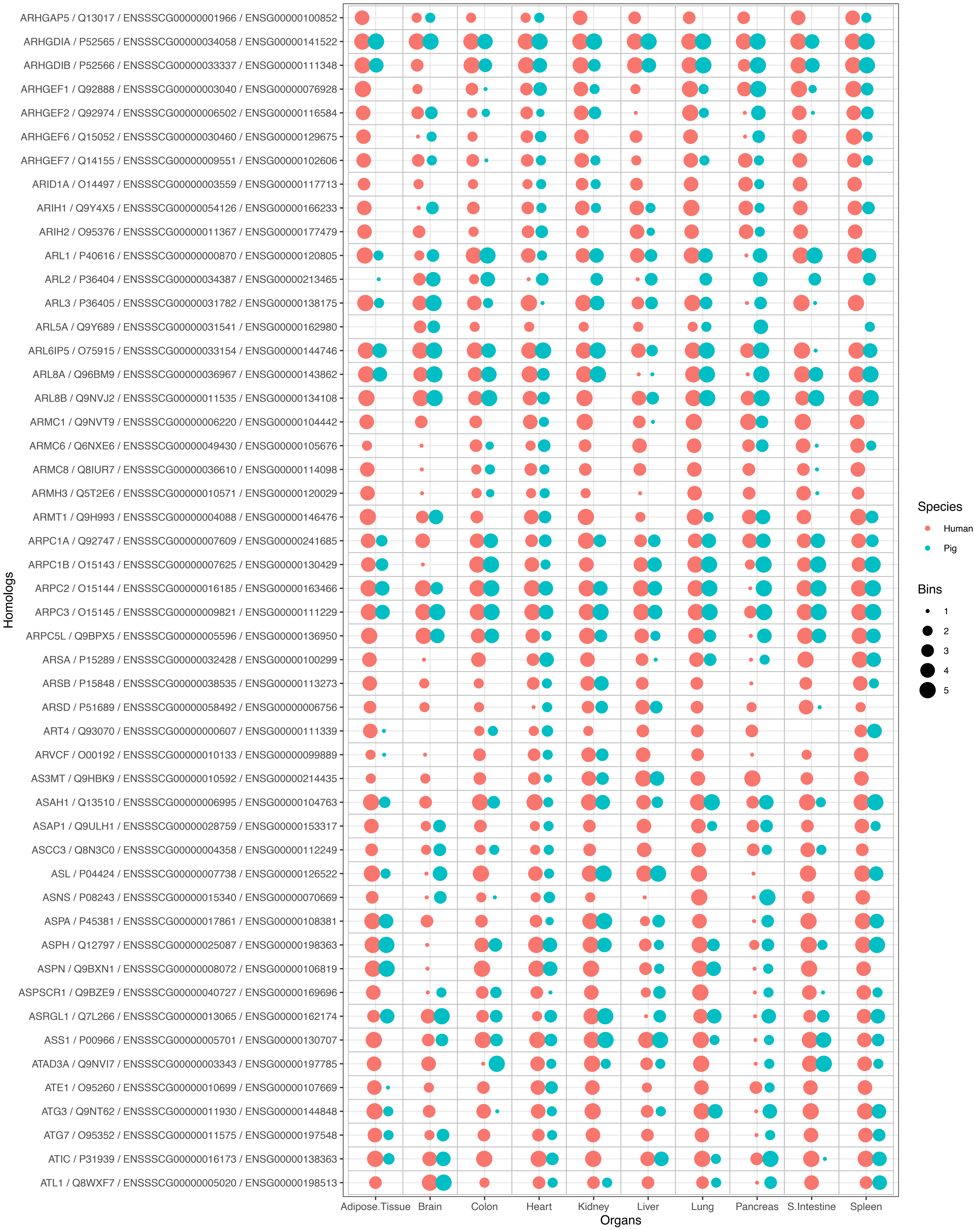
ARHGAP5 / Q13017 / ENSSSCG00000001966 / ENSG00000100852
ARHGDIA / P52565 / ENSSSCG00000034058 / ENSG00000141522
ARHGDIB / P52566 / ENSSSCG00000033337 / ENSG00000111348
ARHGEF1 / Q92888 / ENSSSCG00000003040 / ENSG00000076928
ARHGEF2 / Q92974 / ENSSSCG00000006502 / ENSG00000116584
ARHGEF6 / Q15052 / ENSSSCG00000030460 / ENSG00000129675
ARHGEF7 / Q14155 / ENSSSCG00000009551 / ENSG00000102606
ARID1A / O14497 / ENSSSCG00000003559 / ENSG00000117713
ARIH1 / Q9Y4X5 / ENSSSCG00000054126 / ENSG00000166233
ARIH2 / O95376 / ENSSSCG00000011367 / ENSG00000177479
ARL1 / P40616 / ENSSSCG00000000870 / ENSG00000120805
ARL2 / P36404 / ENSSSCG00000034387 / ENSG00000213465
ARL3 / P36405 / ENSSSCG00000031782 / ENSG00000138175
ARL5A / Q9Y689 / ENSSSCG00000031541 / ENSG00000162980
ARL6IP5 / O75915 / ENSSSCG00000033154 / ENSG00000144746
ARL8A / Q96BM9 / ENSSSCG00000036967 / ENSG00000143862
ARL8B / Q9NVJ2 / ENSSSCG00000011535 / ENSG00000134108
ARMC1 / Q9NVT9 / ENSSSCG00000006220 / ENSG00000104442
ARMC6 / Q6NXE6 / ENSSSCG00000049430 / ENSG00000105676
ARMC8 / Q8IUR7 / ENSSSCG00000036610 / ENSG00000114098
ARMH3 / Q5T2E6 / ENSSSCG00000010571 / ENSG00000120029
ARMT1 / Q9H993 / ENSSSCG00000004088 / ENSG00000146476
ARPC1A / Q92747 / ENSSSCG00000007609 / ENSG00000241685
ARPC1B / O15143 / ENSSSCG00000007625 / ENSG00000130429
ARPC2 / O15144 / ENSSSCG00000016185 / ENSG00000163466
ARPC3 / O15145 / ENSSSCG00000009821 / ENSG00000111229
ARPC5L / Q9BPX5 / ENSSSCG00000005596 / ENSG00000136950
ARSA / P15289 / ENSSSCG00000032428 / ENSG00000100299
ARSB / P15848 / ENSSSCG00000038535 / ENSG00000113273
ARSD / P51689 / ENSSSCG00000058492 / ENSG00000006756
ART4 / Q93070 / ENSSSCG00000000607 / ENSG00000111339
ARVCF / O00192 / ENSSSCG00000010133 / ENSG00000099889
AS3MT / Q9HBK9 / ENSSSCG00000010592 / ENSG00000214435
ASAH1 / Q13510 / ENSSSCG00000006995 / ENSG00000104763
ASAP1 / Q9ULH1 / ENSSSCG00000028759 / ENSG00000153317
ASCC3 / Q8N3C0 / ENSSSCG00000004358 / ENSG00000112249
ASL / P04424 / ENSSSCG00000007738 / ENSG00000126522
ASNS / P08243 / ENSSSCG00000015340 / ENSG00000070669
ASPA / P45381 / ENSSSCG00000017861 / ENSG00000108381
ASPH / Q12797 / ENSSSCG00000025087 / ENSG00000198363
ASPN / Q9BXN1 / ENSSSCG00000008072 / ENSG00000106819
ASPSCR1 / Q9BZE9 / ENSSSCG00000040727 / ENSG00000169696
ASRGL1 / Q7L266 / ENSSSCG00000013065 / ENSG00000162174
ASS1 / P00966 / ENSSSCG00000005701 / ENSG00000130707
ATAD3A / Q9NVI7 / ENSSSCG00000003343 / ENSG00000197785
ATE1 / O95260 / ENSSSCG00000010699 / ENSG00000107669
ATG3 / Q9NT62 / ENSSSCG00000011930 / ENSG00000144848
ATG7 / O95352 / ENSSSCG00000011575 / ENSG00000197548
ATIC / P31939 / ENSSSCG00000016173 / ENSG00000138363
ATL1 / Q8WXF7 / ENSSSCG00000005020 / ENSG00000198513
Homologs
Adipose.Tissue
Brain
Colon
Heart
Kidney
Liver
Lung
Pancreas
S.Intestine
Spleen
Organs
Species
Human
Pig
Bins
1
2
3
4
5

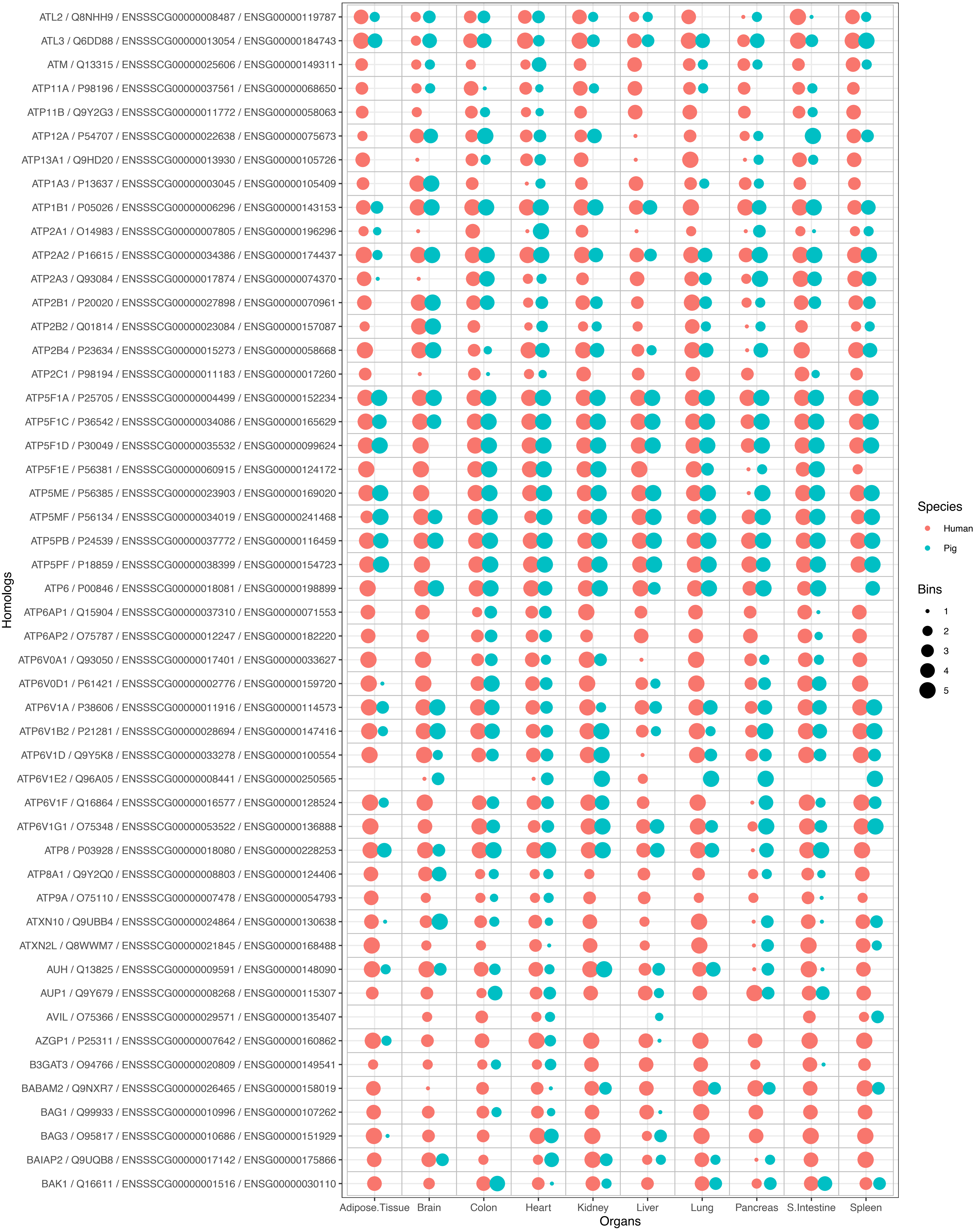

ATL2 / Q8NHH9 / ENSSSCG00000008487 / ENSG00000119787
ATL3 / Q6DD88 / ENSSSCG00000013054 / ENSG00000184743
ATM / Q13315 / ENSSSCG00000025606 / ENSG00000149311
ATP11A / P98196 / ENSSSCG00000037561 / ENSG00000068650
ATP11B / Q9Y2G3 / ENSSSCG00000011772 / ENSG00000058063
ATP12A / P54707 / ENSSSCG00000022638 / ENSG00000075673
ATP13A1 / Q9HD20 / ENSSSCG00000013930 / ENSG00000105726
ATP1A3 / P13637 / ENSSSCG00000003045 / ENSG00000105409
ATP1B1 / P05026 / ENSSSCG00000006296 / ENSG00000143153
ATP2A1 / O14983 / ENSSSCG00000007805 / ENSG00000196296
ATP2A2 / P16615 / ENSSSCG00000034386 / ENSG00000174437
ATP2A3 / Q93084 / ENSSSCG00000017874 / ENSG00000074370
ATP2B1 / P20020 / ENSSSCG00000027898 / ENSG00000070961
ATP2B2 / Q01814 / ENSSSCG00000023084 / ENSG00000157087
ATP2B4 / P23634 / ENSSSCG00000015273 / ENSG00000058668
ATP2C1 / P98194 / ENSSSCG00000011183 / ENSG00000017260
ATP5F1A / P25705 / ENSSSCG00000004499 / ENSG00000152234
ATP5F1C / P36542 / ENSSSCG00000034086 / ENSG00000165629
ATP5F1D / P30049 / ENSSSCG00000035532 / ENSG00000099624
ATP5F1E / P56381 / ENSSSCG00000060915 / ENSG00000124172
ATP5ME / P56385 / ENSSSCG00000023903 / ENSG00000169020
ATP5MF / P56134 / ENSSSCG00000034019 / ENSG00000241468
ATP5PB / P24539 / ENSSSCG00000037772 / ENSG00000116459
ATP5PF / P18859 / ENSSSCG00000038399 / ENSG00000154723
ATP6 / P00846 / ENSSSCG00000018081 / ENSG00000198899
ATP6AP1 / Q15904 / ENSSSCG00000037310 / ENSG00000071553
ATP6AP2 / O75787 / ENSSSCG00000012247 / ENSG00000182220
ATP6V0A1 / Q93050 / ENSSSCG00000017401 / ENSG00000033627
ATP6V0D1 / P61421 / ENSSSCG00000002776 / ENSG00000159720
ATP6V1A / P38606 / ENSSSCG00000011916 / ENSG00000114573
ATP6V1B2 / P21281 / ENSSSCG00000028694 / ENSG00000147416
ATP6V1D / Q9Y5K8 / ENSSSCG00000033278 / ENSG00000100554
ATP6V1E2 / Q96A05 / ENSSSCG00000008441 / ENSG00000250565
ATP6V1F / Q16864 / ENSSSCG00000016577 / ENSG00000128524
ATP6V1G1 / O75348 / ENSSSCG00000053522 / ENSG00000136888
ATP8 / P03928 / ENSSSCG00000018080 / ENSG00000228253
ATP8A1 / Q9Y2Q0 / ENSSSCG00000008803 / ENSG00000124406
ATP9A / O75110 / ENSSSCG00000007478 / ENSG00000054793
ATXN10 / Q9UBB4 / ENSSSCG00000024864 / ENSG00000130638
ATXN2L / Q8WWM7 / ENSSSCG00000021845 / ENSG00000168488
AUH / Q13825 / ENSSSCG00000009591 / ENSG00000148090
AUP1 / Q9Y679 / ENSSSCG00000008268 / ENSG00000115307
AVIL / O75366 / ENSSSCG00000029571 / ENSG00000135407
AZGP1 / P25311 / ENSSSCG00000007642 / ENSG00000160862
B3GAT3 / O94766 / ENSSSCG00000020809 / ENSG00000149541
BABAM2 / Q9NXR7 / ENSSSCG00000026465 / ENSG00000158019
BAG1 / Q99933 / ENSSSCG00000010996 / ENSG00000107262
BAG3 / O95817 / ENSSSCG00000010686 / ENSG00000151929
BAIAP2 / Q9UQB8 / ENSSSCG00000017142 / ENSG00000175866
BAK1 / Q16611 / ENSSSCG00000001516 / ENSG00000030110
Homologs
Adipose.Tissue
Brain
Colon
Heart
Kidney
Liver
Lung
Pancreas
S.Intestine
Spleen
Organs
Species
Human
Pig
Bins
1
2
3
4
5

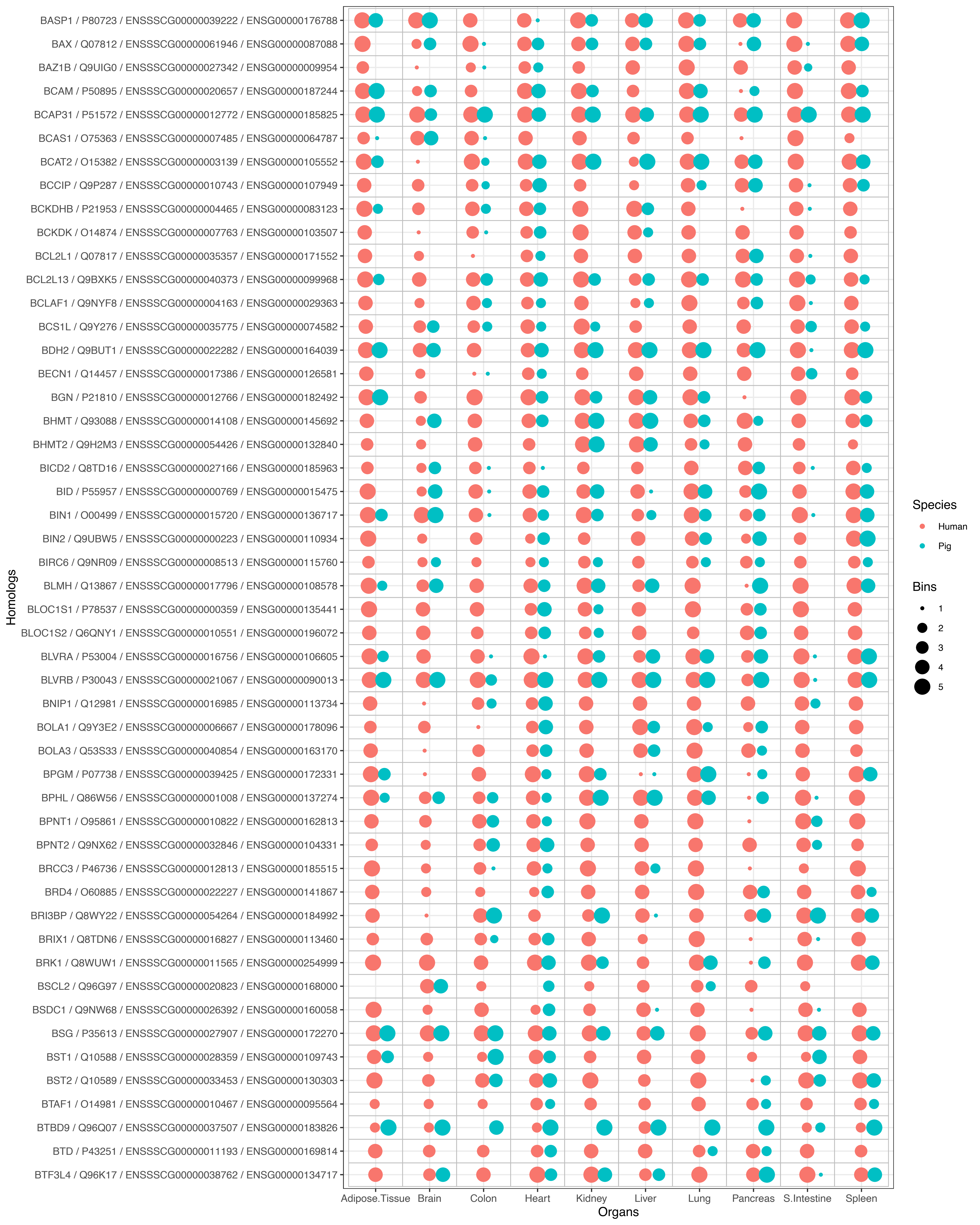

BASP1 / P80723 / ENSSSCG00000039222 / ENSG00000176788
BAX / Q07812 / ENSSSCG00000061946 / ENSG00000087088
BAZ1B / Q9UIG0 / ENSSSCG00000027342 / ENSG00000009954
BCAM / P50895 / ENSSSCG00000020657 / ENSG00000187244
BCAP31 / P51572 / ENSSSCG00000012772 / ENSG00000185825
BCAS1 / O75363 / ENSSSCG00000007485 / ENSG00000064787
BCAT2 / O15382 / ENSSSCG00000003139 / ENSG00000105552
BCCIP / Q9P287 / ENSSSCG00000010743 / ENSG00000107949
BCKDHB / P21953 / ENSSSCG00000004465 / ENSG00000083123
BCKDK / O14874 / ENSSSCG00000007763 / ENSG00000103507
BCL2L1 / Q07817 / ENSSSCG00000035357 / ENSG00000171552
BCL2L13 / Q9BXK5 / ENSSSCG00000040373 / ENSG00000099968
BCLAF1 / Q9NYF8 / ENSSSCG00000004163 / ENSG00000029363
BCS1L / Q9Y276 / ENSSSCG00000035775 / ENSG00000074582
BDH2 / Q9BUT1 / ENSSSCG00000022282 / ENSG00000164039
BECN1 / Q14457 / ENSSSCG00000017386 / ENSG00000126581
BGN / P21810 / ENSSSCG00000012766 / ENSG00000182492
BHMT / Q93088 / ENSSSCG00000014108 / ENSG00000145692
BHMT2 / Q9H2M3 / ENSSSCG00000054426 / ENSG00000132840
BICD2 / Q8TD16 / ENSSSCG00000027166 / ENSG00000185963
BID / P55957 / ENSSSCG00000000769 / ENSG00000015475
BIN1 / O00499 / ENSSSCG00000015720 / ENSG00000136717
BIN2 / Q9UBW5 / ENSSSCG00000000223 / ENSG00000110934
BIRC6 / Q9NR09 / ENSSSCG00000008513 / ENSG00000115760
BLMH / Q13867 / ENSSSCG00000017796 / ENSG00000108578
BLOC1S1 / P78537 / ENSSSCG00000000359 / ENSG00000135441
BLOC1S2 / Q6QNY1 / ENSSSCG00000010551 / ENSG00000196072
BLVRA / P53004 / ENSSSCG00000016756 / ENSG00000106605
BLVRB / P30043 / ENSSSCG00000021067 / ENSG00000090013
BNIP1 / Q12981 / ENSSSCG00000016985 / ENSG00000113734
BOLA1 / Q9Y3E2 / ENSSSCG00000006667 / ENSG00000178096
BOLA3 / Q53S33 / ENSSSCG00000040854 / ENSG00000163170
BPGM / P07738 / ENSSSCG00000039425 / ENSG00000172331
BPHL / Q86W56 / ENSSSCG00000001008 / ENSG00000137274
BPNT1 / O95861 / ENSSSCG00000010822 / ENSG00000162813
BPNT2 / Q9NX62 / ENSSSCG00000032846 / ENSG00000104331
BRCC3 / P46736 / ENSSSCG00000012813 / ENSG00000185515
BRD4 / O60885 / ENSSSCG00000022227 / ENSG00000141867
BRI3BP / Q8WY22 / ENSSSCG00000054264 / ENSG00000184992
BRIX1 / Q8TDN6 / ENSSSCG00000016827 / ENSG00000113460
BRK1 / Q8WUW1 / ENSSSCG00000011565 / ENSG00000254999
BSCL2 / Q96G97 / ENSSSCG00000020823 / ENSG00000168000
BSDC1 / Q9NW68 / ENSSSCG00000026392 / ENSG00000160058
BSG / P35613 / ENSSSCG00000027907 / ENSG00000172270
BST1 / Q10588 / ENSSSCG00000028359 / ENSG00000109743
BST2 / Q10589 / ENSSSCG00000033453 / ENSG00000130303
BTAF1 / O14981 / ENSSSCG00000010467 / ENSG00000095564
BTBD9 / Q96Q07 / ENSSSCG00000037507 / ENSG00000183826
BTD / P43251 / ENSSSCG00000011193 / ENSG00000169814
BTF3L4 / Q96K17 / ENSSSCG00000038762 / ENSG00000134717
Homologs
Adipose.Tissue
Brain
Colon
Heart
Kidney
Liver
Lung
Pancreas
S.Intestine
Spleen
Organs
Species
Human
Pig
Bins
1
2
3
4
5

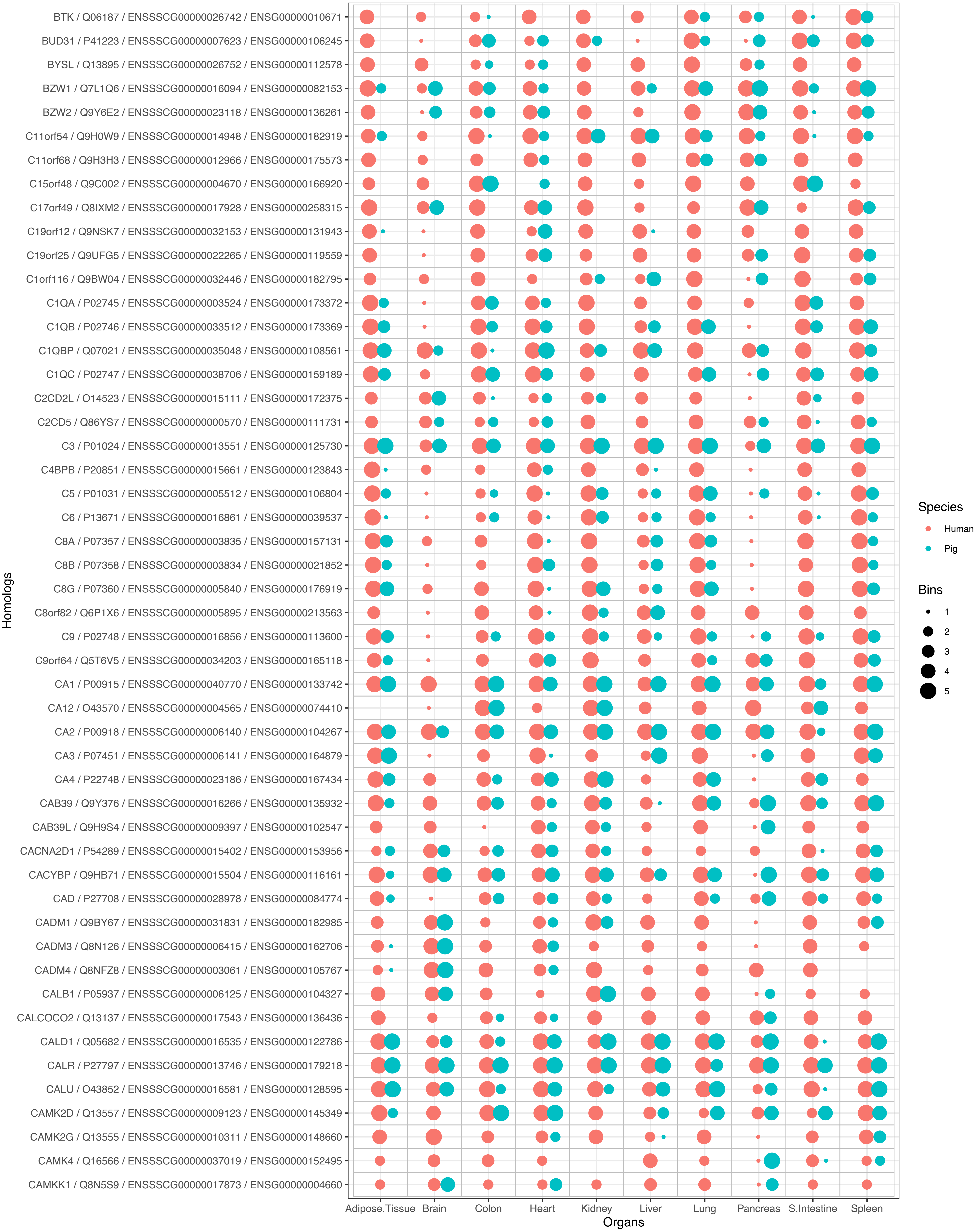

BTK / Q06187 / ENSSSCG00000026742 / ENSG00000010671
BUD31 / P41223 / ENSSSCG00000007623 / ENSG00000106245
BYSL / Q13895 / ENSSSCG00000026752 / ENSG00000112578
BZW1 / Q7L1Q6 / ENSSSCG00000016094 / ENSG00000082153
BZW2 / Q9Y6E2 / ENSSSCG00000023118 / ENSG00000136261
C11orf54 / Q9H0W9 / ENSSSCG00000014948 / ENSG00000182919
C11orf68 / Q9H3H3 / ENSSSCG00000012966 / ENSG00000175573
C15orf48 / Q9C002 / ENSSSCG00000004670 / ENSG00000166920
C17orf49 / Q8IXM2 / ENSSSCG00000017928 / ENSG00000258315
C19orf12 / Q9NSK7 / ENSSSCG00000032153 / ENSG00000131943
C19orf25 / Q9UFG5 / ENSSSCG00000022265 / ENSG00000119559
C1orf116 / Q9BW04 / ENSSSCG00000032446 / ENSG00000182795
C1QA / P02745 / ENSSSCG00000003524 / ENSG00000173372
C1QB / P02746 / ENSSSCG00000033512 / ENSG00000173369
C1QBP / Q07021 / ENSSSCG00000035048 / ENSG00000108561
C1QC / P02747 / ENSSSCG00000038706 / ENSG00000159189
C2CD2L / O14523 / ENSSSCG00000015111 / ENSG00000172375
C2CD5 / Q86YS7 / ENSSSCG00000000570 / ENSG00000111731
C3 / P01024 / ENSSSCG00000013551 / ENSG00000125730
C4BPB / P20851 / ENSSSCG00000015661 / ENSG00000123843
C5 / P01031 / ENSSSCG00000005512 / ENSG00000106804
C6 / P13671 / ENSSSCG00000016861 / ENSG00000039537
C8A / P07357 / ENSSSCG00000003835 / ENSG00000157131
C8B / P07358 / ENSSSCG00000003834 / ENSG00000021852
C8G / P07360 / ENSSSCG00000005840 / ENSG00000176919
C8orf82 / Q6P1X6 / ENSSSCG00000005895 / ENSG00000213563
C9 / P02748 / ENSSSCG00000016856 / ENSG00000113600
C9orf64 / Q5T6V5 / ENSSSCG00000034203 / ENSG00000165118
CA1 / P00915 / ENSSSCG00000040770 / ENSG00000133742
CA12 / O43570 / ENSSSCG00000004565 / ENSG00000074410
CA2 / P00918 / ENSSSCG00000006140 / ENSG00000104267
CA3 / P07451 / ENSSSCG00000006141 / ENSG00000164879
CA4 / P22748 / ENSSSCG00000023186 / ENSG00000167434
CAB39 / Q9Y376 / ENSSSCG00000016266 / ENSG00000135932
CAB39L / Q9H9S4 / ENSSSCG00000009397 / ENSG00000102547
CACNA2D1 / P54289 / ENSSSCG00000015402 / ENSG00000153956
CACYBP / Q9HB71 / ENSSSCG00000015504 / ENSG00000116161
CAD / P27708 / ENSSSCG00000028978 / ENSG00000084774
CADM1 / Q9BY67 / ENSSSCG00000031831 / ENSG00000182985
CADM3 / Q8N126 / ENSSSCG00000006415 / ENSG00000162706
CADM4 / Q8NFZ8 / ENSSSCG00000003061 / ENSG00000105767
CALB1 / P05937 / ENSSSCG00000006125 / ENSG00000104327
CALCOCO2 / Q13137 / ENSSSCG00000017543 / ENSG00000136436
CALD1 / Q05682 / ENSSSCG00000016535 / ENSG00000122786
CALR / P27797 / ENSSSCG00000013746 / ENSG00000179218
CALU / O43852 / ENSSSCG00000016581 / ENSG00000128595
CAMK2D / Q13557 / ENSSSCG00000009123 / ENSG00000145349
CAMK2G / Q13555 / ENSSSCG00000010311 / ENSG00000148660
CAMK4 / Q16566 / ENSSSCG00000037019 / ENSG00000152495
CAMKK1 / Q8N5S9 / ENSSSCG00000017873 / ENSG00000004660
Homologs
Adipose.Tissue
Brain
Colon
Heart
Kidney
Liver
Lung
Pancreas
S.Intestine
Spleen
Organs
Species
Human
Pig
Bins
1
2
3
4
5

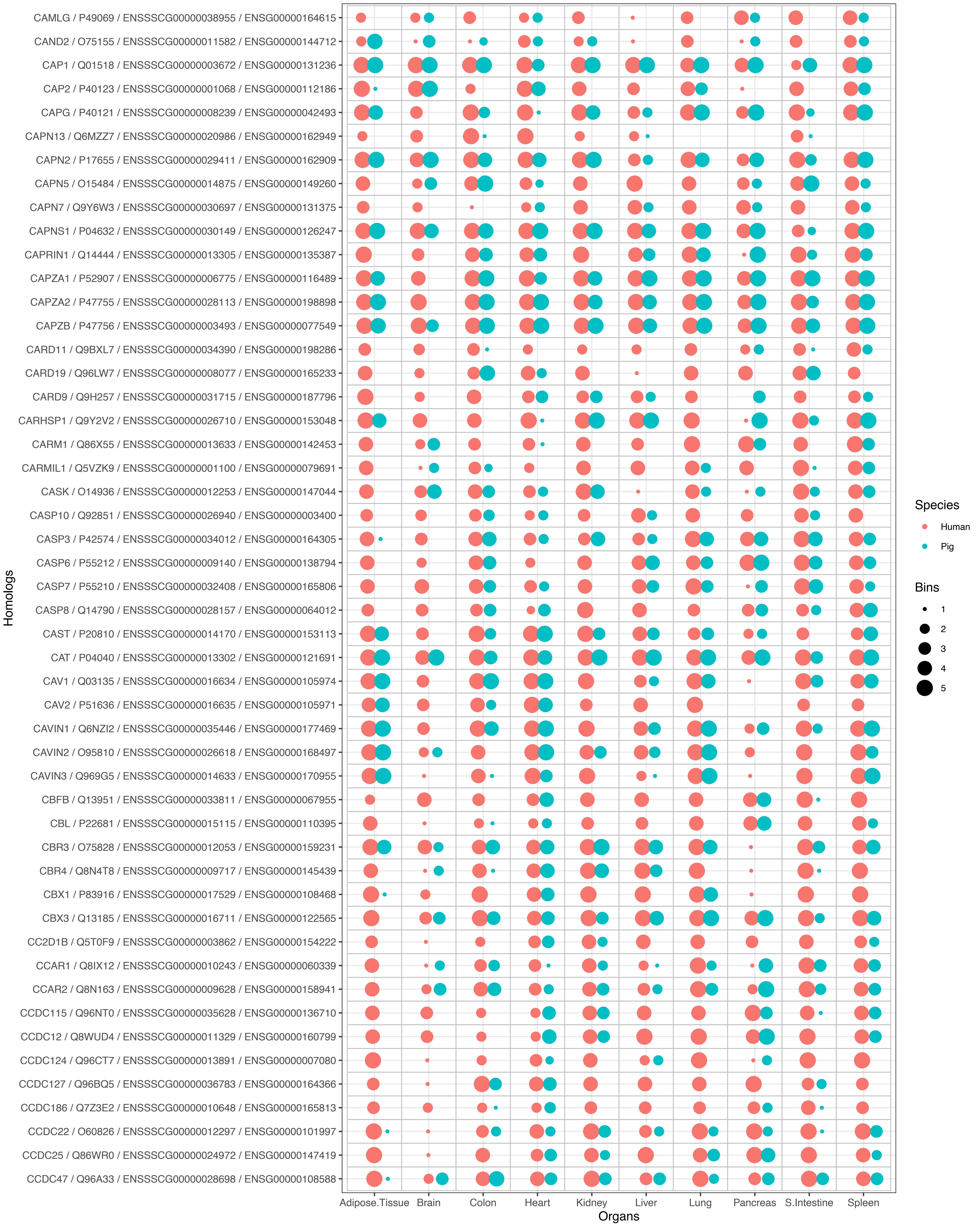

CAMLG / P49069 / ENSSSCG00000038955 / ENSG00000164615
CAND2 / O75155 / ENSSSCG00000011582 / ENSG00000144712
CAP1 / Q01518 / ENSSSCG00000003672 / ENSG00000131236
CAP2 / P40123 / ENSSSCG00000001068 / ENSG00000112186
CAPG / P40121 / ENSSSCG00000008239 / ENSG00000042493
CAPN13 / Q6MZZ7 / ENSSSCG00000020986 / ENSG00000162949
CAPN2 / P17655 / ENSSSCG00000029411 / ENSG00000162909
CAPN5 / O15484 / ENSSSCG00000014875 / ENSG00000149260
CAPN7 / Q9Y6W3 / ENSSSCG00000030697 / ENSG00000131375
CAPNS1 / P04632 / ENSSSCG00000030149 / ENSG00000126247
CAPRIN1 / Q14444 / ENSSSCG00000013305 / ENSG00000135387
CAPZA1 / P52907 / ENSSSCG00000006775 / ENSG00000116489
CAPZA2 / P47755 / ENSSSCG00000028113 / ENSG00000198898
CAPZB / P47756 / ENSSSCG00000003493 / ENSG00000077549
CARD11 / Q9BXL7 / ENSSSCG00000034390 / ENSG00000198286
CARD19 / Q96LW7 / ENSSSCG00000008077 / ENSG00000165233
CARD9 / Q9H257 / ENSSSCG00000031715 / ENSG00000187796
CARHSP1 / Q9Y2V2 / ENSSSCG00000026710 / ENSG00000153048
CARM1 / Q86X55 / ENSSSCG00000013633 / ENSG00000142453
CARMIL1 / Q5VZK9 / ENSSSCG00000001100 / ENSG00000079691
CASK / O14936 / ENSSSCG00000012253 / ENSG00000147044
CASP10 / Q92851 / ENSSSCG00000026940 / ENSG00000003400
CASP3 / P42574 / ENSSSCG00000034012 / ENSG00000164305
CASP6 / P55212 / ENSSSCG00000009140 / ENSG00000138794
CASP7 / P55210 / ENSSSCG00000032408 / ENSG00000165806
CASP8 / Q14790 / ENSSSCG00000028157 / ENSG00000064012
CAST / P20810 / ENSSSCG00000014170 / ENSG00000153113
CAT / P04040 / ENSSSCG00000013302 / ENSG00000121691
CAV1 / Q03135 / ENSSSCG00000016634 / ENSG00000105974
CAV2 / P51636 / ENSSSCG00000016635 / ENSG00000105971
CAVIN1 / Q6NZI2 / ENSSSCG00000035446 / ENSG00000177469
CAVIN2 / O95810 / ENSSSCG00000026618 / ENSG00000168497
CAVIN3 / Q969G5 / ENSSSCG00000014633 / ENSG00000170955
CBFB / Q13951 / ENSSSCG00000033811 / ENSG00000067955
CBL / P22681 / ENSSSCG00000015115 / ENSG00000110395
CBR3 / O75828 / ENSSSCG00000012053 / ENSG00000159231
CBR4 / Q8N4T8 / ENSSSCG00000009717 / ENSG00000145439
CBX1 / P83916 / ENSSSCG00000017529 / ENSG00000108468
CBX3 / Q13185 / ENSSSCG00000016711 / ENSG00000122565
CC2D1B / Q5T0F9 / ENSSSCG00000003862 / ENSG00000154222
CCAR1 / Q8IX12 / ENSSSCG00000010243 / ENSG00000060339
CCAR2 / Q8N163 / ENSSSCG00000009628 / ENSG00000158941
CCDC115 / Q96NT0 / ENSSSCG00000035628 / ENSG00000136710
CCDC12 / Q8WUD4 / ENSSSCG00000011329 / ENSG00000160799
CCDC124 / Q96CT7 / ENSSSCG00000013891 / ENSG00000007080
CCDC127 / Q96BQ5 / ENSSSCG00000036783 / ENSG00000164366
CCDC186 / Q7Z3E2 / ENSSSCG00000010648 / ENSG00000165813
CCDC22 / O60826 / ENSSSCG00000012297 / ENSG00000101997
CCDC25 / Q86WR0 / ENSSSCG00000024972 / ENSG00000147419
CCDC47 / Q96A33 / ENSSSCG00000028698 / ENSG00000108588
Homologs
Adipose.Tissue
Brain
Colon
Heart
Kidney
Liver
Lung
Pancreas
S.Intestine
Spleen
Organs
Species
Human
Pig
Bins
1
2
3
4
5

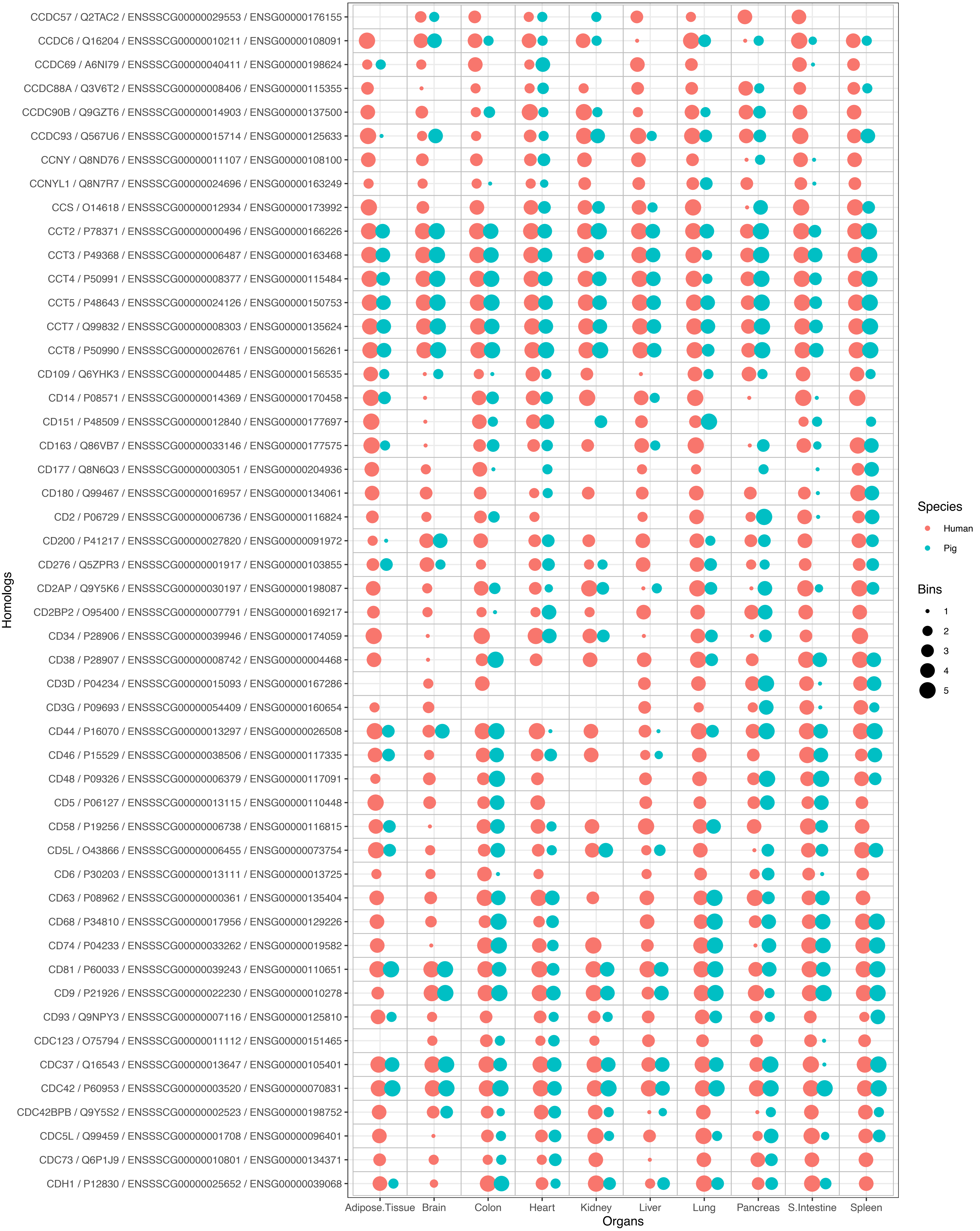

CCDC57 / Q2TAC2 / ENSSSCG00000029553 / ENSG00000176155
CCDC6 / Q16204 / ENSSSCG00000010211 / ENSG00000108091
CCDC69 / A6NI79 / ENSSSCG00000040411 / ENSG00000198624
CCDC88A / Q3V6T2 / ENSSSCG00000008406 / ENSG00000115355
CCDC90B / Q9GZT6 / ENSSSCG00000014903 / ENSG00000137500
CCDC93 / Q567U6 / ENSSSCG00000015714 / ENSG00000125633
CCNY / Q8ND76 / ENSSSCG00000011107 / ENSG00000108100
CCNYL1 / Q8N7R7 / ENSSSCG00000024696 / ENSG00000163249
CCS / O14618 / ENSSSCG00000012934 / ENSG00000173992
CCT2 / P78371 / ENSSSCG00000000496 / ENSG00000166226
CCT3 / P49368 / ENSSSCG00000006487 / ENSG00000163468
CCT4 / P50991 / ENSSSCG00000008377 / ENSG00000115484
CCT5 / P48643 / ENSSSCG00000024126 / ENSG00000150753
CCT7 / Q99832 / ENSSSCG00000008303 / ENSG00000135624
CCT8 / P50990 / ENSSSCG00000026761 / ENSG00000156261
CD109 / Q6YHK3 / ENSSSCG00000004485 / ENSG00000156535
CD14 / P08571 / ENSSSCG00000014369 / ENSG00000170458
CD151 / P48509 / ENSSSCG00000012840 / ENSG00000177697
CD163 / Q86VB7 / ENSSSCG00000033146 / ENSG00000177575
CD177 / Q8N6Q3 / ENSSSCG00000003051 / ENSG00000204936
CD180 / Q99467 / ENSSSCG00000016957 / ENSG00000134061
CD2 / P06729 / ENSSSCG00000006736 / ENSG00000116824
CD200 / P41217 / ENSSSCG00000027820 / ENSG00000091972
CD276 / Q5ZPR3 / ENSSSCG00000001917 / ENSG00000103855
CD2AP / Q9Y5K6 / ENSSSCG00000030197 / ENSG00000198087
CD2BP2 / O95400 / ENSSSCG00000007791 / ENSG00000169217
CD34 / P28906 / ENSSSCG00000039946 / ENSG00000174059
CD38 / P28907 / ENSSSCG00000008742 / ENSG00000004468
CD3D / P04234 / ENSSSCG00000015093 / ENSG00000167286
CD3G / P09693 / ENSSSCG00000054409 / ENSG00000160654
CD44 / P16070 / ENSSSCG00000013297 / ENSG00000026508
CD46 / P15529 / ENSSSCG00000038506 / ENSG00000117335
CD48 / P09326 / ENSSSCG00000006379 / ENSG00000117091
CD5 / P06127 / ENSSSCG00000013115 / ENSG00000110448
CD58 / P19256 / ENSSSCG00000006738 / ENSG00000116815
CD5L / O43866 / ENSSSCG00000006455 / ENSG00000073754
CD6 / P30203 / ENSSSCG00000013111 / ENSG00000013725
CD63 / P08962 / ENSSSCG00000000361 / ENSG00000135404
CD68 / P34810 / ENSSSCG00000017956 / ENSG00000129226
CD74 / P04233 / ENSSSCG00000033262 / ENSG00000019582
CD81 / P60033 / ENSSSCG00000039243 / ENSG00000110651
CD9 / P21926 / ENSSSCG00000022230 / ENSG00000010278
CD93 / Q9NPY3 / ENSSSCG00000007116 / ENSG00000125810
CDC123 / O75794 / ENSSSCG00000011112 / ENSG00000151465
CDC37 / Q16543 / ENSSSCG00000013647 / ENSG00000105401
CDC42 / P60953 / ENSSSCG00000003520 / ENSG00000070831
CDC42BPB / Q9Y5S2 / ENSSSCG00000002523 / ENSG00000198752
CDC5L / Q99459 / ENSSSCG00000001708 / ENSG00000096401
CDC73 / Q6P1J9 / ENSSSCG00000010801 / ENSG00000134371
CDH1 / P12830 / ENSSSCG00000025652 / ENSG00000039068
Homologs
Adipose.Tissue
Brain
Colon
Heart
Kidney
Liver
Lung
Pancreas
S.Intestine
Spleen
Organs
Species
Human
Pig
Bins
1
2
3
4
5

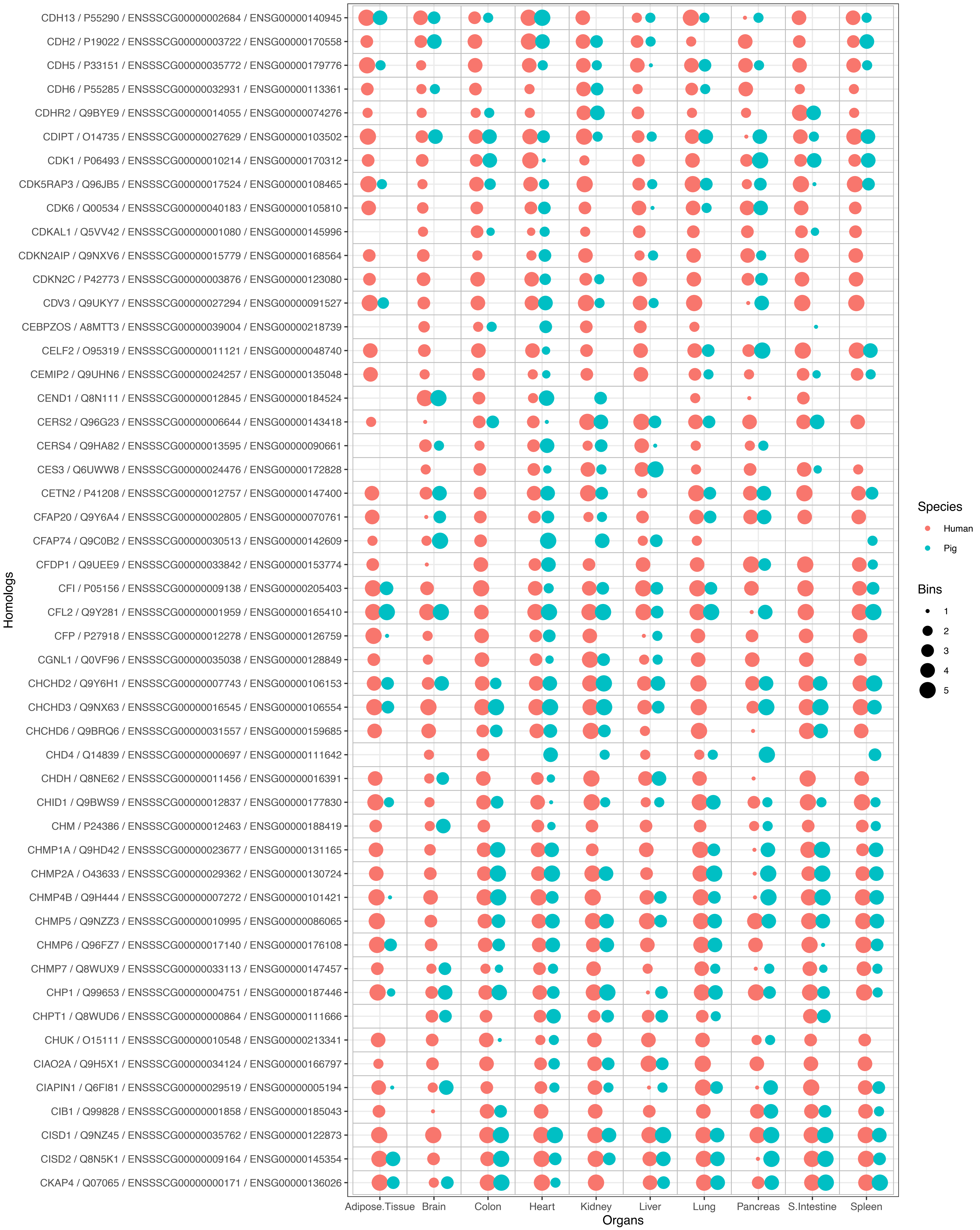

CDH13 / P55290 / ENSSSCG00000002684 / ENSG00000140945
CDH2 / P19022 / ENSSSCG00000003722 / ENSG00000170558
CDH5 / P33151 / ENSSSCG00000035772 / ENSG00000179776
CDH6 / P55285 / ENSSSCG00000032931 / ENSG00000113361
CDHR2 / Q9BYE9 / ENSSSCG00000014055 / ENSG00000074276
CDIPT / O14735 / ENSSSCG00000027629 / ENSG00000103502
CDK1 / P06493 / ENSSSCG00000010214 / ENSG00000170312
CDK5RAP3 / Q96JB5 / ENSSSCG00000017524 / ENSG00000108465
CDK6 / Q00534 / ENSSSCG00000040183 / ENSG00000105810
CDKAL1 / Q5VV42 / ENSSSCG00000001080 / ENSG00000145996
CDKN2AIP / Q9NXV6 / ENSSSCG00000015779 / ENSG00000168564
CDKN2C / P42773 / ENSSSCG00000003876 / ENSG00000123080
CDV3 / Q9UKY7 / ENSSSCG00000027294 / ENSG00000091527
CEBPZOS / A8MTT3 / ENSSSCG00000039004 / ENSG00000218739
CELF2 / O95319 / ENSSSCG00000011121 / ENSG00000048740
CEMIP2 / Q9UHN6 / ENSSSCG00000024257 / ENSG00000135048
CEND1 / Q8N111 / ENSSSCG00000012845 / ENSG00000184524
CERS2 / Q96G23 / ENSSSCG00000006644 / ENSG00000143418
CERS4 / Q9HA82 / ENSSSCG00000013595 / ENSG00000090661
CES3 / Q6UWW8 / ENSSSCG00000024476 / ENSG00000172828
CETN2 / P41208 / ENSSSCG00000012757 / ENSG00000147400
CFAP20 / Q9Y6A4 / ENSSSCG00000002805 / ENSG00000070761
CFAP74 / Q9C0B2 / ENSSSCG00000030513 / ENSG00000142609
CFDP1 / Q9UEE9 / ENSSSCG00000033842 / ENSG00000153774
CFI / P05156 / ENSSSCG00000009138 / ENSG00000205403
CFL2 / Q9Y281 / ENSSSCG00000001959 / ENSG00000165410
CFP / P27918 / ENSSSCG00000012278 / ENSG00000126759
CGNL1 / Q0VF96 / ENSSSCG00000035038 / ENSG00000128849
CHCHD2 / Q9Y6H1 / ENSSSCG00000007743 / ENSG00000106153
CHCHD3 / Q9NX63 / ENSSSCG00000016545 / ENSG00000106554
CHCHD6 / Q9BRQ6 / ENSSSCG00000031557 / ENSG00000159685
CHD4 / Q14839 / ENSSSCG00000000697 / ENSG00000111642
CHDH / Q8NE62 / ENSSSCG00000011456 / ENSG00000016391
CHID1 / Q9BWS9 / ENSSSCG00000012837 / ENSG00000177830
CHM / P24386 / ENSSSCG00000012463 / ENSG00000188419
CHMP1A / Q9HD42 / ENSSSCG00000023677 / ENSG00000131165
CHMP2A / O43633 / ENSSSCG00000029362 / ENSG00000130724
CHMP4B / Q9H444 / ENSSSCG00000007272 / ENSG00000101421
CHMP5 / Q9NZZ3 / ENSSSCG00000010995 / ENSG00000086065
CHMP6 / Q96FZ7 / ENSSSCG00000017140 / ENSG00000176108
CHMP7 / Q8WUX9 / ENSSSCG00000033113 / ENSG00000147457
CHP1 / Q99653 / ENSSSCG00000004751 / ENSG00000187446
CHPT1 / Q8WUD6 / ENSSSCG00000000864 / ENSG00000111666
CHUK / O15111 / ENSSSCG00000010548 / ENSG00000213341
CIAO2A / Q9H5X1 / ENSSSCG00000034124 / ENSG00000166797
CIAPIN1 / Q6FI81 / ENSSSCG00000029519 / ENSG00000005194
CIB1 / Q99828 / ENSSSCG00000001858 / ENSG00000185043
CISD1 / Q9NZ45 / ENSSSCG00000035762 / ENSG00000122873
CISD2 / Q8N5K1 / ENSSSCG00000009164 / ENSG00000145354
CKAP4 / Q07065 / ENSSSCG00000000171 / ENSG00000136026
Homologs
Adipose.Tissue
Brain
Colon
Heart
Kidney
Liver
Lung
Pancreas
S.Intestine
Spleen
Organs
Species
Human
Pig
Bins
1
2
3
4
5

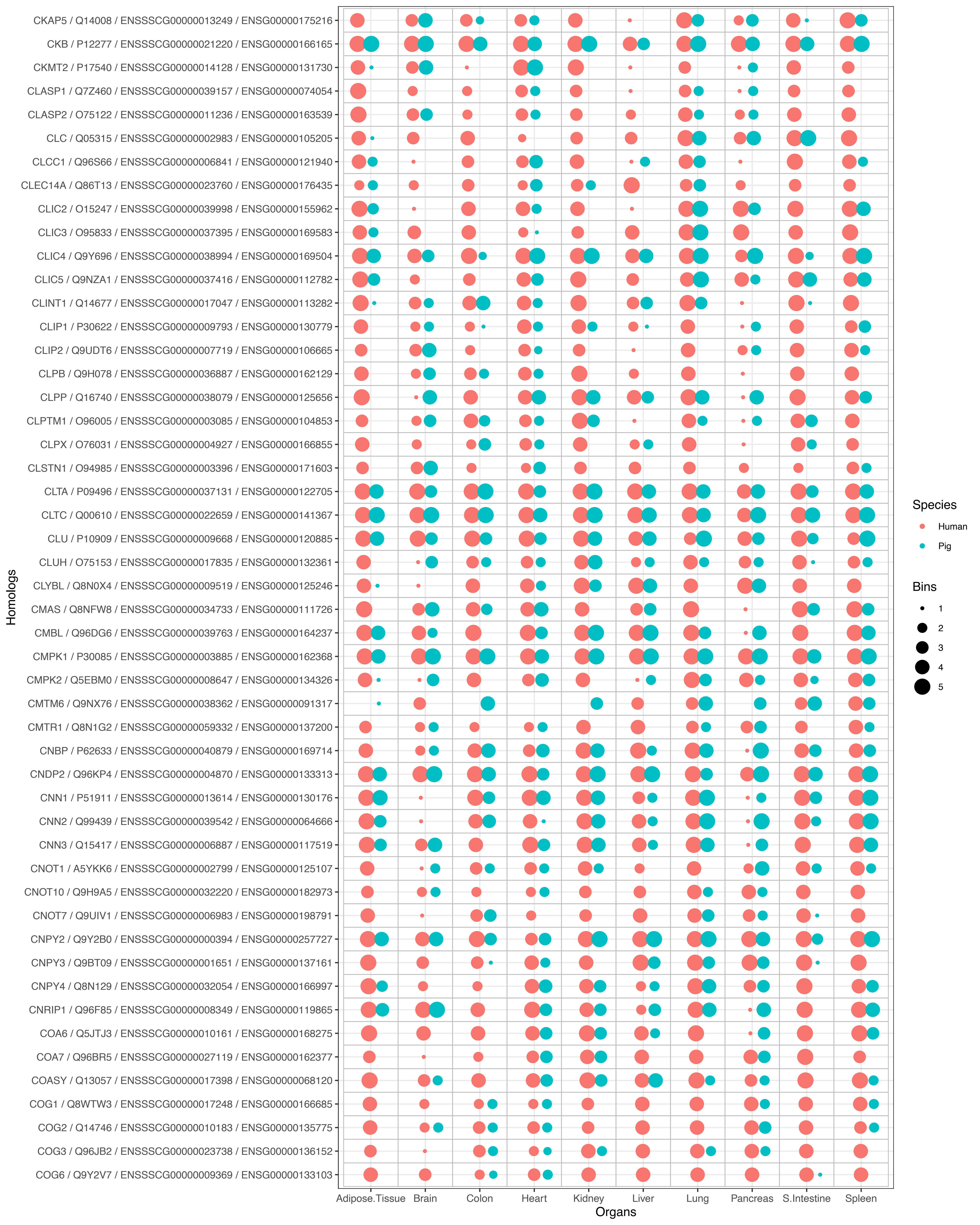
CKAP5 / Q14008 / ENSSSCG00000013249 / ENSG00000175216
CKB / P12277 / ENSSSCG00000021220 / ENSG00000166165
CKMT2 / P17540 / ENSSSCG00000014128 / ENSG00000131730
CLASP1 / Q7Z460 / ENSSSCG00000039157 / ENSG00000074054
CLASP2 / O75122 / ENSSSCG00000011236 / ENSG00000163539
CLC / Q05315 / ENSSSCG00000002983 / ENSG00000105205
CLCC1 / Q96S66 / ENSSSCG00000006841 / ENSG00000121940
CLEC14A / Q86T13 / ENSSSCG00000023760 / ENSG00000176435
CLIC2 / O15247 / ENSSSCG00000039998 / ENSG00000155962
CLIC3 / O95833 / ENSSSCG00000037395 / ENSG00000169583
CLIC4 / Q9Y696 / ENSSSCG00000038994 / ENSG00000169504
CLIC5 / Q9NZA1 / ENSSSCG00000037416 / ENSG00000112782
CLINT1 / Q14677 / ENSSSCG00000017047 / ENSG00000113282
CLIP1 / P30622 / ENSSSCG00000009793 / ENSG00000130779
CLIP2 / Q9UDT6 / ENSSSCG00000007719 / ENSG00000106665
CLPB / Q9H078 / ENSSSCG00000036887 / ENSG00000162129
CLPP / Q16740 / ENSSSCG00000038079 / ENSG00000125656
CLPTM1 / O96005 / ENSSSCG00000003085 / ENSG00000104853
CLPX / O76031 / ENSSSCG00000004927 / ENSG00000166855
CLSTN1 / O94985 / ENSSSCG00000003396 / ENSG00000171603
CLTA / P09496 / ENSSSCG00000037131 / ENSG00000122705
CLTC / Q00610 / ENSSSCG00000022659 / ENSG00000141367
CLU / P10909 / ENSSSCG00000009668 / ENSG00000120885
CLUH / O75153 / ENSSSCG00000017835 / ENSG00000132361
CLYBL / Q8N0X4 / ENSSSCG00000009519 / ENSG00000125246
CMAS / Q8NFW8 / ENSSSCG00000034733 / ENSG00000111726
CMBL / Q96DG6 / ENSSSCG00000039763 / ENSG00000164237
CMPK1 / P30085 / ENSSSCG00000003885 / ENSG00000162368
CMPK2 / Q5EBM0 / ENSSSCG00000008647 / ENSG00000134326
CMTM6 / Q9NX76 / ENSSSCG00000038362 / ENSG00000091317
CMTR1 / Q8N1G2 / ENSSSCG00000059332 / ENSG00000137200
CNBP / P62633 / ENSSSCG00000040879 / ENSG00000169714
CNDP2 / Q96KP4 / ENSSSCG00000004870 / ENSG00000133313
CNN1 / P51911 / ENSSSCG00000013614 / ENSG00000130176
CNN2 / Q99439 / ENSSSCG00000039542 / ENSG00000064666
CNN3 / Q15417 / ENSSSCG00000006887 / ENSG00000117519
CNOT1 / A5YKK6 / ENSSSCG00000002799 / ENSG00000125107
CNOT10 / Q9H9A5 / ENSSSCG00000032220 / ENSG00000182973
CNOT7 / Q9UIV1 / ENSSSCG00000006983 / ENSG00000198791
CNPY2 / Q9Y2B0 / ENSSSCG00000000394 / ENSG00000257727
CNPY3 / Q9BT09 / ENSSSCG00000001651 / ENSG00000137161
CNPY4 / Q8N129 / ENSSSCG00000032054 / ENSG00000166997
CNRIP1 / Q96F85 / ENSSSCG00000008349 / ENSG00000119865
COA6 / Q5JTJ3 / ENSSSCG00000010161 / ENSG00000168275
COA7 / Q96BR5 / ENSSSCG00000027119 / ENSG00000162377
COASY / Q13057 / ENSSSCG00000017398 / ENSG00000068120
COG1 / Q8WTW3 / ENSSSCG00000017248 / ENSG00000166685
COG2 / Q14746 / ENSSSCG00000010183 / ENSG00000135775
COG3 / Q96JB2 / ENSSSCG00000023738 / ENSG00000136152
COG6 / Q9Y2V7 / ENSSSCG00000009369 / ENSG00000133103
Homologs
Adipose.Tissue
Brain
Colon
Heart
Kidney
Liver
Lung
Pancreas
S.Intestine
Spleen
Organs
Species
Human
Pig
Bins
1
2
3
4
5

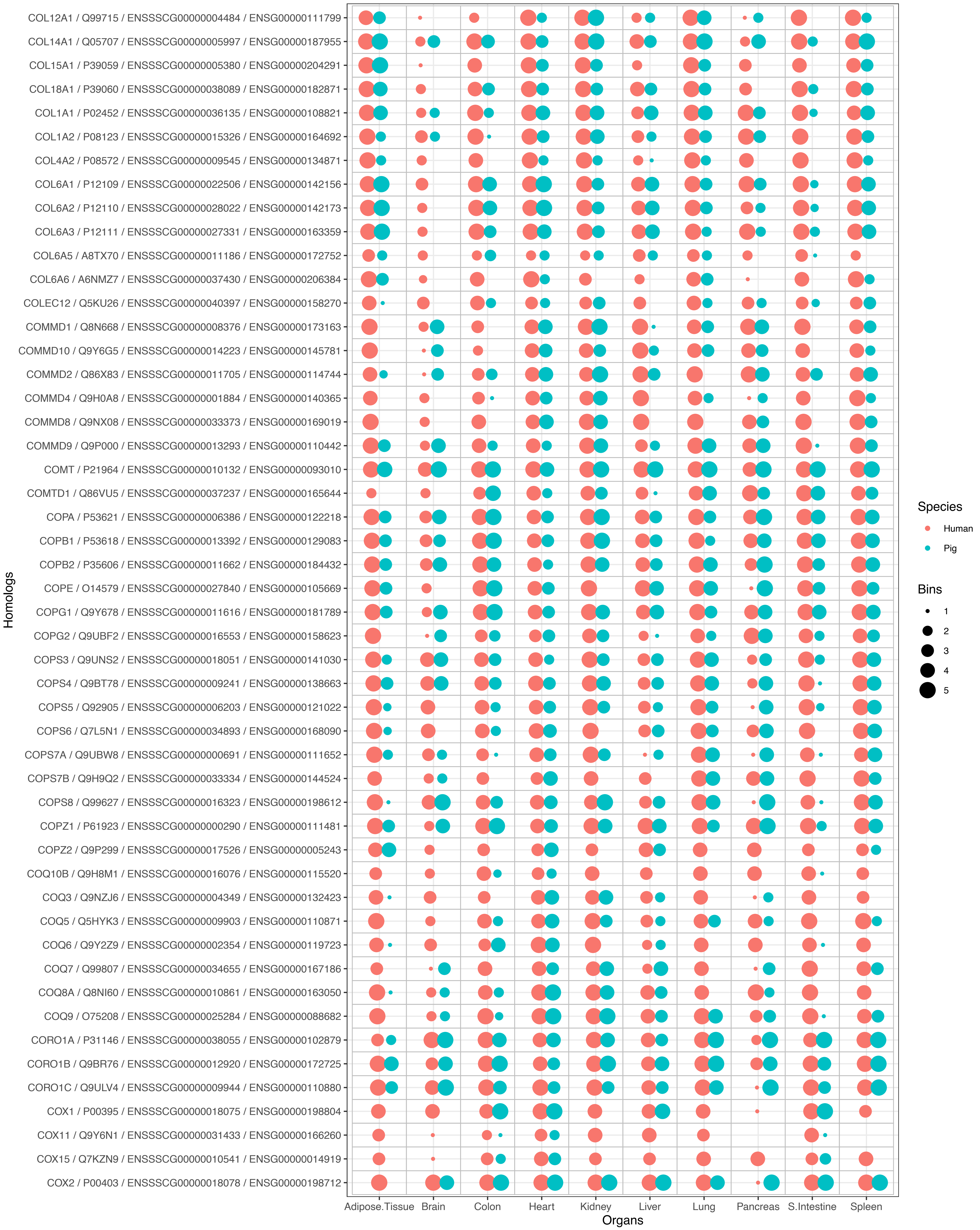
COL12A1 / Q99715 / ENSSSCG00000004484 / ENSG00000111799
COL14A1 / Q05707 / ENSSSCG00000005997 / ENSG00000187955
COL15A1 / P39059 / ENSSSCG00000005380 / ENSG00000204291
COL18A1 / P39060 / ENSSSCG00000038089 / ENSG00000182871
COL1A1 / P02452 / ENSSSCG00000036135 / ENSG00000108821
COL1A2 / P08123 / ENSSSCG00000015326 / ENSG00000164692
COL4A2 / P08572 / ENSSSCG00000009545 / ENSG00000134871
COL6A1 / P12109 / ENSSSCG00000022506 / ENSG00000142156
COL6A2 / P12110 / ENSSSCG00000028022 / ENSG00000142173
COL6A3 / P12111 / ENSSSCG00000027331 / ENSG00000163359
COL6A5 / A8TX70 / ENSSSCG00000011186 / ENSG00000172752
COL6A6 / A6NMZ7 / ENSSSCG00000037430 / ENSG00000206384
COLEC12 / Q5KU26 / ENSSSCG00000040397 / ENSG00000158270
COMMD1 / Q8N668 / ENSSSCG00000008376 / ENSG00000173163
COMMD10 / Q9Y6G5 / ENSSSCG00000014223 / ENSG00000145781
COMMD2 / Q86X83 / ENSSSCG00000011705 / ENSG00000114744
COMMD4 / Q9H0A8 / ENSSSCG00000001884 / ENSG00000140365
COMMD8 / Q9NX08 / ENSSSCG00000033373 / ENSG00000169019
COMMD9 / Q9P000 / ENSSSCG00000013293 / ENSG00000110442
COMT / P21964 / ENSSSCG00000010132 / ENSG00000093010
COMTD1 / Q86VU5 / ENSSSCG00000037237 / ENSG00000165644
COPA / P53621 / ENSSSCG00000006386 / ENSG00000122218
COPB1 / P53618 / ENSSSCG00000013392 / ENSG00000129083
COPB2 / P35606 / ENSSSCG00000011662 / ENSG00000184432
COPE / O14579 / ENSSSCG00000027840 / ENSG00000105669
COPG1 / Q9Y678 / ENSSSCG00000011616 / ENSG00000181789
COPG2 / Q9UBF2 / ENSSSCG00000016553 / ENSG00000158623
COPS3 / Q9UNS2 / ENSSSCG00000018051 / ENSG00000141030
COPS4 / Q9BT78 / ENSSSCG00000009241 / ENSG00000138663
COPS5 / Q92905 / ENSSSCG00000006203 / ENSG00000121022
COPS6 / Q7L5N1 / ENSSSCG00000034893 / ENSG00000168090
COPS7A / Q9UBW8 / ENSSSCG00000000691 / ENSG00000111652
COPS7B / Q9H9Q2 / ENSSSCG00000033334 / ENSG00000144524
COPS8 / Q99627 / ENSSSCG00000016323 / ENSG00000198612
COPZ1 / P61923 / ENSSSCG00000000290 / ENSG00000111481
COPZ2 / Q9P299 / ENSSSCG00000017526 / ENSG00000005243
COQ10B / Q9H8M1 / ENSSSCG00000016076 / ENSG00000115520
COQ3 / Q9NZJ6 / ENSSSCG00000004349 / ENSG00000132423
COQ5 / Q5HYK3 / ENSSSCG00000009903 / ENSG00000110871
COQ6 / Q9Y2Z9 / ENSSSCG00000002354 / ENSG00000119723
COQ7 / Q99807 / ENSSSCG00000034655 / ENSG00000167186
COQ8A / Q8NI60 / ENSSSCG00000010861 / ENSG00000163050
COQ9 / O75208 / ENSSSCG00000025284 / ENSG00000088682
CORO1A / P31146 / ENSSSCG00000038055 / ENSG00000102879
CORO1B / Q9BR76 / ENSSSCG00000012920 / ENSG00000172725
CORO1C / Q9ULV4 / ENSSSCG00000009944 / ENSG00000110880
COX1 / P00395 / ENSSSCG00000018075 / ENSG00000198804
COX11 / Q9Y6N1 / ENSSSCG00000031433 / ENSG00000166260
COX15 / Q7KZN9 / ENSSSCG00000010541 / ENSG00000014919
COX2 / P00403 / ENSSSCG00000018078 / ENSG00000198712
Homologs
Adipose.Tissue
Brain
Colon
Heart
Kidney
Liver
Lung
Pancreas
S.Intestine
Spleen
Organs
Species
Human
Pig
Bins
1
2
3
4
5

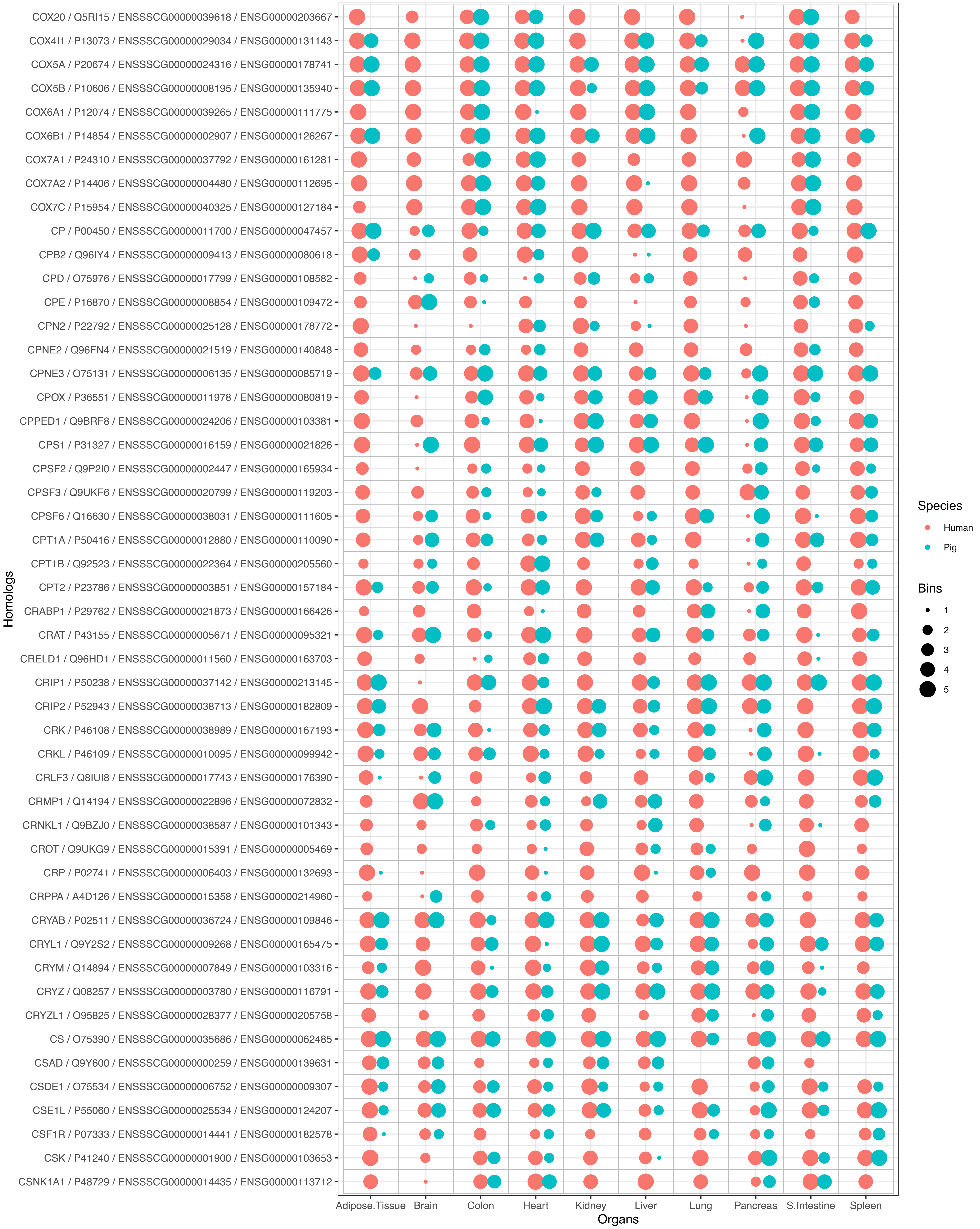
COX20 / Q5RI15 / ENSSSCG00000039618 / ENSG00000203667
COX4I1 / P13073 / ENSSSCG00000029034 / ENSG00000131143
COX5A / P20674 / ENSSSCG00000024316 / ENSG00000178741
COX5B / P10606 / ENSSSCG00000008195 / ENSG00000135940
COX6A1 / P12074 / ENSSSCG00000039265 / ENSG00000111775
COX6B1 / P14854 / ENSSSCG00000002907 / ENSG00000126267
COX7A1 / P24310 / ENSSSCG00000037792 / ENSG00000161281
COX7A2 / P14406 / ENSSSCG00000004480 / ENSG00000112695
COX7C / P15954 / ENSSSCG00000040325 / ENSG00000127184
CP / P00450 / ENSSSCG00000011700 / ENSG00000047457
CPB2 / Q96IY4 / ENSSSCG00000009413 / ENSG00000080618
CPD / O75976 / ENSSSCG00000017799 / ENSG00000108582
CPE / P16870 / ENSSSCG00000008854 / ENSG00000109472
CPN2 / P22792 / ENSSSCG00000025128 / ENSG00000178772
CPNE2 / Q96FN4 / ENSSSCG00000021519 / ENSG00000140848
CPNE3 / O75131 / ENSSSCG00000006135 / ENSG00000085719
CPOX / P36551 / ENSSSCG00000011978 / ENSG00000080819
CPPED1 / Q9BRF8 / ENSSSCG00000024206 / ENSG00000103381
CPS1 / P31327 / ENSSSCG00000016159 / ENSG00000021826
CPSF2 / Q9P2I0 / ENSSSCG00000002447 / ENSG00000165934
CPSF3 / Q9UKF6 / ENSSSCG00000020799 / ENSG00000119203
CPSF6 / Q16630 / ENSSSCG00000038031 / ENSG00000111605
CPT1A / P50416 / ENSSSCG00000012880 / ENSG00000110090
CPT1B / Q92523 / ENSSSCG00000022364 / ENSG00000205560
CPT2 / P23786 / ENSSSCG00000003851 / ENSG00000157184
CRABP1 / P29762 / ENSSSCG00000021873 / ENSG00000166426
CRAT / P43155 / ENSSSCG00000005671 / ENSG00000095321
CRELD1 / Q96HD1 / ENSSSCG00000011560 / ENSG00000163703
CRIP1 / P50238 / ENSSSCG00000037142 / ENSG00000213145
CRIP2 / P52943 / ENSSSCG00000038713 / ENSG00000182809
CRK / P46108 / ENSSSCG00000038989 / ENSG00000167193
CRKL / P46109 / ENSSSCG00000010095 / ENSG00000099942
CRLF3 / Q8IUI8 / ENSSSCG00000017743 / ENSG00000176390
CRMP1 / Q14194 / ENSSSCG00000022896 / ENSG00000072832
CRNKL1 / Q9BZJ0 / ENSSSCG00000038587 / ENSG00000101343
CROT / Q9UKG9 / ENSSSCG00000015391 / ENSG00000005469
CRP / P02741 / ENSSSCG00000006403 / ENSG00000132693
CRPPA / A4D126 / ENSSSCG00000015358 / ENSG00000214960
CRYAB / P02511 / ENSSSCG00000036724 / ENSG00000109846
CRYL1 / Q9Y2S2 / ENSSSCG00000009268 / ENSG00000165475
CRYM / Q14894 / ENSSSCG00000007849 / ENSG00000103316
CRYZ / Q08257 / ENSSSCG00000003780 / ENSG00000116791
CRYZL1 / O95825 / ENSSSCG00000028377 / ENSG00000205758
CS / O75390 / ENSSSCG00000035686 / ENSG00000062485
CSAD / Q9Y600 / ENSSSCG00000000259 / ENSG00000139631
CSDE1 / O75534 / ENSSSCG00000006752 / ENSG00000009307
CSE1L / P55060 / ENSSSCG00000025534 / ENSG00000124207
CSF1R / P07333 / ENSSSCG00000014441 / ENSG00000182578
CSK / P41240 / ENSSSCG00000001900 / ENSG00000103653
CSNK1A1 / P48729 / ENSSSCG00000014435 / ENSG00000113712
Homologs
Adipose.Tissue
Brain
Colon
Heart
Kidney
Liver
Lung
Pancreas
S.Intestine
Spleen
Organs
Species
Human
Pig
Bins
1
2
3
4
5

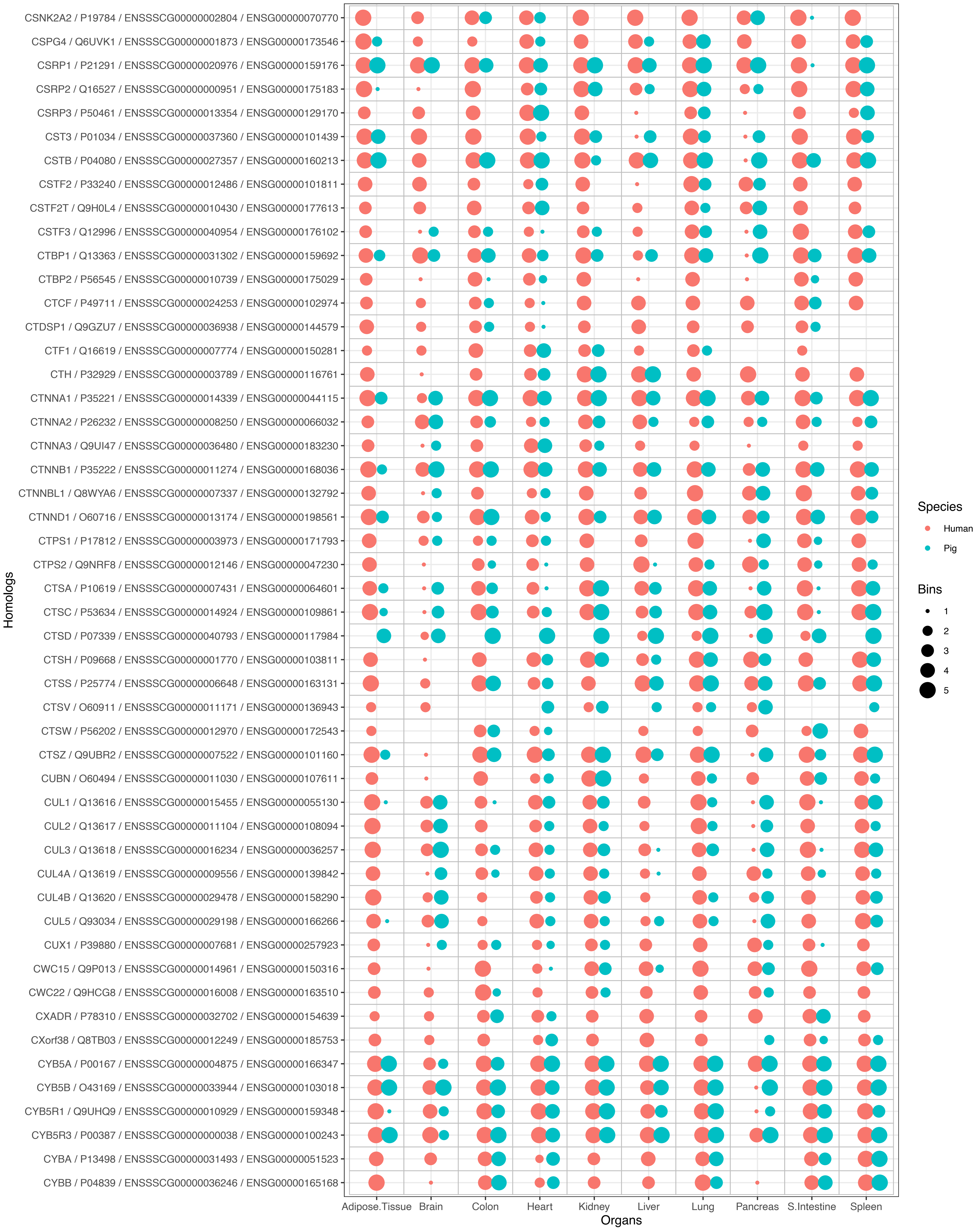

CSNK2A2 / P19784 / ENSSSCG00000002804 / ENSG00000070770
CSPG4 / Q6UVK1 / ENSSSCG00000001873 / ENSG00000173546
CSRP1 / P21291 / ENSSSCG00000020976 / ENSG00000159176
CSRP2 / Q16527 / ENSSSCG00000000951 / ENSG00000175183
CSRP3 / P50461 / ENSSSCG00000013354 / ENSG00000129170
CST3 / P01034 / ENSSSCG00000037360 / ENSG00000101439
CSTB / P04080 / ENSSSCG00000027357 / ENSG00000160213
CSTF2 / P33240 / ENSSSCG00000012486 / ENSG00000101811
CSTF2T / Q9H0L4 / ENSSSCG00000010430 / ENSG00000177613
CSTF3 / Q12996 / ENSSSCG00000040954 / ENSG00000176102
CTBP1 / Q13363 / ENSSSCG00000031302 / ENSG00000159692
CTBP2 / P56545 / ENSSSCG00000010739 / ENSG00000175029
CTCF / P49711 / ENSSSCG00000024253 / ENSG00000102974
CTDSP1 / Q9GZU7 / ENSSSCG00000036938 / ENSG00000144579
CTF1 / Q16619 / ENSSSCG00000007774 / ENSG00000150281
CTH / P32929 / ENSSSCG00000003789 / ENSG00000116761
CTNNA1 / P35221 / ENSSSCG00000014339 / ENSG00000044115
CTNNA2 / P26232 / ENSSSCG00000008250 / ENSG00000066032
CTNNA3 / Q9UI47 / ENSSSCG00000036480 / ENSG00000183230
CTNNB1 / P35222 / ENSSSCG00000011274 / ENSG00000168036
CTNNBL1 / Q8WYA6 / ENSSSCG00000007337 / ENSG00000132792
CTNND1 / O60716 / ENSSSCG00000013174 / ENSG00000198561
CTPS1 / P17812 / ENSSSCG00000003973 / ENSG00000171793
CTPS2 / Q9NRF8 / ENSSSCG00000012146 / ENSG00000047230
CTSA / P10619 / ENSSSCG00000007431 / ENSG00000064601
CTSC / P53634 / ENSSSCG00000014924 / ENSG00000109861
CTSD / P07339 / ENSSSCG00000040793 / ENSG00000117984
CTSH / P09668 / ENSSSCG00000001770 / ENSG00000103811
CTSS / P25774 / ENSSSCG00000006648 / ENSG00000163131
CTSV / O60911 / ENSSSCG00000011171 / ENSG00000136943
CTSW / P56202 / ENSSSCG00000012970 / ENSG00000172543
CTSZ / Q9UBR2 / ENSSSCG00000007522 / ENSG00000101160
CUBN / O60494 / ENSSSCG00000011030 / ENSG00000107611
CUL1 / Q13616 / ENSSSCG00000015455 / ENSG00000055130
CUL2 / Q13617 / ENSSSCG00000011104 / ENSG00000108094
CUL3 / Q13618 / ENSSSCG00000016234 / ENSG00000036257
CUL4A / Q13619 / ENSSSCG00000009556 / ENSG00000139842
CUL4B / Q13620 / ENSSSCG00000029478 / ENSG00000158290
CUL5 / Q93034 / ENSSSCG00000029198 / ENSG00000166266
CUX1 / P39880 / ENSSSCG00000007681 / ENSG00000257923
CWC15 / Q9P013 / ENSSSCG00000014961 / ENSG00000150316
CWC22 / Q9HCG8 / ENSSSCG00000016008 / ENSG00000163510
CXADR / P78310 / ENSSSCG00000032702 / ENSG00000154639
CXorf38 / Q8TB03 / ENSSSCG00000012249 / ENSG00000185753
CYB5A / P00167 / ENSSSCG00000004875 / ENSG00000166347
CYB5B / O43169 / ENSSSCG00000033944 / ENSG00000103018
CYB5R1 / Q9UHQ9 / ENSSSCG00000010929 / ENSG00000159348
CYB5R3 / P00387 / ENSSSCG00000000038 / ENSG00000100243
CYBA / P13498 / ENSSSCG00000031493 / ENSG00000051523
CYBB / P04839 / ENSSSCG00000036246 / ENSG00000165168
Homologs
Adipose.Tissue
Brain
Colon
Heart
Kidney
Liver
Lung
Pancreas
S.Intestine
Spleen
Organs
Species
Human
Pig
Bins
1
2
3
4
5

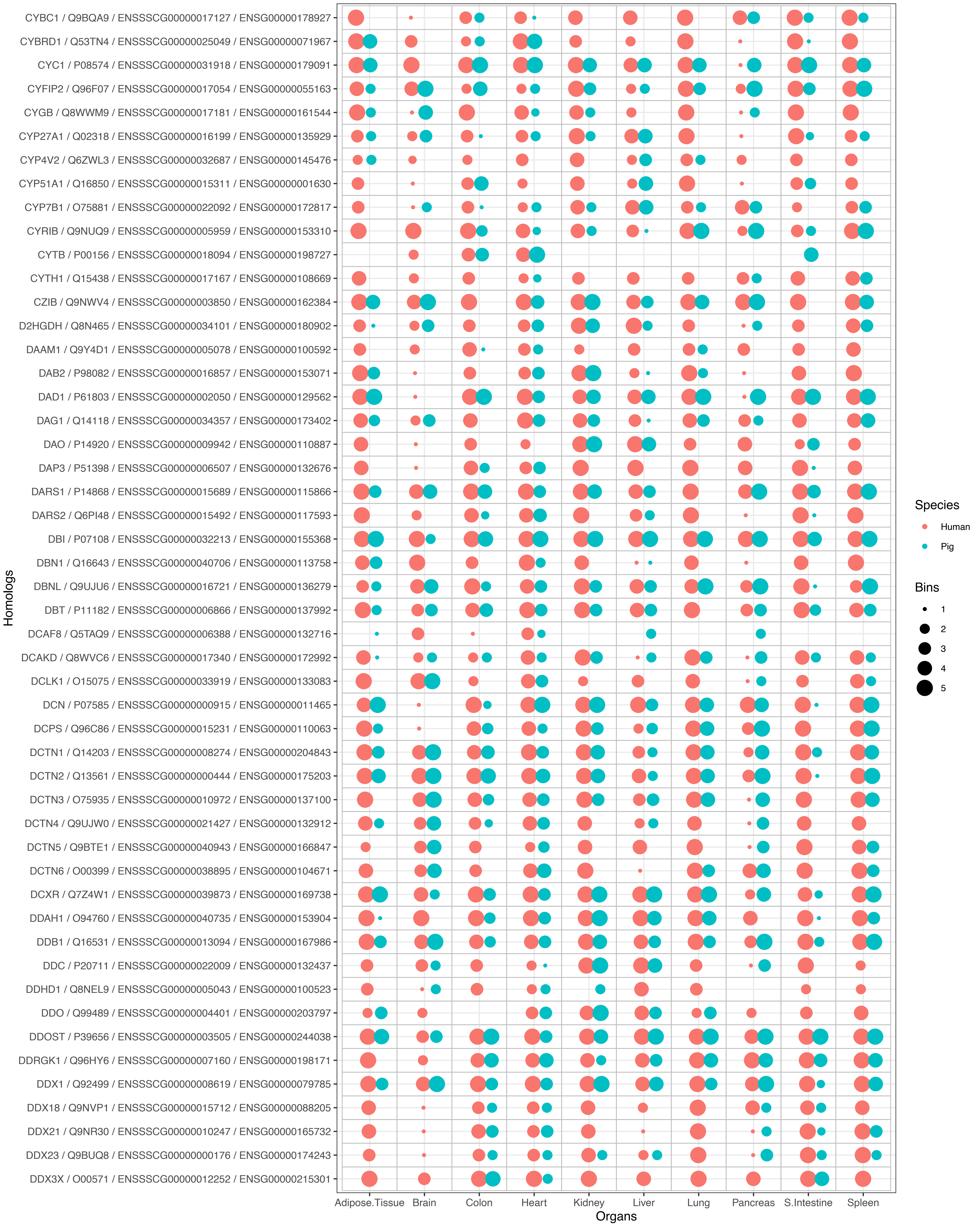
CYBC1 / Q9BQA9 / ENSSSCG00000017127 / ENSG00000178927
CYBRD1 / Q53TN4 / ENSSSCG00000025049 / ENSG00000071967
CYC1 / P08574 / ENSSSCG00000031918 / ENSG00000179091
CYFIP2 / Q96F07 / ENSSSCG00000017054 / ENSG00000055163
CYGB / Q8WWM9 / ENSSSCG00000017181 / ENSG00000161544
CYP27A1 / Q02318 / ENSSSCG00000016199 / ENSG00000135929
CYP4V2 / Q6ZWL3 / ENSSSCG00000032687 / ENSG00000145476
CYP51A1 / Q16850 / ENSSSCG00000015311 / ENSG00000001630
CYP7B1 / O75881 / ENSSSCG00000022092 / ENSG00000172817
CYRIB / Q9NUQ9 / ENSSSCG00000005959 / ENSG00000153310
CYTB / P00156 / ENSSSCG00000018094 / ENSG00000198727
CYTH1 / Q15438 / ENSSSCG00000017167 / ENSG00000108669
CZIB / Q9NWV4 / ENSSSCG00000003850 / ENSG00000162384
D2HGDH / Q8N465 / ENSSSCG00000034101 / ENSG00000180902
DAAM1 / Q9Y4D1 / ENSSSCG00000005078 / ENSG00000100592
DAB2 / P98082 / ENSSSCG00000016857 / ENSG00000153071
DAD1 / P61803 / ENSSSCG00000002050 / ENSG00000129562
DAG1 / Q14118 / ENSSSCG00000034357 / ENSG00000173402
DAO / P14920 / ENSSSCG00000009942 / ENSG00000110887
DAP3 / P51398 / ENSSSCG00000006507 / ENSG00000132676
DARS1 / P14868 / ENSSSCG00000015689 / ENSG00000115866
DARS2 / Q6PI48 / ENSSSCG00000015492 / ENSG00000117593
DBI / P07108 / ENSSSCG00000032213 / ENSG00000155368
DBN1 / Q16643 / ENSSSCG00000040706 / ENSG00000113758
DBNL / Q9UJU6 / ENSSSCG00000016721 / ENSG00000136279
DBT / P11182 / ENSSSCG00000006866 / ENSG00000137992
DCAF8 / Q5TAQ9 / ENSSSCG00000006388 / ENSG00000132716
DCAKD / Q8WVC6 / ENSSSCG00000017340 / ENSG00000172992
DCLK1 / O15075 / ENSSSCG00000033919 / ENSG00000133083
DCN / P07585 / ENSSSCG00000000915 / ENSG00000011465
DCPS / Q96C86 / ENSSSCG00000015231 / ENSG00000110063
DCTN1 / Q14203 / ENSSSCG00000008274 / ENSG00000204843
DCTN2 / Q13561 / ENSSSCG00000000444 / ENSG00000175203
DCTN3 / O75935 / ENSSSCG00000010972 / ENSG00000137100
DCTN4 / Q9UJW0 / ENSSSCG00000021427 / ENSG00000132912
DCTN5 / Q9BTE1 / ENSSSCG00000040943 / ENSG00000166847
DCTN6 / O00399 / ENSSSCG00000038895 / ENSG00000104671
DCXR / Q7Z4W1 / ENSSSCG00000039873 / ENSG00000169738
DDAH1 / O94760 / ENSSSCG00000040735 / ENSG00000153904
DDB1 / Q16531 / ENSSSCG00000013094 / ENSG00000167986
DDC / P20711 / ENSSSCG00000022009 / ENSG00000132437
DDHD1 / Q8NEL9 / ENSSSCG00000005043 / ENSG00000100523
DDO / Q99489 / ENSSSCG00000004401 / ENSG00000203797
DDOST / P39656 / ENSSSCG00000003505 / ENSG00000244038
DDRGK1 / Q96HY6 / ENSSSCG00000007160 / ENSG00000198171
DDX1 / Q92499 / ENSSSCG00000008619 / ENSG00000079785
DDX18 / Q9NVP1 / ENSSSCG00000015712 / ENSG00000088205
DDX21 / Q9NR30 / ENSSSCG00000010247 / ENSG00000165732
DDX23 / Q9BUQ8 / ENSSSCG00000000176 / ENSG00000174243
DDX3X / O00571 / ENSSSCG00000012252 / ENSG00000215301
Homologs
Adipose.Tissue
Brain
Colon
Heart
Kidney
Liver
Lung
Pancreas
S.Intestine
Spleen
Organs
Species
Human
Pig
Bins
1
2
3
4
5

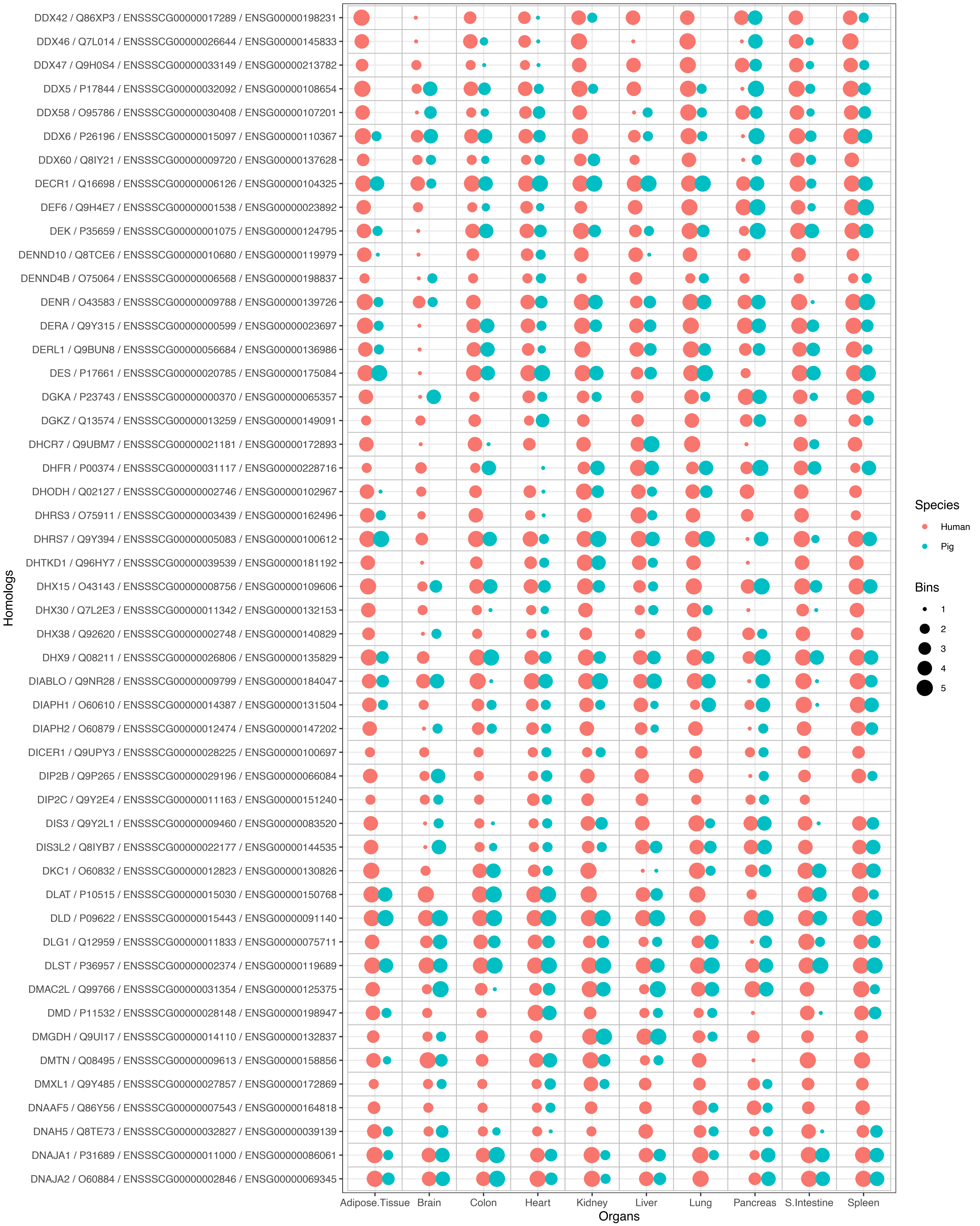
DDX42 / Q86XP3 / ENSSSCG00000017289 / ENSG00000198231
DDX46 / Q7L014 / ENSSSCG00000026644 / ENSG00000145833
DDX47 / Q9H0S4 / ENSSSCG00000033149 / ENSG00000213782
DDX5 / P17844 / ENSSSCG00000032092 / ENSG00000108654
DDX58 / O95786 / ENSSSCG00000030408 / ENSG00000107201
DDX6 / P26196 / ENSSSCG00000015097 / ENSG00000110367
DDX60 / Q8IY21 / ENSSSCG00000009720 / ENSG00000137628
DECR1 / Q16698 / ENSSSCG00000006126 / ENSG00000104325
DEF6 / Q9H4E7 / ENSSSCG00000001538 / ENSG00000023892
DEK / P35659 / ENSSSCG00000001075 / ENSG00000124795
DENND10 / Q8TCE6 / ENSSSCG00000010680 / ENSG00000119979
DENND4B / O75064 / ENSSSCG00000006568 / ENSG00000198837
DENR / O43583 / ENSSSCG00000009788 / ENSG00000139726
DERA / Q9Y315 / ENSSSCG00000000599 / ENSG00000023697
DERL1 / Q9BUN8 / ENSSSCG00000056684 / ENSG00000136986
DES / P17661 / ENSSSCG00000020785 / ENSG00000175084
DGKA / P23743 / ENSSSCG00000000370 / ENSG00000065357
DGKZ / Q13574 / ENSSSCG00000013259 / ENSG00000149091
DHCR7 / Q9UBM7 / ENSSSCG00000021181 / ENSG00000172893
DHFR / P00374 / ENSSSCG00000031117 / ENSG00000228716
DHODH / Q02127 / ENSSSCG00000002746 / ENSG00000102967
DHRS3 / O75911 / ENSSSCG00000003439 / ENSG00000162496
DHRS7 / Q9Y394 / ENSSSCG00000005083 / ENSG00000100612
DHTKD1 / Q96HY7 / ENSSSCG00000039539 / ENSG00000181192
DHX15 / O43143 / ENSSSCG00000008756 / ENSG00000109606
DHX30 / Q7L2E3 / ENSSSCG00000011342 / ENSG00000132153
DHX38 / Q92620 / ENSSSCG00000002748 / ENSG00000140829
DHX9 / Q08211 / ENSSSCG00000026806 / ENSG00000135829
DIABLO / Q9NR28 / ENSSSCG00000009799 / ENSG00000184047
DIAPH1 / O60610 / ENSSSCG00000014387 / ENSG00000131504
DIAPH2 / O60879 / ENSSSCG00000012474 / ENSG00000147202
DICER1 / Q9UPY3 / ENSSSCG00000028225 / ENSG00000100697
DIP2B / Q9P265 / ENSSSCG00000029196 / ENSG00000066084
DIP2C / Q9Y2E4 / ENSSSCG00000011163 / ENSG00000151240
DIS3 / Q9Y2L1 / ENSSSCG00000009460 / ENSG00000083520
DIS3L2 / Q8IYB7 / ENSSSCG00000022177 / ENSG00000144535
DKC1 / O60832 / ENSSSCG00000012823 / ENSG00000130826
DLAT / P10515 / ENSSSCG00000015030 / ENSG00000150768
DLD / P09622 / ENSSSCG00000015443 / ENSG00000091140
DLG1 / Q12959 / ENSSSCG00000011833 / ENSG00000075711
DLST / P36957 / ENSSSCG00000002374 / ENSG00000119689
DMAC2L / Q99766 / ENSSSCG00000031354 / ENSG00000125375
DMD / P11532 / ENSSSCG00000028148 / ENSG00000198947
DMGDH / Q9UI17 / ENSSSCG00000014110 / ENSG00000132837
DMTN / Q08495 / ENSSSCG00000009613 / ENSG00000158856
DMXL1 / Q9Y485 / ENSSSCG00000027857 / ENSG00000172869
DNAAF5 / Q86Y56 / ENSSSCG00000007543 / ENSG00000164818
DNAH5 / Q8TE73 / ENSSSCG00000032827 / ENSG00000039139
DNAJA1 / P31689 / ENSSSCG00000011000 / ENSG00000086061
DNAJA2 / O60884 / ENSSSCG00000002846 / ENSG00000069345
Homologs
Adipose.Tissue
Brain
Colon
Heart
Kidney
Liver
Lung
Pancreas
S.Intestine
Spleen
Organs
Species
Human
Pig
Bins
1
2
3
4
5

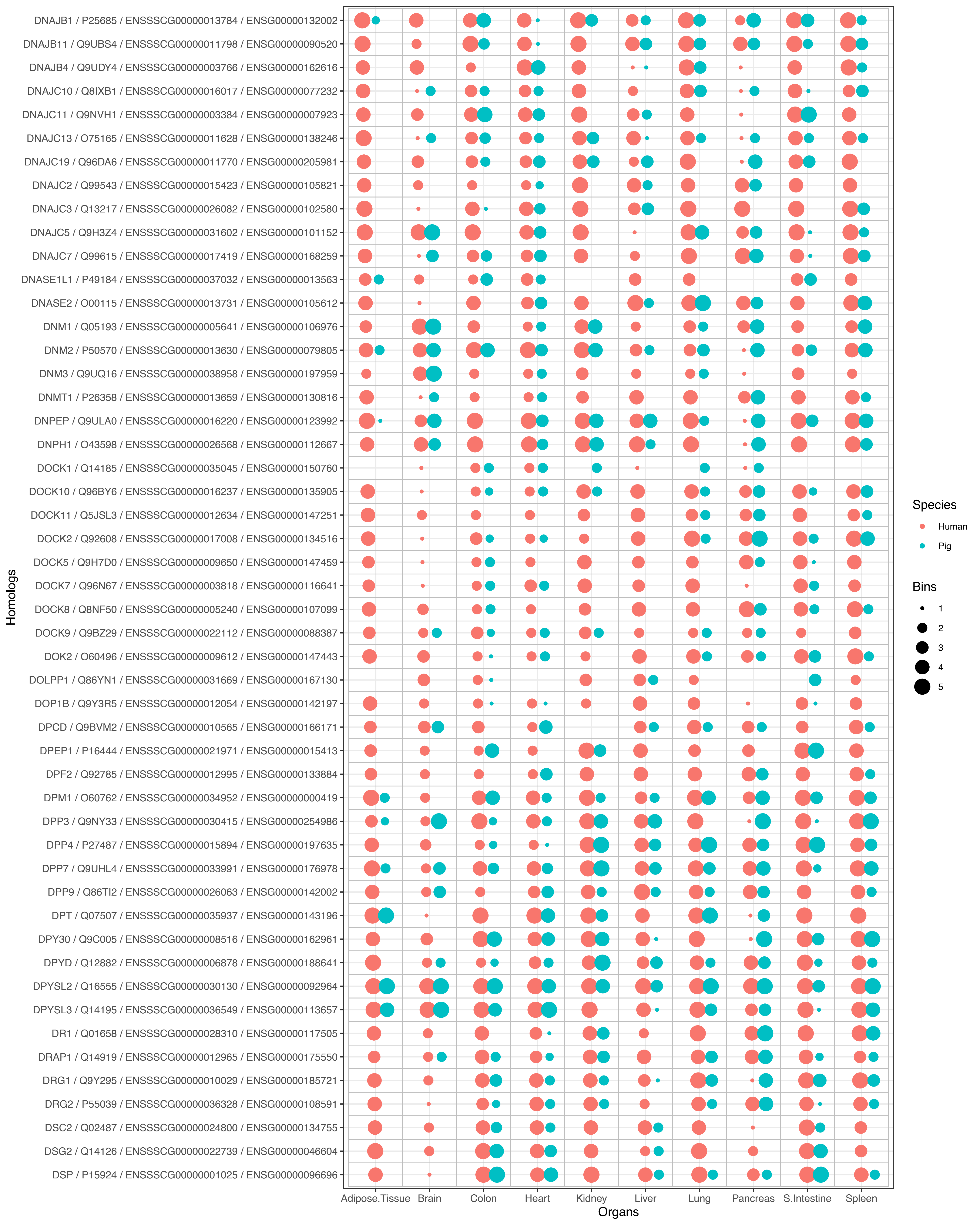
DNAJB1 / P25685 / ENSSSCG00000013784 / ENSG00000132002
DNAJB11 / Q9UBS4 / ENSSSCG00000011798 / ENSG00000090520
DNAJB4 / Q9UDY4 / ENSSSCG00000003766 / ENSG00000162616
DNAJC10 / Q8IXB1 / ENSSSCG00000016017 / ENSG00000077232
DNAJC11 / Q9NVH1 / ENSSSCG00000003384 / ENSG00000007923
DNAJC13 / O75165 / ENSSSCG00000011628 / ENSG00000138246
DNAJC19 / Q96DA6 / ENSSSCG00000011770 / ENSG00000205981
DNAJC2 / Q99543 / ENSSSCG00000015423 / ENSG00000105821
DNAJC3 / Q13217 / ENSSSCG00000026082 / ENSG00000102580
DNAJC5 / Q9H3Z4 / ENSSSCG00000031602 / ENSG00000101152
DNAJC7 / Q99615 / ENSSSCG00000017419 / ENSG00000168259
DNASE1L1 / P49184 / ENSSSCG00000037032 / ENSG00000013563
DNASE2 / O00115 / ENSSSCG00000013731 / ENSG00000105612
DNM1 / Q05193 / ENSSSCG00000005641 / ENSG00000106976
DNM2 / P50570 / ENSSSCG00000013630 / ENSG00000079805
DNM3 / Q9UQ16 / ENSSSCG00000038958 / ENSG00000197959
DNMT1 / P26358 / ENSSSCG00000013659 / ENSG00000130816
DNPEP / Q9ULA0 / ENSSSCG00000016220 / ENSG00000123992
DNPH1 / O43598 / ENSSSCG00000026568 / ENSG00000112667
DOCK1 / Q14185 / ENSSSCG00000035045 / ENSG00000150760
DOCK10 / Q96BY6 / ENSSSCG00000016237 / ENSG00000135905
DOCK11 / Q5JSL3 / ENSSSCG00000012634 / ENSG00000147251
DOCK2 / Q92608 / ENSSSCG00000017008 / ENSG00000134516
DOCK5 / Q9H7D0 / ENSSSCG00000009650 / ENSG00000147459
DOCK7 / Q96N67 / ENSSSCG00000003818 / ENSG00000116641
DOCK8 / Q8NF50 / ENSSSCG00000005240 / ENSG00000107099
DOCK9 / Q9BZ29 / ENSSSCG00000022112 / ENSG00000088387
DOK2 / O60496 / ENSSSCG00000009612 / ENSG00000147443
DOLPP1 / Q86YN1 / ENSSSCG00000031669 / ENSG00000167130
DOP1B / Q9Y3R5 / ENSSSCG00000012054 / ENSG00000142197
DPCD / Q9BVM2 / ENSSSCG00000010565 / ENSG00000166171
DPEP1 / P16444 / ENSSSCG00000021971 / ENSG00000015413
DPF2 / Q92785 / ENSSSCG00000012995 / ENSG00000133884
DPM1 / O60762 / ENSSSCG00000034952 / ENSG00000000419
DPP3 / Q9NY33 / ENSSSCG00000030415 / ENSG00000254986
DPP4 / P27487 / ENSSSCG00000015894 / ENSG00000197635
DPP7 / Q9UHL4 / ENSSSCG00000033991 / ENSG00000176978
DPP9 / Q86TI2 / ENSSSCG00000026063 / ENSG00000142002
DPT / Q07507 / ENSSSCG00000035937 / ENSG00000143196
DPY30 / Q9C005 / ENSSSCG00000008516 / ENSG00000162961
DPYD / Q12882 / ENSSSCG00000006878 / ENSG00000188641
DPYSL2 / Q16555 / ENSSSCG00000030130 / ENSG00000092964
DPYSL3 / Q14195 / ENSSSCG00000036549 / ENSG00000113657
DR1 / Q01658 / ENSSSCG00000028310 / ENSG00000117505
DRAP1 / Q14919 / ENSSSCG00000012965 / ENSG00000175550
DRG1 / Q9Y295 / ENSSSCG00000010029 / ENSG00000185721
DRG2 / P55039 / ENSSSCG00000036328 / ENSG00000108591
DSC2 / Q02487 / ENSSSCG00000024800 / ENSG00000134755
DSG2 / Q14126 / ENSSSCG00000022739 / ENSG00000046604
DSP / P15924 / ENSSSCG00000001025 / ENSG00000096696
Homologs
Adipose.Tissue
Brain
Colon
Heart
Kidney
Liver
Lung
Pancreas
S.Intestine
Spleen
Organs
Species
Human
Pig
Bins
1
2
3
4
5

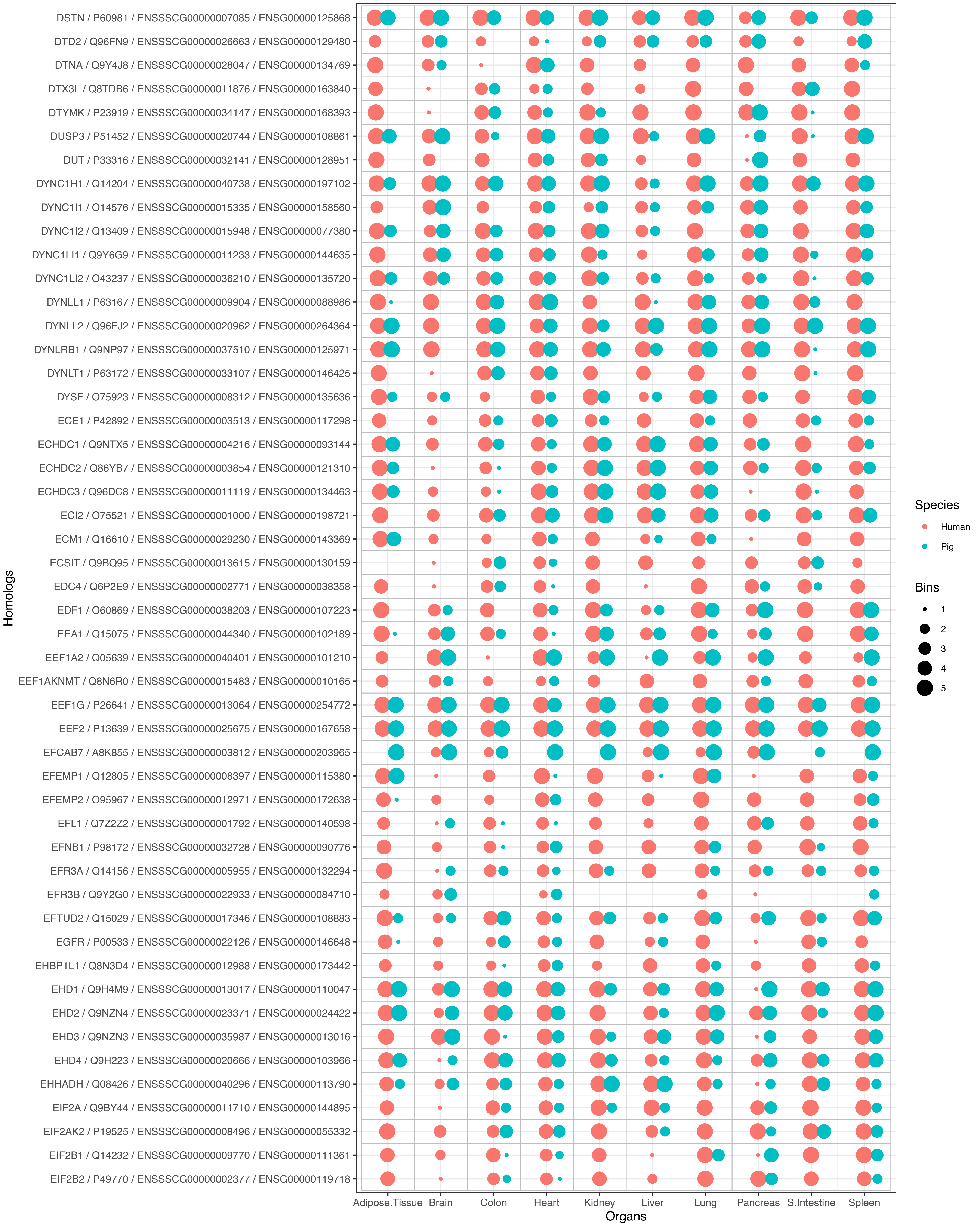

DSTN / P60981 / ENSSSCG00000007085 / ENSG00000125868
DTD2 / Q96FN9 / ENSSSCG00000026663 / ENSG00000129480
DTNA / Q9Y4J8 / ENSSSCG00000028047 / ENSG00000134769
DTX3L / Q8TDB6 / ENSSSCG00000011876 / ENSG00000163840
DTYMK / P23919 / ENSSSCG00000034147 / ENSG00000168393
DUSP3 / P51452 / ENSSSCG00000020744 / ENSG00000108861
DUT / P33316 / ENSSSCG00000032141 / ENSG00000128951
DYNC1H1 / Q14204 / ENSSSCG00000040738 / ENSG00000197102
DYNC1I1 / O14576 / ENSSSCG00000015335 / ENSG00000158560
DYNC1I2 / Q13409 / ENSSSCG00000015948 / ENSG00000077380
DYNC1LI1 / Q9Y6G9 / ENSSSCG00000011233 / ENSG00000144635
DYNC1LI2 / O43237 / ENSSSCG00000036210 / ENSG00000135720
DYNLL1 / P63167 / ENSSSCG00000009904 / ENSG00000088986
DYNLL2 / Q96FJ2 / ENSSSCG00000020962 / ENSG00000264364
DYNLRB1 / Q9NP97 / ENSSSCG00000037510 / ENSG00000125971
DYNLT1 / P63172 / ENSSSCG00000033107 / ENSG00000146425
DYSF / O75923 / ENSSSCG00000008312 / ENSG00000135636
ECE1 / P42892 / ENSSSCG00000003513 / ENSG00000117298
ECHDC1 / Q9NTX5 / ENSSSCG00000004216 / ENSG00000093144
ECHDC2 / Q86YB7 / ENSSSCG00000003854 / ENSG00000121310
ECHDC3 / Q96DC8 / ENSSSCG00000011119 / ENSG00000134463
ECI2 / O75521 / ENSSSCG00000001000 / ENSG00000198721
ECM1 / Q16610 / ENSSSCG00000029230 / ENSG00000143369
ECSIT / Q9BQ95 / ENSSSCG00000013615 / ENSG00000130159
EDC4 / Q6P2E9 / ENSSSCG00000002771 / ENSG00000038358
EDF1 / O60869 / ENSSSCG00000038203 / ENSG00000107223
EEA1 / Q15075 / ENSSSCG00000044340 / ENSG00000102189
EEF1A2 / Q05639 / ENSSSCG00000040401 / ENSG00000101210
EEF1AKNMT / Q8N6R0 / ENSSSCG00000015483 / ENSG00000010165
EEF1G / P26641 / ENSSSCG00000013064 / ENSG00000254772
EEF2 / P13639 / ENSSSCG00000025675 / ENSG00000167658
EFCAB7 / A8K855 / ENSSSCG00000003812 / ENSG00000203965
EFEMP1 / Q12805 / ENSSSCG00000008397 / ENSG00000115380
EFEMP2 / O95967 / ENSSSCG00000012971 / ENSG00000172638
EFL1 / Q7Z2Z2 / ENSSSCG00000001792 / ENSG00000140598
EFNB1 / P98172 / ENSSSCG00000032728 / ENSG00000090776
EFR3A / Q14156 / ENSSSCG00000005955 / ENSG00000132294
EFR3B / Q9Y2G0 / ENSSSCG00000022933 / ENSG00000084710
EFTUD2 / Q15029 / ENSSSCG00000017346 / ENSG00000108883
EGFR / P00533 / ENSSSCG00000022126 / ENSG00000146648
EHBP1L1 / Q8N3D4 / ENSSSCG00000012988 / ENSG00000173442
EHD1 / Q9H4M9 / ENSSSCG00000013017 / ENSG00000110047
EHD2 / Q9NZN4 / ENSSSCG00000023371 / ENSG00000024422
EHD3 / Q9NZN3 / ENSSSCG00000035987 / ENSG00000013016
EHD4 / Q9H223 / ENSSSCG00000020666 / ENSG00000103966
EHHADH / Q08426 / ENSSSCG00000040296 / ENSG00000113790
EIF2A / Q9BY44 / ENSSSCG00000011710 / ENSG00000144895
EIF2AK2 / P19525 / ENSSSCG00000008496 / ENSG00000055332
EIF2B1 / Q14232 / ENSSSCG00000009770 / ENSG00000111361
EIF2B2 / P49770 / ENSSSCG00000002377 / ENSG00000119718
Homologs
Adipose.Tissue
Brain
Colon
Heart
Kidney
Liver
Lung
Pancreas
S.Intestine
Spleen
Organs
Species
Human
Pig
Bins
1
2
3
4
5

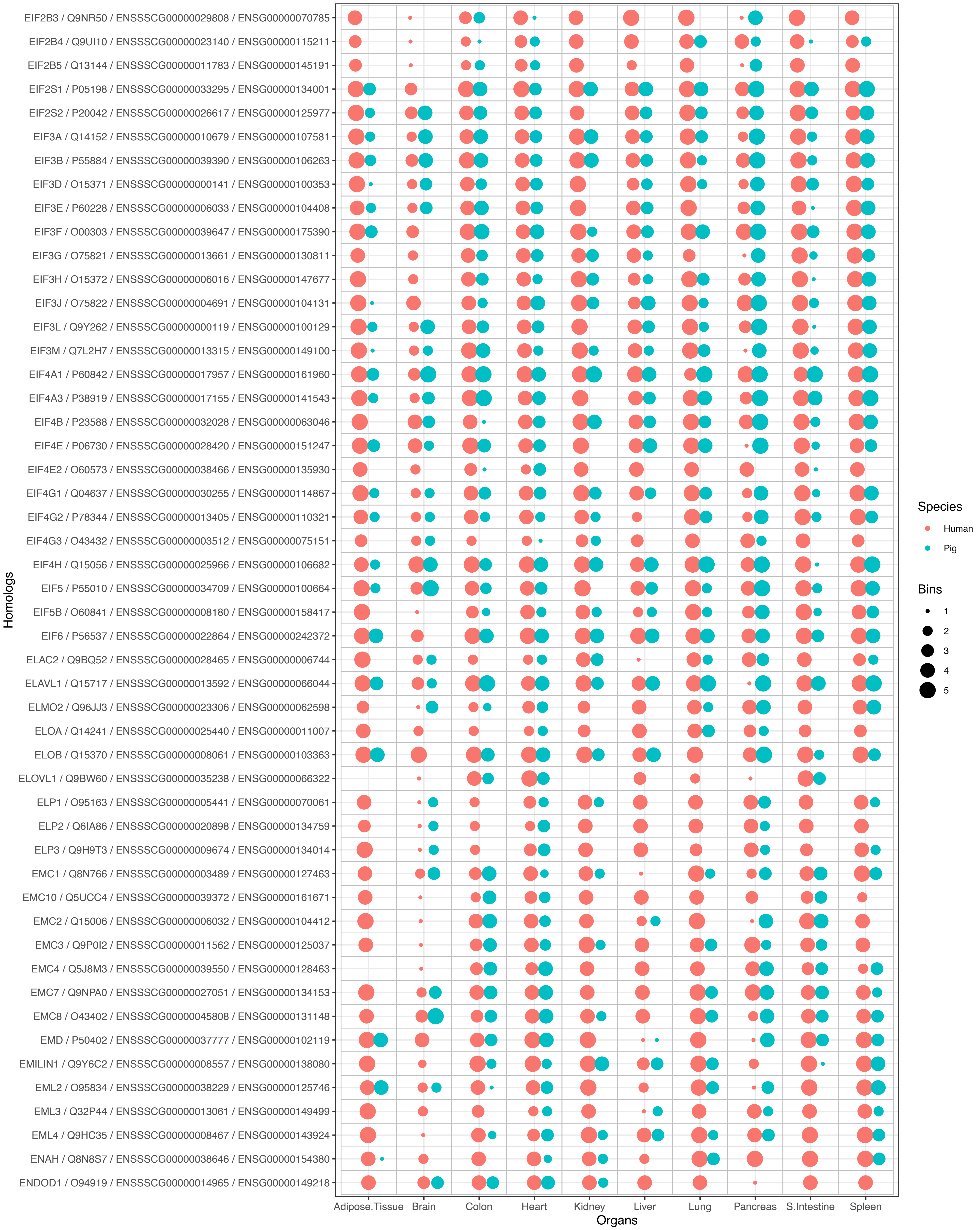

EIF2B3 / Q9NR50 / ENSSSCG00000029808 / ENSG00000070785
EIF2B4 / Q9UI10 / ENSSSCG00000023140 / ENSG00000115211
EIF2B5 / Q13144 / ENSSSCG00000011783 / ENSG00000145191
EIF2S1 / P05198 / ENSSSCG00000033295 / ENSG00000134001
EIF2S2 / P20042 / ENSSSCG00000026617 / ENSG00000125977
EIF3A / Q14152 / ENSSSCG00000010679 / ENSG00000107581
EIF3B / P55884 / ENSSSCG00000039390 / ENSG00000106263
EIF3D / O15371 / ENSSSCG00000000141 / ENSG00000100353
EIF3E / P60228 / ENSSSCG00000006033 / ENSG00000104408
EIF3F / O00303 / ENSSSCG00000039647 / ENSG00000175390
EIF3G / O75821 / ENSSSCG00000013661 / ENSG00000130811
EIF3H / O15372 / ENSSSCG00000006016 / ENSG00000147677
EIF3J / O75822 / ENSSSCG00000004691 / ENSG00000104131
EIF3L / Q9Y262 / ENSSSCG00000000119 / ENSG00000100129
EIF3M / Q7L2H7 / ENSSSCG00000013315 / ENSG00000149100
EIF4A1 / P60842 / ENSSSCG00000017957 / ENSG00000161960
EIF4A3 / P38919 / ENSSSCG00000017155 / ENSG00000141543
EIF4B / P23588 / ENSSSCG00000032028 / ENSG00000063046
EIF4E / P06730 / ENSSSCG00000028420 / ENSG00000151247
EIF4E2 / O60573 / ENSSSCG00000038466 / ENSG00000135930
EIF4G1 / Q04637 / ENSSSCG00000030255 / ENSG00000114867
EIF4G2 / P78344 / ENSSSCG00000013405 / ENSG00000110321
EIF4G3 / O43432 / ENSSSCG00000003512 / ENSG00000075151
EIF4H / Q15056 / ENSSSCG00000025966 / ENSG00000106682
EIF5 / P55010 / ENSSSCG00000034709 / ENSG00000100664
EIF5B / O60841 / ENSSSCG00000008180 / ENSG00000158417
EIF6 / P56537 / ENSSSCG00000022864 / ENSG00000242372
ELAC2 / Q9BQ52 / ENSSSCG00000028465 / ENSG00000006744
ELAVL1 / Q15717 / ENSSSCG00000013592 / ENSG00000066044
ELMO2 / Q96JJ3 / ENSSSCG00000023306 / ENSG00000062598
ELOA / Q14241 / ENSSSCG00000025440 / ENSG00000011007
ELOB / Q15370 / ENSSSCG00000008061 / ENSG00000103363
ELOVL1 / Q9BW60 / ENSSSCG00000035238 / ENSG00000066322
ELP1 / O95163 / ENSSSCG00000005441 / ENSG00000070061
ELP2 / Q6IA86 / ENSSSCG00000020898 / ENSG00000134759
ELP3 / Q9H9T3 / ENSSSCG00000009674 / ENSG00000134014
EMC1 / Q8N766 / ENSSSCG00000003489 / ENSG00000127463
EMC10 / Q5UCC4 / ENSSSCG00000039372 / ENSG00000161671
EMC2 / Q15006 / ENSSSCG00000006032 / ENSG00000104412
EMC3 / Q9P0I2 / ENSSSCG00000011562 / ENSG00000125037
EMC4 / Q5J8M3 / ENSSSCG00000039550 / ENSG00000128463
EMC7 / Q9NPA0 / ENSSSCG00000027051 / ENSG00000134153
EMC8 / O43402 / ENSSSCG00000045808 / ENSG00000131148
EMD / P50402 / ENSSSCG00000037777 / ENSG00000102119
EMILIN1 / Q9Y6C2 / ENSSSCG00000008557 / ENSG00000138080
EML2 / O95834 / ENSSSCG00000038229 / ENSG00000125746
EML3 / Q32P44 / ENSSSCG00000013061 / ENSG00000149499
EML4 / Q9HC35 / ENSSSCG00000008467 / ENSG00000143924
ENAH / Q8N8S7 / ENSSSCG00000038646 / ENSG00000154380
ENDOD1 / O94919 / ENSSSCG00000014965 / ENSG00000149218
Homologs
Adipose.Tissue
Brain
Colon
Heart
Kidney
Liver
Lung
Pancreas
S.Intestine
Spleen
Organs
Species
Human
Pig
Bins
1
2
3
4
5

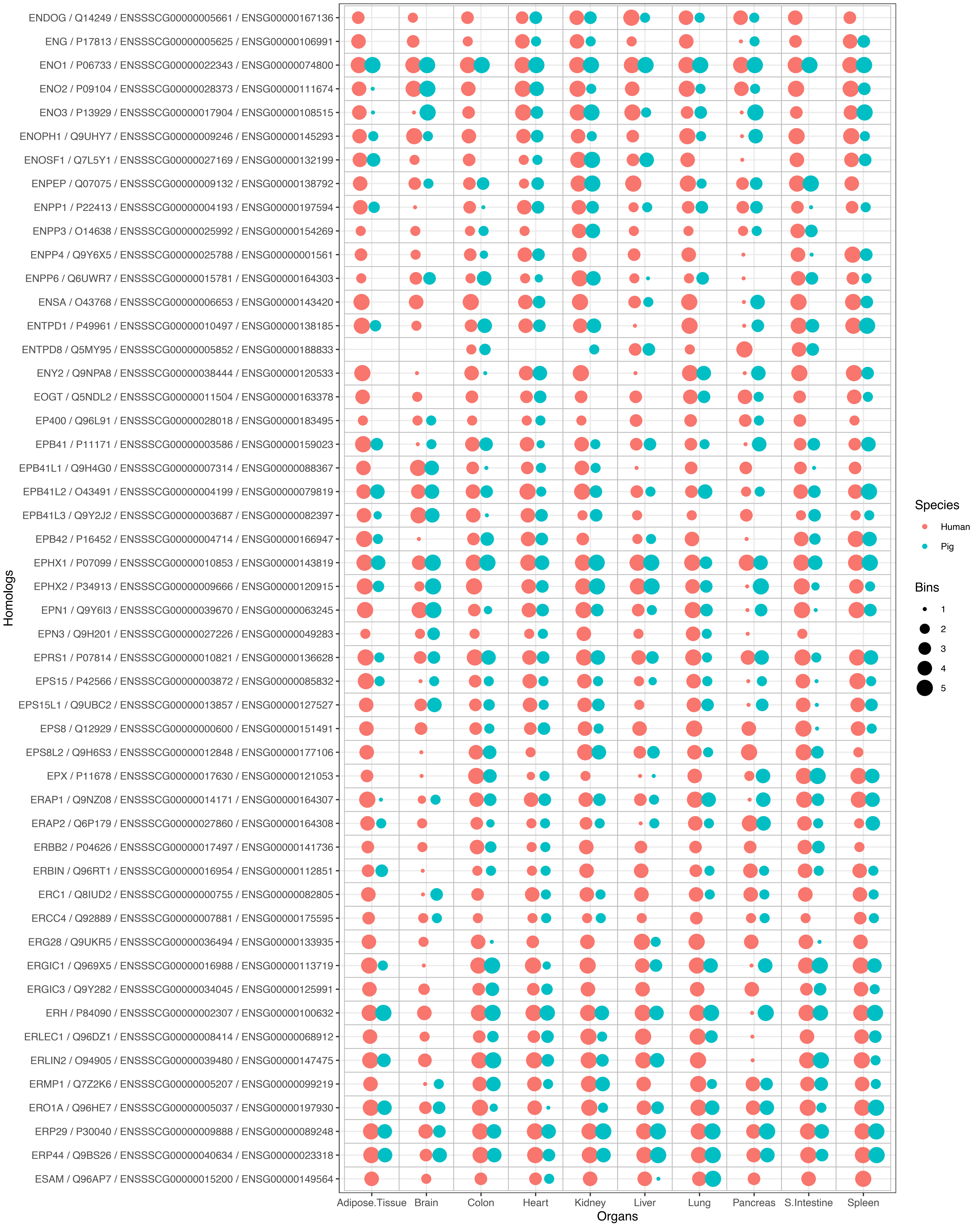
ENDOG / Q14249 / ENSSSCG00000005661 / ENSG00000167136
ENG / P17813 / ENSSSCG00000005625 / ENSG00000106991
ENO1 / P06733 / ENSSSCG00000022343 / ENSG00000074800
ENO2 / P09104 / ENSSSCG00000028373 / ENSG00000111674
ENO3 / P13929 / ENSSSCG00000017904 / ENSG00000108515
ENOPH1 / Q9UHY7 / ENSSSCG00000009246 / ENSG00000145293
ENOSF1 / Q7L5Y1 / ENSSSCG00000027169 / ENSG00000132199
ENPEP / Q07075 / ENSSSCG00000009132 / ENSG00000138792
ENPP1 / P22413 / ENSSSCG00000004193 / ENSG00000197594
ENPP3 / O14638 / ENSSSCG00000025992 / ENSG00000154269
ENPP4 / Q9Y6X5 / ENSSSCG00000025788 / ENSG00000001561
ENPP6 / Q6UWR7 / ENSSSCG00000015781 / ENSG00000164303
ENSA / O43768 / ENSSSCG00000006653 / ENSG00000143420
ENTPD1 / P49961 / ENSSSCG00000010497 / ENSG00000138185
ENTPD8 / Q5MY95 / ENSSSCG00000005852 / ENSG00000188833
ENY2 / Q9NPA8 / ENSSSCG00000038444 / ENSG00000120533
EOGT / Q5NDL2 / ENSSSCG00000011504 / ENSG00000163378
EP400 / Q96L91 / ENSSSCG00000028018 / ENSG00000183495
EPB41 / P11171 / ENSSSCG00000003586 / ENSG00000159023
EPB41L1 / Q9H4G0 / ENSSSCG00000007314 / ENSG00000088367
EPB41L2 / O43491 / ENSSSCG00000004199 / ENSG00000079819
EPB41L3 / Q9Y2J2 / ENSSSCG00000003687 / ENSG00000082397
EPB42 / P16452 / ENSSSCG00000004714 / ENSG00000166947
EPHX1 / P07099 / ENSSSCG00000010853 / ENSG00000143819
EPHX2 / P34913 / ENSSSCG00000009666 / ENSG00000120915
EPN1 / Q9Y6I3 / ENSSSCG00000039670 / ENSG00000063245
EPN3 / Q9H201 / ENSSSCG00000027226 / ENSG00000049283
EPRS1 / P07814 / ENSSSCG00000010821 / ENSG00000136628
EPS15 / P42566 / ENSSSCG00000003872 / ENSG00000085832
EPS15L1 / Q9UBC2 / ENSSSCG00000013857 / ENSG00000127527
EPS8 / Q12929 / ENSSSCG00000000600 / ENSG00000151491
EPS8L2 / Q9H6S3 / ENSSSCG00000012848 / ENSG00000177106
EPX / P11678 / ENSSSCG00000017630 / ENSG00000121053
ERAP1 / Q9NZ08 / ENSSSCG00000014171 / ENSG00000164307
ERAP2 / Q6P179 / ENSSSCG00000027860 / ENSG00000164308
ERBB2 / P04626 / ENSSSCG00000017497 / ENSG00000141736
ERBIN / Q96RT1 / ENSSSCG00000016954 / ENSG00000112851
ERC1 / Q8IUD2 / ENSSSCG00000000755 / ENSG00000082805
ERCC4 / Q92889 / ENSSSCG00000007881 / ENSG00000175595
ERG28 / Q9UKR5 / ENSSSCG00000036494 / ENSG00000133935
ERGIC1 / Q969X5 / ENSSSCG00000016988 / ENSG00000113719
ERGIC3 / Q9Y282 / ENSSSCG00000034045 / ENSG00000125991
ERH / P84090 / ENSSSCG00000002307 / ENSG00000100632
ERLEC1 / Q96DZ1 / ENSSSCG00000008414 / ENSG00000068912
ERLIN2 / O94905 / ENSSSCG00000039480 / ENSG00000147475
ERMP1 / Q7Z2K6 / ENSSSCG00000005207 / ENSG00000099219
ERO1A / Q96HE7 / ENSSSCG00000005037 / ENSG00000197930
ERP29 / P30040 / ENSSSCG00000009888 / ENSG00000089248
ERP44 / Q9BS26 / ENSSSCG00000040634 / ENSG00000023318
ESAM / Q96AP7 / ENSSSCG00000015200 / ENSG00000149564
Homologs
Adipose.Tissue
Brain
Colon
Heart
Kidney
Liver
Lung
Pancreas
S.Intestine
Spleen
Organs
Species
Human
Pig
Bins
1
2
3
4
5

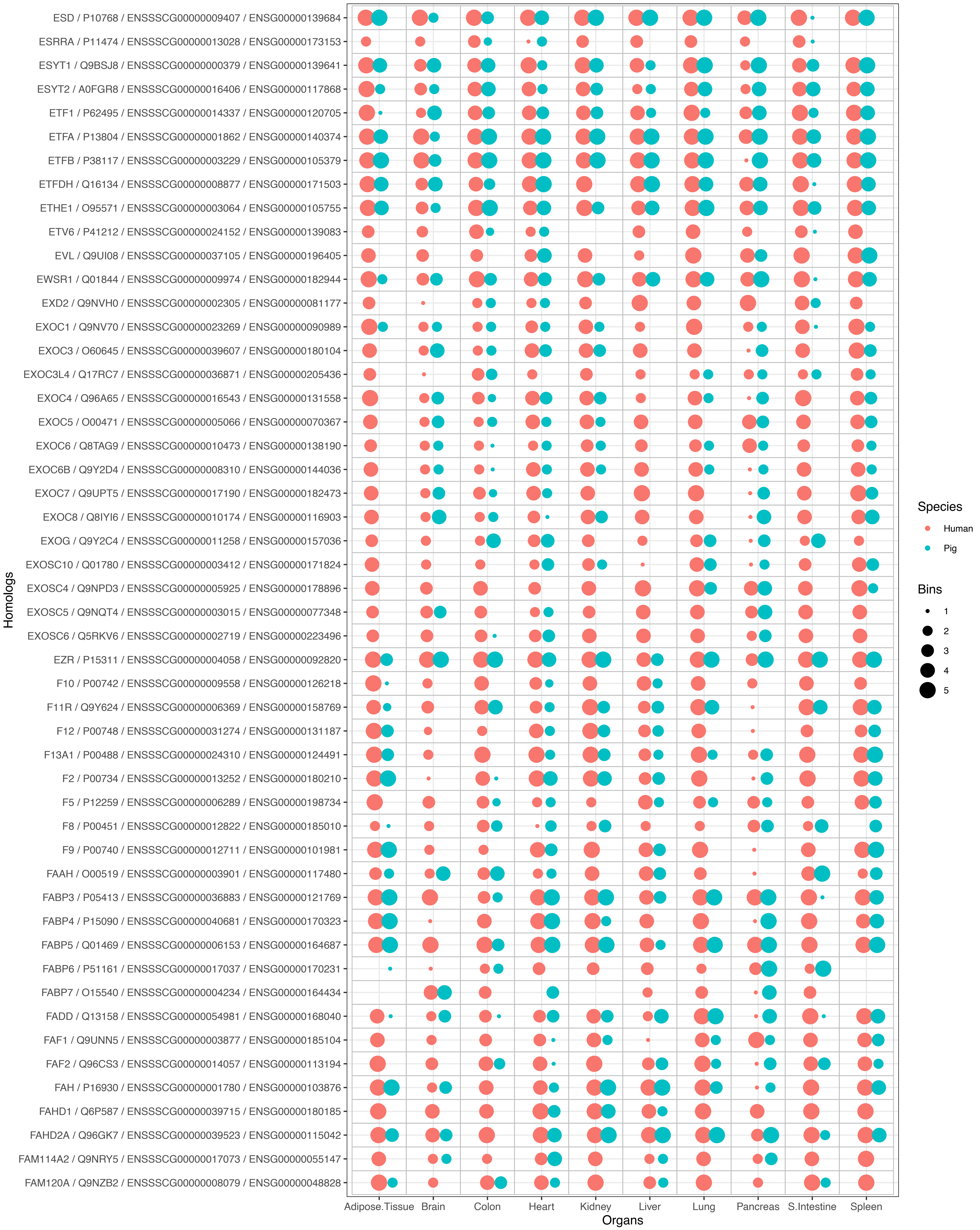
ESD / P10768 / ENSSSCG00000009407 / ENSG00000139684
ESRRA / P11474 / ENSSSCG00000013028 / ENSG00000173153
ESYT1 / Q9BSJ8 / ENSSSCG00000000379 / ENSG00000139641
ESYT2 / A0FGR8 / ENSSSCG00000016406 / ENSG00000117868
ETF1 / P62495 / ENSSSCG00000014337 / ENSG00000120705
ETFA / P13804 / ENSSSCG00000001862 / ENSG00000140374
ETFB / P38117 / ENSSSCG00000003229 / ENSG00000105379
ETFDH / Q16134 / ENSSSCG00000008877 / ENSG00000171503
ETHE1 / O95571 / ENSSSCG00000003064 / ENSG00000105755
ETV6 / P41212 / ENSSSCG00000024152 / ENSG00000139083
EVL / Q9UI08 / ENSSSCG00000037105 / ENSG00000196405
EWSR1 / Q01844 / ENSSSCG00000009974 / ENSG00000182944
EXD2 / Q9NVH0 / ENSSSCG00000002305 / ENSG00000081177
EXOC1 / Q9NV70 / ENSSSCG00000023269 / ENSG00000090989
EXOC3 / O60645 / ENSSSCG00000039607 / ENSG00000180104
EXOC3L4 / Q17RC7 / ENSSSCG00000036871 / ENSG00000205436
EXOC4 / Q96A65 / ENSSSCG00000016543 / ENSG00000131558
EXOC5 / O00471 / ENSSSCG00000005066 / ENSG00000070367
EXOC6 / Q8TAG9 / ENSSSCG00000010473 / ENSG00000138190
EXOC6B / Q9Y2D4 / ENSSSCG00000008310 / ENSG00000144036
EXOC7 / Q9UPT5 / ENSSSCG00000017190 / ENSG00000182473
EXOC8 / Q8IYI6 / ENSSSCG00000010174 / ENSG00000116903
EXOG / Q9Y2C4 / ENSSSCG00000011258 / ENSG00000157036
EXOSC10 / Q01780 / ENSSSCG00000003412 / ENSG00000171824
EXOSC4 / Q9NPD3 / ENSSSCG00000005925 / ENSG00000178896
EXOSC5 / Q9NQT4 / ENSSSCG00000003015 / ENSG00000077348
EXOSC6 / Q5RKV6 / ENSSSCG00000002719 / ENSG00000223496
EZR / P15311 / ENSSSCG00000004058 / ENSG00000092820
F10 / P00742 / ENSSSCG00000009558 / ENSG00000126218
F11R / Q9Y624 / ENSSSCG00000006369 / ENSG00000158769
F12 / P00748 / ENSSSCG00000031274 / ENSG00000131187
F13A1 / P00488 / ENSSSCG00000024310 / ENSG00000124491
F2 / P00734 / ENSSSCG00000013252 / ENSG00000180210
F5 / P12259 / ENSSSCG00000006289 / ENSG00000198734
F8 / P00451 / ENSSSCG00000012822 / ENSG00000185010
F9 / P00740 / ENSSSCG00000012711 / ENSG00000101981
FAAH / O00519 / ENSSSCG00000003901 / ENSG00000117480
FABP3 / P05413 / ENSSSCG00000036883 / ENSG00000121769
FABP4 / P15090 / ENSSSCG00000040681 / ENSG00000170323
FABP5 / Q01469 / ENSSSCG00000006153 / ENSG00000164687
FABP6 / P51161 / ENSSSCG00000017037 / ENSG00000170231
FABP7 / O15540 / ENSSSCG00000004234 / ENSG00000164434
FADD / Q13158 / ENSSSCG00000054981 / ENSG00000168040
FAF1 / Q9UNN5 / ENSSSCG00000003877 / ENSG00000185104
FAF2 / Q96CS3 / ENSSSCG00000014057 / ENSG00000113194
FAH / P16930 / ENSSSCG00000001780 / ENSG00000103876
FAHD1 / Q6P587 / ENSSSCG00000039715 / ENSG00000180185
FAHD2A / Q96GK7 / ENSSSCG00000039523 / ENSG00000115042
FAM114A2 / Q9NRY5 / ENSSSCG00000017073 / ENSG00000055147
FAM120A / Q9NZB2 / ENSSSCG00000008079 / ENSG00000048828
Homologs
Adipose.Tissue
Brain
Colon
Heart
Kidney
Liver
Lung
Pancreas
S.Intestine
Spleen
Organs
Species
Human
Pig
Bins
1
2
3
4
5

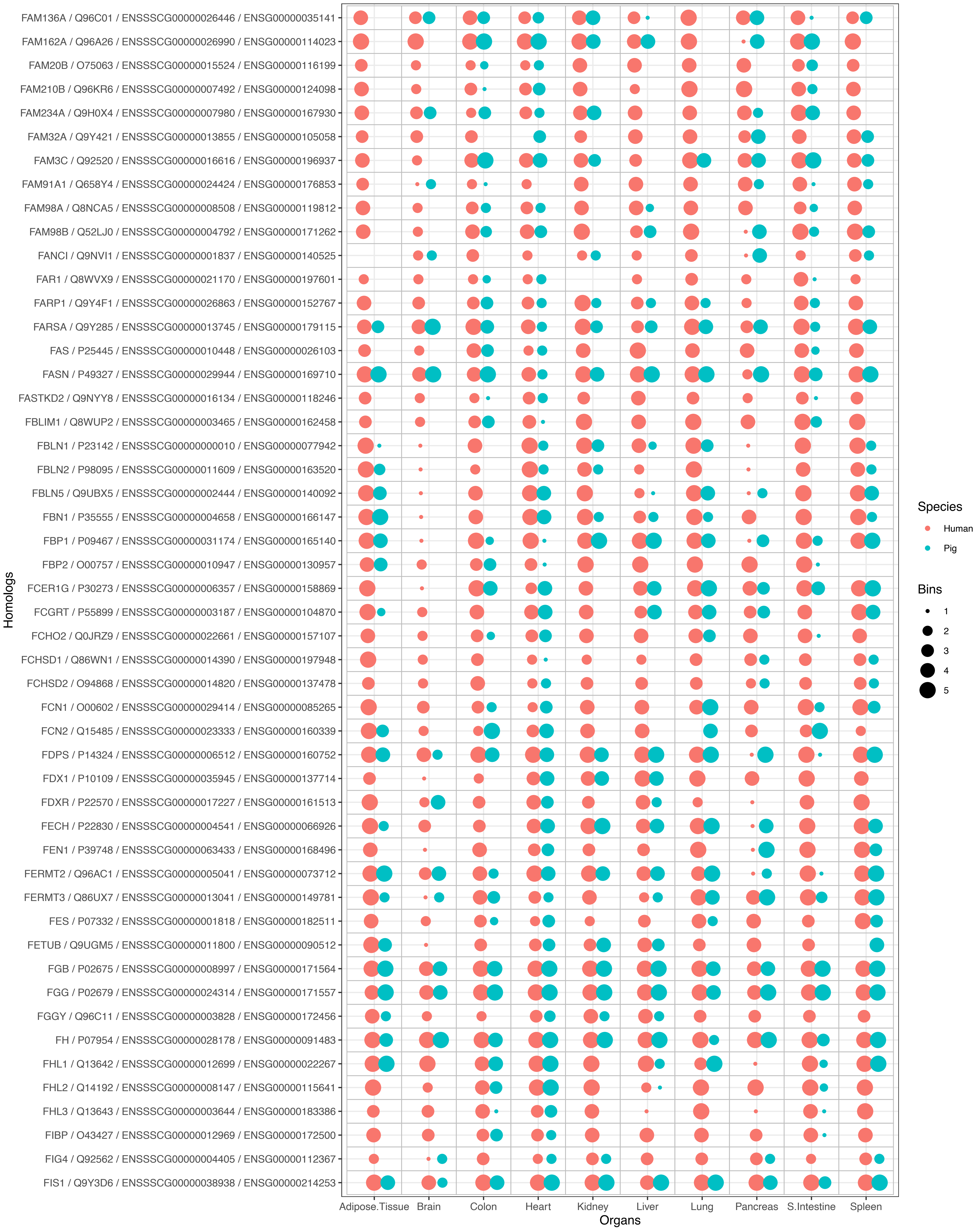
FAM136A / Q96C01 / ENSSSCG00000026446 / ENSG00000035141
FAM162A / Q96A26 / ENSSSCG00000026990 / ENSG00000114023
FAM20B / O75063 / ENSSSCG00000015524 / ENSG00000116199
FAM210B / Q96KR6 / ENSSSCG00000007492 / ENSG00000124098
FAM234A / Q9H0X4 / ENSSSCG00000007980 / ENSG00000167930
FAM32A / Q9Y421 / ENSSSCG00000013855 / ENSG00000105058
FAM3C / Q92520 / ENSSSCG00000016616 / ENSG00000196937
FAM91A1 / Q658Y4 / ENSSSCG00000024424 / ENSG00000176853
FAM98A / Q8NCA5 / ENSSSCG00000008508 / ENSG00000119812
FAM98B / Q52LJ0 / ENSSSCG00000004792 / ENSG00000171262
FANCI / Q9NVI1 / ENSSSCG00000001837 / ENSG00000140525
FAR1 / Q8WVX9 / ENSSSCG00000021170 / ENSG00000197601
FARP1 / Q9Y4F1 / ENSSSCG00000026863 / ENSG00000152767
FARSA / Q9Y285 / ENSSSCG00000013745 / ENSG00000179115
FAS / P25445 / ENSSSCG00000010448 / ENSG00000026103
FASN / P49327 / ENSSSCG00000029944 / ENSG00000169710
FASTKD2 / Q9NYY8 / ENSSSCG00000016134 / ENSG00000118246
FBLIM1 / Q8WUP2 / ENSSSCG00000003465 / ENSG00000162458
FBLN1 / P23142 / ENSSSCG00000000010 / ENSG00000077942
FBLN2 / P98095 / ENSSSCG00000011609 / ENSG00000163520
FBLN5 / Q9UBX5 / ENSSSCG00000002444 / ENSG00000140092
FBN1 / P35555 / ENSSSCG00000004658 / ENSG00000166147
FBP1 / P09467 / ENSSSCG00000031174 / ENSG00000165140
FBP2 / O00757 / ENSSSCG00000010947 / ENSG00000130957
FCER1G / P30273 / ENSSSCG00000006357 / ENSG00000158869
FCGRT / P55899 / ENSSSCG00000003187 / ENSG00000104870
FCHO2 / Q0JRZ9 / ENSSSCG00000022661 / ENSG00000157107
FCHSD1 / Q86WN1 / ENSSSCG00000014390 / ENSG00000197948
FCHSD2 / O94868 / ENSSSCG00000014820 / ENSG00000137478
FCN1 / O00602 / ENSSSCG00000029414 / ENSG00000085265
FCN2 / Q15485 / ENSSSCG00000023333 / ENSG00000160339
FDPS / P14324 / ENSSSCG00000006512 / ENSG00000160752
FDX1 / P10109 / ENSSSCG00000035945 / ENSG00000137714
FDXR / P22570 / ENSSSCG00000017227 / ENSG00000161513
FECH / P22830 / ENSSSCG00000004541 / ENSG00000066926
FEN1 / P39748 / ENSSSCG00000063433 / ENSG00000168496
FERMT2 / Q96AC1 / ENSSSCG00000005041 / ENSG00000073712
FERMT3 / Q86UX7 / ENSSSCG00000013041 / ENSG00000149781
FES / P07332 / ENSSSCG00000001818 / ENSG00000182511
FETUB / Q9UGM5 / ENSSSCG00000011800 / ENSG00000090512
FGB / P02675 / ENSSSCG00000008997 / ENSG00000171564
FGG / P02679 / ENSSSCG00000024314 / ENSG00000171557
FGGY / Q96C11 / ENSSSCG00000003828 / ENSG00000172456
FH / P07954 / ENSSSCG00000028178 / ENSG00000091483
FHL1 / Q13642 / ENSSSCG00000012699 / ENSG00000022267
FHL2 / Q14192 / ENSSSCG00000008147 / ENSG00000115641
FHL3 / Q13643 / ENSSSCG00000003644 / ENSG00000183386
FIBP / O43427 / ENSSSCG00000012969 / ENSG00000172500
FIG4 / Q92562 / ENSSSCG00000004405 / ENSG00000112367
FIS1 / Q9Y3D6 / ENSSSCG00000038938 / ENSG00000214253
Homologs
Adipose.Tissue
Brain
Colon
Heart
Kidney
Liver
Lung
Pancreas
S.Intestine
Spleen
Organs
Species
Human
Pig
Bins
1
2
3
4
5

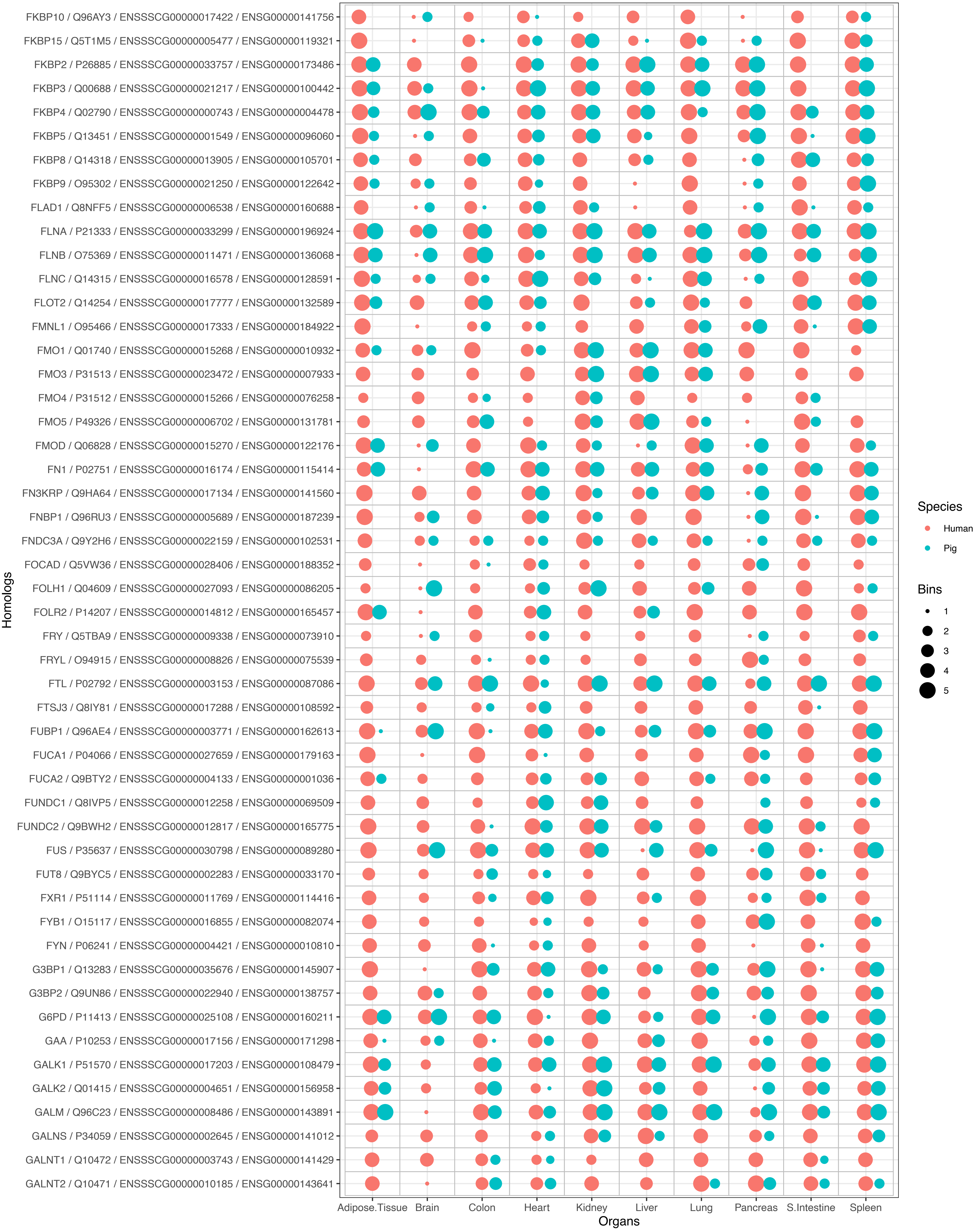
FKBP10 / Q96AY3 / ENSSSCG00000017422 / ENSG00000141756
FKBP15 / Q5T1M5 / ENSSSCG00000005477 / ENSG00000119321
FKBP2 / P26885 / ENSSSCG00000033757 / ENSG00000173486
FKBP3 / Q00688 / ENSSSCG00000021217 / ENSG00000100442
FKBP4 / Q02790 / ENSSSCG00000000743 / ENSG00000004478
FKBP5 / Q13451 / ENSSSCG00000001549 / ENSG00000096060
FKBP8 / Q14318 / ENSSSCG00000013905 / ENSG00000105701
FKBP9 / O95302 / ENSSSCG00000021250 / ENSG00000122642
FLAD1 / Q8NFF5 / ENSSSCG00000006538 / ENSG00000160688
FLNA / P21333 / ENSSSCG00000033299 / ENSG00000196924
FLNB / O75369 / ENSSSCG00000011471 / ENSG00000136068
FLNC / Q14315 / ENSSSCG00000016578 / ENSG00000128591
FLOT2 / Q14254 / ENSSSCG00000017777 / ENSG00000132589
FMNL1 / O95466 / ENSSSCG00000017333 / ENSG00000184922
FMO1 / Q01740 / ENSSSCG00000015268 / ENSG00000010932
FMO3 / P31513 / ENSSSCG00000023472 / ENSG00000007933
FMO4 / P31512 / ENSSSCG00000015266 / ENSG00000076258
FMO5 / P49326 / ENSSSCG00000006702 / ENSG00000131781
FMOD / Q06828 / ENSSSCG00000015270 / ENSG00000122176
FN1 / P02751 / ENSSSCG00000016174 / ENSG00000115414
FN3KRP / Q9HA64 / ENSSSCG00000017134 / ENSG00000141560
FNBP1 / Q96RU3 / ENSSSCG00000005689 / ENSG00000187239
FNDC3A / Q9Y2H6 / ENSSSCG00000022159 / ENSG00000102531
FOCAD / Q5VW36 / ENSSSCG00000028406 / ENSG00000188352
FOLH1 / Q04609 / ENSSSCG00000027093 / ENSG00000086205
FOLR2 / P14207 / ENSSSCG00000014812 / ENSG00000165457
FRY / Q5TBA9 / ENSSSCG00000009338 / ENSG00000073910
FRYL / O94915 / ENSSSCG00000008826 / ENSG00000075539
FTL / P02792 / ENSSSCG00000003153 / ENSG00000087086
FTSJ3 / Q8IY81 / ENSSSCG00000017288 / ENSG00000108592
FUBP1 / Q96AE4 / ENSSSCG00000003771 / ENSG00000162613
FUCA1 / P04066 / ENSSSCG00000027659 / ENSG00000179163
FUCA2 / Q9BTY2 / ENSSSCG00000004133 / ENSG00000001036
FUNDC1 / Q8IVP5 / ENSSSCG00000012258 / ENSG00000069509
FUNDC2 / Q9BWH2 / ENSSSCG00000012817 / ENSG00000165775
FUS / P35637 / ENSSSCG00000030798 / ENSG00000089280
FUT8 / Q9BYC5 / ENSSSCG00000002283 / ENSG00000033170
FXR1 / P51114 / ENSSSCG00000011769 / ENSG00000114416
FYB1 / O15117 / ENSSSCG00000016855 / ENSG00000082074
FYN / P06241 / ENSSSCG00000004421 / ENSG00000010810
G3BP1 / Q13283 / ENSSSCG00000035676 / ENSG00000145907
G3BP2 / Q9UN86 / ENSSSCG00000022940 / ENSG00000138757
G6PD / P11413 / ENSSSCG00000025108 / ENSG00000160211
GAA / P10253 / ENSSSCG00000017156 / ENSG00000171298
GALK1 / P51570 / ENSSSCG00000017203 / ENSG00000108479
GALK2 / Q01415 / ENSSSCG00000004651 / ENSG00000156958
GALM / Q96C23 / ENSSSCG00000008486 / ENSG00000143891
GALNS / P34059 / ENSSSCG00000002645 / ENSG00000141012
GALNT1 / Q10472 / ENSSSCG00000003743 / ENSG00000141429
GALNT2 / Q10471 / ENSSSCG00000010185 / ENSG00000143641
Homologs
Adipose.Tissue
Brain
Colon
Heart
Kidney
Liver
Lung
Pancreas
S.Intestine
Spleen
Organs
Species
Human
Pig
Bins
1
2
3
4
5

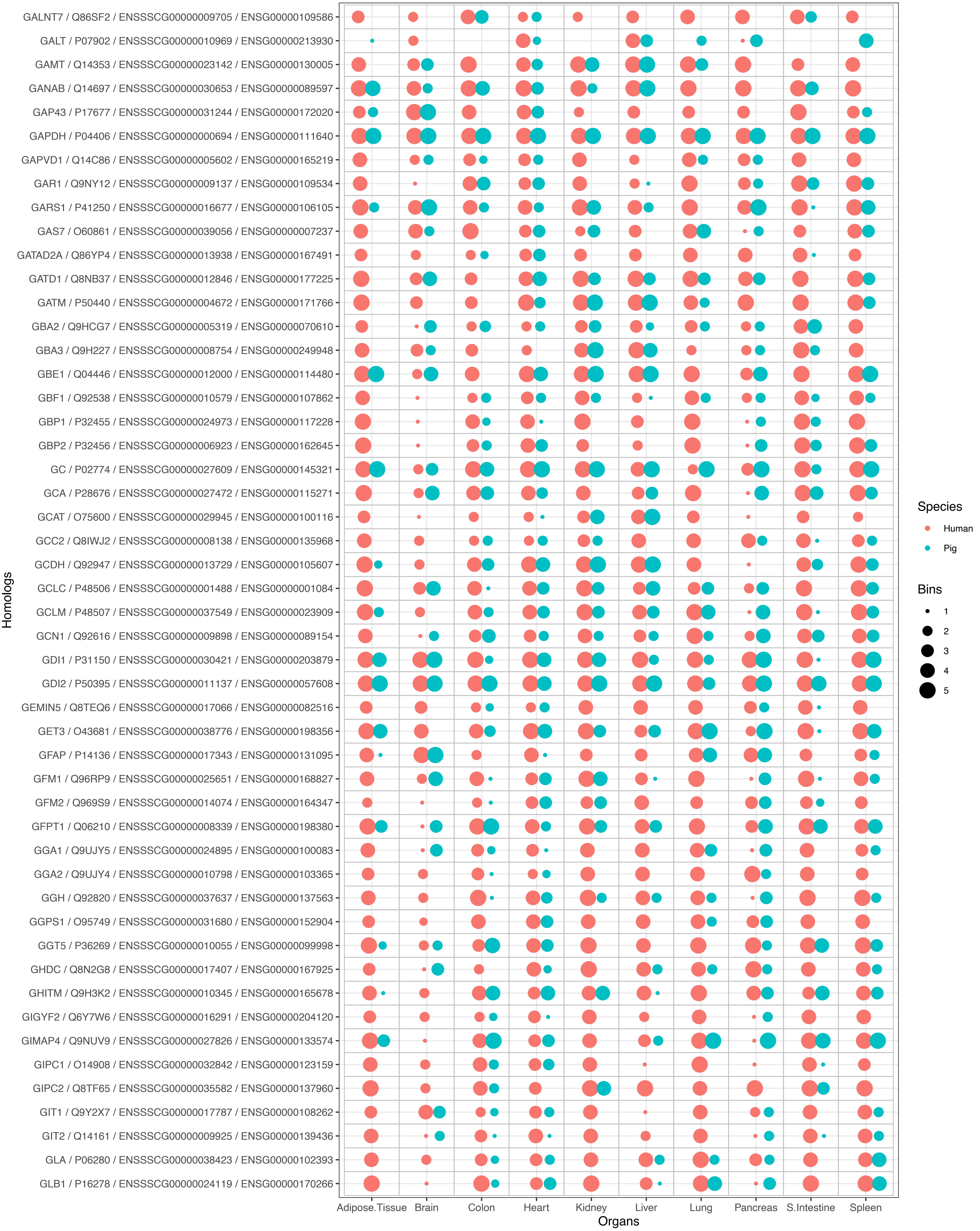

GALNT7 / Q86SF2 / ENSSSCG00000009705 / ENSG00000109586
GALT / P07902 / ENSSSCG00000010969 / ENSG00000213930
GAMT / Q14353 / ENSSSCG00000023142 / ENSG00000130005
GANAB / Q14697 / ENSSSCG00000030653 / ENSG00000089597
GAP43 / P17677 / ENSSSCG00000031244 / ENSG00000172020
GAPDH / P04406 / ENSSSCG00000000694 / ENSG00000111640
GAPVD1 / Q14C86 / ENSSSCG00000005602 / ENSG00000165219
GAR1 / Q9NY12 / ENSSSCG00000009137 / ENSG00000109534
GARS1 / P41250 / ENSSSCG00000016677 / ENSG00000106105
GAS7 / O60861 / ENSSSCG00000039056 / ENSG00000007237
GATAD2A / Q86YP4 / ENSSSCG00000013938 / ENSG00000167491
GATD1 / Q8NB37 / ENSSSCG00000012846 / ENSG00000177225
GATM / P50440 / ENSSSCG00000004672 / ENSG00000171766
GBA2 / Q9HCG7 / ENSSSCG00000005319 / ENSG00000070610
GBA3 / Q9H227 / ENSSSCG00000008754 / ENSG00000249948
GBE1 / Q04446 / ENSSSCG00000012000 / ENSG00000114480
GBF1 / Q92538 / ENSSSCG00000010579 / ENSG00000107862
GBP1 / P32455 / ENSSSCG00000024973 / ENSG00000117228
GBP2 / P32456 / ENSSSCG00000006923 / ENSG00000162645
GC / P02774 / ENSSSCG00000027609 / ENSG00000145321
GCA / P28676 / ENSSSCG00000027472 / ENSG00000115271
GCAT / O75600 / ENSSSCG00000029945 / ENSG00000100116
GCC2 / Q8IWJ2 / ENSSSCG00000008138 / ENSG00000135968
GCDH / Q92947 / ENSSSCG00000013729 / ENSG00000105607
GCLC / P48506 / ENSSSCG00000001488 / ENSG00000001084
GCLM / P48507 / ENSSSCG00000037549 / ENSG00000023909
GCN1 / Q92616 / ENSSSCG00000009898 / ENSG00000089154
GDI1 / P31150 / ENSSSCG00000030421 / ENSG00000203879
GDI2 / P50395 / ENSSSCG00000011137 / ENSG00000057608
GEMIN5 / Q8TEQ6 / ENSSSCG00000017066 / ENSG00000082516
GET3 / O43681 / ENSSSCG00000038776 / ENSG00000198356
GFAP / P14136 / ENSSSCG00000017343 / ENSG00000131095
GFM1 / Q96RP9 / ENSSSCG00000025651 / ENSG00000168827
GFM2 / Q969S9 / ENSSSCG00000014074 / ENSG00000164347
GFPT1 / Q06210 / ENSSSCG00000008339 / ENSG00000198380
GGA1 / Q9UJY5 / ENSSSCG00000024895 / ENSG00000100083
GGA2 / Q9UJY4 / ENSSSCG00000010798 / ENSG00000103365
GGH / Q92820 / ENSSSCG00000037637 / ENSG00000137563
GGPS1 / O95749 / ENSSSCG00000031680 / ENSG00000152904
GGT5 / P36269 / ENSSSCG00000010055 / ENSG00000099998
GHDC / Q8N2G8 / ENSSSCG00000017407 / ENSG00000167925
GHITM / Q9H3K2 / ENSSSCG00000010345 / ENSG00000165678
GIGYF2 / Q6Y7W6 / ENSSSCG00000016291 / ENSG00000204120
GIMAP4 / Q9NUV9 / ENSSSCG00000027826 / ENSG00000133574
GIPC1 / O14908 / ENSSSCG00000032842 / ENSG00000123159
GIPC2 / Q8TF65 / ENSSSCG00000035582 / ENSG00000137960
GIT1 / Q9Y2X7 / ENSSSCG00000017787 / ENSG00000108262
GIT2 / Q14161 / ENSSSCG00000009925 / ENSG00000139436
GLA / P06280 / ENSSSCG00000038423 / ENSG00000102393
GLB1 / P16278 / ENSSSCG00000024119 / ENSG00000170266
Homologs
Adipose.Tissue
Brain
Colon
Heart
Kidney
Liver
Lung
Pancreas
S.Intestine
Spleen
Organs
Species
Human
Pig
Bins
1
2
3
4
5

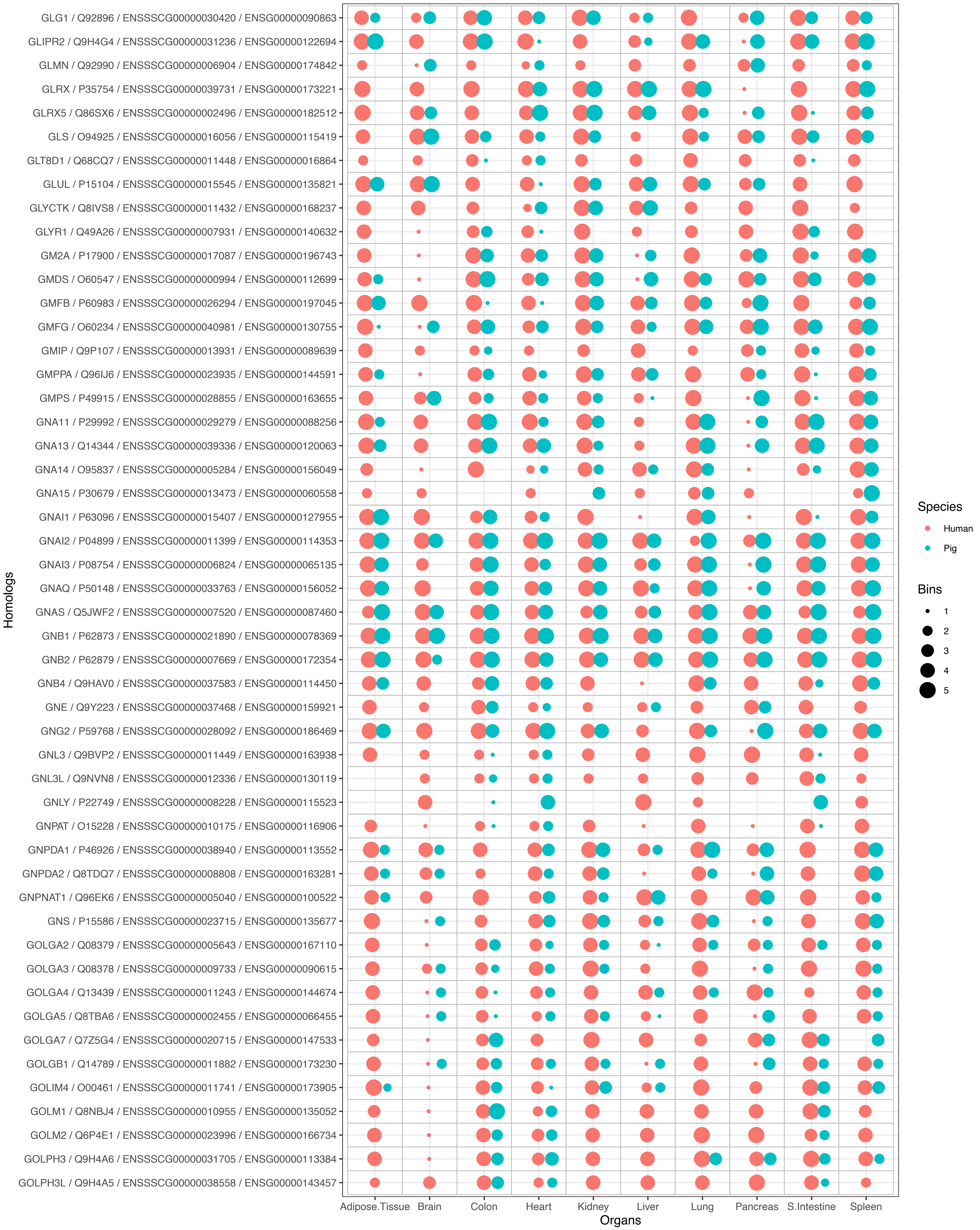
GLG1 / Q92896 / ENSSSCG00000030420 / ENSG00000090863
GLIPR2 / Q9H4G4 / ENSSSCG00000031236 / ENSG00000122694
GLMN / Q92990 / ENSSSCG00000006904 / ENSG00000174842
GLRX / P35754 / ENSSSCG00000039731 / ENSG00000173221
GLRX5 / Q86SX6 / ENSSSCG00000002496 / ENSG00000182512
GLS / O94925 / ENSSSCG00000016056 / ENSG00000115419
GLT8D1 / Q68CQ7 / ENSSSCG00000011448 / ENSG00000016864
GLUL / P15104 / ENSSSCG00000015545 / ENSG00000135821
GLYCTK / Q8IVS8 / ENSSSCG00000011432 / ENSG00000168237
GLYR1 / Q49A26 / ENSSSCG00000007931 / ENSG00000140632
GM2A / P17900 / ENSSSCG00000017087 / ENSG00000196743
GMDS / O60547 / ENSSSCG00000000994 / ENSG00000112699
GMFB / P60983 / ENSSSCG00000026294 / ENSG00000197045
GMFG / O60234 / ENSSSCG00000040981 / ENSG00000130755
GMIP / Q9P107 / ENSSSCG00000013931 / ENSG00000089639
GMPPA / Q96IJ6 / ENSSSCG00000023935 / ENSG00000144591
GMPS / P49915 / ENSSSCG00000028855 / ENSG00000163655
GNA11 / P29992 / ENSSSCG00000029279 / ENSG00000088256
GNA13 / Q14344 / ENSSSCG00000039336 / ENSG00000120063
GNA14 / O95837 / ENSSSCG00000005284 / ENSG00000156049
GNA15 / P30679 / ENSSSCG00000013473 / ENSG00000060558
GNAI1 / P63096 / ENSSSCG00000015407 / ENSG00000127955
GNAI2 / P04899 / ENSSSCG00000011399 / ENSG00000114353
GNAI3 / P08754 / ENSSSCG00000006824 / ENSG00000065135
GNAQ / P50148 / ENSSSCG00000033763 / ENSG00000156052
GNAS / Q5JWF2 / ENSSSCG00000007520 / ENSG00000087460
GNB1 / P62873 / ENSSSCG00000021890 / ENSG00000078369
GNB2 / P62879 / ENSSSCG00000007669 / ENSG00000172354
GNB4 / Q9HAV0 / ENSSSCG00000037583 / ENSG00000114450
GNE / Q9Y223 / ENSSSCG00000037468 / ENSG00000159921
GNG2 / P59768 / ENSSSCG00000028092 / ENSG00000186469
GNL3 / Q9BVP2 / ENSSSCG00000011449 / ENSG00000163938
GNL3L / Q9NVN8 / ENSSSCG00000012336 / ENSG00000130119
GNLY / P22749 / ENSSSCG00000008228 / ENSG00000115523
GNPAT / O15228 / ENSSSCG00000010175 / ENSG00000116906
GNPDA1 / P46926 / ENSSSCG00000038940 / ENSG00000113552
GNPDA2 / Q8TDQ7 / ENSSSCG00000008808 / ENSG00000163281
GNPNAT1 / Q96EK6 / ENSSSCG00000005040 / ENSG00000100522
GNS / P15586 / ENSSSCG00000023715 / ENSG00000135677
GOLGA2 / Q08379 / ENSSSCG00000005643 / ENSG00000167110
GOLGA3 / Q08378 / ENSSSCG00000009733 / ENSG00000090615
GOLGA4 / Q13439 / ENSSSCG00000011243 / ENSG00000144674
GOLGA5 / Q8TBA6 / ENSSSCG00000002455 / ENSG00000066455
GOLGA7 / Q7Z5G4 / ENSSSCG00000020715 / ENSG00000147533
GOLGB1 / Q14789 / ENSSSCG00000011882 / ENSG00000173230
GOLIM4 / O00461 / ENSSSCG00000011741 / ENSG00000173905
GOLM1 / Q8NBJ4 / ENSSSCG00000010955 / ENSG00000135052
GOLM2 / Q6P4E1 / ENSSSCG00000023996 / ENSG00000166734
GOLPH3 / Q9H4A6 / ENSSSCG00000031705 / ENSG00000113384
GOLPH3L / Q9H4A5 / ENSSSCG00000038558 / ENSG00000143457
Homologs
Adipose.Tissue
Brain
Colon
Heart
Kidney
Liver
Lung
Pancreas
S.Intestine
Spleen
Organs
Species
Human
Pig
Bins
1
2
3
4
5

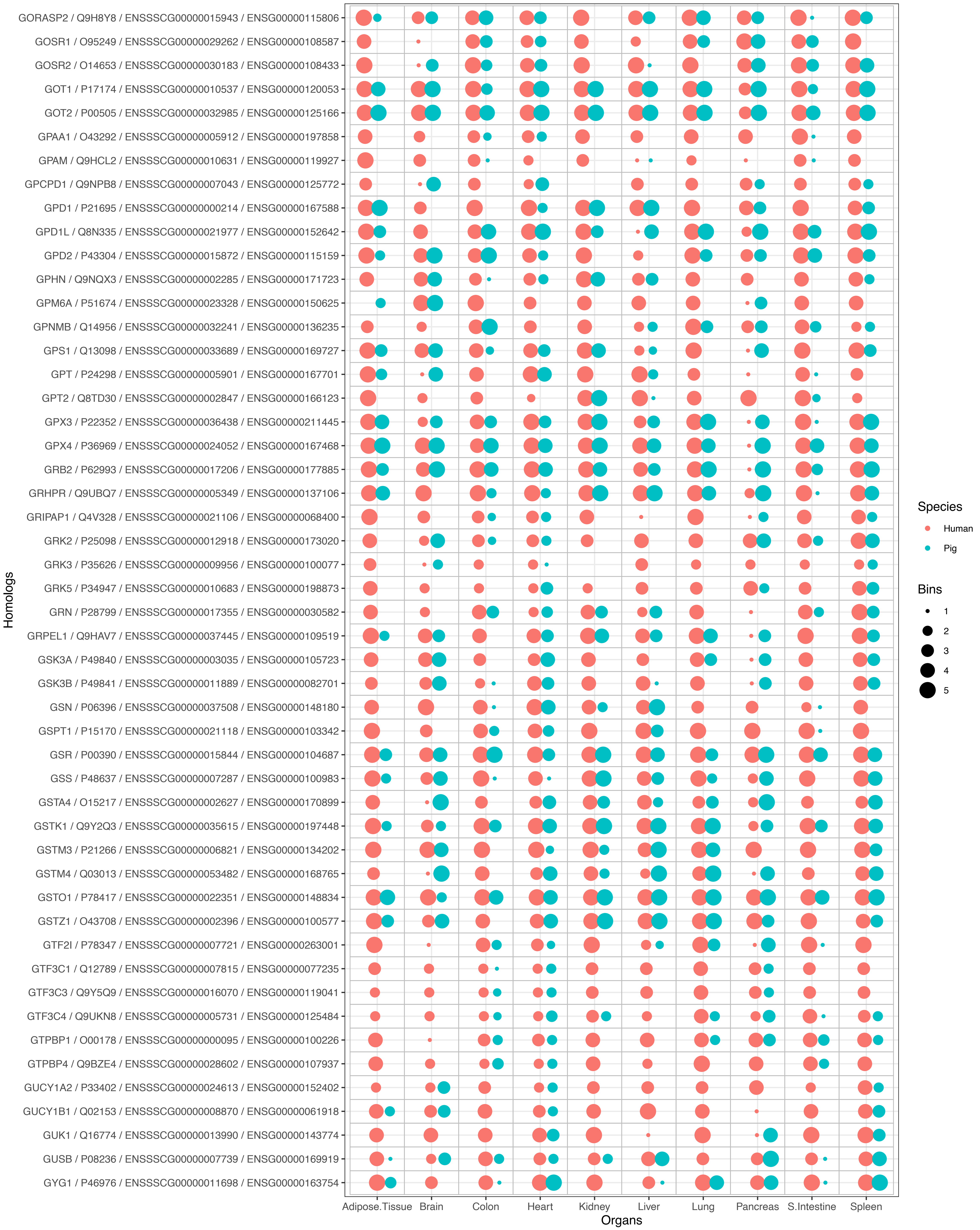

GORASP2 / Q9H8Y8 / ENSSSCG00000015943 / ENSG00000115806
GOSR1 / O95249 / ENSSSCG00000029262 / ENSG00000108587
GOSR2 / O14653 / ENSSSCG00000030183 / ENSG00000108433
GOT1 / P17174 / ENSSSCG00000010537 / ENSG00000120053
GOT2 / P00505 / ENSSSCG00000032985 / ENSG00000125166
GPAA1 / O43292 / ENSSSCG00000005912 / ENSG00000197858
GPAM / Q9HCL2 / ENSSSCG00000010631 / ENSG00000119927
GPCPD1 / Q9NPB8 / ENSSSCG00000007043 / ENSG00000125772
GPD1 / P21695 / ENSSSCG00000000214 / ENSG00000167588
GPD1L / Q8N335 / ENSSSCG00000021977 / ENSG00000152642
GPD2 / P43304 / ENSSSCG00000015872 / ENSG00000115159
GPHN / Q9NQX3 / ENSSSCG00000002285 / ENSG00000171723
GPM6A / P51674 / ENSSSCG00000023328 / ENSG00000150625
GPNMB / Q14956 / ENSSSCG00000032241 / ENSG00000136235
GPS1 / Q13098 / ENSSSCG00000033689 / ENSG00000169727
GPT / P24298 / ENSSSCG00000005901 / ENSG00000167701
GPT2 / Q8TD30 / ENSSSCG00000002847 / ENSG00000166123
GPX3 / P22352 / ENSSSCG00000036438 / ENSG00000211445
GPX4 / P36969 / ENSSSCG00000024052 / ENSG00000167468
GRB2 / P62993 / ENSSSCG00000017206 / ENSG00000177885
GRHPR / Q9UBQ7 / ENSSSCG00000005349 / ENSG00000137106
GRIPAP1 / Q4V328 / ENSSSCG00000021106 / ENSG00000068400
GRK2 / P25098 / ENSSSCG00000012918 / ENSG00000173020
GRK3 / P35626 / ENSSSCG00000009956 / ENSG00000100077
GRK5 / P34947 / ENSSSCG00000010683 / ENSG00000198873
GRN / P28799 / ENSSSCG00000017355 / ENSG00000030582
GRPEL1 / Q9HAV7 / ENSSSCG00000037445 / ENSG00000109519
GSK3A / P49840 / ENSSSCG00000003035 / ENSG00000105723
GSK3B / P49841 / ENSSSCG00000011889 / ENSG00000082701
GSN / P06396 / ENSSSCG00000037508 / ENSG00000148180
GSPT1 / P15170 / ENSSSCG00000021118 / ENSG00000103342
GSR / P00390 / ENSSSCG00000015844 / ENSG00000104687
GSS / P48637 / ENSSSCG00000007287 / ENSG00000100983
GSTA4 / O15217 / ENSSSCG00000002627 / ENSG00000170899
GSTK1 / Q9Y2Q3 / ENSSSCG00000035615 / ENSG00000197448
GSTM3 / P21266 / ENSSSCG00000006821 / ENSG00000134202
GSTM4 / Q03013 / ENSSSCG00000053482 / ENSG00000168765
GSTO1 / P78417 / ENSSSCG00000022351 / ENSG00000148834
GSTZ1 / O43708 / ENSSSCG00000002396 / ENSG00000100577
GTF2I / P78347 / ENSSSCG00000007721 / ENSG00000263001
GTF3C1 / Q12789 / ENSSSCG00000007815 / ENSG00000077235
GTF3C3 / Q9Y5Q9 / ENSSSCG00000016070 / ENSG00000119041
GTF3C4 / Q9UKN8 / ENSSSCG00000005731 / ENSG00000125484
GTPBP1 / O00178 / ENSSSCG00000000095 / ENSG00000100226
GTPBP4 / Q9BZE4 / ENSSSCG00000028602 / ENSG00000107937
GUCY1A2 / P33402 / ENSSSCG00000024613 / ENSG00000152402
GUCY1B1 / Q02153 / ENSSSCG00000008870 / ENSG00000061918
GUK1 / Q16774 / ENSSSCG00000013990 / ENSG00000143774
GUSB / P08236 / ENSSSCG00000007739 / ENSG00000169919
GYG1 / P46976 / ENSSSCG00000011698 / ENSG00000163754
Homologs
Adipose.Tissue
Brain
Colon
Heart
Kidney
Liver
Lung
Pancreas
S.Intestine
Spleen
Organs
Species
Human
Pig
Bins
1
2
3
4
5

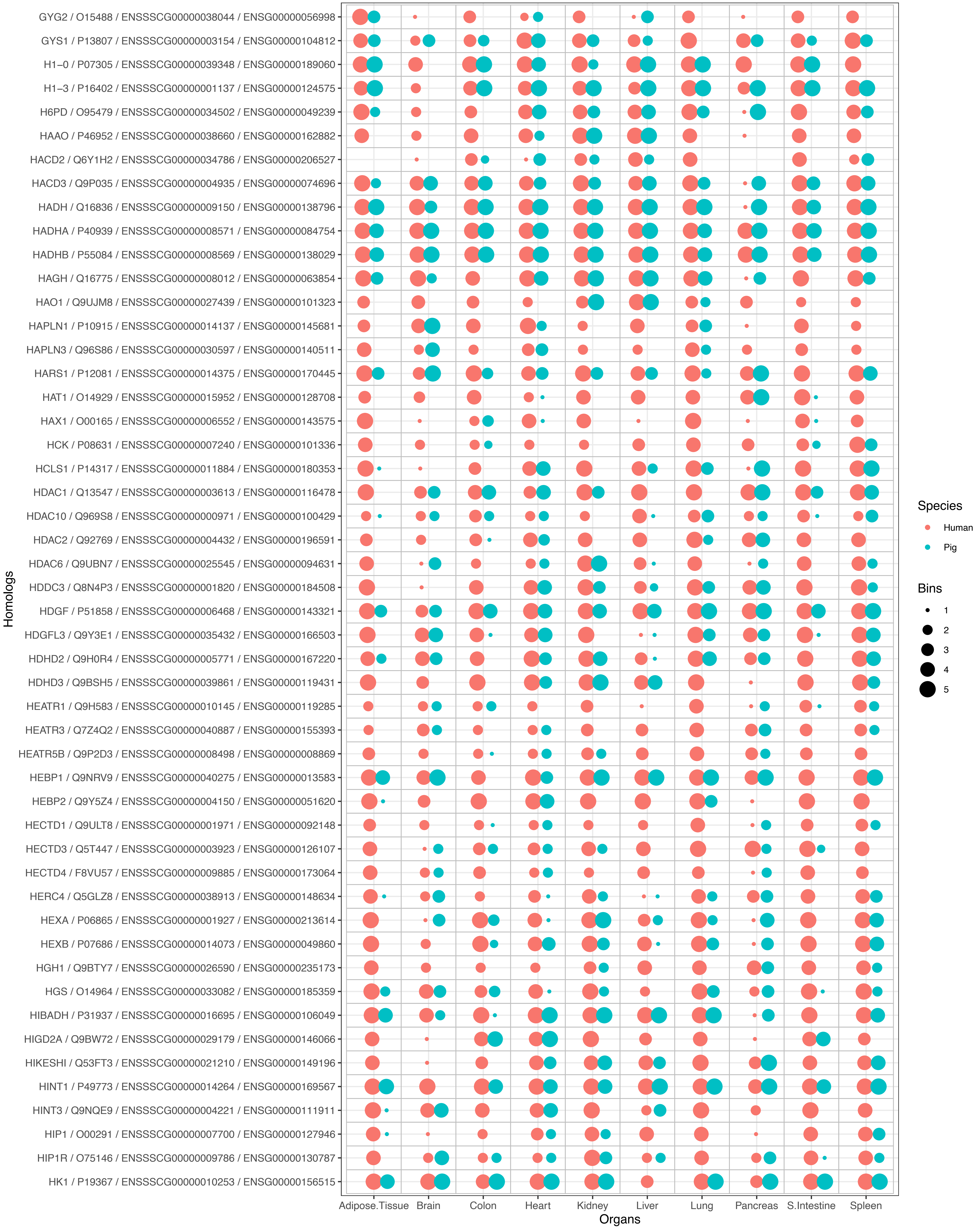

GYG2 / O15488 / ENSSSCG00000038044 / ENSG00000056998
GYS1 / P13807 / ENSSSCG00000003154 / ENSG00000104812
H1−0 / P07305 / ENSSSCG00000039348 / ENSG00000189060
H1−3 / P16402 / ENSSSCG00000001137 / ENSG00000124575
H6PD / O95479 / ENSSSCG00000034502 / ENSG00000049239
HAAO / P46952 / ENSSSCG00000038660 / ENSG00000162882
HACD2 / Q6Y1H2 / ENSSSCG00000034786 / ENSG00000206527
HACD3 / Q9P035 / ENSSSCG00000004935 / ENSG00000074696
HADH / Q16836 / ENSSSCG00000009150 / ENSG00000138796
HADHA / P40939 / ENSSSCG00000008571 / ENSG00000084754
HADHB / P55084 / ENSSSCG00000008569 / ENSG00000138029
HAGH / Q16775 / ENSSSCG00000008012 / ENSG00000063854
HAO1 / Q9UJM8 / ENSSSCG00000027439 / ENSG00000101323
HAPLN1 / P10915 / ENSSSCG00000014137 / ENSG00000145681
HAPLN3 / Q96S86 / ENSSSCG00000030597 / ENSG00000140511
HARS1 / P12081 / ENSSSCG00000014375 / ENSG00000170445
HAT1 / O14929 / ENSSSCG00000015952 / ENSG00000128708
HAX1 / O00165 / ENSSSCG00000006552 / ENSG00000143575
HCK / P08631 / ENSSSCG00000007240 / ENSG00000101336
HCLS1 / P14317 / ENSSSCG00000011884 / ENSG00000180353
HDAC1 / Q13547 / ENSSSCG00000003613 / ENSG00000116478
HDAC10 / Q969S8 / ENSSSCG00000000971 / ENSG00000100429
HDAC2 / Q92769 / ENSSSCG00000004432 / ENSG00000196591
HDAC6 / Q9UBN7 / ENSSSCG00000025545 / ENSG00000094631
HDDC3 / Q8N4P3 / ENSSSCG00000001820 / ENSG00000184508
HDGF / P51858 / ENSSSCG00000006468 / ENSG00000143321
HDGFL3 / Q9Y3E1 / ENSSSCG00000035432 / ENSG00000166503
HDHD2 / Q9H0R4 / ENSSSCG00000005771 / ENSG00000167220
HDHD3 / Q9BSH5 / ENSSSCG00000039861 / ENSG00000119431
HEATR1 / Q9H583 / ENSSSCG00000010145 / ENSG00000119285
HEATR3 / Q7Z4Q2 / ENSSSCG00000040887 / ENSG00000155393
HEATR5B / Q9P2D3 / ENSSSCG00000008498 / ENSG00000008869
HEBP1 / Q9NRV9 / ENSSSCG00000040275 / ENSG00000013583
HEBP2 / Q9Y5Z4 / ENSSSCG00000004150 / ENSG00000051620
HECTD1 / Q9ULT8 / ENSSSCG00000001971 / ENSG00000092148
HECTD3 / Q5T447 / ENSSSCG00000003923 / ENSG00000126107
HECTD4 / F8VU57 / ENSSSCG00000009885 / ENSG00000173064
HERC4 / Q5GLZ8 / ENSSSCG00000038913 / ENSG00000148634
HEXA / P06865 / ENSSSCG00000001927 / ENSG00000213614
HEXB / P07686 / ENSSSCG00000014073 / ENSG00000049860
HGH1 / Q9BTY7 / ENSSSCG00000026590 / ENSG00000235173
HGS / O14964 / ENSSSCG00000033082 / ENSG00000185359
HIBADH / P31937 / ENSSSCG00000016695 / ENSG00000106049
HIGD2A / Q9BW72 / ENSSSCG00000029179 / ENSG00000146066
HIKESHI / Q53FT3 / ENSSSCG00000021210 / ENSG00000149196
HINT1 / P49773 / ENSSSCG00000014264 / ENSG00000169567
HINT3 / Q9NQE9 / ENSSSCG00000004221 / ENSG00000111911
HIP1 / O00291 / ENSSSCG00000007700 / ENSG00000127946
HIP1R / O75146 / ENSSSCG00000009786 / ENSG00000130787
HK1 / P19367 / ENSSSCG00000010253 / ENSG00000156515
Homologs
Adipose.Tissue
Brain
Colon
Heart
Kidney
Liver
Lung
Pancreas
S.Intestine
Spleen
Organs
Species
Human
Pig
Bins
1
2
3
4
5

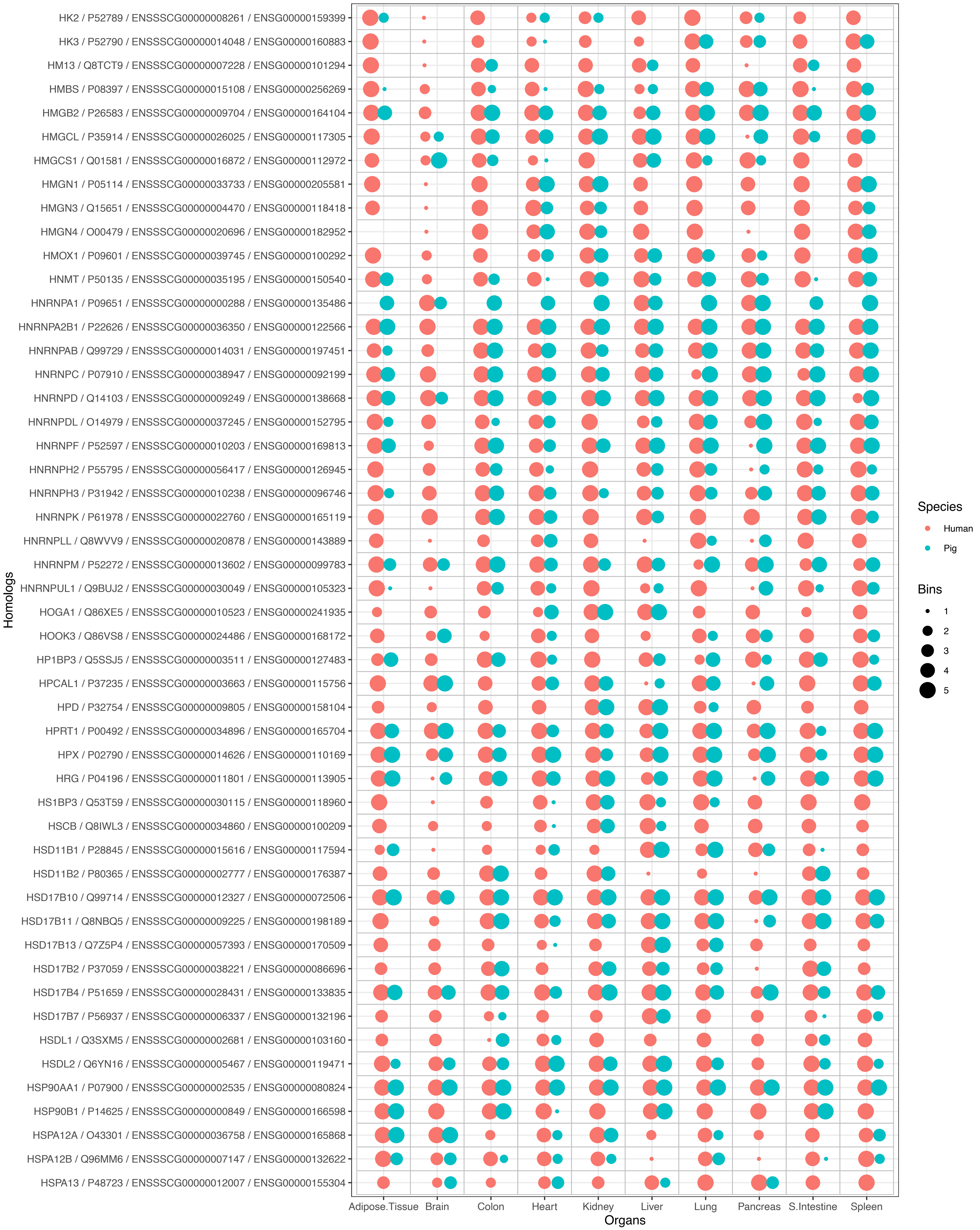
HK2 / P52789 / ENSSSCG00000008261 / ENSG00000159399
HK3 / P52790 / ENSSSCG00000014048 / ENSG00000160883
HM13 / Q8TCT9 / ENSSSCG00000007228 / ENSG00000101294
HMBS / P08397 / ENSSSCG00000015108 / ENSG00000256269
HMGB2 / P26583 / ENSSSCG00000009704 / ENSG00000164104
HMGCL / P35914 / ENSSSCG00000026025 / ENSG00000117305
HMGCS1 / Q01581 / ENSSSCG00000016872 / ENSG00000112972
HMGN1 / P05114 / ENSSSCG00000033733 / ENSG00000205581
HMGN3 / Q15651 / ENSSSCG00000004470 / ENSG00000118418
HMGN4 / O00479 / ENSSSCG00000020696 / ENSG00000182952
HMOX1 / P09601 / ENSSSCG00000039745 / ENSG00000100292
HNMT / P50135 / ENSSSCG00000035195 / ENSG00000150540
HNRNPA1 / P09651 / ENSSSCG00000000288 / ENSG00000135486
HNRNPA2B1 / P22626 / ENSSSCG00000036350 / ENSG00000122566
HNRNPAB / Q99729 / ENSSSCG00000014031 / ENSG00000197451
HNRNPC / P07910 / ENSSSCG00000038947 / ENSG00000092199
HNRNPD / Q14103 / ENSSSCG00000009249 / ENSG00000138668
HNRNPDL / O14979 / ENSSSCG00000037245 / ENSG00000152795
HNRNPF / P52597 / ENSSSCG00000010203 / ENSG00000169813
HNRNPH2 / P55795 / ENSSSCG00000056417 / ENSG00000126945
HNRNPH3 / P31942 / ENSSSCG00000010238 / ENSG00000096746
HNRNPK / P61978 / ENSSSCG00000022760 / ENSG00000165119
HNRNPLL / Q8WVV9 / ENSSSCG00000020878 / ENSG00000143889
HNRNPM / P52272 / ENSSSCG00000013602 / ENSG00000099783
HNRNPUL1 / Q9BUJ2 / ENSSSCG00000030049 / ENSG00000105323
HOGA1 / Q86XE5 / ENSSSCG00000010523 / ENSG00000241935
HOOK3 / Q86VS8 / ENSSSCG00000024486 / ENSG00000168172
HP1BP3 / Q5SSJ5 / ENSSSCG00000003511 / ENSG00000127483
HPCAL1 / P37235 / ENSSSCG00000003663 / ENSG00000115756
HPD / P32754 / ENSSSCG00000009805 / ENSG00000158104
HPRT1 / P00492 / ENSSSCG00000034896 / ENSG00000165704
HPX / P02790 / ENSSSCG00000014626 / ENSG00000110169
HRG / P04196 / ENSSSCG00000011801 / ENSG00000113905
HS1BP3 / Q53T59 / ENSSSCG00000030115 / ENSG00000118960
HSCB / Q8IWL3 / ENSSSCG00000034860 / ENSG00000100209
HSD11B1 / P28845 / ENSSSCG00000015616 / ENSG00000117594
HSD11B2 / P80365 / ENSSSCG00000002777 / ENSG00000176387
HSD17B10 / Q99714 / ENSSSCG00000012327 / ENSG00000072506
HSD17B11 / Q8NBQ5 / ENSSSCG00000009225 / ENSG00000198189
HSD17B13 / Q7Z5P4 / ENSSSCG00000057393 / ENSG00000170509
HSD17B2 / P37059 / ENSSSCG00000038221 / ENSG00000086696
HSD17B4 / P51659 / ENSSSCG00000028431 / ENSG00000133835
HSD17B7 / P56937 / ENSSSCG00000006337 / ENSG00000132196
HSDL1 / Q3SXM5 / ENSSSCG00000002681 / ENSG00000103160
HSDL2 / Q6YN16 / ENSSSCG00000005467 / ENSG00000119471
HSP90AA1 / P07900 / ENSSSCG00000002535 / ENSG00000080824
HSP90B1 / P14625 / ENSSSCG00000000849 / ENSG00000166598
HSPA12A / O43301 / ENSSSCG00000036758 / ENSG00000165868
HSPA12B / Q96MM6 / ENSSSCG00000007147 / ENSG00000132622
HSPA13 / P48723 / ENSSSCG00000012007 / ENSG00000155304
Homologs
Adipose.Tissue
Brain
Colon
Heart
Kidney
Liver
Lung
Pancreas
S.Intestine
Spleen
Organs
Species
Human
Pig
Bins
1
2
3
4
5

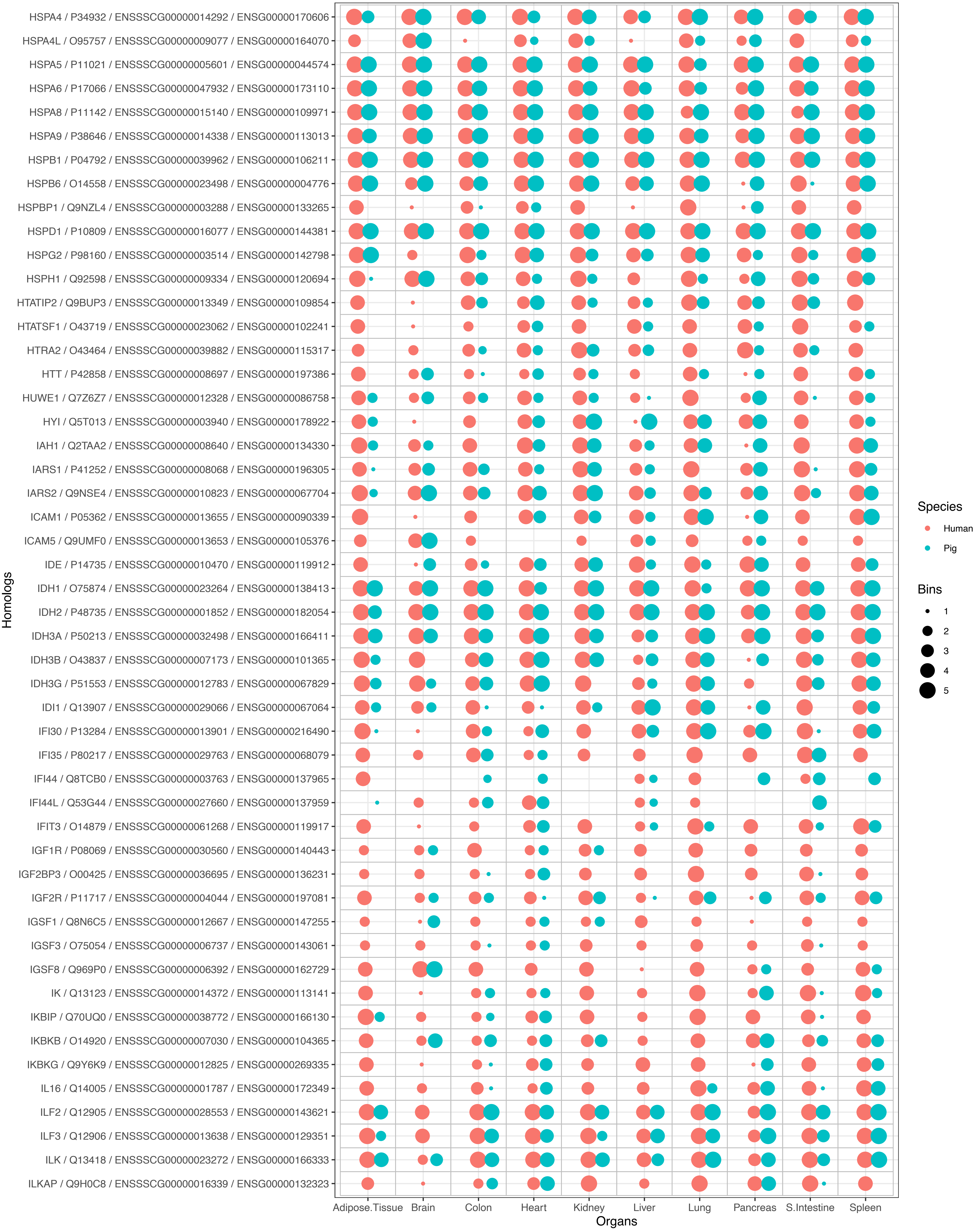

HSPA4 / P34932 / ENSSSCG00000014292 / ENSG00000170606
HSPA4L / O95757 / ENSSSCG00000009077 / ENSG00000164070
HSPA5 / P11021 / ENSSSCG00000005601 / ENSG00000044574
HSPA6 / P17066 / ENSSSCG00000047932 / ENSG00000173110
HSPA8 / P11142 / ENSSSCG00000015140 / ENSG00000109971
HSPA9 / P38646 / ENSSSCG00000014338 / ENSG00000113013
HSPB1 / P04792 / ENSSSCG00000039962 / ENSG00000106211
HSPB6 / O14558 / ENSSSCG00000023498 / ENSG00000004776
HSPBP1 / Q9NZL4 / ENSSSCG00000003288 / ENSG00000133265
HSPD1 / P10809 / ENSSSCG00000016077 / ENSG00000144381
HSPG2 / P98160 / ENSSSCG00000003514 / ENSG00000142798
HSPH1 / Q92598 / ENSSSCG00000009334 / ENSG00000120694
HTATIP2 / Q9BUP3 / ENSSSCG00000013349 / ENSG00000109854
HTATSF1 / O43719 / ENSSSCG00000023062 / ENSG00000102241
HTRA2 / O43464 / ENSSSCG00000039882 / ENSG00000115317
HTT / P42858 / ENSSSCG00000008697 / ENSG00000197386
HUWE1 / Q7Z6Z7 / ENSSSCG00000012328 / ENSG00000086758
HYI / Q5T013 / ENSSSCG00000003940 / ENSG00000178922
IAH1 / Q2TAA2 / ENSSSCG00000008640 / ENSG00000134330
IARS1 / P41252 / ENSSSCG00000008068 / ENSG00000196305
IARS2 / Q9NSE4 / ENSSSCG00000010823 / ENSG00000067704
ICAM1 / P05362 / ENSSSCG00000013655 / ENSG00000090339
ICAM5 / Q9UMF0 / ENSSSCG00000013653 / ENSG00000105376
IDE / P14735 / ENSSSCG00000010470 / ENSG00000119912
IDH1 / O75874 / ENSSSCG00000023264 / ENSG00000138413
IDH2 / P48735 / ENSSSCG00000001852 / ENSG00000182054
IDH3A / P50213 / ENSSSCG00000032498 / ENSG00000166411
IDH3B / O43837 / ENSSSCG00000007173 / ENSG00000101365
IDH3G / P51553 / ENSSSCG00000012783 / ENSG00000067829
IDI1 / Q13907 / ENSSSCG00000029066 / ENSG00000067064
IFI30 / P13284 / ENSSSCG00000013901 / ENSG00000216490
IFI35 / P80217 / ENSSSCG00000029763 / ENSG00000068079
IFI44 / Q8TCB0 / ENSSSCG00000003763 / ENSG00000137965
IFI44L / Q53G44 / ENSSSCG00000027660 / ENSG00000137959
IFIT3 / O14879 / ENSSSCG00000061268 / ENSG00000119917
IGF1R / P08069 / ENSSSCG00000030560 / ENSG00000140443
IGF2BP3 / O00425 / ENSSSCG00000036695 / ENSG00000136231
IGF2R / P11717 / ENSSSCG00000004044 / ENSG00000197081
IGSF1 / Q8N6C5 / ENSSSCG00000012667 / ENSG00000147255
IGSF3 / O75054 / ENSSSCG00000006737 / ENSG00000143061
IGSF8 / Q969P0 / ENSSSCG00000006392 / ENSG00000162729
IK / Q13123 / ENSSSCG00000014372 / ENSG00000113141
IKBIP / Q70UQ0 / ENSSSCG00000038772 / ENSG00000166130
IKBKB / O14920 / ENSSSCG00000007030 / ENSG00000104365
IKBKG / Q9Y6K9 / ENSSSCG00000012825 / ENSG00000269335
IL16 / Q14005 / ENSSSCG00000001787 / ENSG00000172349
ILF2 / Q12905 / ENSSSCG00000028553 / ENSG00000143621
ILF3 / Q12906 / ENSSSCG00000013638 / ENSG00000129351
ILK / Q13418 / ENSSSCG00000023272 / ENSG00000166333
ILKAP / Q9H0C8 / ENSSSCG00000016339 / ENSG00000132323
Homologs
Adipose.Tissue
Brain
Colon
Heart
Kidney
Liver
Lung
Pancreas
S.Intestine
Spleen
Organs
Species
Human
Pig
Bins
1
2
3
4
5

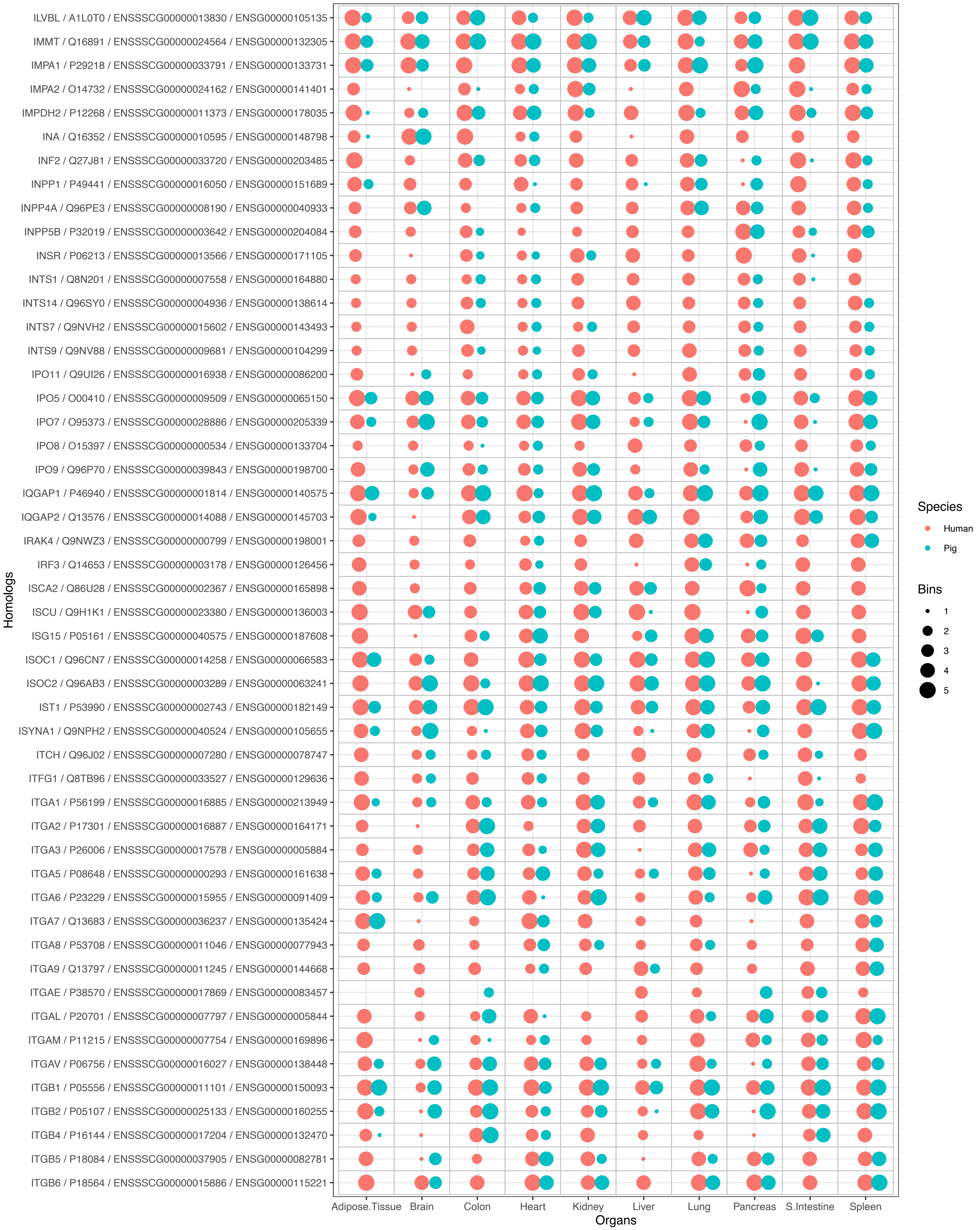

ILVBL / A1L0T0 / ENSSSCG00000013830 / ENSG00000105135
IMMT / Q16891 / ENSSSCG00000024564 / ENSG00000132305
IMPA1 / P29218 / ENSSSCG00000033791 / ENSG00000133731
IMPA2 / O14732 / ENSSSCG00000024162 / ENSG00000141401
IMPDH2 / P12268 / ENSSSCG00000011373 / ENSG00000178035
INA / Q16352 / ENSSSCG00000010595 / ENSG00000148798
INF2 / Q27J81 / ENSSSCG00000033720 / ENSG00000203485
INPP1 / P49441 / ENSSSCG00000016050 / ENSG00000151689
INPP4A / Q96PE3 / ENSSSCG00000008190 / ENSG00000040933
INPP5B / P32019 / ENSSSCG00000003642 / ENSG00000204084
INSR / P06213 / ENSSSCG00000013566 / ENSG00000171105
INTS1 / Q8N201 / ENSSSCG00000007558 / ENSG00000164880
INTS14 / Q96SY0 / ENSSSCG00000004936 / ENSG00000138614
INTS7 / Q9NVH2 / ENSSSCG00000015602 / ENSG00000143493
INTS9 / Q9NV88 / ENSSSCG00000009681 / ENSG00000104299
IPO11 / Q9UI26 / ENSSSCG00000016938 / ENSG00000086200
IPO5 / O00410 / ENSSSCG00000009509 / ENSG00000065150
IPO7 / O95373 / ENSSSCG00000028886 / ENSG00000205339
IPO8 / O15397 / ENSSSCG00000000534 / ENSG00000133704
IPO9 / Q96P70 / ENSSSCG00000039843 / ENSG00000198700
IQGAP1 / P46940 / ENSSSCG00000001814 / ENSG00000140575
IQGAP2 / Q13576 / ENSSSCG00000014088 / ENSG00000145703
IRAK4 / Q9NWZ3 / ENSSSCG00000000799 / ENSG00000198001
IRF3 / Q14653 / ENSSSCG00000003178 / ENSG00000126456
ISCA2 / Q86U28 / ENSSSCG00000002367 / ENSG00000165898
ISCU / Q9H1K1 / ENSSSCG00000023380 / ENSG00000136003
ISG15 / P05161 / ENSSSCG00000040575 / ENSG00000187608
ISOC1 / Q96CN7 / ENSSSCG00000014258 / ENSG00000066583
ISOC2 / Q96AB3 / ENSSSCG00000003289 / ENSG00000063241
IST1 / P53990 / ENSSSCG00000002743 / ENSG00000182149
ISYNA1 / Q9NPH2 / ENSSSCG00000040524 / ENSG00000105655
ITCH / Q96J02 / ENSSSCG00000007280 / ENSG00000078747
ITFG1 / Q8TB96 / ENSSSCG00000033527 / ENSG00000129636
ITGA1 / P56199 / ENSSSCG00000016885 / ENSG00000213949
ITGA2 / P17301 / ENSSSCG00000016887 / ENSG00000164171
ITGA3 / P26006 / ENSSSCG00000017578 / ENSG00000005884
ITGA5 / P08648 / ENSSSCG00000000293 / ENSG00000161638
ITGA6 / P23229 / ENSSSCG00000015955 / ENSG00000091409
ITGA7 / Q13683 / ENSSSCG00000036237 / ENSG00000135424
ITGA8 / P53708 / ENSSSCG00000011046 / ENSG00000077943
ITGA9 / Q13797 / ENSSSCG00000011245 / ENSG00000144668
ITGAE / P38570 / ENSSSCG00000017869 / ENSG00000083457
ITGAL / P20701 / ENSSSCG00000007797 / ENSG00000005844
ITGAM / P11215 / ENSSSCG00000007754 / ENSG00000169896
ITGAV / P06756 / ENSSSCG00000016027 / ENSG00000138448
ITGB1 / P05556 / ENSSSCG00000011101 / ENSG00000150093
ITGB2 / P05107 / ENSSSCG00000025133 / ENSG00000160255
ITGB4 / P16144 / ENSSSCG00000017204 / ENSG00000132470
ITGB5 / P18084 / ENSSSCG00000037905 / ENSG00000082781
ITGB6 / P18564 / ENSSSCG00000015886 / ENSG00000115221
Homologs
Adipose.Tissue
Brain
Colon
Heart
Kidney
Liver
Lung
Pancreas
S.Intestine
Spleen
Organs
Species
Human
Pig
Bins
1
2
3
4
5

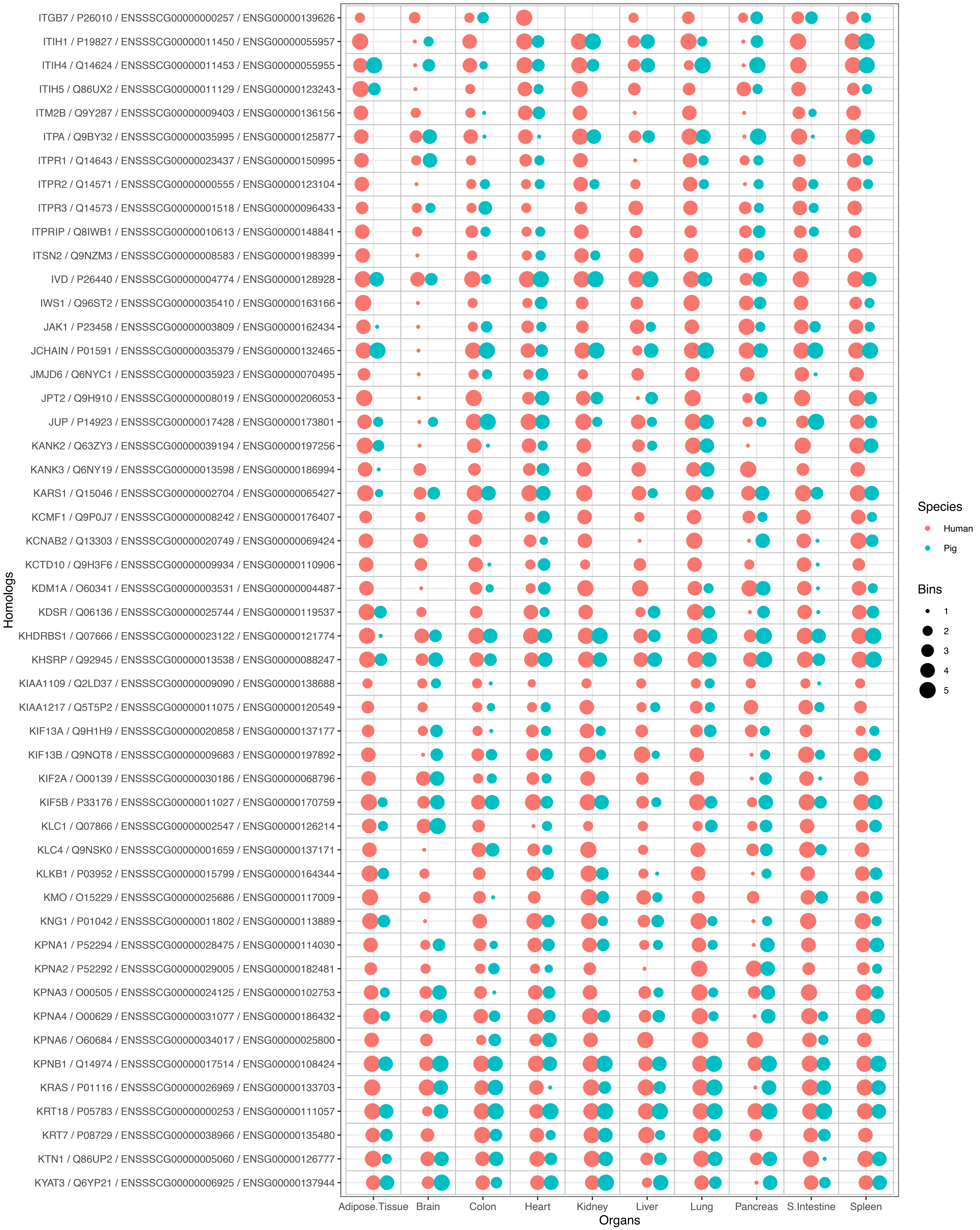
ITGB7 / P26010 / ENSSSCG00000000257 / ENSG00000139626
ITIH1 / P19827 / ENSSSCG00000011450 / ENSG00000055957
ITIH4 / Q14624 / ENSSSCG00000011453 / ENSG00000055955
ITIH5 / Q86UX2 / ENSSSCG00000011129 / ENSG00000123243
ITM2B / Q9Y287 / ENSSSCG00000009403 / ENSG00000136156
ITPA / Q9BY32 / ENSSSCG00000035995 / ENSG00000125877
ITPR1 / Q14643 / ENSSSCG00000023437 / ENSG00000150995
ITPR2 / Q14571 / ENSSSCG00000000555 / ENSG00000123104
ITPR3 / Q14573 / ENSSSCG00000001518 / ENSG00000096433
ITPRIP / Q8IWB1 / ENSSSCG00000010613 / ENSG00000148841
ITSN2 / Q9NZM3 / ENSSSCG00000008583 / ENSG00000198399
IVD / P26440 / ENSSSCG00000004774 / ENSG00000128928
IWS1 / Q96ST2 / ENSSSCG00000035410 / ENSG00000163166
JAK1 / P23458 / ENSSSCG00000003809 / ENSG00000162434
JCHAIN / P01591 / ENSSSCG00000035379 / ENSG00000132465
JMJD6 / Q6NYC1 / ENSSSCG00000035923 / ENSG00000070495
JPT2 / Q9H910 / ENSSSCG00000008019 / ENSG00000206053
JUP / P14923 / ENSSSCG00000017428 / ENSG00000173801
KANK2 / Q63ZY3 / ENSSSCG00000039194 / ENSG00000197256
KANK3 / Q6NY19 / ENSSSCG00000013598 / ENSG00000186994
KARS1 / Q15046 / ENSSSCG00000002704 / ENSG00000065427
KCMF1 / Q9P0J7 / ENSSSCG00000008242 / ENSG00000176407
KCNAB2 / Q13303 / ENSSSCG00000020749 / ENSG00000069424
KCTD10 / Q9H3F6 / ENSSSCG00000009934 / ENSG00000110906
KDM1A / O60341 / ENSSSCG00000003531 / ENSG00000004487
KDSR / Q06136 / ENSSSCG00000025744 / ENSG00000119537
KHDRBS1 / Q07666 / ENSSSCG00000023122 / ENSG00000121774
KHSRP / Q92945 / ENSSSCG00000013538 / ENSG00000088247
KIAA1109 / Q2LD37 / ENSSSCG00000009090 / ENSG00000138688
KIAA1217 / Q5T5P2 / ENSSSCG00000011075 / ENSG00000120549
KIF13A / Q9H1H9 / ENSSSCG00000020858 / ENSG00000137177
KIF13B / Q9NQT8 / ENSSSCG00000009683 / ENSG00000197892
KIF2A / O00139 / ENSSSCG00000030186 / ENSG00000068796
KIF5B / P33176 / ENSSSCG00000011027 / ENSG00000170759
KLC1 / Q07866 / ENSSSCG00000002547 / ENSG00000126214
KLC4 / Q9NSK0 / ENSSSCG00000001659 / ENSG00000137171
KLKB1 / P03952 / ENSSSCG00000015799 / ENSG00000164344
KMO / O15229 / ENSSSCG00000025686 / ENSG00000117009
KNG1 / P01042 / ENSSSCG00000011802 / ENSG00000113889
KPNA1 / P52294 / ENSSSCG00000028475 / ENSG00000114030
KPNA2 / P52292 / ENSSSCG00000029005 / ENSG00000182481
KPNA3 / O00505 / ENSSSCG00000024125 / ENSG00000102753
KPNA4 / O00629 / ENSSSCG00000031077 / ENSG00000186432
KPNA6 / O60684 / ENSSSCG00000034017 / ENSG00000025800
KPNB1 / Q14974 / ENSSSCG00000017514 / ENSG00000108424
KRAS / P01116 / ENSSSCG00000026969 / ENSG00000133703
KRT18 / P05783 / ENSSSCG00000000253 / ENSG00000111057
KRT7 / P08729 / ENSSSCG00000038966 / ENSG00000135480
KTN1 / Q86UP2 / ENSSSCG00000005060 / ENSG00000126777
KYAT3 / Q6YP21 / ENSSSCG00000006925 / ENSG00000137944
Homologs
Adipose.Tissue
Brain
Colon
Heart
Kidney
Liver
Lung
Pancreas
S.Intestine
Spleen
Organs
Species
Human
Pig
Bins
1
2
3
4
5

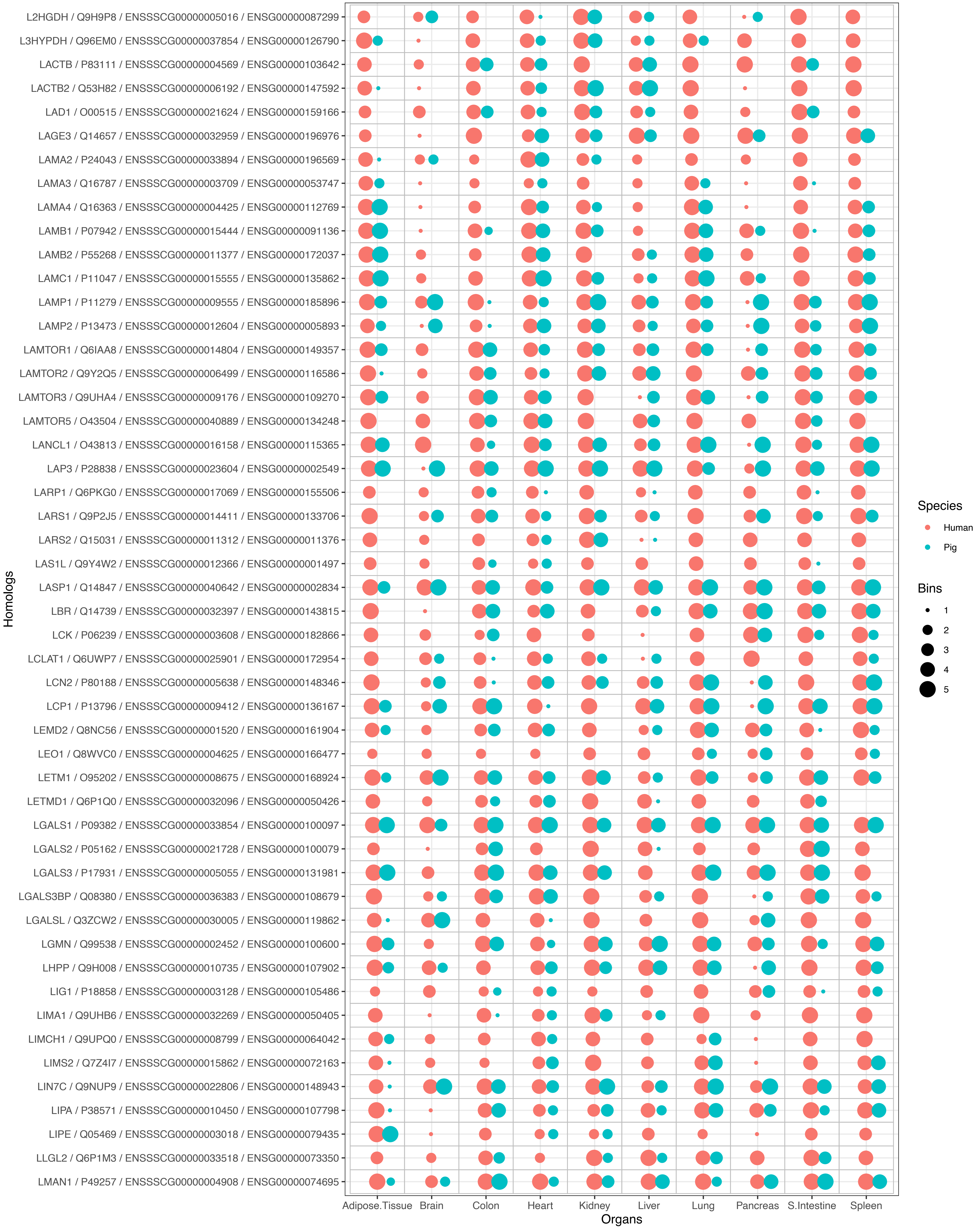
L2HGDH / Q9H9P8 / ENSSSCG00000005016 / ENSG00000087299
L3HYPDH / Q96EM0 / ENSSSCG00000037854 / ENSG00000126790
LACTB / P83111 / ENSSSCG00000004569 / ENSG00000103642
LACTB2 / Q53H82 / ENSSSCG00000006192 / ENSG00000147592
LAD1 / O00515 / ENSSSCG00000021624 / ENSG00000159166
LAGE3 / Q14657 / ENSSSCG00000032959 / ENSG00000196976
LAMA2 / P24043 / ENSSSCG00000033894 / ENSG00000196569
LAMA3 / Q16787 / ENSSSCG00000003709 / ENSG00000053747
LAMA4 / Q16363 / ENSSSCG00000004425 / ENSG00000112769
LAMB1 / P07942 / ENSSSCG00000015444 / ENSG00000091136
LAMB2 / P55268 / ENSSSCG00000011377 / ENSG00000172037
LAMC1 / P11047 / ENSSSCG00000015555 / ENSG00000135862
LAMP1 / P11279 / ENSSSCG00000009555 / ENSG00000185896
LAMP2 / P13473 / ENSSSCG00000012604 / ENSG00000005893
LAMTOR1 / Q6IAA8 / ENSSSCG00000014804 / ENSG00000149357
LAMTOR2 / Q9Y2Q5 / ENSSSCG00000006499 / ENSG00000116586
LAMTOR3 / Q9UHA4 / ENSSSCG00000009176 / ENSG00000109270
LAMTOR5 / O43504 / ENSSSCG00000040889 / ENSG00000134248
LANCL1 / O43813 / ENSSSCG00000016158 / ENSG00000115365
LAP3 / P28838 / ENSSSCG00000023604 / ENSG00000002549
LARP1 / Q6PKG0 / ENSSSCG00000017069 / ENSG00000155506
LARS1 / Q9P2J5 / ENSSSCG00000014411 / ENSG00000133706
LARS2 / Q15031 / ENSSSCG00000011312 / ENSG00000011376
LAS1L / Q9Y4W2 / ENSSSCG00000012366 / ENSG00000001497
LASP1 / Q14847 / ENSSSCG00000040642 / ENSG00000002834
LBR / Q14739 / ENSSSCG00000032397 / ENSG00000143815
LCK / P06239 / ENSSSCG00000003608 / ENSG00000182866
LCLAT1 / Q6UWP7 / ENSSSCG00000025901 / ENSG00000172954
LCN2 / P80188 / ENSSSCG00000005638 / ENSG00000148346
LCP1 / P13796 / ENSSSCG00000009412 / ENSG00000136167
LEMD2 / Q8NC56 / ENSSSCG00000001520 / ENSG00000161904
LEO1 / Q8WVC0 / ENSSSCG00000004625 / ENSG00000166477
LETM1 / O95202 / ENSSSCG00000008675 / ENSG00000168924
LETMD1 / Q6P1Q0 / ENSSSCG00000032096 / ENSG00000050426
LGALS1 / P09382 / ENSSSCG00000033854 / ENSG00000100097
LGALS2 / P05162 / ENSSSCG00000021728 / ENSG00000100079
LGALS3 / P17931 / ENSSSCG00000005055 / ENSG00000131981
LGALS3BP / Q08380 / ENSSSCG00000036383 / ENSG00000108679
LGALSL / Q3ZCW2 / ENSSSCG00000030005 / ENSG00000119862
LGMN / Q99538 / ENSSSCG00000002452 / ENSG00000100600
LHPP / Q9H008 / ENSSSCG00000010735 / ENSG00000107902
LIG1 / P18858 / ENSSSCG00000003128 / ENSG00000105486
LIMA1 / Q9UHB6 / ENSSSCG00000032269 / ENSG00000050405
LIMCH1 / Q9UPQ0 / ENSSSCG00000008799 / ENSG00000064042
LIMS2 / Q7Z4I7 / ENSSSCG00000015862 / ENSG00000072163
LIN7C / Q9NUP9 / ENSSSCG00000022806 / ENSG00000148943
LIPA / P38571 / ENSSSCG00000010450 / ENSG00000107798
LIPE / Q05469 / ENSSSCG00000003018 / ENSG00000079435
LLGL2 / Q6P1M3 / ENSSSCG00000033518 / ENSG00000073350
LMAN1 / P49257 / ENSSSCG00000004908 / ENSG00000074695
Homologs
Adipose.Tissue
Brain
Colon
Heart
Kidney
Liver
Lung
Pancreas
S.Intestine
Spleen
Organs
Species
Human
Pig
Bins
1
2
3
4
5

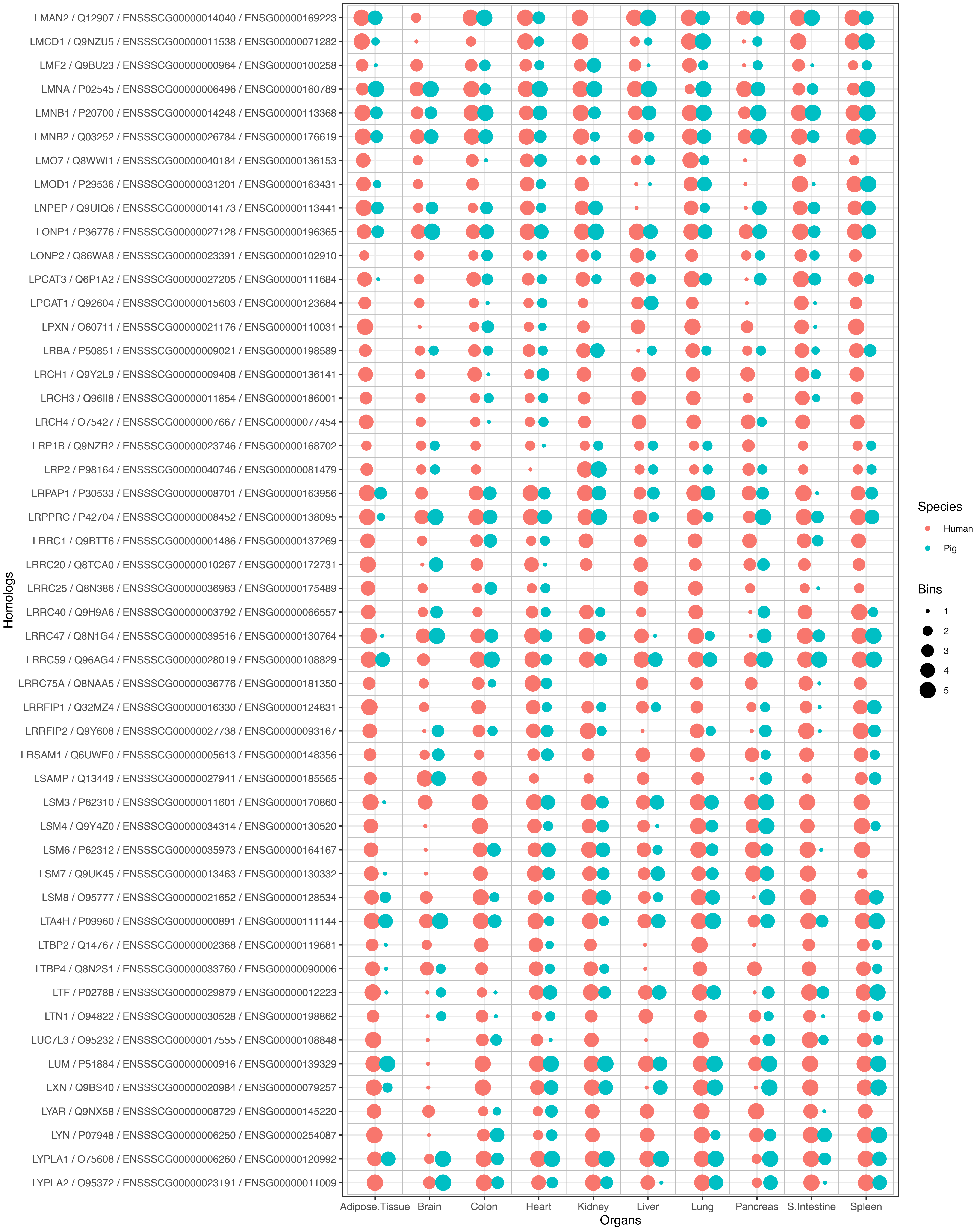

LMAN2 / Q12907 / ENSSSCG00000014040 / ENSG00000169223
LMCD1 / Q9NZU5 / ENSSSCG00000011538 / ENSG00000071282
LMF2 / Q9BU23 / ENSSSCG00000000964 / ENSG00000100258
LMNA / P02545 / ENSSSCG00000006496 / ENSG00000160789
LMNB1 / P20700 / ENSSSCG00000014248 / ENSG00000113368
LMNB2 / Q03252 / ENSSSCG00000026784 / ENSG00000176619
LMO7 / Q8WWI1 / ENSSSCG00000040184 / ENSG00000136153
LMOD1 / P29536 / ENSSSCG00000031201 / ENSG00000163431
LNPEP / Q9UIQ6 / ENSSSCG00000014173 / ENSG00000113441
LONP1 / P36776 / ENSSSCG00000027128 / ENSG00000196365
LONP2 / Q86WA8 / ENSSSCG00000023391 / ENSG00000102910
LPCAT3 / Q6P1A2 / ENSSSCG00000027205 / ENSG00000111684
LPGAT1 / Q92604 / ENSSSCG00000015603 / ENSG00000123684
LPXN / O60711 / ENSSSCG00000021176 / ENSG00000110031
LRBA / P50851 / ENSSSCG00000009021 / ENSG00000198589
LRCH1 / Q9Y2L9 / ENSSSCG00000009408 / ENSG00000136141
LRCH3 / Q96II8 / ENSSSCG00000011854 / ENSG00000186001
LRCH4 / O75427 / ENSSSCG00000007667 / ENSG00000077454
LRP1B / Q9NZR2 / ENSSSCG00000023746 / ENSG00000168702
LRP2 / P98164 / ENSSSCG00000040746 / ENSG00000081479
LRPAP1 / P30533 / ENSSSCG00000008701 / ENSG00000163956
LRPPRC / P42704 / ENSSSCG00000008452 / ENSG00000138095
LRRC1 / Q9BTT6 / ENSSSCG00000001486 / ENSG00000137269
LRRC20 / Q8TCA0 / ENSSSCG00000010267 / ENSG00000172731
LRRC25 / Q8N386 / ENSSSCG00000036963 / ENSG00000175489
LRRC40 / Q9H9A6 / ENSSSCG00000003792 / ENSG00000066557
LRRC47 / Q8N1G4 / ENSSSCG00000039516 / ENSG00000130764
LRRC59 / Q96AG4 / ENSSSCG00000028019 / ENSG00000108829
LRRC75A / Q8NAA5 / ENSSSCG00000036776 / ENSG00000181350
LRRFIP1 / Q32MZ4 / ENSSSCG00000016330 / ENSG00000124831
LRRFIP2 / Q9Y608 / ENSSSCG00000027738 / ENSG00000093167
LRSAM1 / Q6UWE0 / ENSSSCG00000005613 / ENSG00000148356
LSAMP / Q13449 / ENSSSCG00000027941 / ENSG00000185565
LSM3 / P62310 / ENSSSCG00000011601 / ENSG00000170860
LSM4 / Q9Y4Z0 / ENSSSCG00000034314 / ENSG00000130520
LSM6 / P62312 / ENSSSCG00000035973 / ENSG00000164167
LSM7 / Q9UK45 / ENSSSCG00000013463 / ENSG00000130332
LSM8 / O95777 / ENSSSCG00000021652 / ENSG00000128534
LTA4H / P09960 / ENSSSCG00000000891 / ENSG00000111144
LTBP2 / Q14767 / ENSSSCG00000002368 / ENSG00000119681
LTBP4 / Q8N2S1 / ENSSSCG00000033760 / ENSG00000090006
LTF / P02788 / ENSSSCG00000029879 / ENSG00000012223
LTN1 / O94822 / ENSSSCG00000030528 / ENSG00000198862
LUC7L3 / O95232 / ENSSSCG00000017555 / ENSG00000108848
LUM / P51884 / ENSSSCG00000000916 / ENSG00000139329
LXN / Q9BS40 / ENSSSCG00000020984 / ENSG00000079257
LYAR / Q9NX58 / ENSSSCG00000008729 / ENSG00000145220
LYN / P07948 / ENSSSCG00000006250 / ENSG00000254087
LYPLA1 / O75608 / ENSSSCG00000006260 / ENSG00000120992
LYPLA2 / O95372 / ENSSSCG00000023191 / ENSG00000011009
Homologs
Adipose.Tissue
Brain
Colon
Heart
Kidney
Liver
Lung
Pancreas
S.Intestine
Spleen
Organs
Species
Human
Pig
Bins
1
2
3
4
5

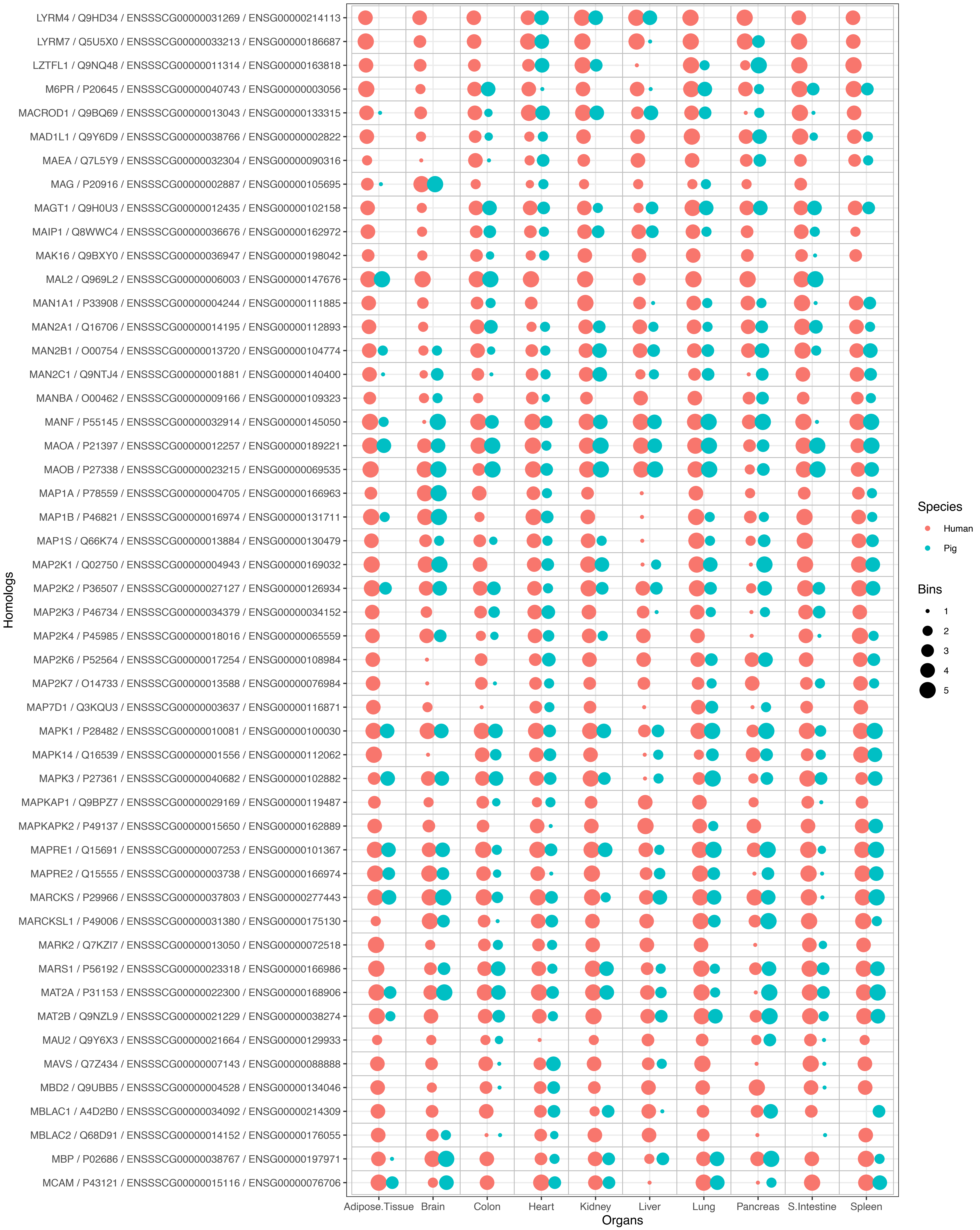

LYRM4 / Q9HD34 / ENSSSCG00000031269 / ENSG00000214113
LYRM7 / Q5U5X0 / ENSSSCG00000033213 / ENSG00000186687
LZTFL1 / Q9NQ48 / ENSSSCG00000011314 / ENSG00000163818
M6PR / P20645 / ENSSSCG00000040743 / ENSG00000003056
MACROD1 / Q9BQ69 / ENSSSCG00000013043 / ENSG00000133315
MAD1L1 / Q9Y6D9 / ENSSSCG00000038766 / ENSG00000002822
MAEA / Q7L5Y9 / ENSSSCG00000032304 / ENSG00000090316
MAG / P20916 / ENSSSCG00000002887 / ENSG00000105695
MAGT1 / Q9H0U3 / ENSSSCG00000012435 / ENSG00000102158
MAIP1 / Q8WWC4 / ENSSSCG00000036676 / ENSG00000162972
MAK16 / Q9BXY0 / ENSSSCG00000036947 / ENSG00000198042
MAL2 / Q969L2 / ENSSSCG00000006003 / ENSG00000147676
MAN1A1 / P33908 / ENSSSCG00000004244 / ENSG00000111885
MAN2A1 / Q16706 / ENSSSCG00000014195 / ENSG00000112893
MAN2B1 / O00754 / ENSSSCG00000013720 / ENSG00000104774
MAN2C1 / Q9NTJ4 / ENSSSCG00000001881 / ENSG00000140400
MANBA / O00462 / ENSSSCG00000009166 / ENSG00000109323
MANF / P55145 / ENSSSCG00000032914 / ENSG00000145050
MAOA / P21397 / ENSSSCG00000012257 / ENSG00000189221
MAOB / P27338 / ENSSSCG00000023215 / ENSG00000069535
MAP1A / P78559 / ENSSSCG00000004705 / ENSG00000166963
MAP1B / P46821 / ENSSSCG00000016974 / ENSG00000131711
MAP1S / Q66K74 / ENSSSCG00000013884 / ENSG00000130479
MAP2K1 / Q02750 / ENSSSCG00000004943 / ENSG00000169032
MAP2K2 / P36507 / ENSSSCG00000027127 / ENSG00000126934
MAP2K3 / P46734 / ENSSSCG00000034379 / ENSG00000034152
MAP2K4 / P45985 / ENSSSCG00000018016 / ENSG00000065559
MAP2K6 / P52564 / ENSSSCG00000017254 / ENSG00000108984
MAP2K7 / O14733 / ENSSSCG00000013588 / ENSG00000076984
MAP7D1 / Q3KQU3 / ENSSSCG00000003637 / ENSG00000116871
MAPK1 / P28482 / ENSSSCG00000010081 / ENSG00000100030
MAPK14 / Q16539 / ENSSSCG00000001556 / ENSG00000112062
MAPK3 / P27361 / ENSSSCG00000040682 / ENSG00000102882
MAPKAP1 / Q9BPZ7 / ENSSSCG00000029169 / ENSG00000119487
MAPKAPK2 / P49137 / ENSSSCG00000015650 / ENSG00000162889
MAPRE1 / Q15691 / ENSSSCG00000007253 / ENSG00000101367
MAPRE2 / Q15555 / ENSSSCG00000003738 / ENSG00000166974
MARCKS / P29966 / ENSSSCG00000037803 / ENSG00000277443
MARCKSL1 / P49006 / ENSSSCG00000031380 / ENSG00000175130
MARK2 / Q7KZI7 / ENSSSCG00000013050 / ENSG00000072518
MARS1 / P56192 / ENSSSCG00000023318 / ENSG00000166986
MAT2A / P31153 / ENSSSCG00000022300 / ENSG00000168906
MAT2B / Q9NZL9 / ENSSSCG00000021229 / ENSG00000038274
MAU2 / Q9Y6X3 / ENSSSCG00000021664 / ENSG00000129933
MAVS / Q7Z434 / ENSSSCG00000007143 / ENSG00000088888
MBD2 / Q9UBB5 / ENSSSCG00000004528 / ENSG00000134046
MBLAC1 / A4D2B0 / ENSSSCG00000034092 / ENSG00000214309
MBLAC2 / Q68D91 / ENSSSCG00000014152 / ENSG00000176055
MBP / P02686 / ENSSSCG00000038767 / ENSG00000197971
MCAM / P43121 / ENSSSCG00000015116 / ENSG00000076706
Homologs
Adipose.Tissue
Brain
Colon
Heart
Kidney
Liver
Lung
Pancreas
S.Intestine
Spleen
Organs
Species
Human
Pig
Bins
1
2
3
4
5

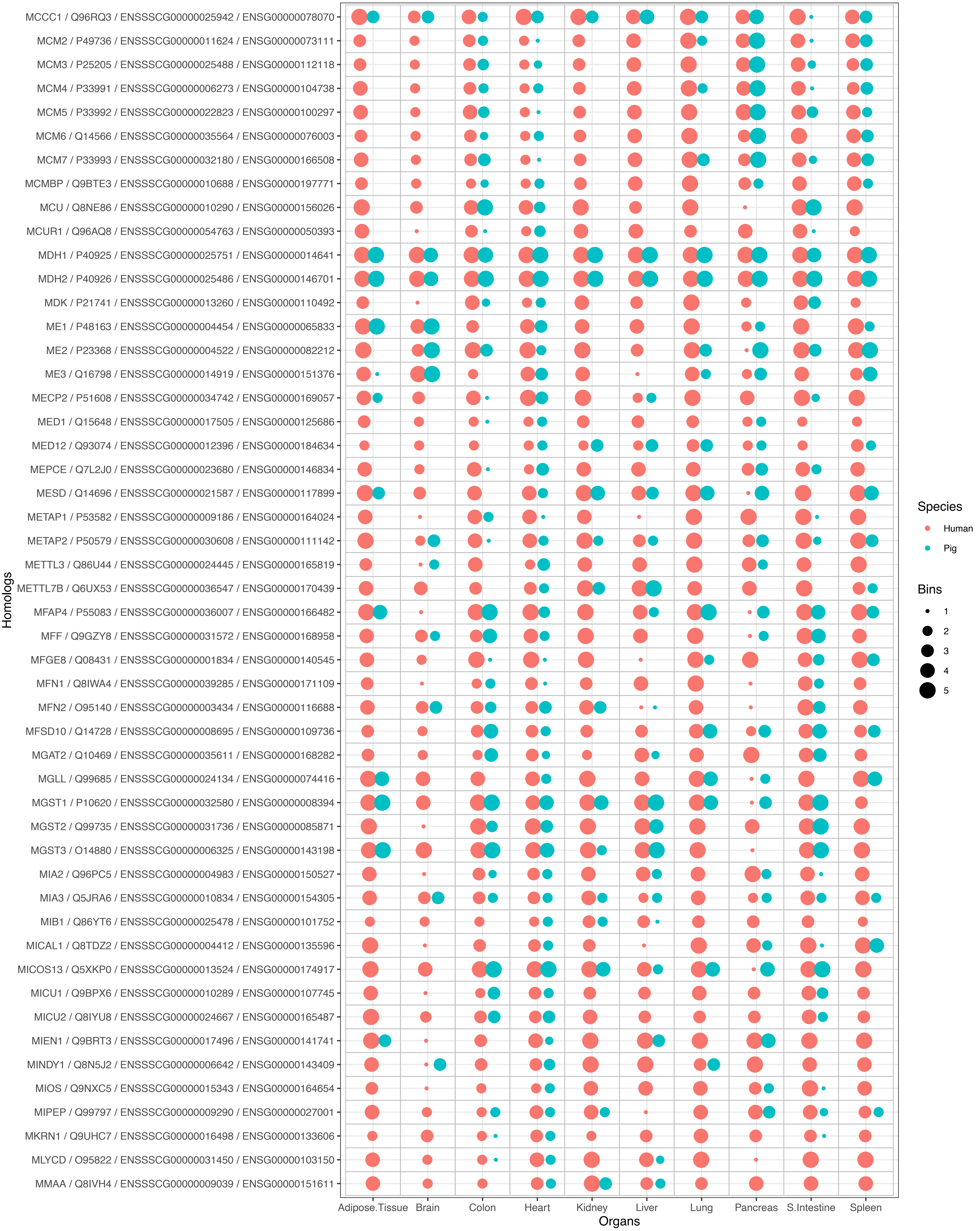
MCCC1 / Q96RQ3 / ENSSSCG00000025942 / ENSG00000078070
MCM2 / P49736 / ENSSSCG00000011624 / ENSG00000073111
MCM3 / P25205 / ENSSSCG00000025488 / ENSG00000112118
MCM4 / P33991 / ENSSSCG00000006273 / ENSG00000104738
MCM5 / P33992 / ENSSSCG00000022823 / ENSG00000100297
MCM6 / Q14566 / ENSSSCG00000035564 / ENSG00000076003
MCM7 / P33993 / ENSSSCG00000032180 / ENSG00000166508
MCMBP / Q9BTE3 / ENSSSCG00000010688 / ENSG00000197771
MCU / Q8NE86 / ENSSSCG00000010290 / ENSG00000156026
MCUR1 / Q96AQ8 / ENSSSCG00000054763 / ENSG00000050393
MDH1 / P40925 / ENSSSCG00000025751 / ENSG00000014641
MDH2 / P40926 / ENSSSCG00000025486 / ENSG00000146701
MDK / P21741 / ENSSSCG00000013260 / ENSG00000110492
ME1 / P48163 / ENSSSCG00000004454 / ENSG00000065833
ME2 / P23368 / ENSSSCG00000004522 / ENSG00000082212
ME3 / Q16798 / ENSSSCG00000014919 / ENSG00000151376
MECP2 / P51608 / ENSSSCG00000034742 / ENSG00000169057
MED1 / Q15648 / ENSSSCG00000017505 / ENSG00000125686
MED12 / Q93074 / ENSSSCG00000012396 / ENSG00000184634
MEPCE / Q7L2J0 / ENSSSCG00000023680 / ENSG00000146834
MESD / Q14696 / ENSSSCG00000021587 / ENSG00000117899
METAP1 / P53582 / ENSSSCG00000009186 / ENSG00000164024
METAP2 / P50579 / ENSSSCG00000030608 / ENSG00000111142
METTL3 / Q86U44 / ENSSSCG00000024445 / ENSG00000165819
METTL7B / Q6UX53 / ENSSSCG00000036547 / ENSG00000170439
MFAP4 / P55083 / ENSSSCG00000036007 / ENSG00000166482
MFF / Q9GZY8 / ENSSSCG00000031572 / ENSG00000168958
MFGE8 / Q08431 / ENSSSCG00000001834 / ENSG00000140545
MFN1 / Q8IWA4 / ENSSSCG00000039285 / ENSG00000171109
MFN2 / O95140 / ENSSSCG00000003434 / ENSG00000116688
MFSD10 / Q14728 / ENSSSCG00000008695 / ENSG00000109736
MGAT2 / Q10469 / ENSSSCG00000035611 / ENSG00000168282
MGLL / Q99685 / ENSSSCG00000024134 / ENSG00000074416
MGST1 / P10620 / ENSSSCG00000032580 / ENSG00000008394
MGST2 / Q99735 / ENSSSCG00000031736 / ENSG00000085871
MGST3 / O14880 / ENSSSCG00000006325 / ENSG00000143198
MIA2 / Q96PC5 / ENSSSCG00000004983 / ENSG00000150527
MIA3 / Q5JRA6 / ENSSSCG00000010834 / ENSG00000154305
MIB1 / Q86YT6 / ENSSSCG00000025478 / ENSG00000101752
MICAL1 / Q8TDZ2 / ENSSSCG00000004412 / ENSG00000135596
MICOS13 / Q5XKP0 / ENSSSCG00000013524 / ENSG00000174917
MICU1 / Q9BPX6 / ENSSSCG00000010289 / ENSG00000107745
MICU2 / Q8IYU8 / ENSSSCG00000024667 / ENSG00000165487
MIEN1 / Q9BRT3 / ENSSSCG00000017496 / ENSG00000141741
MINDY1 / Q8N5J2 / ENSSSCG00000006642 / ENSG00000143409
MIOS / Q9NXC5 / ENSSSCG00000015343 / ENSG00000164654
MIPEP / Q99797 / ENSSSCG00000009290 / ENSG00000027001
MKRN1 / Q9UHC7 / ENSSSCG00000016498 / ENSG00000133606
MLYCD / O95822 / ENSSSCG00000031450 / ENSG00000103150
MMAA / Q8IVH4 / ENSSSCG00000009039 / ENSG00000151611
Homologs
Adipose.Tissue
Brain
Colon
Heart
Kidney
Liver
Lung
Pancreas
S.Intestine
Spleen
Organs
Species
Human
Pig
Bins
1
2
3
4
5

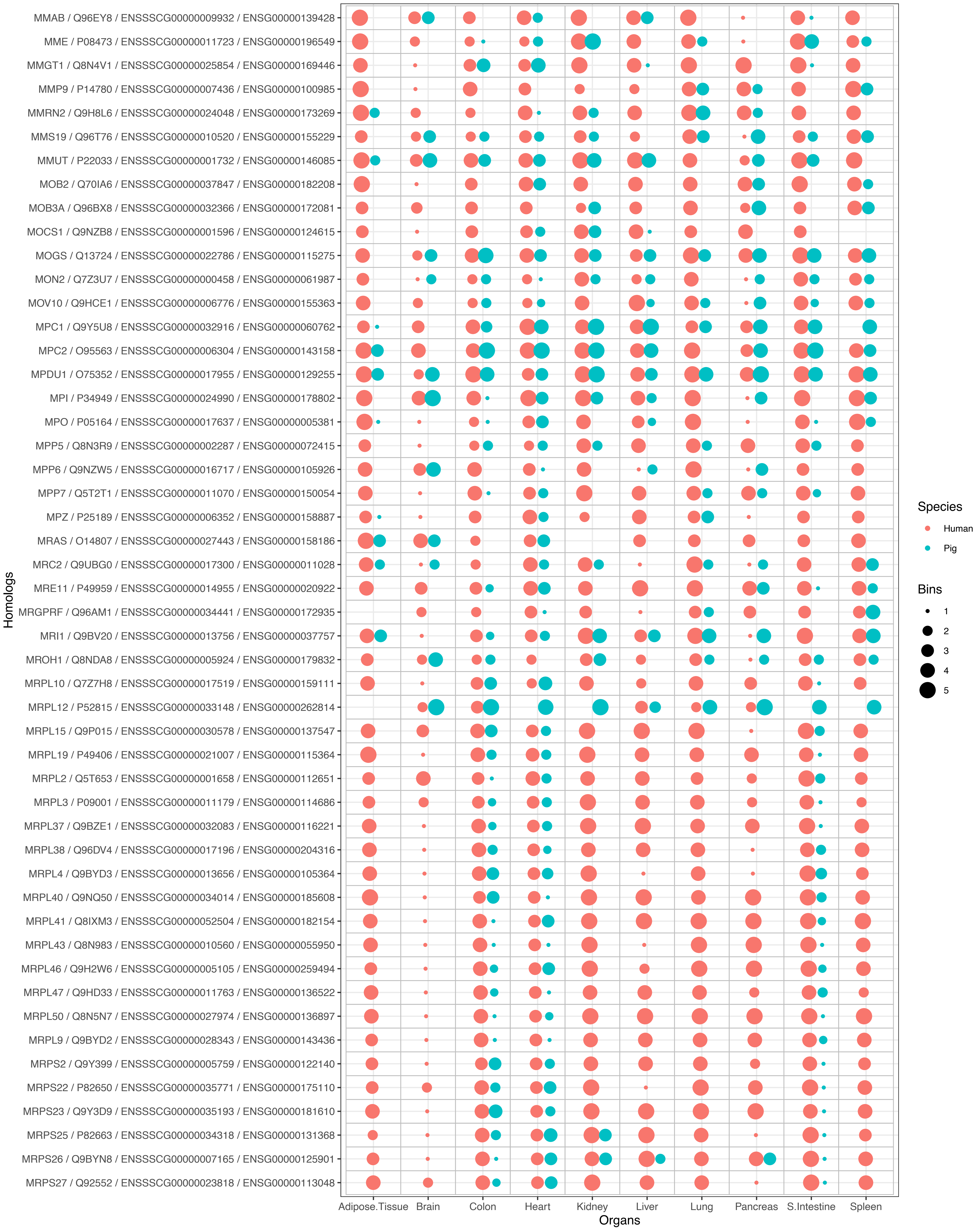
MMAB / Q96EY8 / ENSSSCG00000009932 / ENSG00000139428
MME / P08473 / ENSSSCG00000011723 / ENSG00000196549
MMGT1 / Q8N4V1 / ENSSSCG00000025854 / ENSG00000169446
MMP9 / P14780 / ENSSSCG00000007436 / ENSG00000100985
MMRN2 / Q9H8L6 / ENSSSCG00000024048 / ENSG00000173269
MMS19 / Q96T76 / ENSSSCG00000010520 / ENSG00000155229
MMUT / P22033 / ENSSSCG00000001732 / ENSG00000146085
MOB2 / Q70IA6 / ENSSSCG00000037847 / ENSG00000182208
MOB3A / Q96BX8 / ENSSSCG00000032366 / ENSG00000172081
MOCS1 / Q9NZB8 / ENSSSCG00000001596 / ENSG00000124615
MOGS / Q13724 / ENSSSCG00000022786 / ENSG00000115275
MON2 / Q7Z3U7 / ENSSSCG00000000458 / ENSG00000061987
MOV10 / Q9HCE1 / ENSSSCG00000006776 / ENSG00000155363
MPC1 / Q9Y5U8 / ENSSSCG00000032916 / ENSG00000060762
MPC2 / O95563 / ENSSSCG00000006304 / ENSG00000143158
MPDU1 / O75352 / ENSSSCG00000017955 / ENSG00000129255
MPI / P34949 / ENSSSCG00000024990 / ENSG00000178802
MPO / P05164 / ENSSSCG00000017637 / ENSG00000005381
MPP5 / Q8N3R9 / ENSSSCG00000002287 / ENSG00000072415
MPP6 / Q9NZW5 / ENSSSCG00000016717 / ENSG00000105926
MPP7 / Q5T2T1 / ENSSSCG00000011070 / ENSG00000150054
MPZ / P25189 / ENSSSCG00000006352 / ENSG00000158887
MRAS / O14807 / ENSSSCG00000027443 / ENSG00000158186
MRC2 / Q9UBG0 / ENSSSCG00000017300 / ENSG00000011028
MRE11 / P49959 / ENSSSCG00000014955 / ENSG00000020922
MRGPRF / Q96AM1 / ENSSSCG00000034441 / ENSG00000172935
MRI1 / Q9BV20 / ENSSSCG00000013756 / ENSG00000037757
MROH1 / Q8NDA8 / ENSSSCG00000005924 / ENSG00000179832
MRPL10 / Q7Z7H8 / ENSSSCG00000017519 / ENSG00000159111
MRPL12 / P52815 / ENSSSCG00000033148 / ENSG00000262814
MRPL15 / Q9P015 / ENSSSCG00000030578 / ENSG00000137547
MRPL19 / P49406 / ENSSSCG00000021007 / ENSG00000115364
MRPL2 / Q5T653 / ENSSSCG00000001658 / ENSG00000112651
MRPL3 / P09001 / ENSSSCG00000011179 / ENSG00000114686
MRPL37 / Q9BZE1 / ENSSSCG00000032083 / ENSG00000116221
MRPL38 / Q96DV4 / ENSSSCG00000017196 / ENSG00000204316
MRPL4 / Q9BYD3 / ENSSSCG00000013656 / ENSG00000105364
MRPL40 / Q9NQ50 / ENSSSCG00000034014 / ENSG00000185608
MRPL41 / Q8IXM3 / ENSSSCG00000052504 / ENSG00000182154
MRPL43 / Q8N983 / ENSSSCG00000010560 / ENSG00000055950
MRPL46 / Q9H2W6 / ENSSSCG00000005105 / ENSG00000259494
MRPL47 / Q9HD33 / ENSSSCG00000011763 / ENSG00000136522
MRPL50 / Q8N5N7 / ENSSSCG00000027974 / ENSG00000136897
MRPL9 / Q9BYD2 / ENSSSCG00000028343 / ENSG00000143436
MRPS2 / Q9Y399 / ENSSSCG00000005759 / ENSG00000122140
MRPS22 / P82650 / ENSSSCG00000035771 / ENSG00000175110
MRPS23 / Q9Y3D9 / ENSSSCG00000035193 / ENSG00000181610
MRPS25 / P82663 / ENSSSCG00000034318 / ENSG00000131368
MRPS26 / Q9BYN8 / ENSSSCG00000007165 / ENSG00000125901
MRPS27 / Q92552 / ENSSSCG00000023818 / ENSG00000113048
Homologs
Adipose.Tissue
Brain
Colon
Heart
Kidney
Liver
Lung
Pancreas
S.Intestine
Spleen
Organs
Species
Human
Pig
Bins
1
2
3
4
5

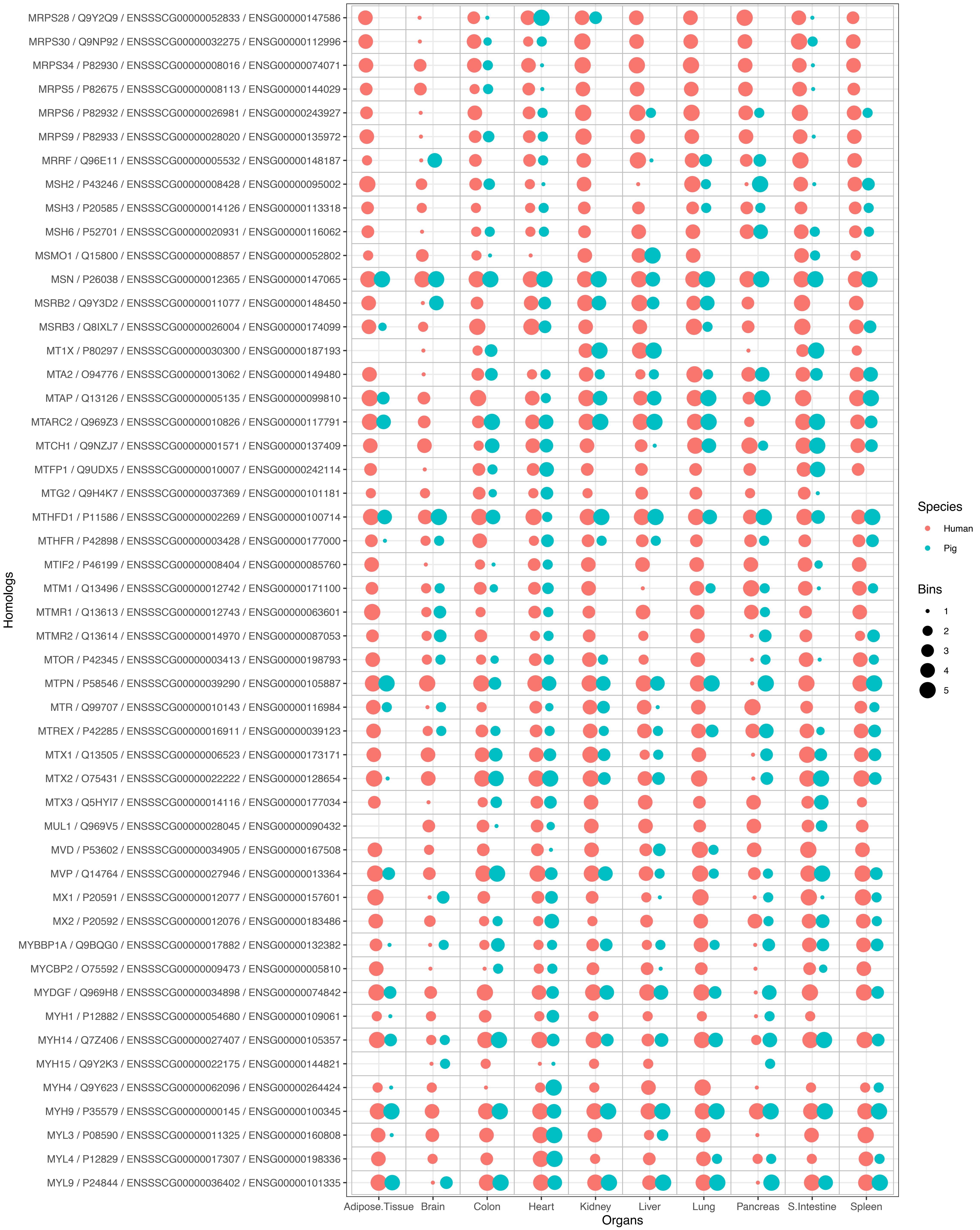
MRPS28 / Q9Y2Q9 / ENSSSCG00000052833 / ENSG00000147586
MRPS30 / Q9NP92 / ENSSSCG00000032275 / ENSG00000112996
MRPS34 / P82930 / ENSSSCG00000008016 / ENSG00000074071
MRPS5 / P82675 / ENSSSCG00000008113 / ENSG00000144029
MRPS6 / P82932 / ENSSSCG00000026981 / ENSG00000243927
MRPS9 / P82933 / ENSSSCG00000028020 / ENSG00000135972
MRRF / Q96E11 / ENSSSCG00000005532 / ENSG00000148187
MSH2 / P43246 / ENSSSCG00000008428 / ENSG00000095002
MSH3 / P20585 / ENSSSCG00000014126 / ENSG00000113318
MSH6 / P52701 / ENSSSCG00000020931 / ENSG00000116062
MSMO1 / Q15800 / ENSSSCG00000008857 / ENSG00000052802
MSN / P26038 / ENSSSCG00000012365 / ENSG00000147065
MSRB2 / Q9Y3D2 / ENSSSCG00000011077 / ENSG00000148450
MSRB3 / Q8IXL7 / ENSSSCG00000026004 / ENSG00000174099
MT1X / P80297 / ENSSSCG00000030300 / ENSG00000187193
MTA2 / O94776 / ENSSSCG00000013062 / ENSG00000149480
MTAP / Q13126 / ENSSSCG00000005135 / ENSG00000099810
MTARC2 / Q969Z3 / ENSSSCG00000010826 / ENSG00000117791
MTCH1 / Q9NZJ7 / ENSSSCG00000001571 / ENSG00000137409
MTFP1 / Q9UDX5 / ENSSSCG00000010007 / ENSG00000242114
MTG2 / Q9H4K7 / ENSSSCG00000037369 / ENSG00000101181
MTHFD1 / P11586 / ENSSSCG00000002269 / ENSG00000100714
MTHFR / P42898 / ENSSSCG00000003428 / ENSG00000177000
MTIF2 / P46199 / ENSSSCG00000008404 / ENSG00000085760
MTM1 / Q13496 / ENSSSCG00000012742 / ENSG00000171100
MTMR1 / Q13613 / ENSSSCG00000012743 / ENSG00000063601
MTMR2 / Q13614 / ENSSSCG00000014970 / ENSG00000087053
MTOR / P42345 / ENSSSCG00000003413 / ENSG00000198793
MTPN / P58546 / ENSSSCG00000039290 / ENSG00000105887
MTR / Q99707 / ENSSSCG00000010143 / ENSG00000116984
MTREX / P42285 / ENSSSCG00000016911 / ENSG00000039123
MTX1 / Q13505 / ENSSSCG00000006523 / ENSG00000173171
MTX2 / O75431 / ENSSSCG00000022222 / ENSG00000128654
MTX3 / Q5HYI7 / ENSSSCG00000014116 / ENSG00000177034
MUL1 / Q969V5 / ENSSSCG00000028045 / ENSG00000090432
MVD / P53602 / ENSSSCG00000034905 / ENSG00000167508
MVP / Q14764 / ENSSSCG00000027946 / ENSG00000013364
MX1 / P20591 / ENSSSCG00000012077 / ENSG00000157601
MX2 / P20592 / ENSSSCG00000012076 / ENSG00000183486
MYBBP1A / Q9BQG0 / ENSSSCG00000017882 / ENSG00000132382
MYCBP2 / O75592 / ENSSSCG00000009473 / ENSG00000005810
MYDGF / Q969H8 / ENSSSCG00000034898 / ENSG00000074842
MYH1 / P12882 / ENSSSCG00000054680 / ENSG00000109061
MYH14 / Q7Z406 / ENSSSCG00000027407 / ENSG00000105357
MYH15 / Q9Y2K3 / ENSSSCG00000022175 / ENSG00000144821
MYH4 / Q9Y623 / ENSSSCG00000062096 / ENSG00000264424
MYH9 / P35579 / ENSSSCG00000000145 / ENSG00000100345
MYL3 / P08590 / ENSSSCG00000011325 / ENSG00000160808
MYL4 / P12829 / ENSSSCG00000017307 / ENSG00000198336
MYL9 / P24844 / ENSSSCG00000036402 / ENSG00000101335
Homologs
Adipose.Tissue
Brain
Colon
Heart
Kidney
Liver
Lung
Pancreas
S.Intestine
Spleen
Organs
Species
Human
Pig
Bins
1
2
3
4
5

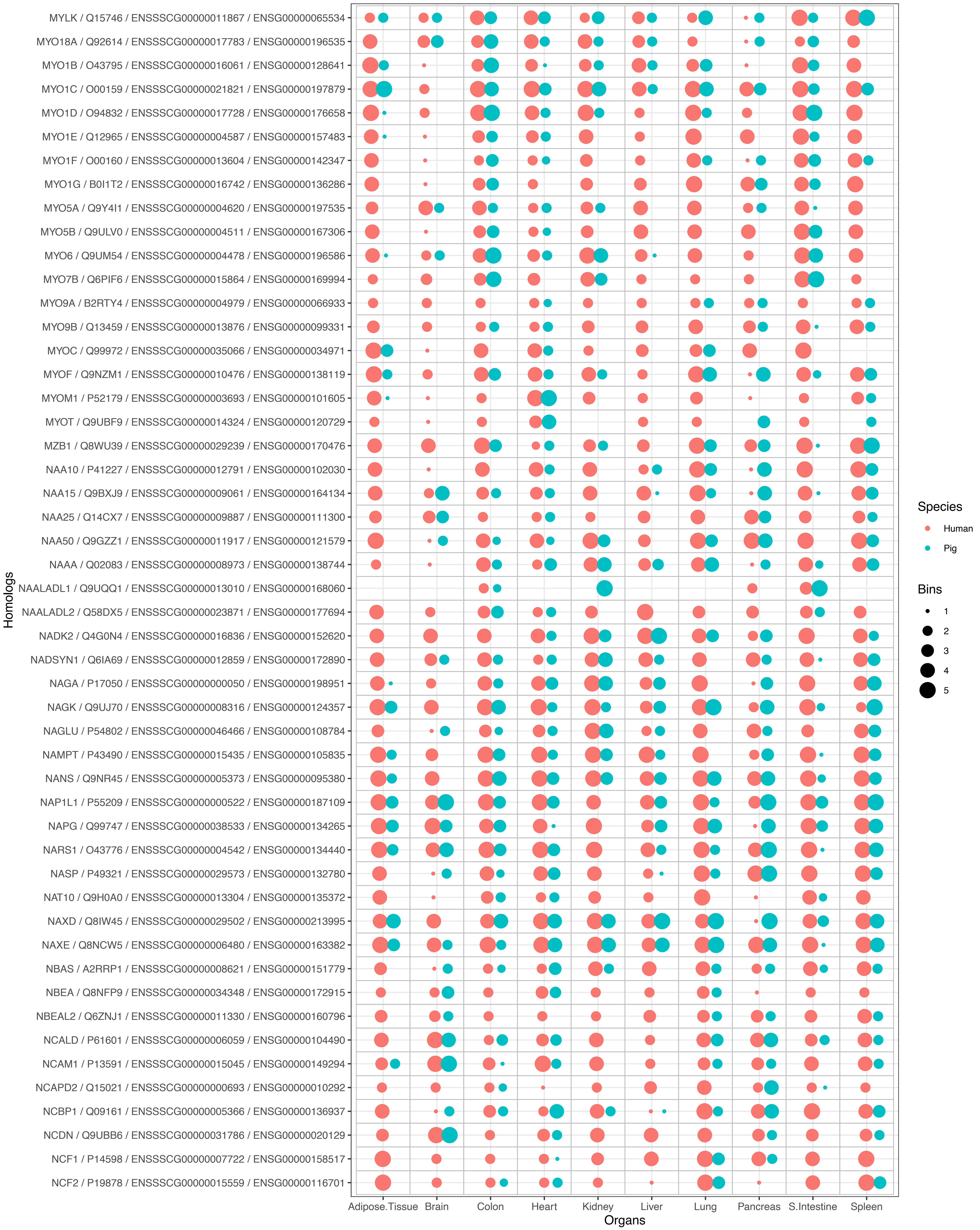

Homologs
MYLK / Q15746 / ENSSSCG00000011867 / ENSG00000065534
MYO18A / Q92614 / ENSSSCG00000017783 / ENSG00000196535
MYO1B / O43795 / ENSSSCG00000016061 / ENSG00000128641
MYO1C / O00159 / ENSSSCG00000021821 / ENSG00000197879
MYO1D / O94832 / ENSSSCG00000017728 / ENSG00000176658
MYO1E / Q12965 / ENSSSCG00000004587 / ENSG00000157483
MYO1F / O00160 / ENSSSCG00000013604 / ENSG00000142347
MYO1G / B0I1T2 / ENSSSCG00000016742 / ENSG00000136286
MYO5A / Q9Y4I1 / ENSSSCG00000004620 / ENSG00000197535
MYO5B / Q9ULV0 / ENSSSCG00000004511 / ENSG00000167306
MYO6 / Q9UM54 / ENSSSCG00000004478 / ENSG00000196586
MYO7B / Q6PIF6 / ENSSSCG00000015864 / ENSG00000169994
MYO9A / B2RTY4 / ENSSSCG00000004979 / ENSG00000066933
MYO9B / Q13459 / ENSSSCG00000013876 / ENSG00000099331
MYOC / Q99972 / ENSSSCG00000035066 / ENSG00000034971
MYOF / Q9NZM1 / ENSSSCG00000010476 / ENSG00000138119
MYOM1 / P52179 / ENSSSCG00000003693 / ENSG00000101605
MYOT / Q9UBF9 / ENSSSCG00000014324 / ENSG00000120729
MZB1 / Q8WU39 / ENSSSCG00000029239 / ENSG00000170476
NAA10 / P41227 / ENSSSCG00000012791 / ENSG00000102030
NAA15 / Q9BXJ9 / ENSSSCG00000009061 / ENSG00000164134
NAA25 / Q14CX7 / ENSSSCG00000009887 / ENSG00000111300
NAA50 / Q9GZZ1 / ENSSSCG00000011917 / ENSG00000121579
NAAA / Q02083 / ENSSSCG00000008973 / ENSG00000138744
NAALADL1 / Q9UQQ1 / ENSSSCG00000013010 / ENSG00000168060
NAALADL2 / Q58DX5 / ENSSSCG00000023871 / ENSG00000177694
NADK2 / Q4G0N4 / ENSSSCG00000016836 / ENSG00000152620
NADSYN1 / Q6IA69 / ENSSSCG00000012859 / ENSG00000172890
NAGA / P17050 / ENSSSCG00000000050 / ENSG00000198951
NAGK / Q9UJ70 / ENSSSCG00000008316 / ENSG00000124357
NAGLU / P54802 / ENSSSCG00000046466 / ENSG00000108784
NAMPT / P43490 / ENSSSCG00000015435 / ENSG00000105835
NANS / Q9NR45 / ENSSSCG00000005373 / ENSG00000095380
NAP1L1 / P55209 / ENSSSCG00000000522 / ENSG00000187109
NAPG / Q99747 / ENSSSCG00000038533 / ENSG00000134265
NARS1 / O43776 / ENSSSCG00000004542 / ENSG00000134440
NASP / P49321 / ENSSSCG00000029573 / ENSG00000132780
NAT10 / Q9H0A0 / ENSSSCG00000013304 / ENSG00000135372
NAXD / Q8IW45 / ENSSSCG00000029502 / ENSG00000213995
NAXE / Q8NCW5 / ENSSSCG00000006480 / ENSG00000163382
NBAS / A2RRP1 / ENSSSCG00000008621 / ENSG00000151779
NBEA / Q8NFP9 / ENSSSCG00000034348 / ENSG00000172915
NBEAL2 / Q6ZNJ1 / ENSSSCG00000011330 / ENSG00000160796
NCALD / P61601 / ENSSSCG00000006059 / ENSG00000104490
NCAM1 / P13591 / ENSSSCG00000015045 / ENSG00000149294
NCAPD2 / Q15021 / ENSSSCG00000000693 / ENSG00000010292
NCBP1 / Q09161 / ENSSSCG00000005366 / ENSG00000136937
NCDN / Q9UBB6 / ENSSSCG00000031786 / ENSG00000020129
NCF1 / P14598 / ENSSSCG00000007722 / ENSG00000158517
NCF2 / P19878 / ENSSSCG00000015559 / ENSG00000116701
Adipose.Tissue
Brain
Colon
Heart
Kidney
Liver
Lung
Pancreas
S.Intestine
Spleen
Organs
Species
Human
Pig
Bins
1
2
3
4
5

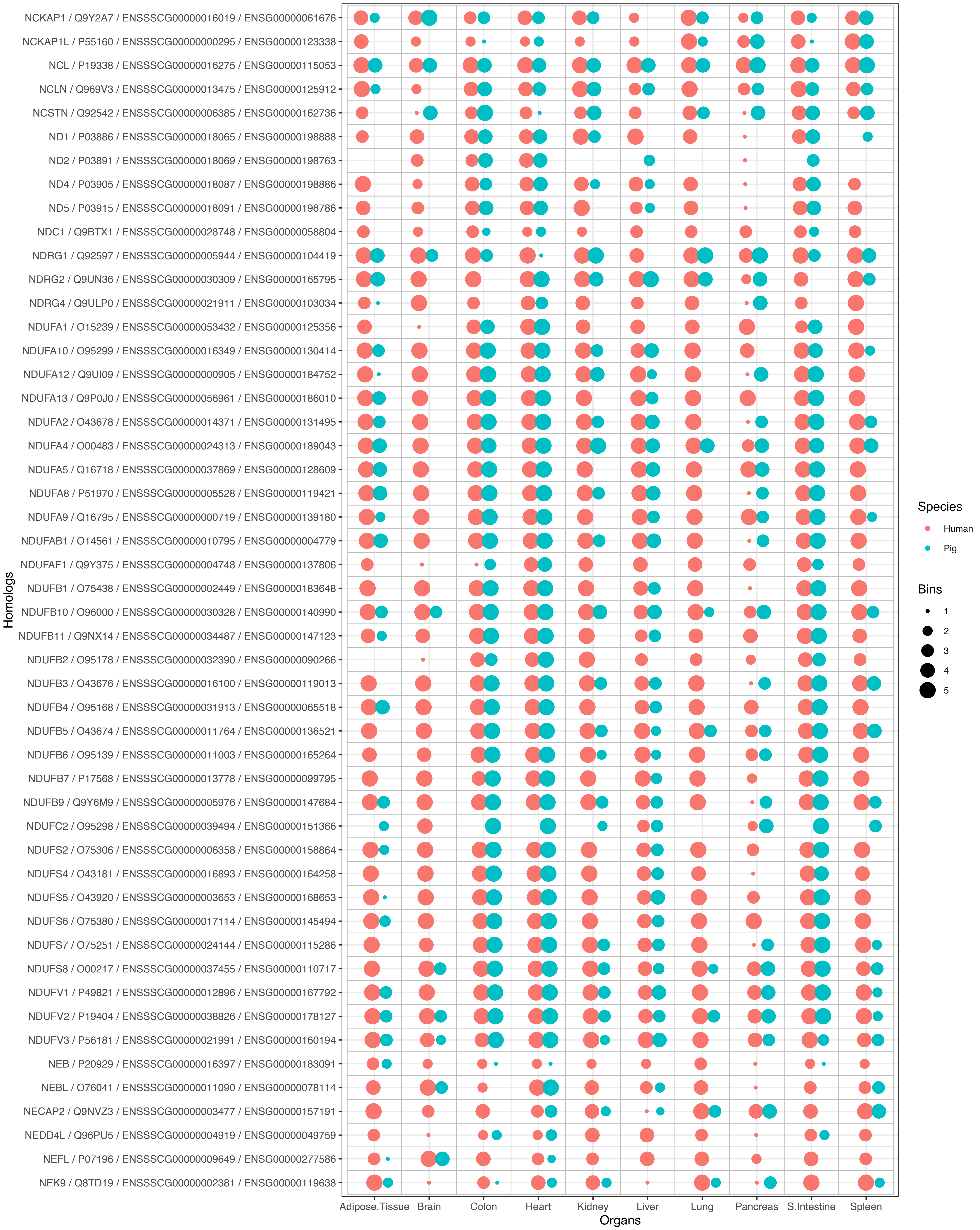
NCKAP1 / Q9Y2A7 / ENSSSCG00000016019 / ENSG00000061676
NCKAP1L / P55160 / ENSSSCG00000000295 / ENSG00000123338
NCL / P19338 / ENSSSCG00000016275 / ENSG00000115053
NCLN / Q969V3 / ENSSSCG00000013475 / ENSG00000125912
NCSTN / Q92542 / ENSSSCG00000006385 / ENSG00000162736
ND1 / P03886 / ENSSSCG00000018065 / ENSG00000198888
ND2 / P03891 / ENSSSCG00000018069 / ENSG00000198763
ND4 / P03905 / ENSSSCG00000018087 / ENSG00000198886
ND5 / P03915 / ENSSSCG00000018091 / ENSG00000198786
NDC1 / Q9BTX1 / ENSSSCG00000028748 / ENSG00000058804
NDRG1 / Q92597 / ENSSSCG00000005944 / ENSG00000104419
NDRG2 / Q9UN36 / ENSSSCG00000030309 / ENSG00000165795
NDRG4 / Q9ULP0 / ENSSSCG00000021911 / ENSG00000103034
NDUFA1 / O15239 / ENSSSCG00000053432 / ENSG00000125356
NDUFA10 / O95299 / ENSSSCG00000016349 / ENSG00000130414
NDUFA12 / Q9UI09 / ENSSSCG00000000905 / ENSG00000184752
NDUFA13 / Q9P0J0 / ENSSSCG00000056961 / ENSG00000186010
NDUFA2 / O43678 / ENSSSCG00000014371 / ENSG00000131495
NDUFA4 / O00483 / ENSSSCG00000024313 / ENSG00000189043
NDUFA5 / Q16718 / ENSSSCG00000037869 / ENSG00000128609
NDUFA8 / P51970 / ENSSSCG00000005528 / ENSG00000119421
NDUFA9 / Q16795 / ENSSSCG00000000719 / ENSG00000139180
NDUFAB1 / O14561 / ENSSSCG00000010795 / ENSG00000004779
NDUFAF1 / Q9Y375 / ENSSSCG00000004748 / ENSG00000137806
NDUFB1 / O75438 / ENSSSCG00000002449 / ENSG00000183648
NDUFB10 / O96000 / ENSSSCG00000030328 / ENSG00000140990
NDUFB11 / Q9NX14 / ENSSSCG00000034487 / ENSG00000147123
NDUFB2 / O95178 / ENSSSCG00000032390 / ENSG00000090266
NDUFB3 / O43676 / ENSSSCG00000016100 / ENSG00000119013
NDUFB4 / O95168 / ENSSSCG00000031913 / ENSG00000065518
NDUFB5 / O43674 / ENSSSCG00000011764 / ENSG00000136521
NDUFB6 / O95139 / ENSSSCG00000011003 / ENSG00000165264
NDUFB7 / P17568 / ENSSSCG00000013778 / ENSG00000099795
NDUFB9 / Q9Y6M9 / ENSSSCG00000005976 / ENSG00000147684
NDUFC2 / O95298 / ENSSSCG00000039494 / ENSG00000151366
NDUFS2 / O75306 / ENSSSCG00000006358 / ENSG00000158864
NDUFS4 / O43181 / ENSSSCG00000016893 / ENSG00000164258
NDUFS5 / O43920 / ENSSSCG00000003653 / ENSG00000168653
NDUFS6 / O75380 / ENSSSCG00000017114 / ENSG00000145494
NDUFS7 / O75251 / ENSSSCG00000024144 / ENSG00000115286
NDUFS8 / O00217 / ENSSSCG00000037455 / ENSG00000110717
NDUFV1 / P49821 / ENSSSCG00000012896 / ENSG00000167792
NDUFV2 / P19404 / ENSSSCG00000038826 / ENSG00000178127
NDUFV3 / P56181 / ENSSSCG00000021991 / ENSG00000160194
NEB / P20929 / ENSSSCG00000016397 / ENSG00000183091
NEBL / O76041 / ENSSSCG00000011090 / ENSG00000078114
NECAP2 / Q9NVZ3 / ENSSSCG00000003477 / ENSG00000157191
NEDD4L / Q96PU5 / ENSSSCG00000004919 / ENSG00000049759
NEFL / P07196 / ENSSSCG00000009649 / ENSG00000277586
NEK9 / Q8TD19 / ENSSSCG00000002381 / ENSG00000119638
Homologs
Adipose.Tissue
Brain
Colon
Heart
Kidney
Liver
Lung
Pancreas
S.Intestine
Spleen
Organs
Species
Human
Pig
Bins
1
2
3
4
5

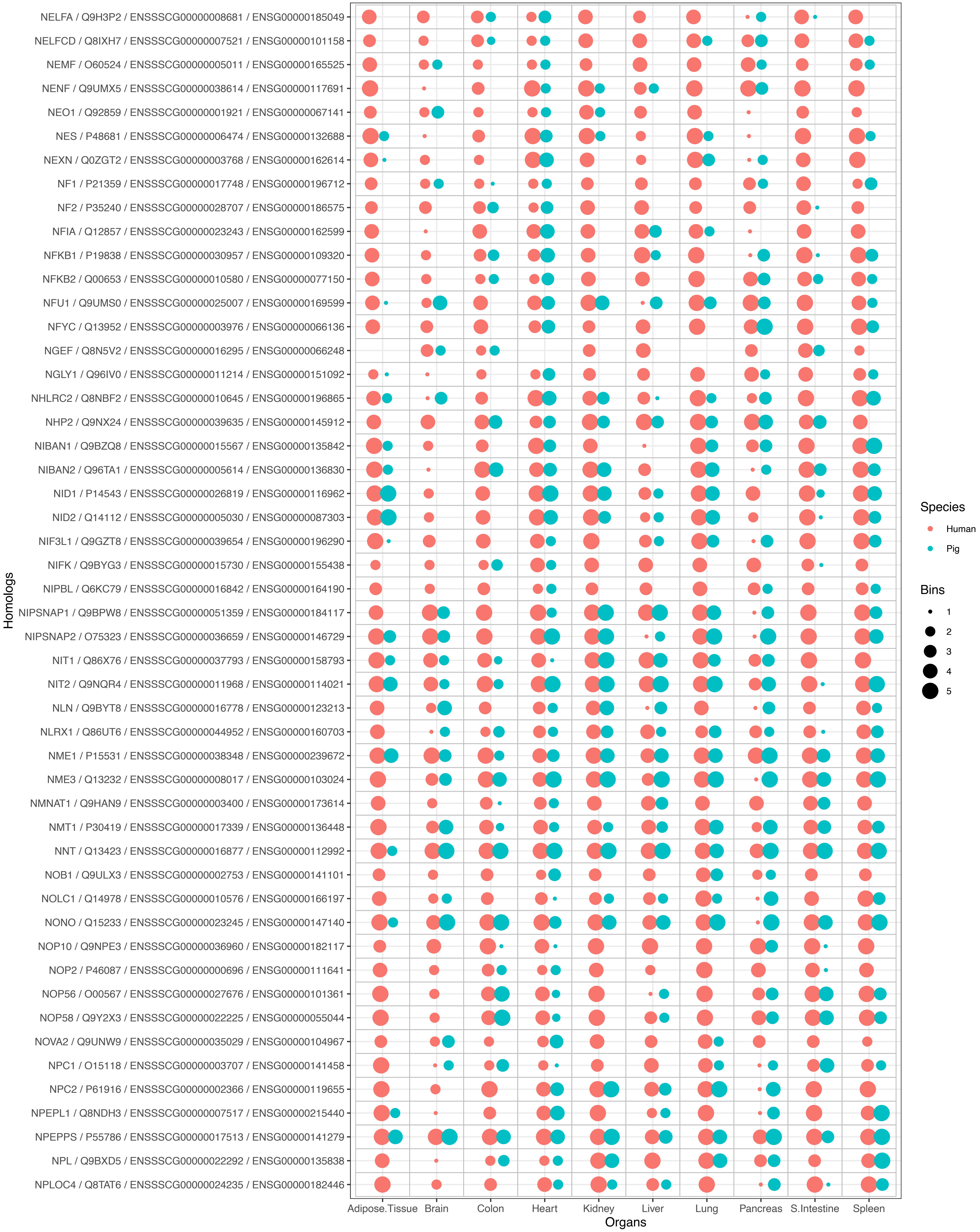
NELFA / Q9H3P2 / ENSSSCG00000008681 / ENSG00000185049
NELFCD / Q8IXH7 / ENSSSCG00000007521 / ENSG00000101158
NEMF / O60524 / ENSSSCG00000005011 / ENSG00000165525
NENF / Q9UMX5 / ENSSSCG00000038614 / ENSG00000117691
NEO1 / Q92859 / ENSSSCG00000001921 / ENSG00000067141
NES / P48681 / ENSSSCG00000006474 / ENSG00000132688
NEXN / Q0ZGT2 / ENSSSCG00000003768 / ENSG00000162614
NF1 / P21359 / ENSSSCG00000017748 / ENSG00000196712
NF2 / P35240 / ENSSSCG00000028707 / ENSG00000186575
NFIA / Q12857 / ENSSSCG00000023243 / ENSG00000162599
NFKB1 / P19838 / ENSSSCG00000030957 / ENSG00000109320
NFKB2 / Q00653 / ENSSSCG00000010580 / ENSG00000077150
NFU1 / Q9UMS0 / ENSSSCG00000025007 / ENSG00000169599
NFYC / Q13952 / ENSSSCG00000003976 / ENSG00000066136
NGEF / Q8N5V2 / ENSSSCG00000016295 / ENSG00000066248
NGLY1 / Q96IV0 / ENSSSCG00000011214 / ENSG00000151092
NHLRC2 / Q8NBF2 / ENSSSCG00000010645 / ENSG00000196865
NHP2 / Q9NX24 / ENSSSCG00000039635 / ENSG00000145912
NIBAN1 / Q9BZQ8 / ENSSSCG00000015567 / ENSG00000135842
NIBAN2 / Q96TA1 / ENSSSCG00000005614 / ENSG00000136830
NID1 / P14543 / ENSSSCG00000026819 / ENSG00000116962
NID2 / Q14112 / ENSSSCG00000005030 / ENSG00000087303
NIF3L1 / Q9GZT8 / ENSSSCG00000039654 / ENSG00000196290
NIFK / Q9BYG3 / ENSSSCG00000015730 / ENSG00000155438
NIPBL / Q6KC79 / ENSSSCG00000016842 / ENSG00000164190
NIPSNAP1 / Q9BPW8 / ENSSSCG00000051359 / ENSG00000184117
NIPSNAP2 / O75323 / ENSSSCG00000036659 / ENSG00000146729
NIT1 / Q86X76 / ENSSSCG00000037793 / ENSG00000158793
NIT2 / Q9NQR4 / ENSSSCG00000011968 / ENSG00000114021
NLN / Q9BYT8 / ENSSSCG00000016778 / ENSG00000123213
NLRX1 / Q86UT6 / ENSSSCG00000044952 / ENSG00000160703
NME1 / P15531 / ENSSSCG00000038348 / ENSG00000239672
NME3 / Q13232 / ENSSSCG00000008017 / ENSG00000103024
NMNAT1 / Q9HAN9 / ENSSSCG00000003400 / ENSG00000173614
NMT1 / P30419 / ENSSSCG00000017339 / ENSG00000136448
NNT / Q13423 / ENSSSCG00000016877 / ENSG00000112992
NOB1 / Q9ULX3 / ENSSSCG00000002753 / ENSG00000141101
NOLC1 / Q14978 / ENSSSCG00000010576 / ENSG00000166197
NONO / Q15233 / ENSSSCG00000023245 / ENSG00000147140
NOP10 / Q9NPE3 / ENSSSCG00000036960 / ENSG00000182117
NOP2 / P46087 / ENSSSCG00000000696 / ENSG00000111641
NOP56 / O00567 / ENSSSCG00000027676 / ENSG00000101361
NOP58 / Q9Y2X3 / ENSSSCG00000022225 / ENSG00000055044
NOVA2 / Q9UNW9 / ENSSSCG00000035029 / ENSG00000104967
NPC1 / O15118 / ENSSSCG00000003707 / ENSG00000141458
NPC2 / P61916 / ENSSSCG00000002366 / ENSG00000119655
NPEPL1 / Q8NDH3 / ENSSSCG00000007517 / ENSG00000215440
NPEPPS / P55786 / ENSSSCG00000017513 / ENSG00000141279
NPL / Q9BXD5 / ENSSSCG00000022292 / ENSG00000135838
NPLOC4 / Q8TAT6 / ENSSSCG00000024235 / ENSG00000182446
Homologs
Adipose.Tissue
Brain
Colon
Heart
Kidney
Liver
Lung
Pancreas
S.Intestine
Spleen
Organs
Species
Human
Pig
Bins
1
2
3
4
5

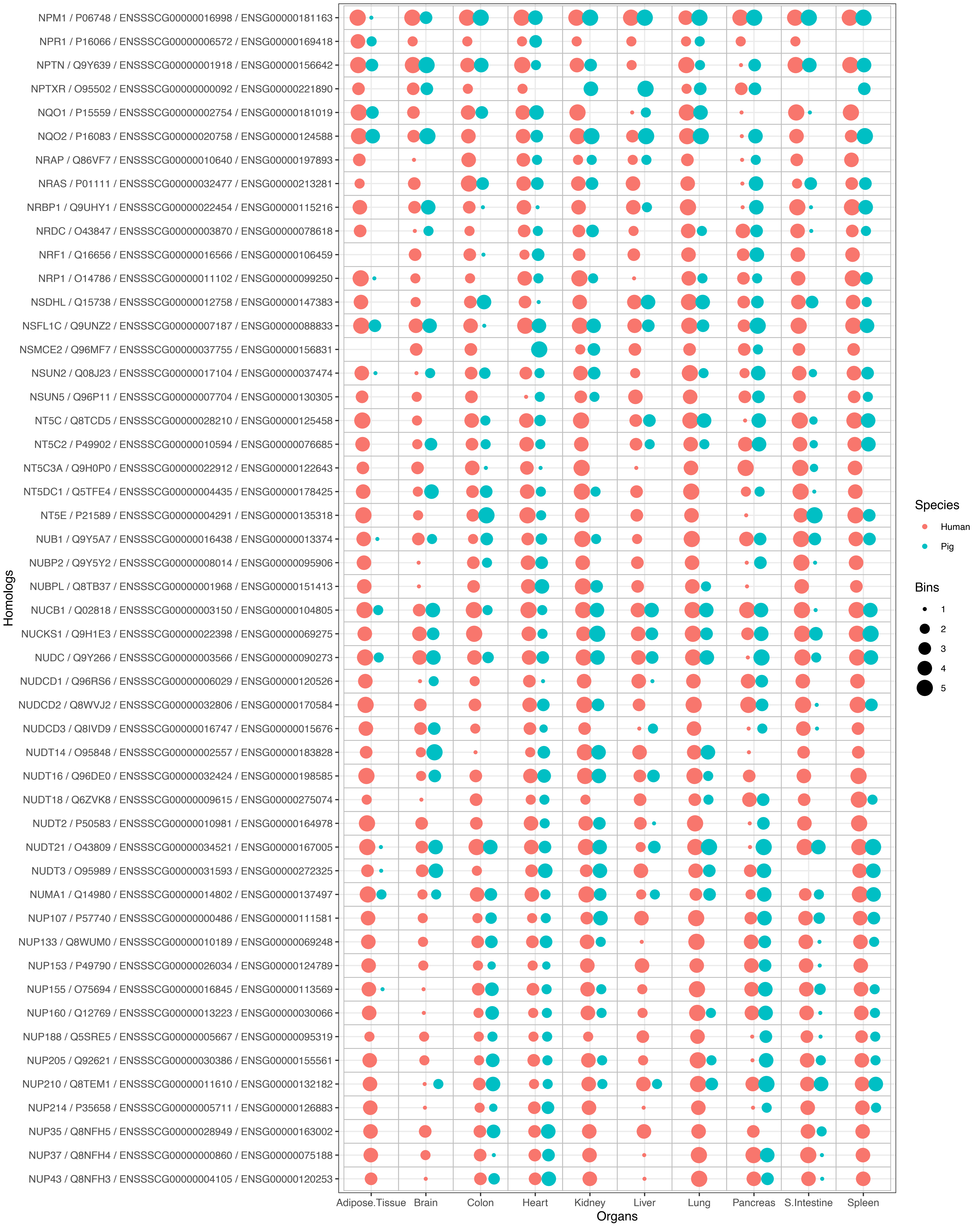

NPM1 / P06748 / ENSSSCG00000016998 / ENSG00000181163
NPR1 / P16066 / ENSSSCG00000006572 / ENSG00000169418
NPTN / Q9Y639 / ENSSSCG00000001918 / ENSG00000156642
NPTXR / O95502 / ENSSSCG00000000092 / ENSG00000221890
NQO1 / P15559 / ENSSSCG00000002754 / ENSG00000181019
NQO2 / P16083 / ENSSSCG00000020758 / ENSG00000124588
NRAP / Q86VF7 / ENSSSCG00000010640 / ENSG00000197893
NRAS / P01111 / ENSSSCG00000032477 / ENSG00000213281
NRBP1 / Q9UHY1 / ENSSSCG00000022454 / ENSG00000115216
NRDC / O43847 / ENSSSCG00000003870 / ENSG00000078618
NRF1 / Q16656 / ENSSSCG00000016566 / ENSG00000106459
NRP1 / O14786 / ENSSSCG00000011102 / ENSG00000099250
NSDHL / Q15738 / ENSSSCG00000012758 / ENSG00000147383
NSFL1C / Q9UNZ2 / ENSSSCG00000007187 / ENSG00000088833
NSMCE2 / Q96MF7 / ENSSSCG00000037755 / ENSG00000156831
NSUN2 / Q08J23 / ENSSSCG00000017104 / ENSG00000037474
NSUN5 / Q96P11 / ENSSSCG00000007704 / ENSG00000130305
NT5C / Q8TCD5 / ENSSSCG00000028210 / ENSG00000125458
NT5C2 / P49902 / ENSSSCG00000010594 / ENSG00000076685
NT5C3A / Q9H0P0 / ENSSSCG00000022912 / ENSG00000122643
NT5DC1 / Q5TFE4 / ENSSSCG00000004435 / ENSG00000178425
NT5E / P21589 / ENSSSCG00000004291 / ENSG00000135318
NUB1 / Q9Y5A7 / ENSSSCG00000016438 / ENSG00000013374
NUBP2 / Q9Y5Y2 / ENSSSCG00000008014 / ENSG00000095906
NUBPL / Q8TB37 / ENSSSCG00000001968 / ENSG00000151413
NUCB1 / Q02818 / ENSSSCG00000003150 / ENSG00000104805
NUCKS1 / Q9H1E3 / ENSSSCG00000022398 / ENSG00000069275
NUDC / Q9Y266 / ENSSSCG00000003566 / ENSG00000090273
NUDCD1 / Q96RS6 / ENSSSCG00000006029 / ENSG00000120526
NUDCD2 / Q8WVJ2 / ENSSSCG00000032806 / ENSG00000170584
NUDCD3 / Q8IVD9 / ENSSSCG00000016747 / ENSG00000015676
NUDT14 / O95848 / ENSSSCG00000002557 / ENSG00000183828
NUDT16 / Q96DE0 / ENSSSCG00000032424 / ENSG00000198585
NUDT18 / Q6ZVK8 / ENSSSCG00000009615 / ENSG00000275074
NUDT2 / P50583 / ENSSSCG00000010981 / ENSG00000164978
NUDT21 / O43809 / ENSSSCG00000034521 / ENSG00000167005
NUDT3 / O95989 / ENSSSCG00000031593 / ENSG00000272325
NUMA1 / Q14980 / ENSSSCG00000014802 / ENSG00000137497
NUP107 / P57740 / ENSSSCG00000000486 / ENSG00000111581
NUP133 / Q8WUM0 / ENSSSCG00000010189 / ENSG00000069248
NUP153 / P49790 / ENSSSCG00000026034 / ENSG00000124789
NUP155 / O75694 / ENSSSCG00000016845 / ENSG00000113569
NUP160 / Q12769 / ENSSSCG00000013223 / ENSG00000030066
NUP188 / Q5SRE5 / ENSSSCG00000005667 / ENSG00000095319
NUP205 / Q92621 / ENSSSCG00000030386 / ENSG00000155561
NUP210 / Q8TEM1 / ENSSSCG00000011610 / ENSG00000132182
NUP214 / P35658 / ENSSSCG00000005711 / ENSG00000126883
NUP35 / Q8NFH5 / ENSSSCG00000028949 / ENSG00000163002
NUP37 / Q8NFH4 / ENSSSCG00000000860 / ENSG00000075188
NUP43 / Q8NFH3 / ENSSSCG00000004105 / ENSG00000120253
Homologs
Adipose.Tissue
Brain
Colon
Heart
Kidney
Liver
Lung
Pancreas
S.Intestine
Spleen
Organs
Species
Human
Pig
Bins
1
2
3
4
5

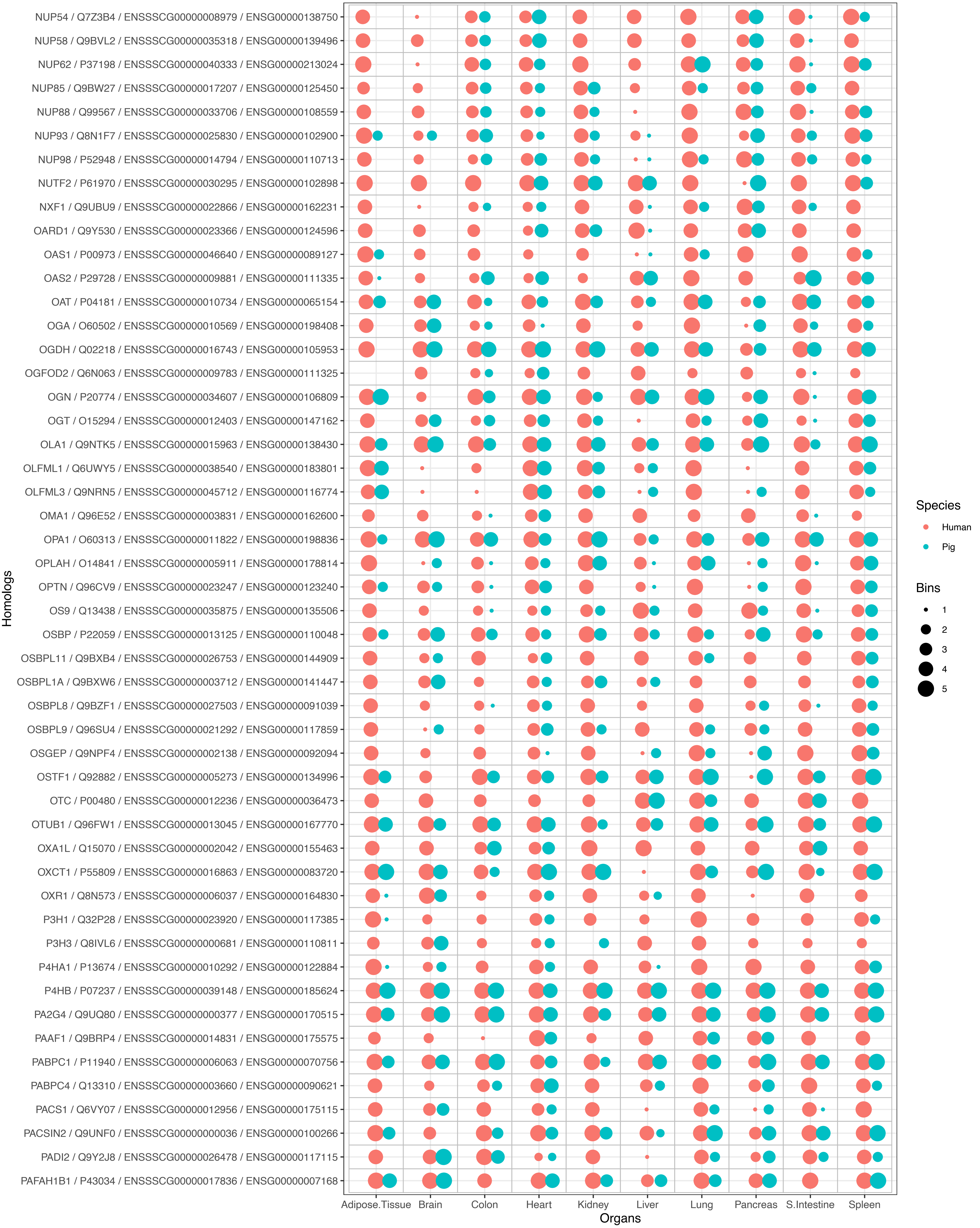
NUP54 / Q7Z3B4 / ENSSSCG00000008979 / ENSG00000138750
NUP58 / Q9BVL2 / ENSSSCG00000035318 / ENSG00000139496
NUP62 / P37198 / ENSSSCG00000040333 / ENSG00000213024
NUP85 / Q9BW27 / ENSSSCG00000017207 / ENSG00000125450
NUP88 / Q99567 / ENSSSCG00000033706 / ENSG00000108559
NUP93 / Q8N1F7 / ENSSSCG00000025830 / ENSG00000102900
NUP98 / P52948 / ENSSSCG00000014794 / ENSG00000110713
NUTF2 / P61970 / ENSSSCG00000030295 / ENSG00000102898
NXF1 / Q9UBU9 / ENSSSCG00000022866 / ENSG00000162231
OARD1 / Q9Y530 / ENSSSCG00000023366 / ENSG00000124596
OAS1 / P00973 / ENSSSCG00000046640 / ENSG00000089127
OAS2 / P29728 / ENSSSCG00000009881 / ENSG00000111335
OAT / P04181 / ENSSSCG00000010734 / ENSG00000065154
OGA / O60502 / ENSSSCG00000010569 / ENSG00000198408
OGDH / Q02218 / ENSSSCG00000016743 / ENSG00000105953
OGFOD2 / Q6N063 / ENSSSCG00000009783 / ENSG00000111325
OGN / P20774 / ENSSSCG00000034607 / ENSG00000106809
OGT / O15294 / ENSSSCG00000012403 / ENSG00000147162
OLA1 / Q9NTK5 / ENSSSCG00000015963 / ENSG00000138430
OLFML1 / Q6UWY5 / ENSSSCG00000038540 / ENSG00000183801
OLFML3 / Q9NRN5 / ENSSSCG00000045712 / ENSG00000116774
OMA1 / Q96E52 / ENSSSCG00000003831 / ENSG00000162600
OPA1 / O60313 / ENSSSCG00000011822 / ENSG00000198836
OPLAH / O14841 / ENSSSCG00000005911 / ENSG00000178814
OPTN / Q96CV9 / ENSSSCG00000023247 / ENSG00000123240
OS9 / Q13438 / ENSSSCG00000035875 / ENSG00000135506
OSBP / P22059 / ENSSSCG00000013125 / ENSG00000110048
OSBPL11 / Q9BXB4 / ENSSSCG00000026753 / ENSG00000144909
OSBPL1A / Q9BXW6 / ENSSSCG00000003712 / ENSG00000141447
OSBPL8 / Q9BZF1 / ENSSSCG00000027503 / ENSG00000091039
OSBPL9 / Q96SU4 / ENSSSCG00000021292 / ENSG00000117859
OSGEP / Q9NPF4 / ENSSSCG00000002138 / ENSG00000092094
OSTF1 / Q92882 / ENSSSCG00000005273 / ENSG00000134996
OTC / P00480 / ENSSSCG00000012236 / ENSG00000036473
OTUB1 / Q96FW1 / ENSSSCG00000013045 / ENSG00000167770
OXA1L / Q15070 / ENSSSCG00000002042 / ENSG00000155463
OXCT1 / P55809 / ENSSSCG00000016863 / ENSG00000083720
OXR1 / Q8N573 / ENSSSCG00000006037 / ENSG00000164830
P3H1 / Q32P28 / ENSSSCG00000023920 / ENSG00000117385
P3H3 / Q8IVL6 / ENSSSCG00000000681 / ENSG00000110811
P4HA1 / P13674 / ENSSSCG00000010292 / ENSG00000122884
P4HB / P07237 / ENSSSCG00000039148 / ENSG00000185624
PA2G4 / Q9UQ80 / ENSSSCG00000000377 / ENSG00000170515
PAAF1 / Q9BRP4 / ENSSSCG00000014831 / ENSG00000175575
PABPC1 / P11940 / ENSSSCG00000006063 / ENSG00000070756
PABPC4 / Q13310 / ENSSSCG00000003660 / ENSG00000090621
PACS1 / Q6VY07 / ENSSSCG00000012956 / ENSG00000175115
PACSIN2 / Q9UNF0 / ENSSSCG00000000036 / ENSG00000100266
PADI2 / Q9Y2J8 / ENSSSCG00000026478 / ENSG00000117115
PAFAH1B1 / P43034 / ENSSSCG00000017836 / ENSG00000007168
Homologs
Adipose.Tissue
Brain
Colon
Heart
Kidney
Liver
Lung
Pancreas
S.Intestine
Spleen
Organs
Species
Human
Pig
Bins
1
2
3
4
5

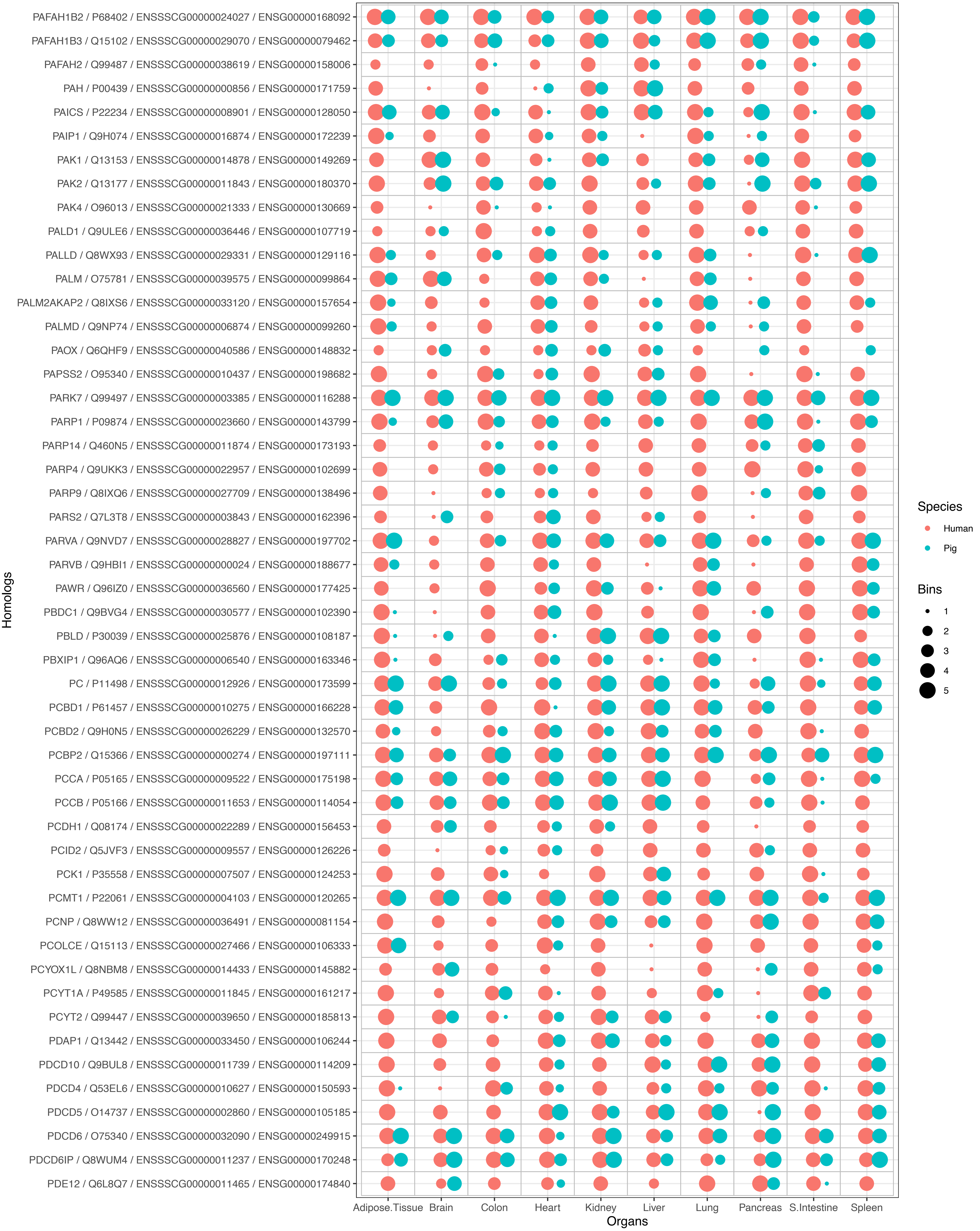
PAFAH1B2 / P68402 / ENSSSCG00000024027 / ENSG00000168092
PAFAH1B3 / Q15102 / ENSSSCG00000029070 / ENSG00000079462
PAFAH2 / Q99487 / ENSSSCG00000038619 / ENSG00000158006
PAH / P00439 / ENSSSCG00000000856 / ENSG00000171759
PAICS / P22234 / ENSSSCG00000008901 / ENSG00000128050
PAIP1 / Q9H074 / ENSSSCG00000016874 / ENSG00000172239
PAK1 / Q13153 / ENSSSCG00000014878 / ENSG00000149269
PAK2 / Q13177 / ENSSSCG00000011843 / ENSG00000180370
PAK4 / O96013 / ENSSSCG00000021333 / ENSG00000130669
PALD1 / Q9ULE6 / ENSSSCG00000036446 / ENSG00000107719
PALLD / Q8WX93 / ENSSSCG00000029331 / ENSG00000129116
PALM / O75781 / ENSSSCG00000039575 / ENSG00000099864
PALM2AKAP2 / Q8IXS6 / ENSSSCG00000033120 / ENSG00000157654
PALMD / Q9NP74 / ENSSSCG00000006874 / ENSG00000099260
PAOX / Q6QHF9 / ENSSSCG00000040586 / ENSG00000148832
PAPSS2 / O95340 / ENSSSCG00000010437 / ENSG00000198682
PARK7 / Q99497 / ENSSSCG00000003385 / ENSG00000116288
PARP1 / P09874 / ENSSSCG00000023660 / ENSG00000143799
PARP14 / Q460N5 / ENSSSCG00000011874 / ENSG00000173193
PARP4 / Q9UKK3 / ENSSSCG00000022957 / ENSG00000102699
PARP9 / Q8IXQ6 / ENSSSCG00000027709 / ENSG00000138496
PARS2 / Q7L3T8 / ENSSSCG00000003843 / ENSG00000162396
PARVA / Q9NVD7 / ENSSSCG00000028827 / ENSG00000197702
PARVB / Q9HBI1 / ENSSSCG00000000024 / ENSG00000188677
PAWR / Q96IZ0 / ENSSSCG00000036560 / ENSG00000177425
PBDC1 / Q9BVG4 / ENSSSCG00000030577 / ENSG00000102390
PBLD / P30039 / ENSSSCG00000025876 / ENSG00000108187
PBXIP1 / Q96AQ6 / ENSSSCG00000006540 / ENSG00000163346
PC / P11498 / ENSSSCG00000012926 / ENSG00000173599
PCBD1 / P61457 / ENSSSCG00000010275 / ENSG00000166228
PCBD2 / Q9H0N5 / ENSSSCG00000026229 / ENSG00000132570
PCBP2 / Q15366 / ENSSSCG00000000274 / ENSG00000197111
PCCA / P05165 / ENSSSCG00000009522 / ENSG00000175198
PCCB / P05166 / ENSSSCG00000011653 / ENSG00000114054
PCDH1 / Q08174 / ENSSSCG00000022289 / ENSG00000156453
PCID2 / Q5JVF3 / ENSSSCG00000009557 / ENSG00000126226
PCK1 / P35558 / ENSSSCG00000007507 / ENSG00000124253
PCMT1 / P22061 / ENSSSCG00000004103 / ENSG00000120265
PCNP / Q8WW12 / ENSSSCG00000036491 / ENSG00000081154
PCOLCE / Q15113 / ENSSSCG00000027466 / ENSG00000106333
PCYOX1L / Q8NBM8 / ENSSSCG00000014433 / ENSG00000145882
PCYT1A / P49585 / ENSSSCG00000011845 / ENSG00000161217
PCYT2 / Q99447 / ENSSSCG00000039650 / ENSG00000185813
PDAP1 / Q13442 / ENSSSCG00000033450 / ENSG00000106244
PDCD10 / Q9BUL8 / ENSSSCG00000011739 / ENSG00000114209
PDCD4 / Q53EL6 / ENSSSCG00000010627 / ENSG00000150593
PDCD5 / O14737 / ENSSSCG00000002860 / ENSG00000105185
PDCD6 / O75340 / ENSSSCG00000032090 / ENSG00000249915
PDCD6IP / Q8WUM4 / ENSSSCG00000011237 / ENSG00000170248
PDE12 / Q6L8Q7 / ENSSSCG00000011465 / ENSG00000174840
Homologs
Adipose.Tissue
Brain
Colon
Heart
Kidney
Liver
Lung
Pancreas
S.Intestine
Spleen
Organs
Species
Human
Pig
Bins
1
2
3
4
5

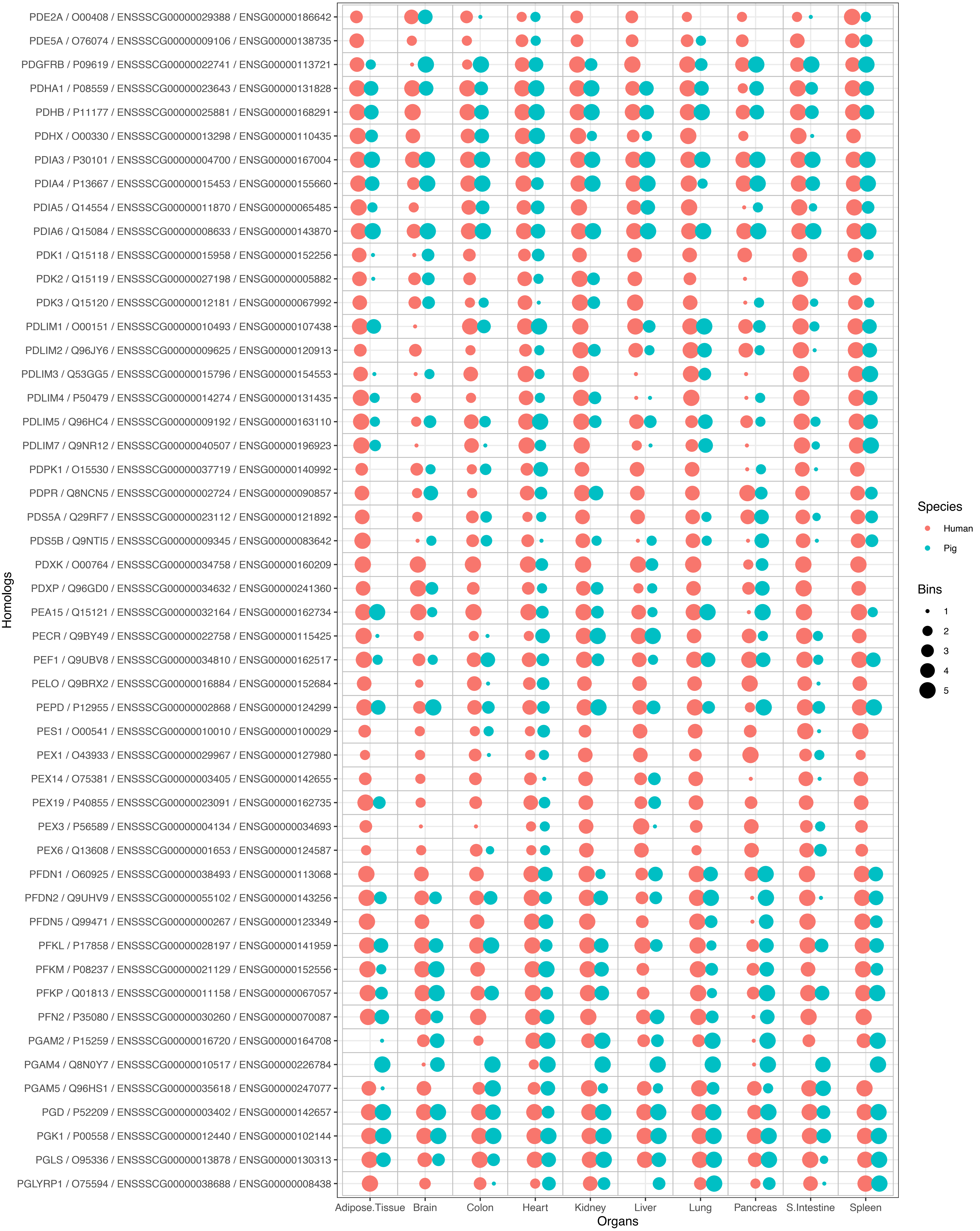
PDE2A / O00408 / ENSSSCG00000029388 / ENSG00000186642
PDE5A / O76074 / ENSSSCG00000009106 / ENSG00000138735
PDGFRB / P09619 / ENSSSCG00000022741 / ENSG00000113721
PDHA1 / P08559 / ENSSSCG00000023643 / ENSG00000131828
PDHB / P11177 / ENSSSCG00000025881 / ENSG00000168291
PDHX / O00330 / ENSSSCG00000013298 / ENSG00000110435
PDIA3 / P30101 / ENSSSCG00000004700 / ENSG00000167004
PDIA4 / P13667 / ENSSSCG00000015453 / ENSG00000155660
PDIA5 / Q14554 / ENSSSCG00000011870 / ENSG00000065485
PDIA6 / Q15084 / ENSSSCG00000008633 / ENSG00000143870
PDK1 / Q15118 / ENSSSCG00000015958 / ENSG00000152256
PDK2 / Q15119 / ENSSSCG00000027198 / ENSG00000005882
PDK3 / Q15120 / ENSSSCG00000012181 / ENSG00000067992
PDLIM1 / O00151 / ENSSSCG00000010493 / ENSG00000107438
PDLIM2 / Q96JY6 / ENSSSCG00000009625 / ENSG00000120913
PDLIM3 / Q53GG5 / ENSSSCG00000015796 / ENSG00000154553
PDLIM4 / P50479 / ENSSSCG00000014274 / ENSG00000131435
PDLIM5 / Q96HC4 / ENSSSCG00000009192 / ENSG00000163110
PDLIM7 / Q9NR12 / ENSSSCG00000040507 / ENSG00000196923
PDPK1 / O15530 / ENSSSCG00000037719 / ENSG00000140992
PDPR / Q8NCN5 / ENSSSCG00000002724 / ENSG00000090857
PDS5A / Q29RF7 / ENSSSCG00000023112 / ENSG00000121892
PDS5B / Q9NTI5 / ENSSSCG00000009345 / ENSG00000083642
PDXK / O00764 / ENSSSCG00000034758 / ENSG00000160209
PDXP / Q96GD0 / ENSSSCG00000034632 / ENSG00000241360
PEA15 / Q15121 / ENSSSCG00000032164 / ENSG00000162734
PECR / Q9BY49 / ENSSSCG00000022758 / ENSG00000115425
PEF1 / Q9UBV8 / ENSSSCG00000034810 / ENSG00000162517
PELO / Q9BRX2 / ENSSSCG00000016884 / ENSG00000152684
PEPD / P12955 / ENSSSCG00000002868 / ENSG00000124299
PES1 / O00541 / ENSSSCG00000010010 / ENSG00000100029
PEX1 / O43933 / ENSSSCG00000029967 / ENSG00000127980
PEX14 / O75381 / ENSSSCG00000003405 / ENSG00000142655
PEX19 / P40855 / ENSSSCG00000023091 / ENSG00000162735
PEX3 / P56589 / ENSSSCG00000004134 / ENSG00000034693
PEX6 / Q13608 / ENSSSCG00000001653 / ENSG00000124587
PFDN1 / O60925 / ENSSSCG00000038493 / ENSG00000113068
PFDN2 / Q9UHV9 / ENSSSCG00000055102 / ENSG00000143256
PFDN5 / Q99471 / ENSSSCG00000000267 / ENSG00000123349
PFKL / P17858 / ENSSSCG00000028197 / ENSG00000141959
PFKM / P08237 / ENSSSCG00000021129 / ENSG00000152556
PFKP / Q01813 / ENSSSCG00000011158 / ENSG00000067057
PFN2 / P35080 / ENSSSCG00000030260 / ENSG00000070087
PGAM2 / P15259 / ENSSSCG00000016720 / ENSG00000164708
PGAM4 / Q8N0Y7 / ENSSSCG00000010517 / ENSG00000226784
PGAM5 / Q96HS1 / ENSSSCG00000035618 / ENSG00000247077
PGD / P52209 / ENSSSCG00000003402 / ENSG00000142657
PGK1 / P00558 / ENSSSCG00000012440 / ENSG00000102144
PGLS / O95336 / ENSSSCG00000013878 / ENSG00000130313
PGLYRP1 / O75594 / ENSSSCG00000038688 / ENSG00000008438
Homologs
Adipose.Tissue
Brain
Colon
Heart
Kidney
Liver
Lung
Pancreas
S.Intestine
Spleen
Organs
Species
Human
Pig
Bins
1
2
3
4
5

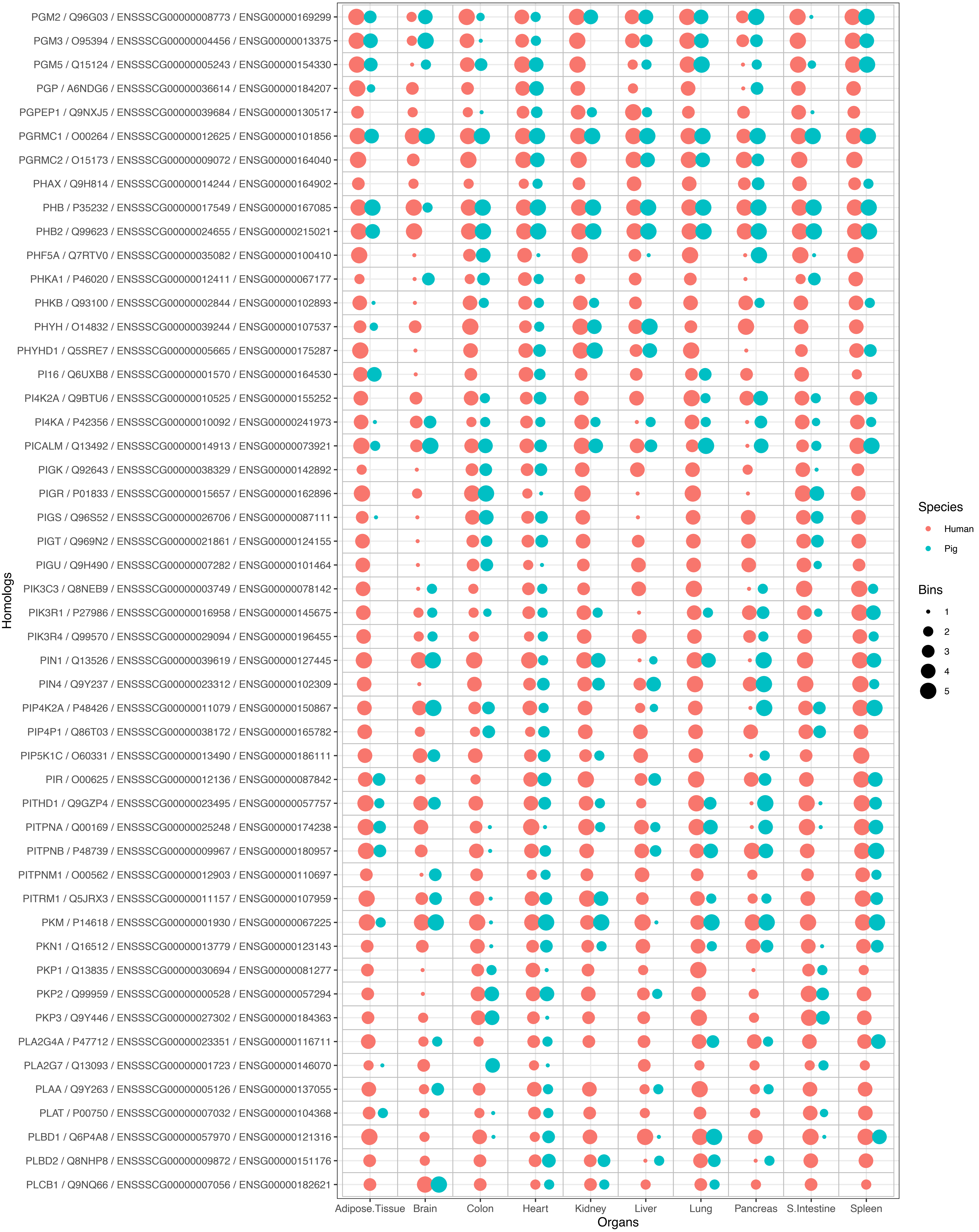
PGM2 / Q96G03 / ENSSSCG00000008773 / ENSG00000169299
PGM3 / O95394 / ENSSSCG00000004456 / ENSG00000013375
PGM5 / Q15124 / ENSSSCG00000005243 / ENSG00000154330
PGP / A6NDG6 / ENSSSCG00000036614 / ENSG00000184207
PGPEP1 / Q9NXJ5 / ENSSSCG00000039684 / ENSG00000130517
PGRMC1 / O00264 / ENSSSCG00000012625 / ENSG00000101856
PGRMC2 / O15173 / ENSSSCG00000009072 / ENSG00000164040
PHAX / Q9H814 / ENSSSCG00000014244 / ENSG00000164902
PHB / P35232 / ENSSSCG00000017549 / ENSG00000167085
PHB2 / Q99623 / ENSSSCG00000024655 / ENSG00000215021
PHF5A / Q7RTV0 / ENSSSCG00000035082 / ENSG00000100410
PHKA1 / P46020 / ENSSSCG00000012411 / ENSG00000067177
PHKB / Q93100 / ENSSSCG00000002844 / ENSG00000102893
PHYH / O14832 / ENSSSCG00000039244 / ENSG00000107537
PHYHD1 / Q5SRE7 / ENSSSCG00000005665 / ENSG00000175287
PI16 / Q6UXB8 / ENSSSCG00000001570 / ENSG00000164530
PI4K2A / Q9BTU6 / ENSSSCG00000010525 / ENSG00000155252
PI4KA / P42356 / ENSSSCG00000010092 / ENSG00000241973
PICALM / Q13492 / ENSSSCG00000014913 / ENSG00000073921
PIGK / Q92643 / ENSSSCG00000038329 / ENSG00000142892
PIGR / P01833 / ENSSSCG00000015657 / ENSG00000162896
PIGS / Q96S52 / ENSSSCG00000026706 / ENSG00000087111
PIGT / Q969N2 / ENSSSCG00000021861 / ENSG00000124155
PIGU / Q9H490 / ENSSSCG00000007282 / ENSG00000101464
PIK3C3 / Q8NEB9 / ENSSSCG00000003749 / ENSG00000078142
PIK3R1 / P27986 / ENSSSCG00000016958 / ENSG00000145675
PIK3R4 / Q99570 / ENSSSCG00000029094 / ENSG00000196455
PIN1 / Q13526 / ENSSSCG00000039619 / ENSG00000127445
PIN4 / Q9Y237 / ENSSSCG00000023312 / ENSG00000102309
PIP4K2A / P48426 / ENSSSCG00000011079 / ENSG00000150867
PIP4P1 / Q86T03 / ENSSSCG00000038172 / ENSG00000165782
PIP5K1C / O60331 / ENSSSCG00000013490 / ENSG00000186111
PIR / O00625 / ENSSSCG00000012136 / ENSG00000087842
PITHD1 / Q9GZP4 / ENSSSCG00000023495 / ENSG00000057757
PITPNA / Q00169 / ENSSSCG00000025248 / ENSG00000174238
PITPNB / P48739 / ENSSSCG00000009967 / ENSG00000180957
PITPNM1 / O00562 / ENSSSCG00000012903 / ENSG00000110697
PITRM1 / Q5JRX3 / ENSSSCG00000011157 / ENSG00000107959
PKM / P14618 / ENSSSCG00000001930 / ENSG00000067225
PKN1 / Q16512 / ENSSSCG00000013779 / ENSG00000123143
PKP1 / Q13835 / ENSSSCG00000030694 / ENSG00000081277
PKP2 / Q99959 / ENSSSCG00000000528 / ENSG00000057294
PKP3 / Q9Y446 / ENSSSCG00000027302 / ENSG00000184363
PLA2G4A / P47712 / ENSSSCG00000023351 / ENSG00000116711
PLA2G7 / Q13093 / ENSSSCG00000001723 / ENSG00000146070
PLAA / Q9Y263 / ENSSSCG00000005126 / ENSG00000137055
PLAT / P00750 / ENSSSCG00000007032 / ENSG00000104368
PLBD1 / Q6P4A8 / ENSSSCG00000057970 / ENSG00000121316
PLBD2 / Q8NHP8 / ENSSSCG00000009872 / ENSG00000151176
PLCB1 / Q9NQ66 / ENSSSCG00000007056 / ENSG00000182621
Homologs
Adipose.Tissue
Brain
Colon
Heart
Kidney
Liver
Lung
Pancreas
S.Intestine
Spleen
Organs
Species
Human
Pig
Bins
1
2
3
4
5

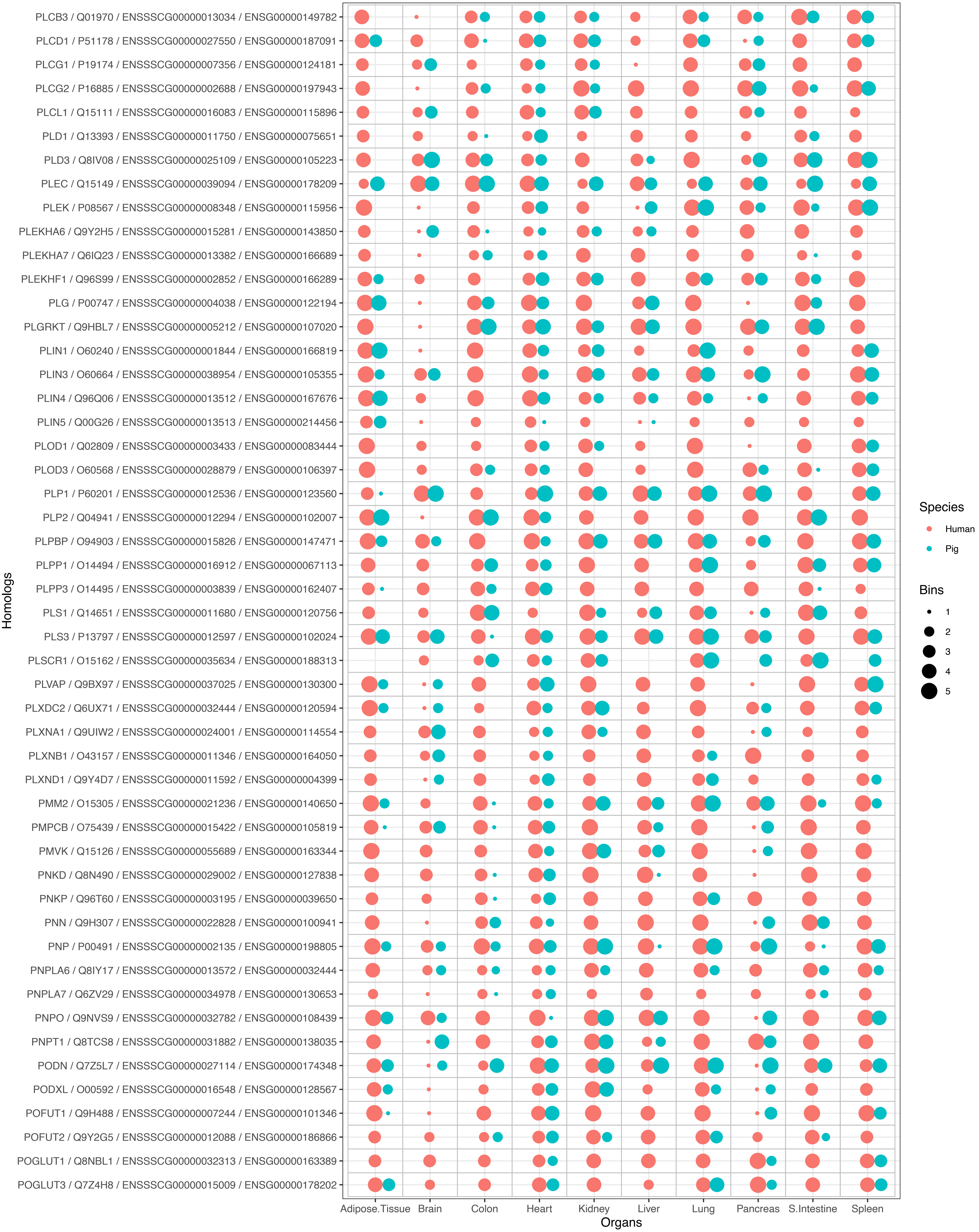
PLCB3 / Q01970 / ENSSSCG00000013034 / ENSG00000149782
PLCD1 / P51178 / ENSSSCG00000027550 / ENSG00000187091
PLCG1 / P19174 / ENSSSCG00000007356 / ENSG00000124181
PLCG2 / P16885 / ENSSSCG00000002688 / ENSG00000197943
PLCL1 / Q15111 / ENSSSCG00000016083 / ENSG00000115896
PLD1 / Q13393 / ENSSSCG00000011750 / ENSG00000075651
PLD3 / Q8IV08 / ENSSSCG00000025109 / ENSG00000105223
PLEC / Q15149 / ENSSSCG00000039094 / ENSG00000178209
PLEK / P08567 / ENSSSCG00000008348 / ENSG00000115956
PLEKHA6 / Q9Y2H5 / ENSSSCG00000015281 / ENSG00000143850
PLEKHA7 / Q6IQ23 / ENSSSCG00000013382 / ENSG00000166689
PLEKHF1 / Q96S99 / ENSSSCG00000002852 / ENSG00000166289
PLG / P00747 / ENSSSCG00000004038 / ENSG00000122194
PLGRKT / Q9HBL7 / ENSSSCG00000005212 / ENSG00000107020
PLIN1 / O60240 / ENSSSCG00000001844 / ENSG00000166819
PLIN3 / O60664 / ENSSSCG00000038954 / ENSG00000105355
PLIN4 / Q96Q06 / ENSSSCG00000013512 / ENSG00000167676
PLIN5 / Q00G26 / ENSSSCG00000013513 / ENSG00000214456
PLOD1 / Q02809 / ENSSSCG00000003433 / ENSG00000083444
PLOD3 / O60568 / ENSSSCG00000028879 / ENSG00000106397
PLP1 / P60201 / ENSSSCG00000012536 / ENSG00000123560
PLP2 / Q04941 / ENSSSCG00000012294 / ENSG00000102007
PLPBP / O94903 / ENSSSCG00000015826 / ENSG00000147471
PLPP1 / O14494 / ENSSSCG00000016912 / ENSG00000067113
PLPP3 / O14495 / ENSSSCG00000003839 / ENSG00000162407
PLS1 / Q14651 / ENSSSCG00000011680 / ENSG00000120756
PLS3 / P13797 / ENSSSCG00000012597 / ENSG00000102024
PLSCR1 / O15162 / ENSSSCG00000035634 / ENSG00000188313
PLVAP / Q9BX97 / ENSSSCG00000037025 / ENSG00000130300
PLXDC2 / Q6UX71 / ENSSSCG00000032444 / ENSG00000120594
PLXNA1 / Q9UIW2 / ENSSSCG00000024001 / ENSG00000114554
PLXNB1 / O43157 / ENSSSCG00000011346 / ENSG00000164050
PLXND1 / Q9Y4D7 / ENSSSCG00000011592 / ENSG00000004399
PMM2 / O15305 / ENSSSCG00000021236 / ENSG00000140650
PMPCB / O75439 / ENSSSCG00000015422 / ENSG00000105819
PMVK / Q15126 / ENSSSCG00000055689 / ENSG00000163344
PNKD / Q8N490 / ENSSSCG00000029002 / ENSG00000127838
PNKP / Q96T60 / ENSSSCG00000003195 / ENSG00000039650
PNN / Q9H307 / ENSSSCG00000022828 / ENSG00000100941
PNP / P00491 / ENSSSCG00000002135 / ENSG00000198805
PNPLA6 / Q8IY17 / ENSSSCG00000013572 / ENSG00000032444
PNPLA7 / Q6ZV29 / ENSSSCG00000034978 / ENSG00000130653
PNPO / Q9NVS9 / ENSSSCG00000032782 / ENSG00000108439
PNPT1 / Q8TCS8 / ENSSSCG00000031882 / ENSG00000138035
PODN / Q7Z5L7 / ENSSSCG00000027114 / ENSG00000174348
PODXL / O00592 / ENSSSCG00000016548 / ENSG00000128567
POFUT1 / Q9H488 / ENSSSCG00000007244 / ENSG00000101346
POFUT2 / Q9Y2G5 / ENSSSCG00000012088 / ENSG00000186866
POGLUT1 / Q8NBL1 / ENSSSCG00000032313 / ENSG00000163389
POGLUT3 / Q7Z4H8 / ENSSSCG00000015009 / ENSG00000178202
Homologs
Adipose.Tissue
Brain
Colon
Heart
Kidney
Liver
Lung
Pancreas
S.Intestine
Spleen
Organs
Species
Human
Pig
Bins
1
2
3
4
5

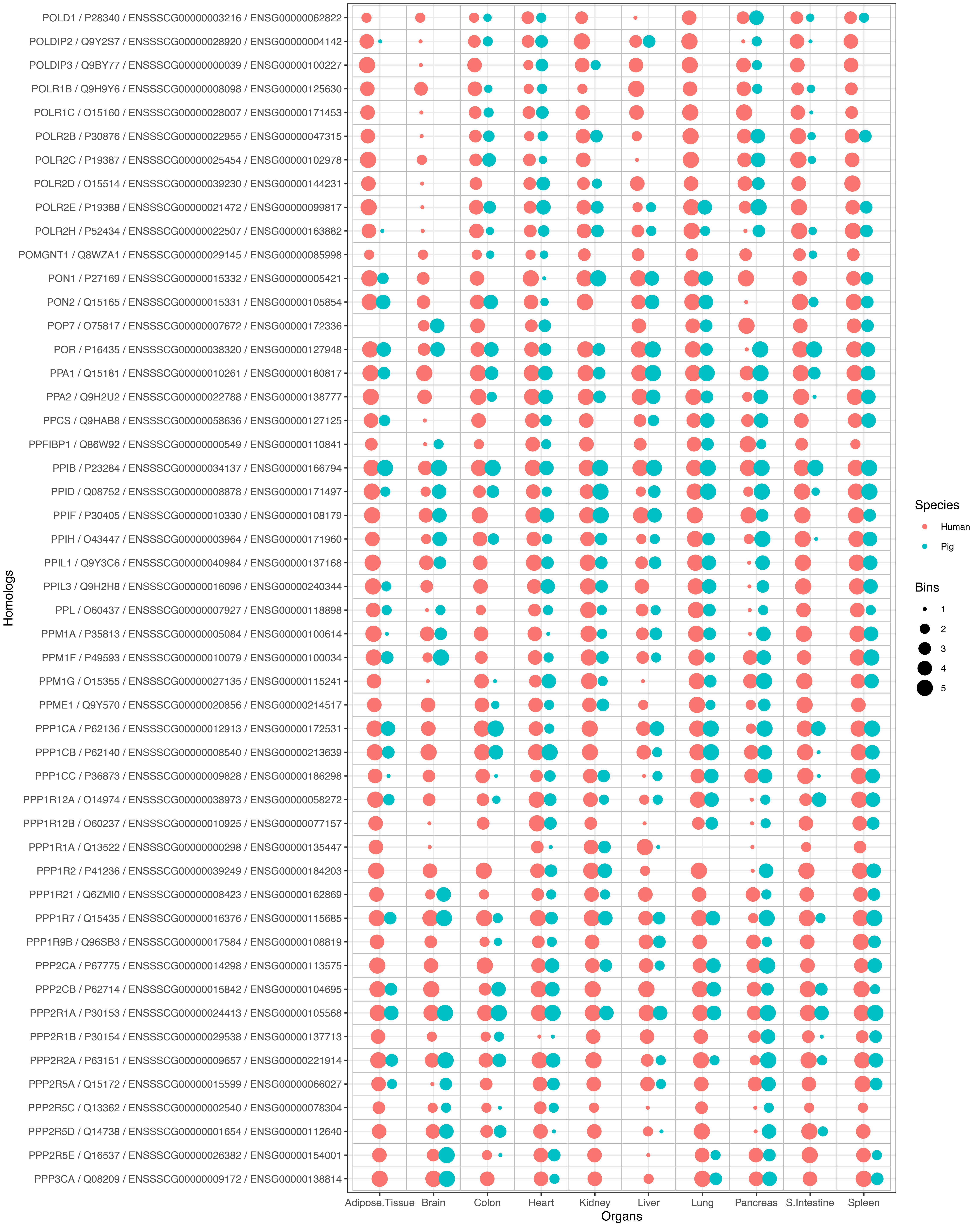
POLD1 / P28340 / ENSSSCG00000003216 / ENSG00000062822
POLDIP2 / Q9Y2S7 / ENSSSCG00000028920 / ENSG00000004142
POLDIP3 / Q9BY77 / ENSSSCG00000000039 / ENSG00000100227
POLR1B / Q9H9Y6 / ENSSSCG00000008098 / ENSG00000125630
POLR1C / O15160 / ENSSSCG00000028007 / ENSG00000171453
POLR2B / P30876 / ENSSSCG00000022955 / ENSG00000047315
POLR2C / P19387 / ENSSSCG00000025454 / ENSG00000102978
POLR2D / O15514 / ENSSSCG00000039230 / ENSG00000144231
POLR2E / P19388 / ENSSSCG00000021472 / ENSG00000099817
POLR2H / P52434 / ENSSSCG00000022507 / ENSG00000163882
POMGNT1 / Q8WZA1 / ENSSSCG00000029145 / ENSG00000085998
PON1 / P27169 / ENSSSCG00000015332 / ENSG00000005421
PON2 / Q15165 / ENSSSCG00000015331 / ENSG00000105854
POP7 / O75817 / ENSSSCG00000007672 / ENSG00000172336
POR / P16435 / ENSSSCG00000038320 / ENSG00000127948
PPA1 / Q15181 / ENSSSCG00000010261 / ENSG00000180817
PPA2 / Q9H2U2 / ENSSSCG00000022788 / ENSG00000138777
PPCS / Q9HAB8 / ENSSSCG00000058636 / ENSG00000127125
PPFIBP1 / Q86W92 / ENSSSCG00000000549 / ENSG00000110841
PPIB / P23284 / ENSSSCG00000034137 / ENSG00000166794
PPID / Q08752 / ENSSSCG00000008878 / ENSG00000171497
PPIF / P30405 / ENSSSCG00000010330 / ENSG00000108179
PPIH / O43447 / ENSSSCG00000003964 / ENSG00000171960
PPIL1 / Q9Y3C6 / ENSSSCG00000040984 / ENSG00000137168
PPIL3 / Q9H2H8 / ENSSSCG00000016096 / ENSG00000240344
PPL / O60437 / ENSSSCG00000007927 / ENSG00000118898
PPM1A / P35813 / ENSSSCG00000005084 / ENSG00000100614
PPM1F / P49593 / ENSSSCG00000010079 / ENSG00000100034
PPM1G / O15355 / ENSSSCG00000027135 / ENSG00000115241
PPME1 / Q9Y570 / ENSSSCG00000020856 / ENSG00000214517
PPP1CA / P62136 / ENSSSCG00000012913 / ENSG00000172531
PPP1CB / P62140 / ENSSSCG00000008540 / ENSG00000213639
PPP1CC / P36873 / ENSSSCG00000009828 / ENSG00000186298
PPP1R12A / O14974 / ENSSSCG00000038973 / ENSG00000058272
PPP1R12B / O60237 / ENSSSCG00000010925 / ENSG00000077157
PPP1R1A / Q13522 / ENSSSCG00000000298 / ENSG00000135447
PPP1R2 / P41236 / ENSSSCG00000039249 / ENSG00000184203
PPP1R21 / Q6ZMI0 / ENSSSCG00000008423 / ENSG00000162869
PPP1R7 / Q15435 / ENSSSCG00000016376 / ENSG00000115685
PPP1R9B / Q96SB3 / ENSSSCG00000017584 / ENSG00000108819
PPP2CA / P67775 / ENSSSCG00000014298 / ENSG00000113575
PPP2CB / P62714 / ENSSSCG00000015842 / ENSG00000104695
PPP2R1A / P30153 / ENSSSCG00000024413 / ENSG00000105568
PPP2R1B / P30154 / ENSSSCG00000029538 / ENSG00000137713
PPP2R2A / P63151 / ENSSSCG00000009657 / ENSG00000221914
PPP2R5A / Q15172 / ENSSSCG00000015599 / ENSG00000066027
PPP2R5C / Q13362 / ENSSSCG00000002540 / ENSG00000078304
PPP2R5D / Q14738 / ENSSSCG00000001654 / ENSG00000112640
PPP2R5E / Q16537 / ENSSSCG00000026382 / ENSG00000154001
PPP3CA / Q08209 / ENSSSCG00000009172 / ENSG00000138814
Homologs
Adipose.Tissue
Brain
Colon
Heart
Kidney
Liver
Lung
Pancreas
S.Intestine
Spleen
Organs
Species
Human
Pig
Bins
1
2
3
4
5

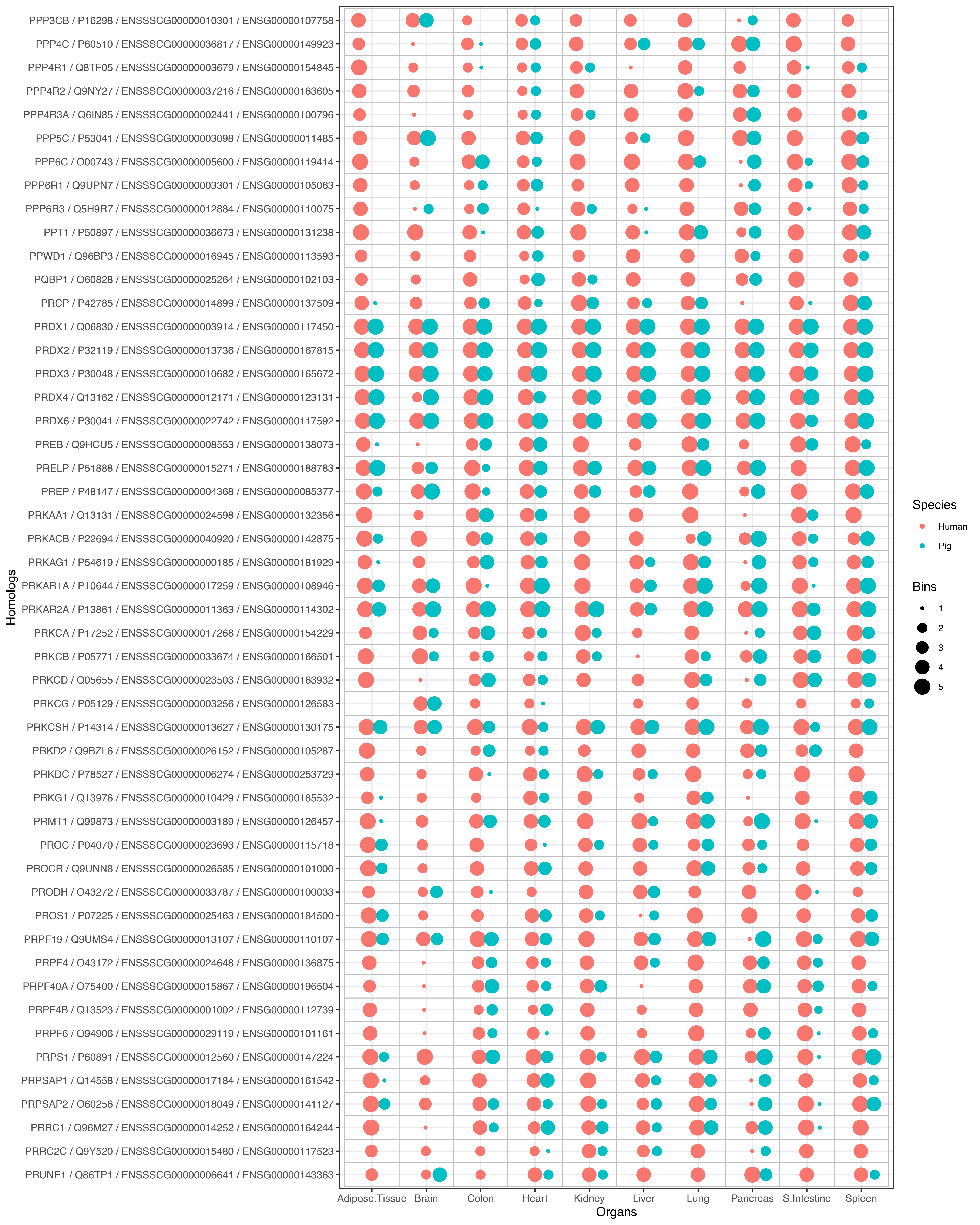

PPP3CB / P16298 / ENSSSCG00000010301 / ENSG00000107758
PPP4C / P60510 / ENSSSCG00000036817 / ENSG00000149923
PPP4R1 / Q8TF05 / ENSSSCG00000003679 / ENSG00000154845
PPP4R2 / Q9NY27 / ENSSSCG00000037216 / ENSG00000163605
PPP4R3A / Q6IN85 / ENSSSCG00000002441 / ENSG00000100796
PPP5C / P53041 / ENSSSCG00000003098 / ENSG00000011485
PPP6C / O00743 / ENSSSCG00000005600 / ENSG00000119414
PPP6R1 / Q9UPN7 / ENSSSCG00000003301 / ENSG00000105063
PPP6R3 / Q5H9R7 / ENSSSCG00000012884 / ENSG00000110075
PPT1 / P50897 / ENSSSCG00000036673 / ENSG00000131238
PPWD1 / Q96BP3 / ENSSSCG00000016945 / ENSG00000113593
PQBP1 / O60828 / ENSSSCG00000025264 / ENSG00000102103
PRCP / P42785 / ENSSSCG00000014899 / ENSG00000137509
PRDX1 / Q06830 / ENSSSCG00000003914 / ENSG00000117450
PRDX2 / P32119 / ENSSSCG00000013736 / ENSG00000167815
PRDX3 / P30048 / ENSSSCG00000010682 / ENSG00000165672
PRDX4 / Q13162 / ENSSSCG00000012171 / ENSG00000123131
PRDX6 / P30041 / ENSSSCG00000022742 / ENSG00000117592
PREB / Q9HCU5 / ENSSSCG00000008553 / ENSG00000138073
PRELP / P51888 / ENSSSCG00000015271 / ENSG00000188783
PREP / P48147 / ENSSSCG00000004368 / ENSG00000085377
PRKAA1 / Q13131 / ENSSSCG00000024598 / ENSG00000132356
PRKACB / P22694 / ENSSSCG00000040920 / ENSG00000142875
PRKAG1 / P54619 / ENSSSCG00000000185 / ENSG00000181929
PRKAR1A / P10644 / ENSSSCG00000017259 / ENSG00000108946
PRKAR2A / P13861 / ENSSSCG00000011363 / ENSG00000114302
PRKCA / P17252 / ENSSSCG00000017268 / ENSG00000154229
PRKCB / P05771 / ENSSSCG00000033674 / ENSG00000166501
PRKCD / Q05655 / ENSSSCG00000023503 / ENSG00000163932
PRKCG / P05129 / ENSSSCG00000003256 / ENSG00000126583
PRKCSH / P14314 / ENSSSCG00000013627 / ENSG00000130175
PRKD2 / Q9BZL6 / ENSSSCG00000026152 / ENSG00000105287
PRKDC / P78527 / ENSSSCG00000006274 / ENSG00000253729
PRKG1 / Q13976 / ENSSSCG00000010429 / ENSG00000185532
PRMT1 / Q99873 / ENSSSCG00000003189 / ENSG00000126457
PROC / P04070 / ENSSSCG00000023693 / ENSG00000115718
PROCR / Q9UNN8 / ENSSSCG00000026585 / ENSG00000101000
PRODH / O43272 / ENSSSCG00000033787 / ENSG00000100033
PROS1 / P07225 / ENSSSCG00000025463 / ENSG00000184500
PRPF19 / Q9UMS4 / ENSSSCG00000013107 / ENSG00000110107
PRPF4 / O43172 / ENSSSCG00000024648 / ENSG00000136875
PRPF40A / O75400 / ENSSSCG00000015867 / ENSG00000196504
PRPF4B / Q13523 / ENSSSCG00000001002 / ENSG00000112739
PRPF6 / O94906 / ENSSSCG00000029119 / ENSG00000101161
PRPS1 / P60891 / ENSSSCG00000012560 / ENSG00000147224
PRPSAP1 / Q14558 / ENSSSCG00000017184 / ENSG00000161542
PRPSAP2 / O60256 / ENSSSCG00000018049 / ENSG00000141127
PRRC1 / Q96M27 / ENSSSCG00000014252 / ENSG00000164244
PRRC2C / Q9Y520 / ENSSSCG00000015480 / ENSG00000117523
PRUNE1 / Q86TP1 / ENSSSCG00000006641 / ENSG00000143363
Homologs
Adipose.Tissue
Brain
Colon
Heart
Kidney
Liver
Lung
Pancreas
S.Intestine
Spleen
Organs
Species
Human
Pig
Bins
1
2
3
4
5

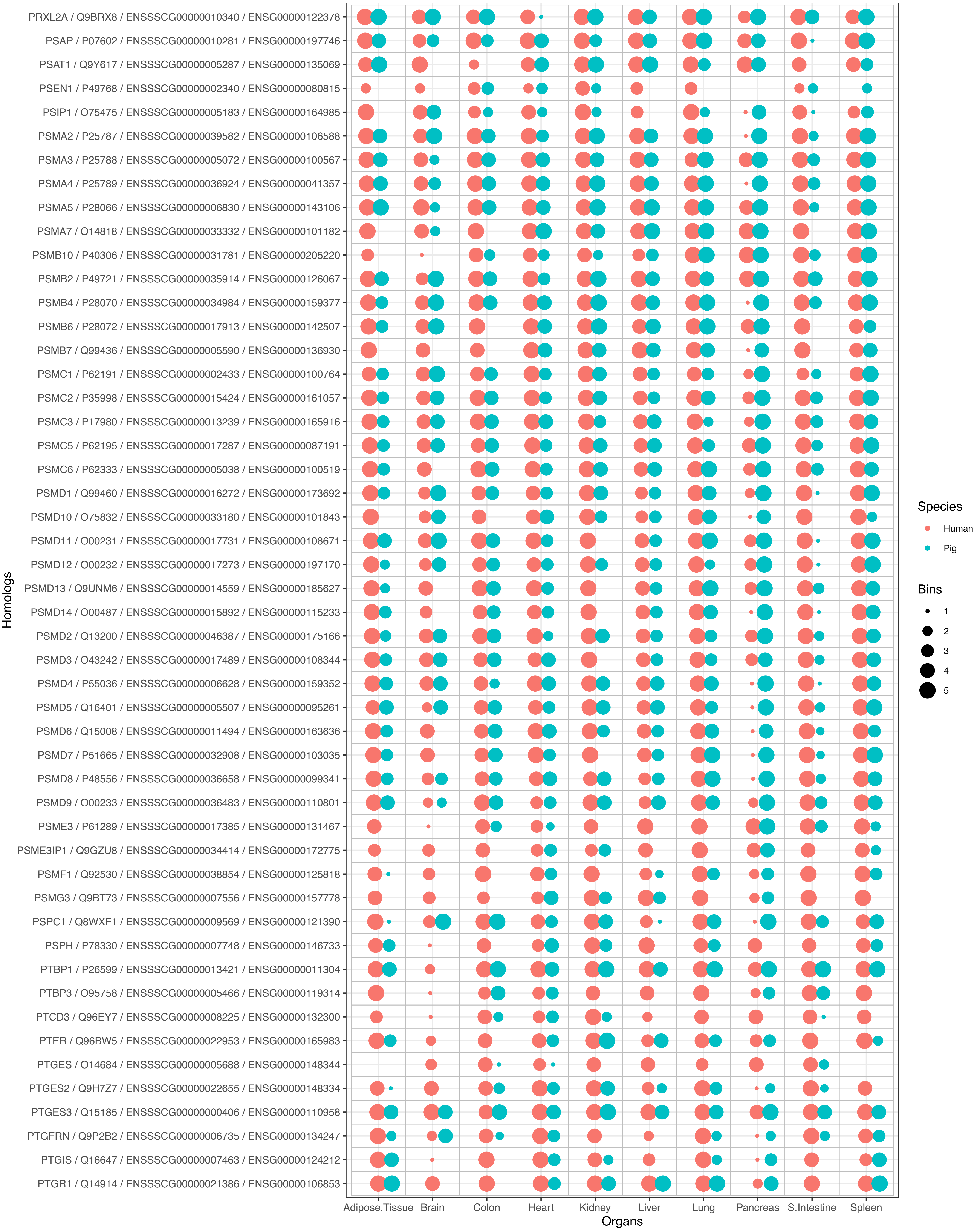
PRXL2A / Q9BRX8 / ENSSSCG00000010340 / ENSG00000122378
PSAP / P07602 / ENSSSCG00000010281 / ENSG00000197746
PSAT1 / Q9Y617 / ENSSSCG00000005287 / ENSG00000135069
PSEN1 / P49768 / ENSSSCG00000002340 / ENSG00000080815
PSIP1 / O75475 / ENSSSCG00000005183 / ENSG00000164985
PSMA2 / P25787 / ENSSSCG00000039582 / ENSG00000106588
PSMA3 / P25788 / ENSSSCG00000005072 / ENSG00000100567
PSMA4 / P25789 / ENSSSCG00000036924 / ENSG00000041357
PSMA5 / P28066 / ENSSSCG00000006830 / ENSG00000143106
PSMA7 / O14818 / ENSSSCG00000033332 / ENSG00000101182
PSMB10 / P40306 / ENSSSCG00000031781 / ENSG00000205220
PSMB2 / P49721 / ENSSSCG00000035914 / ENSG00000126067
PSMB4 / P28070 / ENSSSCG00000034984 / ENSG00000159377
PSMB6 / P28072 / ENSSSCG00000017913 / ENSG00000142507
PSMB7 / Q99436 / ENSSSCG00000005590 / ENSG00000136930
PSMC1 / P62191 / ENSSSCG00000002433 / ENSG00000100764
PSMC2 / P35998 / ENSSSCG00000015424 / ENSG00000161057
PSMC3 / P17980 / ENSSSCG00000013239 / ENSG00000165916
PSMC5 / P62195 / ENSSSCG00000017287 / ENSG00000087191
PSMC6 / P62333 / ENSSSCG00000005038 / ENSG00000100519
PSMD1 / Q99460 / ENSSSCG00000016272 / ENSG00000173692
PSMD10 / O75832 / ENSSSCG00000033180 / ENSG00000101843
PSMD11 / O00231 / ENSSSCG00000017731 / ENSG00000108671
PSMD12 / O00232 / ENSSSCG00000017273 / ENSG00000197170
PSMD13 / Q9UNM6 / ENSSSCG00000014559 / ENSG00000185627
PSMD14 / O00487 / ENSSSCG00000015892 / ENSG00000115233
PSMD2 / Q13200 / ENSSSCG00000046387 / ENSG00000175166
PSMD3 / O43242 / ENSSSCG00000017489 / ENSG00000108344
PSMD4 / P55036 / ENSSSCG00000006628 / ENSG00000159352
PSMD5 / Q16401 / ENSSSCG00000005507 / ENSG00000095261
PSMD6 / Q15008 / ENSSSCG00000011494 / ENSG00000163636
PSMD7 / P51665 / ENSSSCG00000032908 / ENSG00000103035
PSMD8 / P48556 / ENSSSCG00000036658 / ENSG00000099341
PSMD9 / O00233 / ENSSSCG00000036483 / ENSG00000110801
PSME3 / P61289 / ENSSSCG00000017385 / ENSG00000131467
PSME3IP1 / Q9GZU8 / ENSSSCG00000034414 / ENSG00000172775
PSMF1 / Q92530 / ENSSSCG00000038854 / ENSG00000125818
PSMG3 / Q9BT73 / ENSSSCG00000007556 / ENSG00000157778
PSPC1 / Q8WXF1 / ENSSSCG00000009569 / ENSG00000121390
PSPH / P78330 / ENSSSCG00000007748 / ENSG00000146733
PTBP1 / P26599 / ENSSSCG00000013421 / ENSG00000011304
PTBP3 / O95758 / ENSSSCG00000005466 / ENSG00000119314
PTCD3 / Q96EY7 / ENSSSCG00000008225 / ENSG00000132300
PTER / Q96BW5 / ENSSSCG00000022953 / ENSG00000165983
PTGES / O14684 / ENSSSCG00000005688 / ENSG00000148344
PTGES2 / Q9H7Z7 / ENSSSCG00000022655 / ENSG00000148334
PTGES3 / Q15185 / ENSSSCG00000000406 / ENSG00000110958
PTGFRN / Q9P2B2 / ENSSSCG00000006735 / ENSG00000134247
PTGIS / Q16647 / ENSSSCG00000007463 / ENSG00000124212
PTGR1 / Q14914 / ENSSSCG00000021386 / ENSG00000106853
Homologs
Adipose.Tissue
Brain
Colon
Heart
Kidney
Liver
Lung
Pancreas
S.Intestine
Spleen
Organs
Species
Human
Pig
Bins
1
2
3
4
5

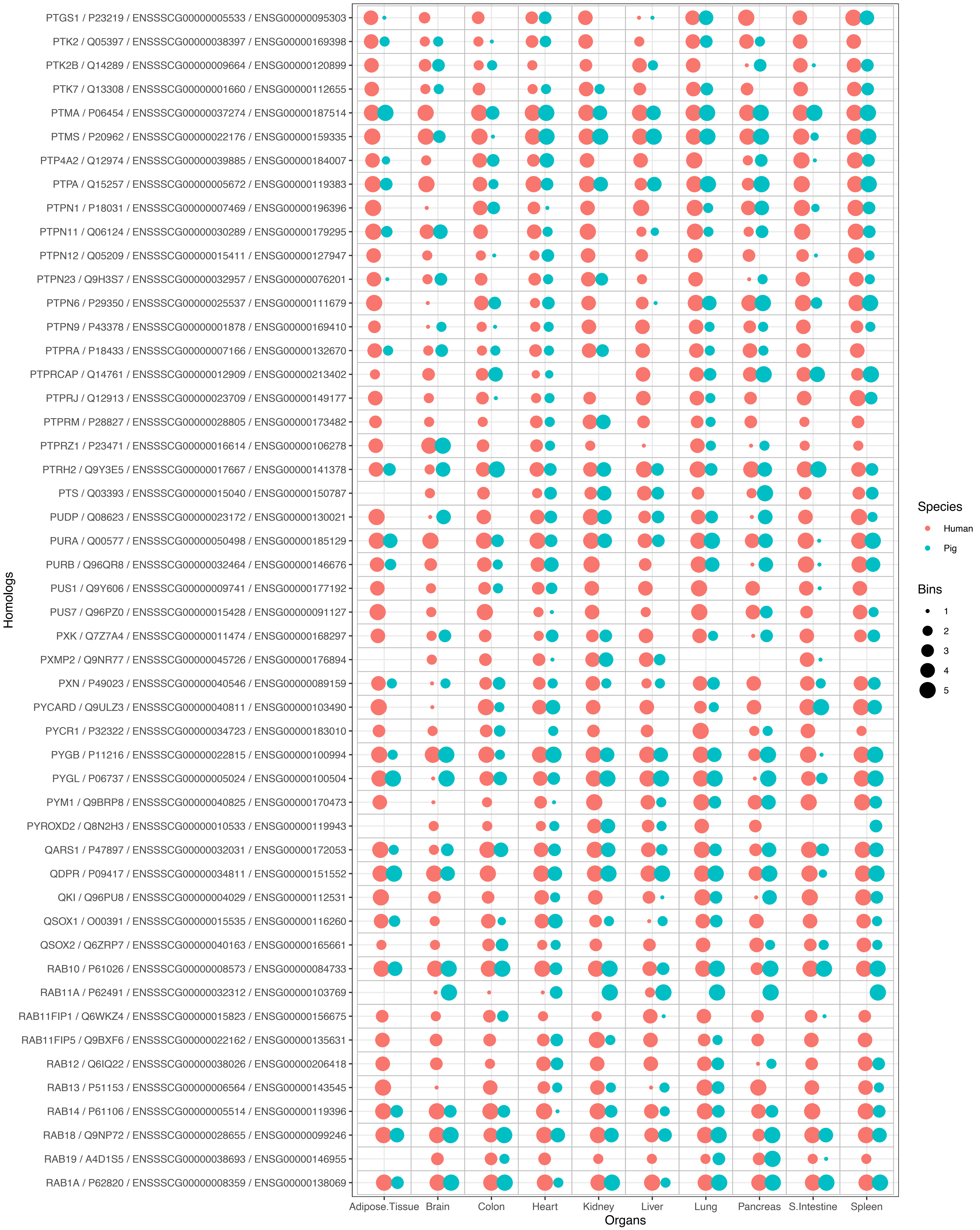
PTGS1 / P23219 / ENSSSCG00000005533 / ENSG00000095303
PTK2 / Q05397 / ENSSSCG00000038397 / ENSG00000169398
PTK2B / Q14289 / ENSSSCG00000009664 / ENSG00000120899
PTK7 / Q13308 / ENSSSCG00000001660 / ENSG00000112655
PTMA / P06454 / ENSSSCG00000037274 / ENSG00000187514
PTMS / P20962 / ENSSSCG00000022176 / ENSG00000159335
PTP4A2 / Q12974 / ENSSSCG00000039885 / ENSG00000184007
PTPA / Q15257 / ENSSSCG00000005672 / ENSG00000119383
PTPN1 / P18031 / ENSSSCG00000007469 / ENSG00000196396
PTPN11 / Q06124 / ENSSSCG00000030289 / ENSG00000179295
PTPN12 / Q05209 / ENSSSCG00000015411 / ENSG00000127947
PTPN23 / Q9H3S7 / ENSSSCG00000032957 / ENSG00000076201
PTPN6 / P29350 / ENSSSCG00000025537 / ENSG00000111679
PTPN9 / P43378 / ENSSSCG00000001878 / ENSG00000169410
PTPRA / P18433 / ENSSSCG00000007166 / ENSG00000132670
PTPRCAP / Q14761 / ENSSSCG00000012909 / ENSG00000213402
PTPRJ / Q12913 / ENSSSCG00000023709 / ENSG00000149177
PTPRM / P28827 / ENSSSCG00000028805 / ENSG00000173482
PTPRZ1 / P23471 / ENSSSCG00000016614 / ENSG00000106278
PTRH2 / Q9Y3E5 / ENSSSCG00000017667 / ENSG00000141378
PTS / Q03393 / ENSSSCG00000015040 / ENSG00000150787
PUDP / Q08623 / ENSSSCG00000023172 / ENSG00000130021
PURA / Q00577 / ENSSSCG00000050498 / ENSG00000185129
PURB / Q96QR8 / ENSSSCG00000032464 / ENSG00000146676
PUS1 / Q9Y606 / ENSSSCG00000009741 / ENSG00000177192
PUS7 / Q96PZ0 / ENSSSCG00000015428 / ENSG00000091127
PXK / Q7Z7A4 / ENSSSCG00000011474 / ENSG00000168297
PXMP2 / Q9NR77 / ENSSSCG00000045726 / ENSG00000176894
PXN / P49023 / ENSSSCG00000040546 / ENSG00000089159
PYCARD / Q9ULZ3 / ENSSSCG00000040811 / ENSG00000103490
PYCR1 / P32322 / ENSSSCG00000034723 / ENSG00000183010
PYGB / P11216 / ENSSSCG00000022815 / ENSG00000100994
PYGL / P06737 / ENSSSCG00000005024 / ENSG00000100504
PYM1 / Q9BRP8 / ENSSSCG00000040825 / ENSG00000170473
PYROXD2 / Q8N2H3 / ENSSSCG00000010533 / ENSG00000119943
QARS1 / P47897 / ENSSSCG00000032031 / ENSG00000172053
QDPR / P09417 / ENSSSCG00000034811 / ENSG00000151552
QKI / Q96PU8 / ENSSSCG00000004029 / ENSG00000112531
QSOX1 / O00391 / ENSSSCG00000015535 / ENSG00000116260
QSOX2 / Q6ZRP7 / ENSSSCG00000040163 / ENSG00000165661
RAB10 / P61026 / ENSSSCG00000008573 / ENSG00000084733
RAB11A / P62491 / ENSSSCG00000032312 / ENSG00000103769
RAB11FIP1 / Q6WKZ4 / ENSSSCG00000015823 / ENSG00000156675
RAB11FIP5 / Q9BXF6 / ENSSSCG00000022162 / ENSG00000135631
RAB12 / Q6IQ22 / ENSSSCG00000038026 / ENSG00000206418
RAB13 / P51153 / ENSSSCG00000006564 / ENSG00000143545
RAB14 / P61106 / ENSSSCG00000005514 / ENSG00000119396
RAB18 / Q9NP72 / ENSSSCG00000028655 / ENSG00000099246
RAB19 / A4D1S5 / ENSSSCG00000038693 / ENSG00000146955
RAB1A / P62820 / ENSSSCG00000008359 / ENSG00000138069
Homologs
Adipose.Tissue
Brain
Colon
Heart
Kidney
Liver
Lung
Pancreas
S.Intestine
Spleen
Organs
Species
Human
Pig
Bins
1
2
3
4
5

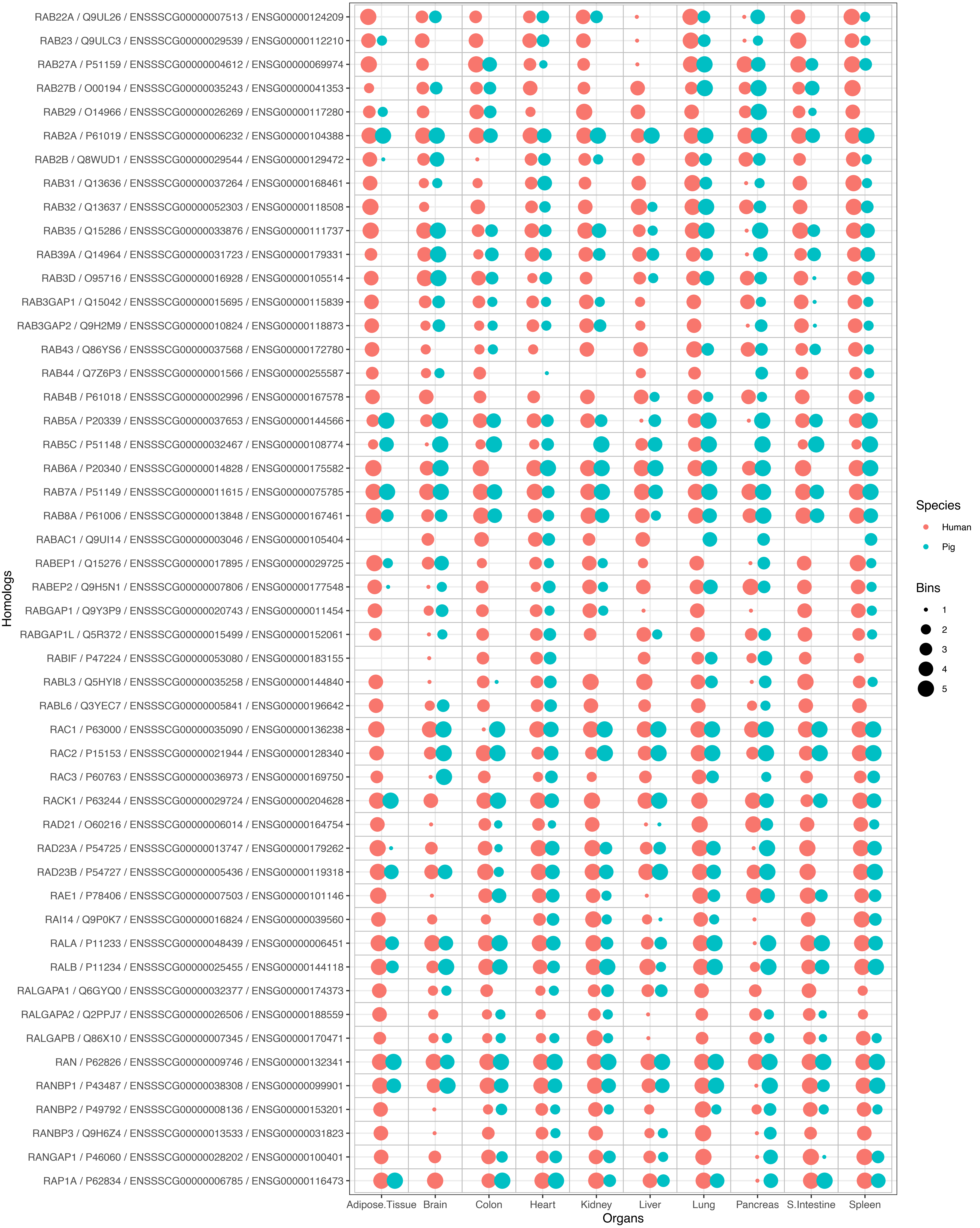
RAB22A / Q9UL26 / ENSSSCG00000007513 / ENSG00000124209
RAB23 / Q9ULC3 / ENSSSCG00000029539 / ENSG00000112210
RAB27A / P51159 / ENSSSCG00000004612 / ENSG00000069974
RAB27B / O00194 / ENSSSCG00000035243 / ENSG00000041353
RAB29 / O14966 / ENSSSCG00000026269 / ENSG00000117280
RAB2A / P61019 / ENSSSCG00000006232 / ENSG00000104388
RAB2B / Q8WUD1 / ENSSSCG00000029544 / ENSG00000129472
RAB31 / Q13636 / ENSSSCG00000037264 / ENSG00000168461
RAB32 / Q13637 / ENSSSCG00000052303 / ENSG00000118508
RAB35 / Q15286 / ENSSSCG00000033876 / ENSG00000111737
RAB39A / Q14964 / ENSSSCG00000031723 / ENSG00000179331
RAB3D / O95716 / ENSSSCG00000016928 / ENSG00000105514
RAB3GAP1 / Q15042 / ENSSSCG00000015695 / ENSG00000115839
RAB3GAP2 / Q9H2M9 / ENSSSCG00000010824 / ENSG00000118873
RAB43 / Q86YS6 / ENSSSCG00000037568 / ENSG00000172780
RAB44 / Q7Z6P3 / ENSSSCG00000001566 / ENSG00000255587
RAB4B / P61018 / ENSSSCG00000002996 / ENSG00000167578
RAB5A / P20339 / ENSSSCG00000037653 / ENSG00000144566
RAB5C / P51148 / ENSSSCG00000032467 / ENSG00000108774
RAB6A / P20340 / ENSSSCG00000014828 / ENSG00000175582
RAB7A / P51149 / ENSSSCG00000011615 / ENSG00000075785
RAB8A / P61006 / ENSSSCG00000013848 / ENSG00000167461
RABAC1 / Q9UI14 / ENSSSCG00000003046 / ENSG00000105404
RABEP1 / Q15276 / ENSSSCG00000017895 / ENSG00000029725
RABEP2 / Q9H5N1 / ENSSSCG00000007806 / ENSG00000177548
RABGAP1 / Q9Y3P9 / ENSSSCG00000020743 / ENSG00000011454
RABGAP1L / Q5R372 / ENSSSCG00000015499 / ENSG00000152061
RABIF / P47224 / ENSSSCG00000053080 / ENSG00000183155
RABL3 / Q5HYI8 / ENSSSCG00000035258 / ENSG00000144840
RABL6 / Q3YEC7 / ENSSSCG00000005841 / ENSG00000196642
RAC1 / P63000 / ENSSSCG00000035090 / ENSG00000136238
RAC2 / P15153 / ENSSSCG00000021944 / ENSG00000128340
RAC3 / P60763 / ENSSSCG00000036973 / ENSG00000169750
RACK1 / P63244 / ENSSSCG00000029724 / ENSG00000204628
RAD21 / O60216 / ENSSSCG00000006014 / ENSG00000164754
RAD23A / P54725 / ENSSSCG00000013747 / ENSG00000179262
RAD23B / P54727 / ENSSSCG00000005436 / ENSG00000119318
RAE1 / P78406 / ENSSSCG00000007503 / ENSG00000101146
RAI14 / Q9P0K7 / ENSSSCG00000016824 / ENSG00000039560
RALA / P11233 / ENSSSCG00000048439 / ENSG00000006451
RALB / P11234 / ENSSSCG00000025455 / ENSG00000144118
RALGAPA1 / Q6GYQ0 / ENSSSCG00000032377 / ENSG00000174373
RALGAPA2 / Q2PPJ7 / ENSSSCG00000026506 / ENSG00000188559
RALGAPB / Q86X10 / ENSSSCG00000007345 / ENSG00000170471
RAN / P62826 / ENSSSCG00000009746 / ENSG00000132341
RANBP1 / P43487 / ENSSSCG00000038308 / ENSG00000099901
RANBP2 / P49792 / ENSSSCG00000008136 / ENSG00000153201
RANBP3 / Q9H6Z4 / ENSSSCG00000013533 / ENSG00000031823
RANGAP1 / P46060 / ENSSSCG00000028202 / ENSG00000100401
RAP1A / P62834 / ENSSSCG00000006785 / ENSG00000116473
Homologs
Adipose.Tissue
Brain
Colon
Heart
Kidney
Liver
Lung
Pancreas
S.Intestine
Spleen
Organs
Species
Human
Pig
Bins
1
2
3
4
5

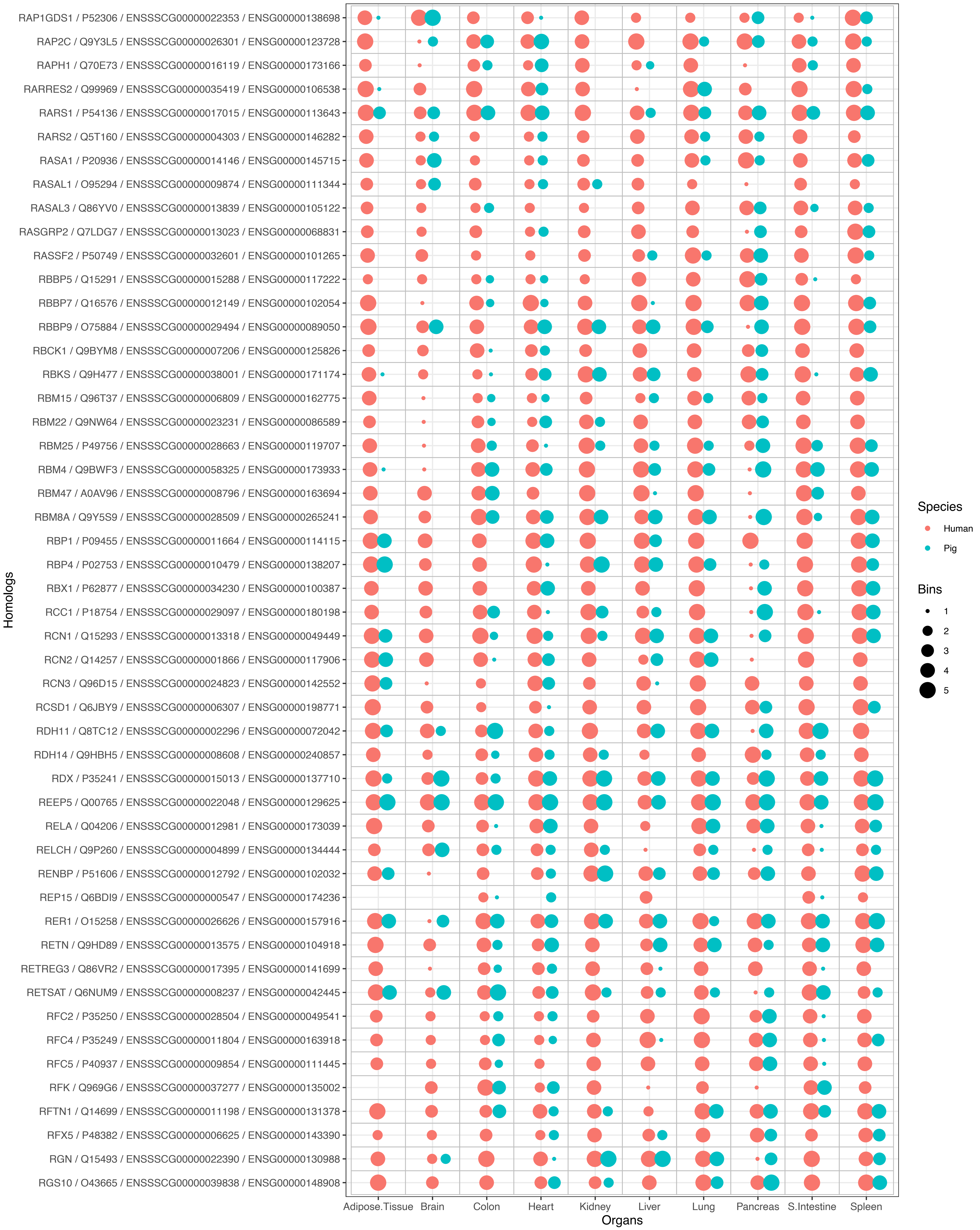
RAP1GDS1 / P52306 / ENSSSCG00000022353 / ENSG00000138698
RAP2C / Q9Y3L5 / ENSSSCG00000026301 / ENSG00000123728
RAPH1 / Q70E73 / ENSSSCG00000016119 / ENSG00000173166
RARRES2 / Q99969 / ENSSSCG00000035419 / ENSG00000106538
RARS1 / P54136 / ENSSSCG00000017015 / ENSG00000113643
RARS2 / Q5T160 / ENSSSCG00000004303 / ENSG00000146282
RASA1 / P20936 / ENSSSCG00000014146 / ENSG00000145715
RASAL1 / O95294 / ENSSSCG00000009874 / ENSG00000111344
RASAL3 / Q86YV0 / ENSSSCG00000013839 / ENSG00000105122
RASGRP2 / Q7LDG7 / ENSSSCG00000013023 / ENSG00000068831
RASSF2 / P50749 / ENSSSCG00000032601 / ENSG00000101265
RBBP5 / Q15291 / ENSSSCG00000015288 / ENSG00000117222
RBBP7 / Q16576 / ENSSSCG00000012149 / ENSG00000102054
RBBP9 / O75884 / ENSSSCG00000029494 / ENSG00000089050
RBCK1 / Q9BYM8 / ENSSSCG00000007206 / ENSG00000125826
RBKS / Q9H477 / ENSSSCG00000038001 / ENSG00000171174
RBM15 / Q96T37 / ENSSSCG00000006809 / ENSG00000162775
RBM22 / Q9NW64 / ENSSSCG00000023231 / ENSG00000086589
RBM25 / P49756 / ENSSSCG00000028663 / ENSG00000119707
RBM4 / Q9BWF3 / ENSSSCG00000058325 / ENSG00000173933
RBM47 / A0AV96 / ENSSSCG00000008796 / ENSG00000163694
RBM8A / Q9Y5S9 / ENSSSCG00000028509 / ENSG00000265241
RBP1 / P09455 / ENSSSCG00000011664 / ENSG00000114115
RBP4 / P02753 / ENSSSCG00000010479 / ENSG00000138207
RBX1 / P62877 / ENSSSCG00000034230 / ENSG00000100387
RCC1 / P18754 / ENSSSCG00000029097 / ENSG00000180198
RCN1 / Q15293 / ENSSSCG00000013318 / ENSG00000049449
RCN2 / Q14257 / ENSSSCG00000001866 / ENSG00000117906
RCN3 / Q96D15 / ENSSSCG00000024823 / ENSG00000142552
RCSD1 / Q6JBY9 / ENSSSCG00000006307 / ENSG00000198771
RDH11 / Q8TC12 / ENSSSCG00000002296 / ENSG00000072042
RDH14 / Q9HBH5 / ENSSSCG00000008608 / ENSG00000240857
RDX / P35241 / ENSSSCG00000015013 / ENSG00000137710
REEP5 / Q00765 / ENSSSCG00000022048 / ENSG00000129625
RELA / Q04206 / ENSSSCG00000012981 / ENSG00000173039
RELCH / Q9P260 / ENSSSCG00000004899 / ENSG00000134444
RENBP / P51606 / ENSSSCG00000012792 / ENSG00000102032
REP15 / Q6BDI9 / ENSSSCG00000000547 / ENSG00000174236
RER1 / O15258 / ENSSSCG00000026626 / ENSG00000157916
RETN / Q9HD89 / ENSSSCG00000013575 / ENSG00000104918
RETREG3 / Q86VR2 / ENSSSCG00000017395 / ENSG00000141699
RETSAT / Q6NUM9 / ENSSSCG00000008237 / ENSG00000042445
RFC2 / P35250 / ENSSSCG00000028504 / ENSG00000049541
RFC4 / P35249 / ENSSSCG00000011804 / ENSG00000163918
RFC5 / P40937 / ENSSSCG00000009854 / ENSG00000111445
RFK / Q969G6 / ENSSSCG00000037277 / ENSG00000135002
RFTN1 / Q14699 / ENSSSCG00000011198 / ENSG00000131378
RFX5 / P48382 / ENSSSCG00000006625 / ENSG00000143390
RGN / Q15493 / ENSSSCG00000022390 / ENSG00000130988
RGS10 / O43665 / ENSSSCG00000039838 / ENSG00000148908
Homologs
Adipose.Tissue
Brain
Colon
Heart
Kidney
Liver
Lung
Pancreas
S.Intestine
Spleen
Organs
Species
Human
Pig
Bins
1
2
3
4
5

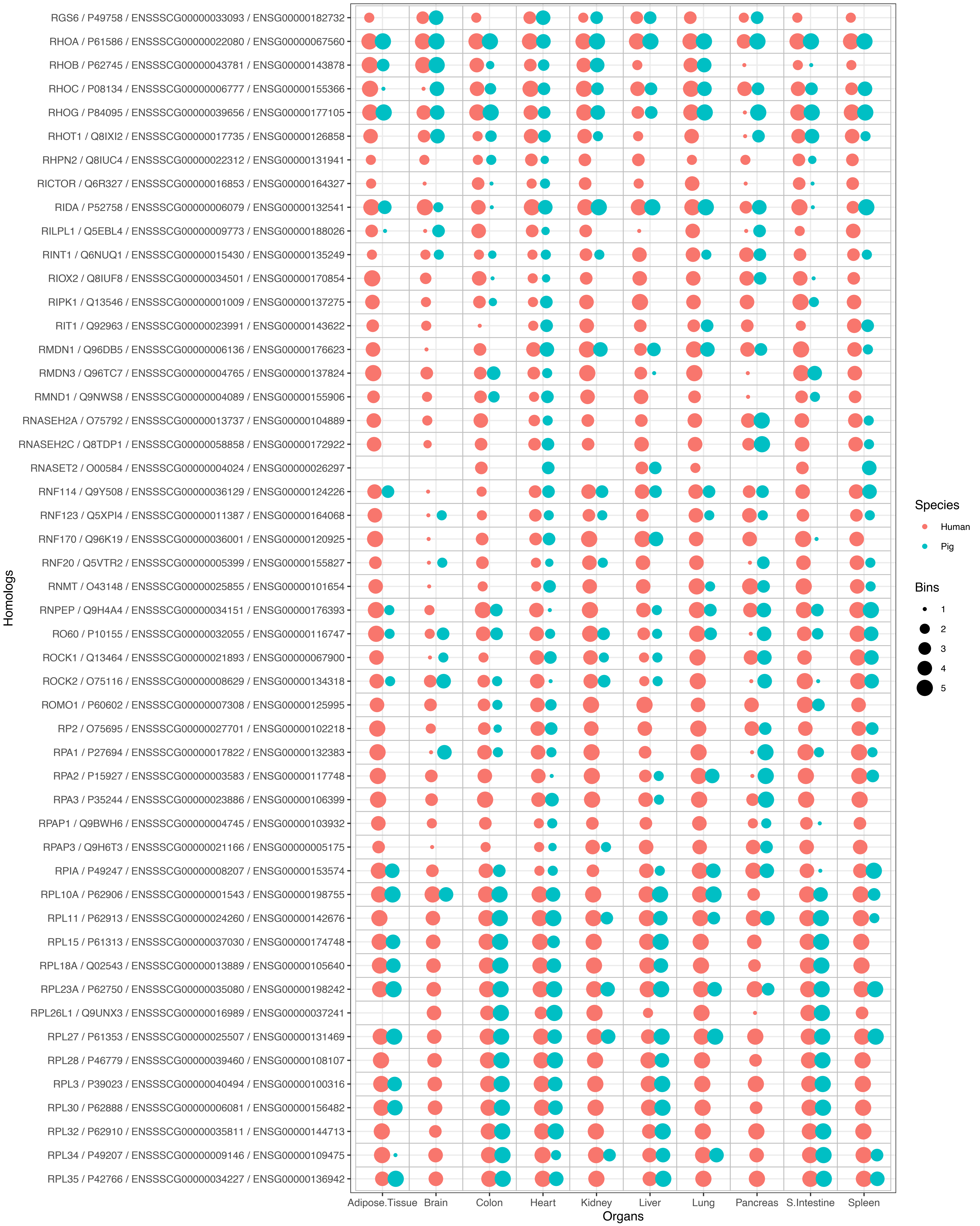
RGS6 / P49758 / ENSSSCG00000033093 / ENSG00000182732
RHOA / P61586 / ENSSSCG00000022080 / ENSG00000067560
RHOB / P62745 / ENSSSCG00000043781 / ENSG00000143878
RHOC / P08134 / ENSSSCG00000006777 / ENSG00000155366
RHOG / P84095 / ENSSSCG00000039656 / ENSG00000177105
RHOT1 / Q8IXI2 / ENSSSCG00000017735 / ENSG00000126858
RHPN2 / Q8IUC4 / ENSSSCG00000022312 / ENSG00000131941
RICTOR / Q6R327 / ENSSSCG00000016853 / ENSG00000164327
RIDA / P52758 / ENSSSCG00000006079 / ENSG00000132541
RILPL1 / Q5EBL4 / ENSSSCG00000009773 / ENSG00000188026
RINT1 / Q6NUQ1 / ENSSSCG00000015430 / ENSG00000135249
RIOX2 / Q8IUF8 / ENSSSCG00000034501 / ENSG00000170854
RIPK1 / Q13546 / ENSSSCG00000001009 / ENSG00000137275
RIT1 / Q92963 / ENSSSCG00000023991 / ENSG00000143622
RMDN1 / Q96DB5 / ENSSSCG00000006136 / ENSG00000176623
RMDN3 / Q96TC7 / ENSSSCG00000004765 / ENSG00000137824
RMND1 / Q9NWS8 / ENSSSCG00000004089 / ENSG00000155906
RNASEH2A / O75792 / ENSSSCG00000013737 / ENSG00000104889
RNASEH2C / Q8TDP1 / ENSSSCG00000058858 / ENSG00000172922
RNASET2 / O00584 / ENSSSCG00000004024 / ENSG00000026297
RNF114 / Q9Y508 / ENSSSCG00000036129 / ENSG00000124226
RNF123 / Q5XPI4 / ENSSSCG00000011387 / ENSG00000164068
RNF170 / Q96K19 / ENSSSCG00000036001 / ENSG00000120925
RNF20 / Q5VTR2 / ENSSSCG00000005399 / ENSG00000155827
RNMT / O43148 / ENSSSCG00000025855 / ENSG00000101654
RNPEP / Q9H4A4 / ENSSSCG00000034151 / ENSG00000176393
RO60 / P10155 / ENSSSCG00000032055 / ENSG00000116747
ROCK1 / Q13464 / ENSSSCG00000021893 / ENSG00000067900
ROCK2 / O75116 / ENSSSCG00000008629 / ENSG00000134318
ROMO1 / P60602 / ENSSSCG00000007308 / ENSG00000125995
RP2 / O75695 / ENSSSCG00000027701 / ENSG00000102218
RPA1 / P27694 / ENSSSCG00000017822 / ENSG00000132383
RPA2 / P15927 / ENSSSCG00000003583 / ENSG00000117748
RPA3 / P35244 / ENSSSCG00000023886 / ENSG00000106399
RPAP1 / Q9BWH6 / ENSSSCG00000004745 / ENSG00000103932
RPAP3 / Q9H6T3 / ENSSSCG00000021166 / ENSG00000005175
RPIA / P49247 / ENSSSCG00000008207 / ENSG00000153574
RPL10A / P62906 / ENSSSCG00000001543 / ENSG00000198755
RPL11 / P62913 / ENSSSCG00000024260 / ENSG00000142676
RPL15 / P61313 / ENSSSCG00000037030 / ENSG00000174748
RPL18A / Q02543 / ENSSSCG00000013889 / ENSG00000105640
RPL23A / P62750 / ENSSSCG00000035080 / ENSG00000198242
RPL26L1 / Q9UNX3 / ENSSSCG00000016989 / ENSG00000037241
RPL27 / P61353 / ENSSSCG00000025507 / ENSG00000131469
RPL28 / P46779 / ENSSSCG00000039460 / ENSG00000108107
RPL3 / P39023 / ENSSSCG00000040494 / ENSG00000100316
RPL30 / P62888 / ENSSSCG00000006081 / ENSG00000156482
RPL32 / P62910 / ENSSSCG00000035811 / ENSG00000144713
RPL34 / P49207 / ENSSSCG00000009146 / ENSG00000109475
RPL35 / P42766 / ENSSSCG00000034227 / ENSG00000136942
Homologs
Adipose.Tissue
Brain
Colon
Heart
Kidney
Liver
Lung
Pancreas
S.Intestine
Spleen
Organs
Species
Human
Pig
Bins
1
2
3
4
5

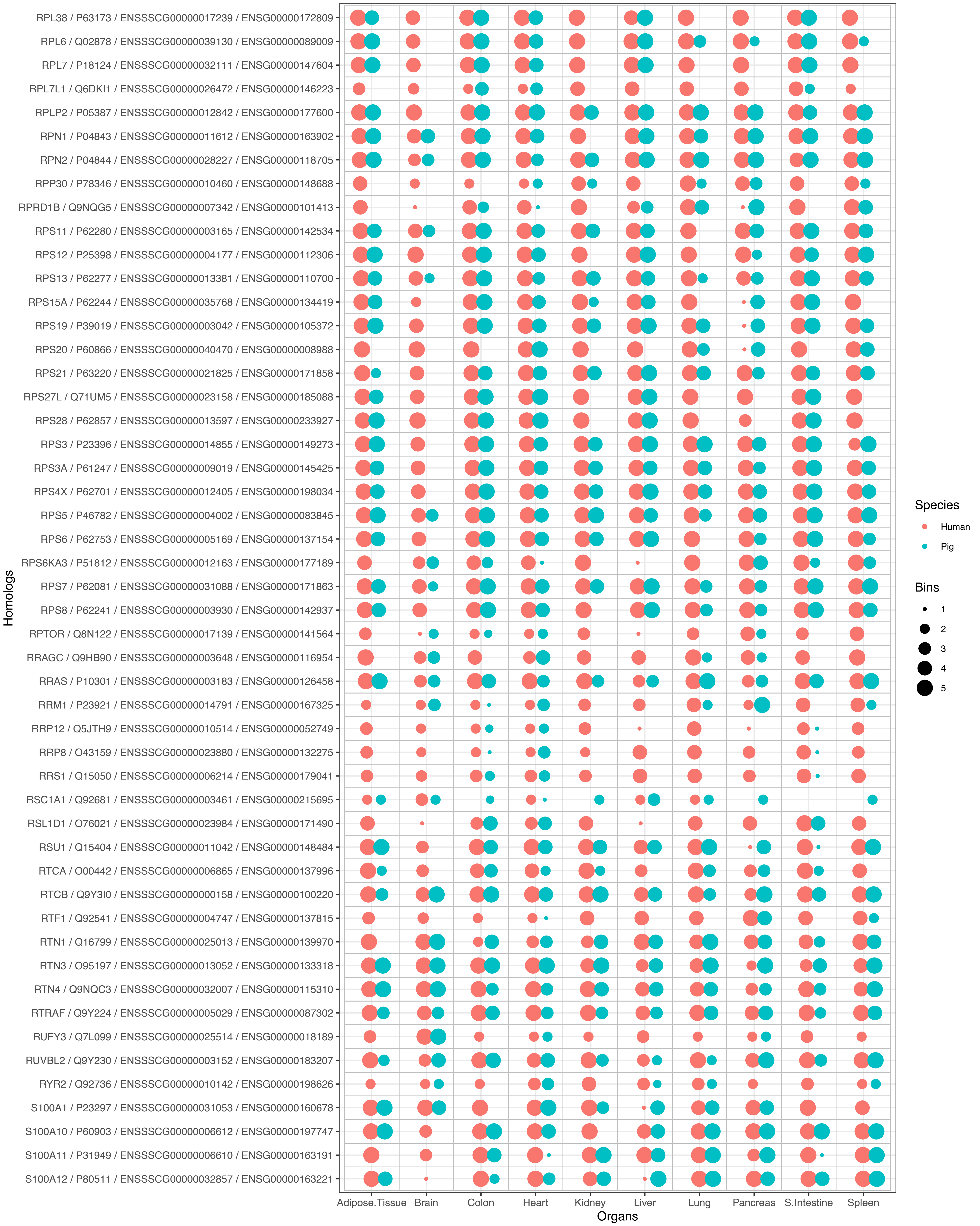

RPL38 / P63173 / ENSSSCG00000017239 / ENSG00000172809
RPL6 / Q02878 / ENSSSCG00000039130 / ENSG00000089009
RPL7 / P18124 / ENSSSCG00000032111 / ENSG00000147604
RPL7L1 / Q6DKI1 / ENSSSCG00000026472 / ENSG00000146223
RPLP2 / P05387 / ENSSSCG00000012842 / ENSG00000177600
RPN1 / P04843 / ENSSSCG00000011612 / ENSG00000163902
RPN2 / P04844 / ENSSSCG00000028227 / ENSG00000118705
RPP30 / P78346 / ENSSSCG00000010460 / ENSG00000148688
RPRD1B / Q9NQG5 / ENSSSCG00000007342 / ENSG00000101413
RPS11 / P62280 / ENSSSCG00000003165 / ENSG00000142534
RPS12 / P25398 / ENSSSCG00000004177 / ENSG00000112306
RPS13 / P62277 / ENSSSCG00000013381 / ENSG00000110700
RPS15A / P62244 / ENSSSCG00000035768 / ENSG00000134419
RPS19 / P39019 / ENSSSCG00000003042 / ENSG00000105372
RPS20 / P60866 / ENSSSCG00000040470 / ENSG00000008988
RPS21 / P63220 / ENSSSCG00000021825 / ENSG00000171858
RPS27L / Q71UM5 / ENSSSCG00000023158 / ENSG00000185088
RPS28 / P62857 / ENSSSCG00000013597 / ENSG00000233927
RPS3 / P23396 / ENSSSCG00000014855 / ENSG00000149273
RPS3A / P61247 / ENSSSCG00000009019 / ENSG00000145425
RPS4X / P62701 / ENSSSCG00000012405 / ENSG00000198034
RPS5 / P46782 / ENSSSCG00000004002 / ENSG00000083845
RPS6 / P62753 / ENSSSCG00000005169 / ENSG00000137154
RPS6KA3 / P51812 / ENSSSCG00000012163 / ENSG00000177189
RPS7 / P62081 / ENSSSCG00000031088 / ENSG00000171863
RPS8 / P62241 / ENSSSCG00000003930 / ENSG00000142937
RPTOR / Q8N122 / ENSSSCG00000017139 / ENSG00000141564
RRAGC / Q9HB90 / ENSSSCG00000003648 / ENSG00000116954
RRAS / P10301 / ENSSSCG00000003183 / ENSG00000126458
RRM1 / P23921 / ENSSSCG00000014791 / ENSG00000167325
RRP12 / Q5JTH9 / ENSSSCG00000010514 / ENSG00000052749
RRP8 / O43159 / ENSSSCG00000023880 / ENSG00000132275
RRS1 / Q15050 / ENSSSCG00000006214 / ENSG00000179041
RSC1A1 / Q92681 / ENSSSCG00000003461 / ENSG00000215695
RSL1D1 / O76021 / ENSSSCG00000023984 / ENSG00000171490
RSU1 / Q15404 / ENSSSCG00000011042 / ENSG00000148484
RTCA / O00442 / ENSSSCG00000006865 / ENSG00000137996
RTCB / Q9Y3I0 / ENSSSCG00000000158 / ENSG00000100220
RTF1 / Q92541 / ENSSSCG00000004747 / ENSG00000137815
RTN1 / Q16799 / ENSSSCG00000025013 / ENSG00000139970
RTN3 / O95197 / ENSSSCG00000013052 / ENSG00000133318
RTN4 / Q9NQC3 / ENSSSCG00000032007 / ENSG00000115310
RTRAF / Q9Y224 / ENSSSCG00000005029 / ENSG00000087302
RUFY3 / Q7L099 / ENSSSCG00000025514 / ENSG00000018189
RUVBL2 / Q9Y230 / ENSSSCG00000003152 / ENSG00000183207
RYR2 / Q92736 / ENSSSCG00000010142 / ENSG00000198626
S100A1 / P23297 / ENSSSCG00000031053 / ENSG00000160678
S100A10 / P60903 / ENSSSCG00000006612 / ENSG00000197747
S100A11 / P31949 / ENSSSCG00000006610 / ENSG00000163191
S100A12 / P80511 / ENSSSCG00000032857 / ENSG00000163221
Homologs
Adipose.Tissue
Brain
Colon
Heart
Kidney
Liver
Lung
Pancreas
S.Intestine
Spleen
Organs
Species
Human
Pig
Bins
1
2
3
4
5

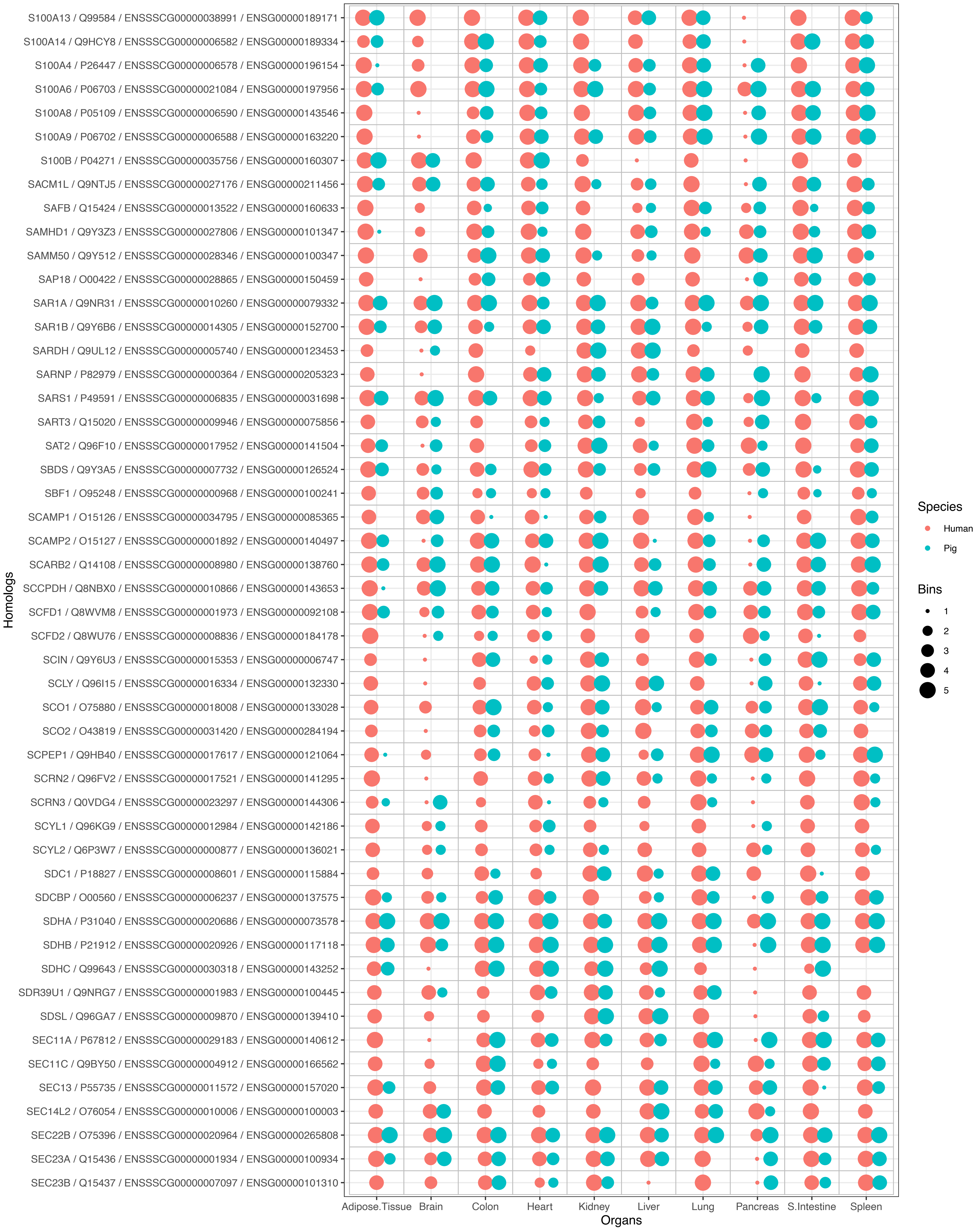
S100A13 / Q99584 / ENSSSCG00000038991 / ENSG00000189171
S100A14 / Q9HCY8 / ENSSSCG00000006582 / ENSG00000189334
S100A4 / P26447 / ENSSSCG00000006578 / ENSG00000196154
S100A6 / P06703 / ENSSSCG00000021084 / ENSG00000197956
S100A8 / P05109 / ENSSSCG00000006590 / ENSG00000143546
S100A9 / P06702 / ENSSSCG00000006588 / ENSG00000163220
S100B / P04271 / ENSSSCG00000035756 / ENSG00000160307
SACM1L / Q9NTJ5 / ENSSSCG00000027176 / ENSG00000211456
SAFB / Q15424 / ENSSSCG00000013522 / ENSG00000160633
SAMHD1 / Q9Y3Z3 / ENSSSCG00000027806 / ENSG00000101347
SAMM50 / Q9Y512 / ENSSSCG00000028346 / ENSG00000100347
SAP18 / O00422 / ENSSSCG00000028865 / ENSG00000150459
SAR1A / Q9NR31 / ENSSSCG00000010260 / ENSG00000079332
SAR1B / Q9Y6B6 / ENSSSCG00000014305 / ENSG00000152700
SARDH / Q9UL12 / ENSSSCG00000005740 / ENSG00000123453
SARNP / P82979 / ENSSSCG00000000364 / ENSG00000205323
SARS1 / P49591 / ENSSSCG00000006835 / ENSG00000031698
SART3 / Q15020 / ENSSSCG00000009946 / ENSG00000075856
SAT2 / Q96F10 / ENSSSCG00000017952 / ENSG00000141504
SBDS / Q9Y3A5 / ENSSSCG00000007732 / ENSG00000126524
SBF1 / O95248 / ENSSSCG00000000968 / ENSG00000100241
SCAMP1 / O15126 / ENSSSCG00000034795 / ENSG00000085365
SCAMP2 / O15127 / ENSSSCG00000001892 / ENSG00000140497
SCARB2 / Q14108 / ENSSSCG00000008980 / ENSG00000138760
SCCPDH / Q8NBX0 / ENSSSCG00000010866 / ENSG00000143653
SCFD1 / Q8WVM8 / ENSSSCG00000001973 / ENSG00000092108
SCFD2 / Q8WU76 / ENSSSCG00000008836 / ENSG00000184178
SCIN / Q9Y6U3 / ENSSSCG00000015353 / ENSG00000006747
SCLY / Q96I15 / ENSSSCG00000016334 / ENSG00000132330
SCO1 / O75880 / ENSSSCG00000018008 / ENSG00000133028
SCO2 / O43819 / ENSSSCG00000031420 / ENSG00000284194
SCPEP1 / Q9HB40 / ENSSSCG00000017617 / ENSG00000121064
SCRN2 / Q96FV2 / ENSSSCG00000017521 / ENSG00000141295
SCRN3 / Q0VDG4 / ENSSSCG00000023297 / ENSG00000144306
SCYL1 / Q96KG9 / ENSSSCG00000012984 / ENSG00000142186
SCYL2 / Q6P3W7 / ENSSSCG00000000877 / ENSG00000136021
SDC1 / P18827 / ENSSSCG00000008601 / ENSG00000115884
SDCBP / O00560 / ENSSSCG00000006237 / ENSG00000137575
SDHA / P31040 / ENSSSCG00000020686 / ENSG00000073578
SDHB / P21912 / ENSSSCG00000020926 / ENSG00000117118
SDHC / Q99643 / ENSSSCG00000030318 / ENSG00000143252
SDR39U1 / Q9NRG7 / ENSSSCG00000001983 / ENSG00000100445
SDSL / Q96GA7 / ENSSSCG00000009870 / ENSG00000139410
SEC11A / P67812 / ENSSSCG00000029183 / ENSG00000140612
SEC11C / Q9BY50 / ENSSSCG00000004912 / ENSG00000166562
SEC13 / P55735 / ENSSSCG00000011572 / ENSG00000157020
SEC14L2 / O76054 / ENSSSCG00000010006 / ENSG00000100003
SEC22B / O75396 / ENSSSCG00000020964 / ENSG00000265808
SEC23A / Q15436 / ENSSSCG00000001934 / ENSG00000100934
SEC23B / Q15437 / ENSSSCG00000007097 / ENSG00000101310
Homologs
Adipose.Tissue
Brain
Colon
Heart
Kidney
Liver
Lung
Pancreas
S.Intestine
Spleen
Organs
Species
Human
Pig
Bins
1
2
3
4
5

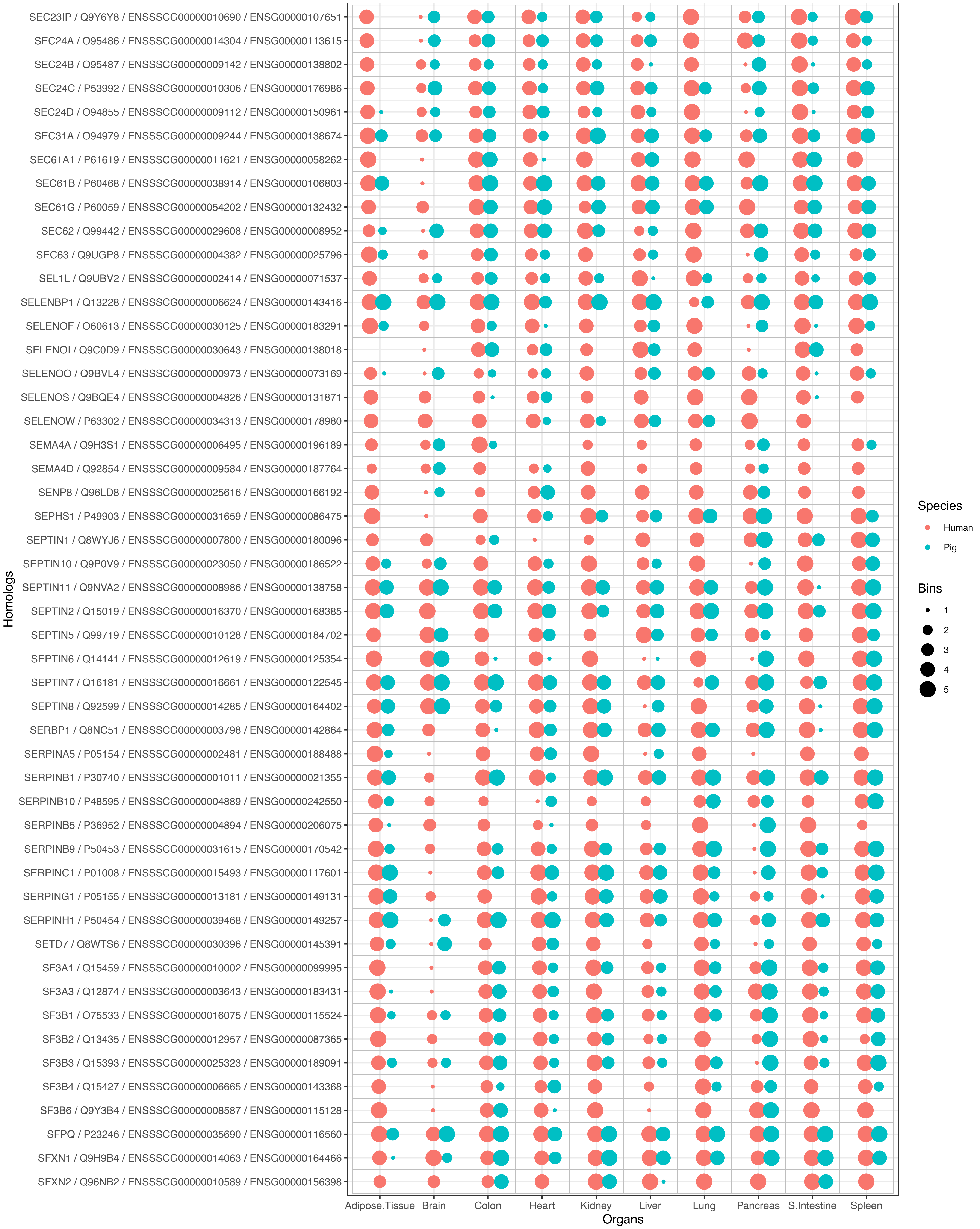
SEC23IP / Q9Y6Y8 / ENSSSCG00000010690 / ENSG00000107651
SEC24A / O95486 / ENSSSCG00000014304 / ENSG00000113615
SEC24B / O95487 / ENSSSCG00000009142 / ENSG00000138802
SEC24C / P53992 / ENSSSCG00000010306 / ENSG00000176986
SEC24D / O94855 / ENSSSCG00000009112 / ENSG00000150961
SEC31A / O94979 / ENSSSCG00000009244 / ENSG00000138674
SEC61A1 / P61619 / ENSSSCG00000011621 / ENSG00000058262
SEC61B / P60468 / ENSSSCG00000038914 / ENSG00000106803
SEC61G / P60059 / ENSSSCG00000054202 / ENSG00000132432
SEC62 / Q99442 / ENSSSCG00000029608 / ENSG00000008952
SEC63 / Q9UGP8 / ENSSSCG00000004382 / ENSG00000025796
SEL1L / Q9UBV2 / ENSSSCG00000002414 / ENSG00000071537
SELENBP1 / Q13228 / ENSSSCG00000006624 / ENSG00000143416
SELENOF / O60613 / ENSSSCG00000030125 / ENSG00000183291
SELENOI / Q9C0D9 / ENSSSCG00000030643 / ENSG00000138018
SELENOO / Q9BVL4 / ENSSSCG00000000973 / ENSG00000073169
SELENOS / Q9BQE4 / ENSSSCG00000004826 / ENSG00000131871
SELENOW / P63302 / ENSSSCG00000034313 / ENSG00000178980
SEMA4A / Q9H3S1 / ENSSSCG00000006495 / ENSG00000196189
SEMA4D / Q92854 / ENSSSCG00000009584 / ENSG00000187764
SENP8 / Q96LD8 / ENSSSCG00000025616 / ENSG00000166192
SEPHS1 / P49903 / ENSSSCG00000031659 / ENSG00000086475
SEPTIN1 / Q8WYJ6 / ENSSSCG00000007800 / ENSG00000180096
SEPTIN10 / Q9P0V9 / ENSSSCG00000023050 / ENSG00000186522
SEPTIN11 / Q9NVA2 / ENSSSCG00000008986 / ENSG00000138758
SEPTIN2 / Q15019 / ENSSSCG00000016370 / ENSG00000168385
SEPTIN5 / Q99719 / ENSSSCG00000010128 / ENSG00000184702
SEPTIN6 / Q14141 / ENSSSCG00000012619 / ENSG00000125354
SEPTIN7 / Q16181 / ENSSSCG00000016661 / ENSG00000122545
SEPTIN8 / Q92599 / ENSSSCG00000014285 / ENSG00000164402
SERBP1 / Q8NC51 / ENSSSCG00000003798 / ENSG00000142864
SERPINA5 / P05154 / ENSSSCG00000002481 / ENSG00000188488
SERPINB1 / P30740 / ENSSSCG00000001011 / ENSG00000021355
SERPINB10 / P48595 / ENSSSCG00000004889 / ENSG00000242550
SERPINB5 / P36952 / ENSSSCG00000004894 / ENSG00000206075
SERPINB9 / P50453 / ENSSSCG00000031615 / ENSG00000170542
SERPINC1 / P01008 / ENSSSCG00000015493 / ENSG00000117601
SERPING1 / P05155 / ENSSSCG00000013181 / ENSG00000149131
SERPINH1 / P50454 / ENSSSCG00000039468 / ENSG00000149257
SETD7 / Q8WTS6 / ENSSSCG00000030396 / ENSG00000145391
SF3A1 / Q15459 / ENSSSCG00000010002 / ENSG00000099995
SF3A3 / Q12874 / ENSSSCG00000003643 / ENSG00000183431
SF3B1 / O75533 / ENSSSCG00000016075 / ENSG00000115524
SF3B2 / Q13435 / ENSSSCG00000012957 / ENSG00000087365
SF3B3 / Q15393 / ENSSSCG00000025323 / ENSG00000189091
SF3B4 / Q15427 / ENSSSCG00000006665 / ENSG00000143368
SF3B6 / Q9Y3B4 / ENSSSCG00000008587 / ENSG00000115128
SFPQ / P23246 / ENSSSCG00000035690 / ENSG00000116560
SFXN1 / Q9H9B4 / ENSSSCG00000014063 / ENSG00000164466
SFXN2 / Q96NB2 / ENSSSCG00000010589 / ENSG00000156398
Homologs
Adipose.Tissue
Brain
Colon
Heart
Kidney
Liver
Lung
Pancreas
S.Intestine
Spleen
Organs
Species
Human
Pig
Bins
1
2
3
4
5

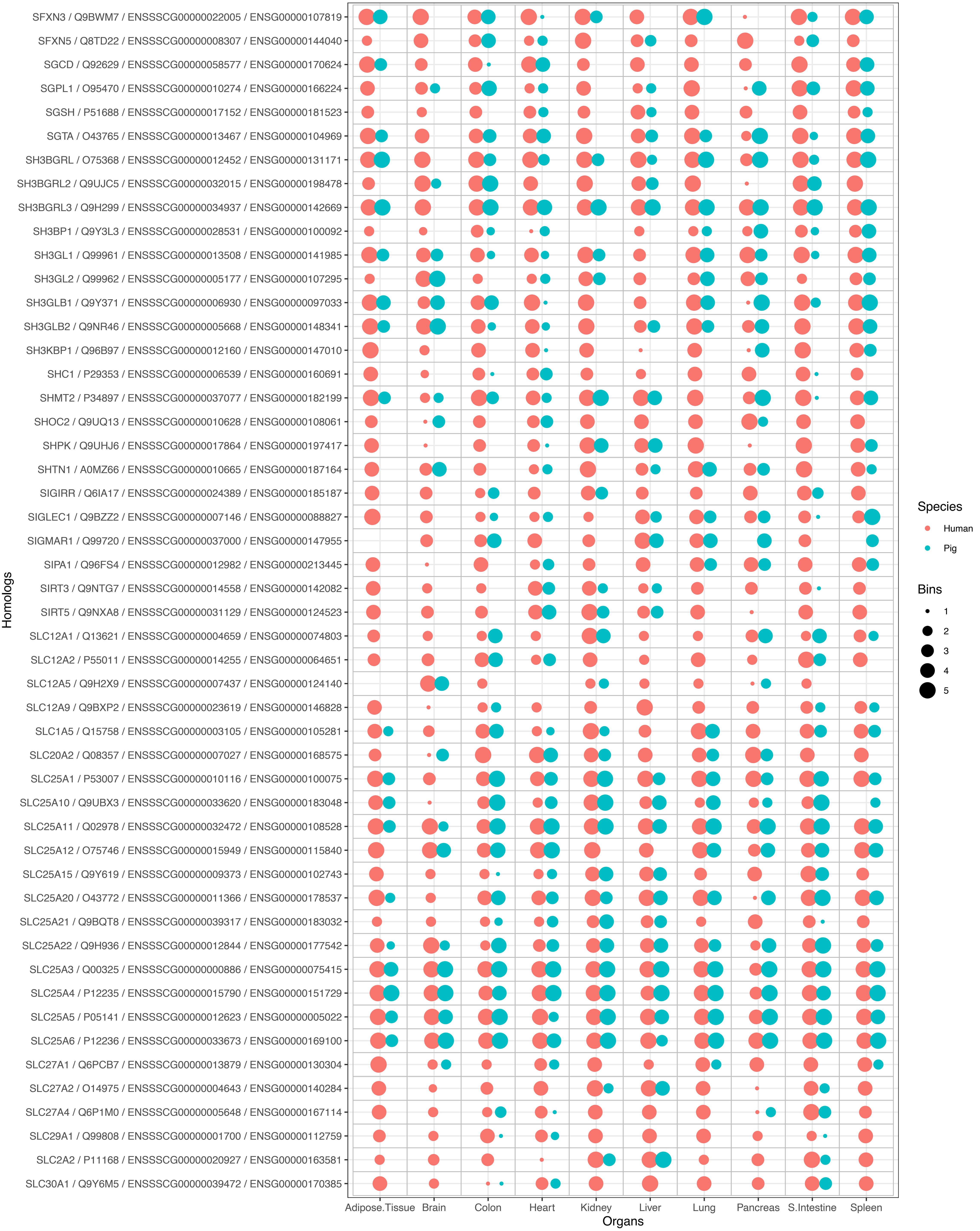
SFXN3 / Q9BWM7 / ENSSSCG00000022005 / ENSG00000107819
SFXN5 / Q8TD22 / ENSSSCG00000008307 / ENSG00000144040
SGCD / Q92629 / ENSSSCG00000058577 / ENSG00000170624
SGPL1 / O95470 / ENSSSCG00000010274 / ENSG00000166224
SGSH / P51688 / ENSSSCG00000017152 / ENSG00000181523
SGTA / O43765 / ENSSSCG00000013467 / ENSG00000104969
SH3BGRL / O75368 / ENSSSCG00000012452 / ENSG00000131171
SH3BGRL2 / Q9UJC5 / ENSSSCG00000032015 / ENSG00000198478
SH3BGRL3 / Q9H299 / ENSSSCG00000034937 / ENSG00000142669
SH3BP1 / Q9Y3L3 / ENSSSCG00000028531 / ENSG00000100092
SH3GL1 / Q99961 / ENSSSCG00000013508 / ENSG00000141985
SH3GL2 / Q99962 / ENSSSCG00000005177 / ENSG00000107295
SH3GLB1 / Q9Y371 / ENSSSCG00000006930 / ENSG00000097033
SH3GLB2 / Q9NR46 / ENSSSCG00000005668 / ENSG00000148341
SH3KBP1 / Q96B97 / ENSSSCG00000012160 / ENSG00000147010
SHC1 / P29353 / ENSSSCG00000006539 / ENSG00000160691
SHMT2 / P34897 / ENSSSCG00000037077 / ENSG00000182199
SHOC2 / Q9UQ13 / ENSSSCG00000010628 / ENSG00000108061
SHPK / Q9UHJ6 / ENSSSCG00000017864 / ENSG00000197417
SHTN1 / A0MZ66 / ENSSSCG00000010665 / ENSG00000187164
SIGIRR / Q6IA17 / ENSSSCG00000024389 / ENSG00000185187
SIGLEC1 / Q9BZZ2 / ENSSSCG00000007146 / ENSG00000088827
SIGMAR1 / Q99720 / ENSSSCG00000037000 / ENSG00000147955
SIPA1 / Q96FS4 / ENSSSCG00000012982 / ENSG00000213445
SIRT3 / Q9NTG7 / ENSSSCG00000014558 / ENSG00000142082
SIRT5 / Q9NXA8 / ENSSSCG00000031129 / ENSG00000124523
SLC12A1 / Q13621 / ENSSSCG00000004659 / ENSG00000074803
SLC12A2 / P55011 / ENSSSCG00000014255 / ENSG00000064651
SLC12A5 / Q9H2X9 / ENSSSCG00000007437 / ENSG00000124140
SLC12A9 / Q9BXP2 / ENSSSCG00000023619 / ENSG00000146828
SLC1A5 / Q15758 / ENSSSCG00000003105 / ENSG00000105281
SLC20A2 / Q08357 / ENSSSCG00000007027 / ENSG00000168575
SLC25A1 / P53007 / ENSSSCG00000010116 / ENSG00000100075
SLC25A10 / Q9UBX3 / ENSSSCG00000033620 / ENSG00000183048
SLC25A11 / Q02978 / ENSSSCG00000032472 / ENSG00000108528
SLC25A12 / O75746 / ENSSSCG00000015949 / ENSG00000115840
SLC25A15 / Q9Y619 / ENSSSCG00000009373 / ENSG00000102743
SLC25A20 / O43772 / ENSSSCG00000011366 / ENSG00000178537
SLC25A21 / Q9BQT8 / ENSSSCG00000039317 / ENSG00000183032
SLC25A22 / Q9H936 / ENSSSCG00000012844 / ENSG00000177542
SLC25A3 / Q00325 / ENSSSCG00000000886 / ENSG00000075415
SLC25A4 / P12235 / ENSSSCG00000015790 / ENSG00000151729
SLC25A5 / P05141 / ENSSSCG00000012623 / ENSG00000005022
SLC25A6 / P12236 / ENSSSCG00000033673 / ENSG00000169100
SLC27A1 / Q6PCB7 / ENSSSCG00000013879 / ENSG00000130304
SLC27A2 / O14975 / ENSSSCG00000004643 / ENSG00000140284
SLC27A4 / Q6P1M0 / ENSSSCG00000005648 / ENSG00000167114
SLC29A1 / Q99808 / ENSSSCG00000001700 / ENSG00000112759
SLC2A2 / P11168 / ENSSSCG00000020927 / ENSG00000163581
SLC30A1 / Q9Y6M5 / ENSSSCG00000039472 / ENSG00000170385
Homologs
Adipose.Tissue
Brain
Colon
Heart
Kidney
Liver
Lung
Pancreas
S.Intestine
Spleen
Organs
Species
Human
Pig
Bins
1
2
3
4
5

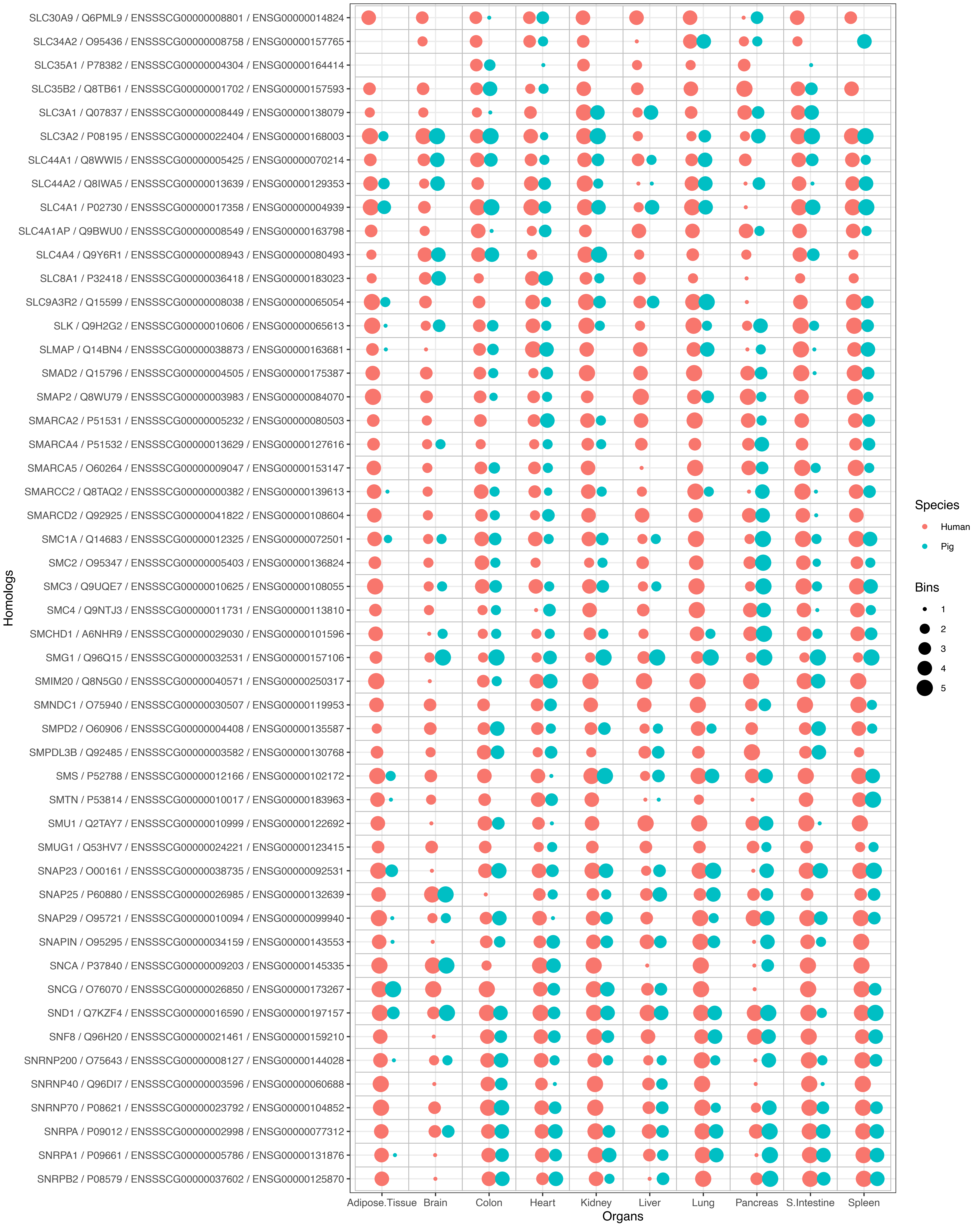
SLC30A9 / Q6PML9 / ENSSSCG00000008801 / ENSG00000014824
SLC34A2 / O95436 / ENSSSCG00000008758 / ENSG00000157765
SLC35A1 / P78382 / ENSSSCG00000004304 / ENSG00000164414
SLC35B2 / Q8TB61 / ENSSSCG00000001702 / ENSG00000157593
SLC3A1 / Q07837 / ENSSSCG00000008449 / ENSG00000138079
SLC3A2 / P08195 / ENSSSCG00000022404 / ENSG00000168003
SLC44A1 / Q8WWI5 / ENSSSCG00000005425 / ENSG00000070214
SLC44A2 / Q8IWA5 / ENSSSCG00000013639 / ENSG00000129353
SLC4A1 / P02730 / ENSSSCG00000017358 / ENSG00000004939
SLC4A1AP / Q9BWU0 / ENSSSCG00000008549 / ENSG00000163798
SLC4A4 / Q9Y6R1 / ENSSSCG00000008943 / ENSG00000080493
SLC8A1 / P32418 / ENSSSCG00000036418 / ENSG00000183023
SLC9A3R2 / Q15599 / ENSSSCG00000008038 / ENSG00000065054
SLK / Q9H2G2 / ENSSSCG00000010606 / ENSG00000065613
SLMAP / Q14BN4 / ENSSSCG00000038873 / ENSG00000163681
SMAD2 / Q15796 / ENSSSCG00000004505 / ENSG00000175387
SMAP2 / Q8WU79 / ENSSSCG00000003983 / ENSG00000084070
SMARCA2 / P51531 / ENSSSCG00000005232 / ENSG00000080503
SMARCA4 / P51532 / ENSSSCG00000013629 / ENSG00000127616
SMARCA5 / O60264 / ENSSSCG00000009047 / ENSG00000153147
SMARCC2 / Q8TAQ2 / ENSSSCG00000000382 / ENSG00000139613
SMARCD2 / Q92925 / ENSSSCG00000041822 / ENSG00000108604
SMC1A / Q14683 / ENSSSCG00000012325 / ENSG00000072501
SMC2 / O95347 / ENSSSCG00000005403 / ENSG00000136824
SMC3 / Q9UQE7 / ENSSSCG00000010625 / ENSG00000108055
SMC4 / Q9NTJ3 / ENSSSCG00000011731 / ENSG00000113810
SMCHD1 / A6NHR9 / ENSSSCG00000029030 / ENSG00000101596
SMG1 / Q96Q15 / ENSSSCG00000032531 / ENSG00000157106
SMIM20 / Q8N5G0 / ENSSSCG00000040571 / ENSG00000250317
SMNDC1 / O75940 / ENSSSCG00000030507 / ENSG00000119953
SMPD2 / O60906 / ENSSSCG00000004408 / ENSG00000135587
SMPDL3B / Q92485 / ENSSSCG00000003582 / ENSG00000130768
SMS / P52788 / ENSSSCG00000012166 / ENSG00000102172
SMTN / P53814 / ENSSSCG00000010017 / ENSG00000183963
SMU1 / Q2TAY7 / ENSSSCG00000010999 / ENSG00000122692
SMUG1 / Q53HV7 / ENSSSCG00000024221 / ENSG00000123415
SNAP23 / O00161 / ENSSSCG00000038735 / ENSG00000092531
SNAP25 / P60880 / ENSSSCG00000026985 / ENSG00000132639
SNAP29 / O95721 / ENSSSCG00000010094 / ENSG00000099940
SNAPIN / O95295 / ENSSSCG00000034159 / ENSG00000143553
SNCA / P37840 / ENSSSCG00000009203 / ENSG00000145335
SNCG / O76070 / ENSSSCG00000026850 / ENSG00000173267
SND1 / Q7KZF4 / ENSSSCG00000016590 / ENSG00000197157
SNF8 / Q96H20 / ENSSSCG00000021461 / ENSG00000159210
SNRNP200 / O75643 / ENSSSCG00000008127 / ENSG00000144028
SNRNP40 / Q96DI7 / ENSSSCG00000003596 / ENSG00000060688
SNRNP70 / P08621 / ENSSSCG00000023792 / ENSG00000104852
SNRPA / P09012 / ENSSSCG00000002998 / ENSG00000077312
SNRPA1 / P09661 / ENSSSCG00000005786 / ENSG00000131876
SNRPB2 / P08579 / ENSSSCG00000037602 / ENSG00000125870
Homologs
Adipose.Tissue
Brain
Colon
Heart
Kidney
Liver
Lung
Pancreas
S.Intestine
Spleen
Organs
Species
Human
Pig
Bins
1
2
3
4
5

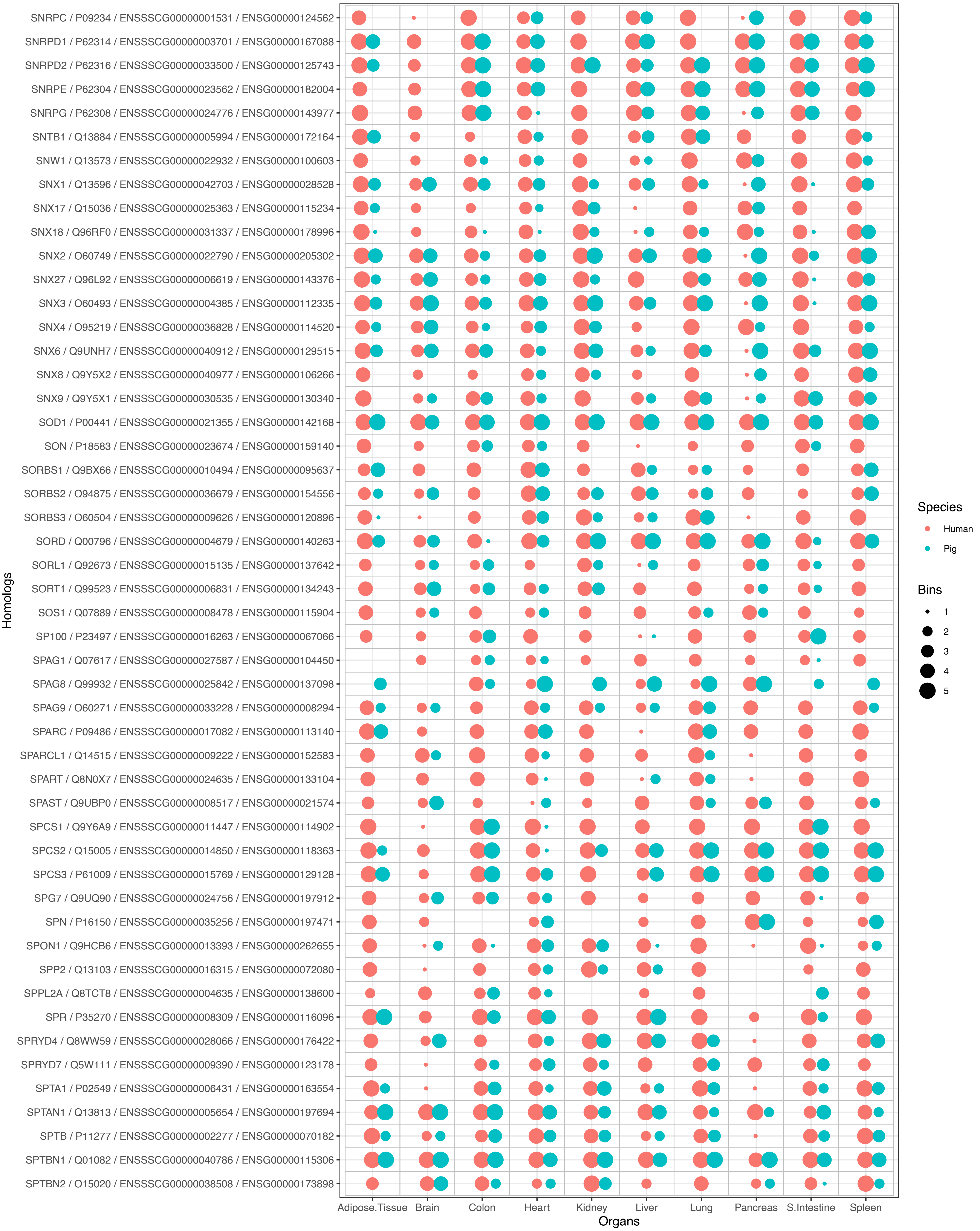
SNRPC / P09234 / ENSSSCG00000001531 / ENSG00000124562
SNRPD1 / P62314 / ENSSSCG00000003701 / ENSG00000167088
SNRPD2 / P62316 / ENSSSCG00000033500 / ENSG00000125743
SNRPE / P62304 / ENSSSCG00000023562 / ENSG00000182004
SNRPG / P62308 / ENSSSCG00000024776 / ENSG00000143977
SNTB1 / Q13884 / ENSSSCG00000005994 / ENSG00000172164
SNW1 / Q13573 / ENSSSCG00000022932 / ENSG00000100603
SNX1 / Q13596 / ENSSSCG00000042703 / ENSG00000028528
SNX17 / Q15036 / ENSSSCG00000025363 / ENSG00000115234
SNX18 / Q96RF0 / ENSSSCG00000031337 / ENSG00000178996
SNX2 / O60749 / ENSSSCG00000022790 / ENSG00000205302
SNX27 / Q96L92 / ENSSSCG00000006619 / ENSG00000143376
SNX3 / O60493 / ENSSSCG00000004385 / ENSG00000112335
SNX4 / O95219 / ENSSSCG00000036828 / ENSG00000114520
SNX6 / Q9UNH7 / ENSSSCG00000040912 / ENSG00000129515
SNX8 / Q9Y5X2 / ENSSSCG00000040977 / ENSG00000106266
SNX9 / Q9Y5X1 / ENSSSCG00000030535 / ENSG00000130340
SOD1 / P00441 / ENSSSCG00000021355 / ENSG00000142168
SON / P18583 / ENSSSCG00000023674 / ENSG00000159140
SORBS1 / Q9BX66 / ENSSSCG00000010494 / ENSG00000095637
SORBS2 / O94875 / ENSSSCG00000036679 / ENSG00000154556
SORBS3 / O60504 / ENSSSCG00000009626 / ENSG00000120896
SORD / Q00796 / ENSSSCG00000004679 / ENSG00000140263
SORL1 / Q92673 / ENSSSCG00000015135 / ENSG00000137642
SORT1 / Q99523 / ENSSSCG00000006831 / ENSG00000134243
SOS1 / Q07889 / ENSSSCG00000008478 / ENSG00000115904
SP100 / P23497 / ENSSSCG00000016263 / ENSG00000067066
SPAG1 / Q07617 / ENSSSCG00000027587 / ENSG00000104450
SPAG8 / Q99932 / ENSSSCG00000025842 / ENSG00000137098
SPAG9 / O60271 / ENSSSCG00000033228 / ENSG00000008294
SPARC / P09486 / ENSSSCG00000017082 / ENSG00000113140
SPARCL1 / Q14515 / ENSSSCG00000009222 / ENSG00000152583
SPART / Q8N0X7 / ENSSSCG00000024635 / ENSG00000133104
SPAST / Q9UBP0 / ENSSSCG00000008517 / ENSG00000021574
SPCS1 / Q9Y6A9 / ENSSSCG00000011447 / ENSG00000114902
SPCS2 / Q15005 / ENSSSCG00000014850 / ENSG00000118363
SPCS3 / P61009 / ENSSSCG00000015769 / ENSG00000129128
SPG7 / Q9UQ90 / ENSSSCG00000024756 / ENSG00000197912
SPN / P16150 / ENSSSCG00000035256 / ENSG00000197471
SPON1 / Q9HCB6 / ENSSSCG00000013393 / ENSG00000262655
SPP2 / Q13103 / ENSSSCG00000016315 / ENSG00000072080
SPPL2A / Q8TCT8 / ENSSSCG00000004635 / ENSG00000138600
SPR / P35270 / ENSSSCG00000008309 / ENSG00000116096
SPRYD4 / Q8WW59 / ENSSSCG00000028066 / ENSG00000176422
SPRYD7 / Q5W111 / ENSSSCG00000009390 / ENSG00000123178
SPTA1 / P02549 / ENSSSCG00000006431 / ENSG00000163554
SPTAN1 / Q13813 / ENSSSCG00000005654 / ENSG00000197694
SPTB / P11277 / ENSSSCG00000002277 / ENSG00000070182
SPTBN1 / Q01082 / ENSSSCG00000040786 / ENSG00000115306
SPTBN2 / O15020 / ENSSSCG00000038508 / ENSG00000173898
Homologs
Adipose.Tissue
Brain
Colon
Heart
Kidney
Liver
Lung
Pancreas
S.Intestine
Spleen
Organs
Species
Human
Pig
Bins
1
2
3
4
5

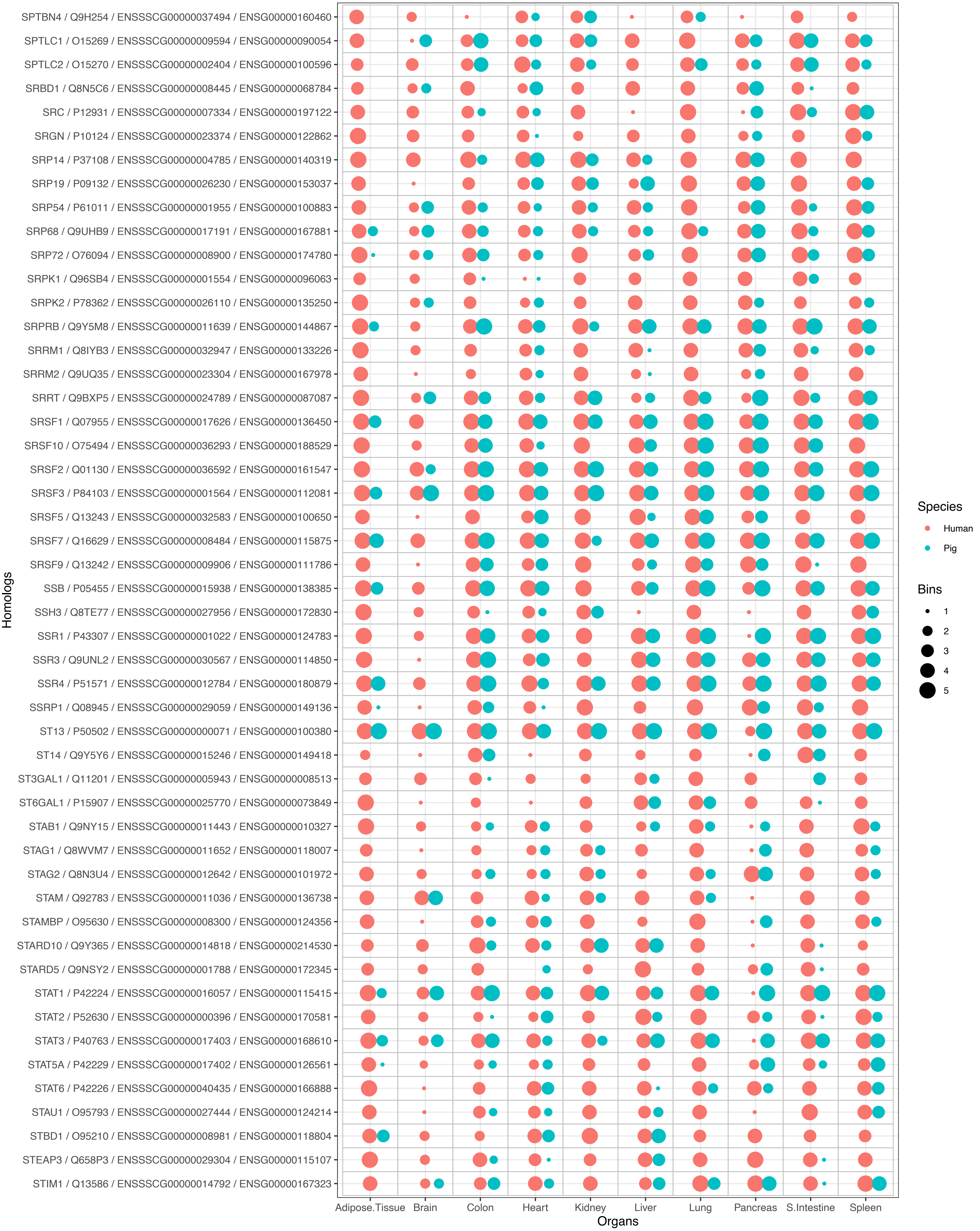
SPTBN4 / Q9H254 / ENSSSCG00000037494 / ENSG00000160460
SPTLC1 / O15269 / ENSSSCG00000009594 / ENSG00000090054
SPTLC2 / O15270 / ENSSSCG00000002404 / ENSG00000100596
SRBD1 / Q8N5C6 / ENSSSCG00000008445 / ENSG00000068784
SRC / P12931 / ENSSSCG00000007334 / ENSG00000197122
SRGN / P10124 / ENSSSCG00000023374 / ENSG00000122862
SRP14 / P37108 / ENSSSCG00000004785 / ENSG00000140319
SRP19 / P09132 / ENSSSCG00000026230 / ENSG00000153037
SRP54 / P61011 / ENSSSCG00000001955 / ENSG00000100883
SRP68 / Q9UHB9 / ENSSSCG00000017191 / ENSG00000167881
SRP72 / O76094 / ENSSSCG00000008900 / ENSG00000174780
SRPK1 / Q96SB4 / ENSSSCG00000001554 / ENSG00000096063
SRPK2 / P78362 / ENSSSCG00000026110 / ENSG00000135250
SRPRB / Q9Y5M8 / ENSSSCG00000011639 / ENSG00000144867
SRRM1 / Q8IYB3 / ENSSSCG00000032947 / ENSG00000133226
SRRM2 / Q9UQ35 / ENSSSCG00000023304 / ENSG00000167978
SRRT / Q9BXP5 / ENSSSCG00000024789 / ENSG00000087087
SRSF1 / Q07955 / ENSSSCG00000017626 / ENSG00000136450
SRSF10 / O75494 / ENSSSCG00000036293 / ENSG00000188529
SRSF2 / Q01130 / ENSSSCG00000036592 / ENSG00000161547
SRSF3 / P84103 / ENSSSCG00000001564 / ENSG00000112081
SRSF5 / Q13243 / ENSSSCG00000032583 / ENSG00000100650
SRSF7 / Q16629 / ENSSSCG00000008484 / ENSG00000115875
SRSF9 / Q13242 / ENSSSCG00000009906 / ENSG00000111786
SSB / P05455 / ENSSSCG00000015938 / ENSG00000138385
SSH3 / Q8TE77 / ENSSSCG00000027956 / ENSG00000172830
SSR1 / P43307 / ENSSSCG00000001022 / ENSG00000124783
SSR3 / Q9UNL2 / ENSSSCG00000030567 / ENSG00000114850
SSR4 / P51571 / ENSSSCG00000012784 / ENSG00000180879
SSRP1 / Q08945 / ENSSSCG00000029059 / ENSG00000149136
ST13 / P50502 / ENSSSCG00000000071 / ENSG00000100380
ST14 / Q9Y5Y6 / ENSSSCG00000015246 / ENSG00000149418
ST3GAL1 / Q11201 / ENSSSCG00000005943 / ENSG00000008513
ST6GAL1 / P15907 / ENSSSCG00000025770 / ENSG00000073849
STAB1 / Q9NY15 / ENSSSCG00000011443 / ENSG00000010327
STAG1 / Q8WVM7 / ENSSSCG00000011652 / ENSG00000118007
STAG2 / Q8N3U4 / ENSSSCG00000012642 / ENSG00000101972
STAM / Q92783 / ENSSSCG00000011036 / ENSG00000136738
STAMBP / O95630 / ENSSSCG00000008300 / ENSG00000124356
STARD10 / Q9Y365 / ENSSSCG00000014818 / ENSG00000214530
STARD5 / Q9NSY2 / ENSSSCG00000001788 / ENSG00000172345
STAT1 / P42224 / ENSSSCG00000016057 / ENSG00000115415
STAT2 / P52630 / ENSSSCG00000000396 / ENSG00000170581
STAT3 / P40763 / ENSSSCG00000017403 / ENSG00000168610
STAT5A / P42229 / ENSSSCG00000017402 / ENSG00000126561
STAT6 / P42226 / ENSSSCG00000040435 / ENSG00000166888
STAU1 / O95793 / ENSSSCG00000027444 / ENSG00000124214
STBD1 / O95210 / ENSSSCG00000008981 / ENSG00000118804
STEAP3 / Q658P3 / ENSSSCG00000029304 / ENSG00000115107
STIM1 / Q13586 / ENSSSCG00000014792 / ENSG00000167323
Homologs
Adipose.Tissue
Brain
Colon
Heart
Kidney
Liver
Lung
Pancreas
S.Intestine
Spleen
Organs
Species
Human
Pig
Bins
1
2
3
4
5

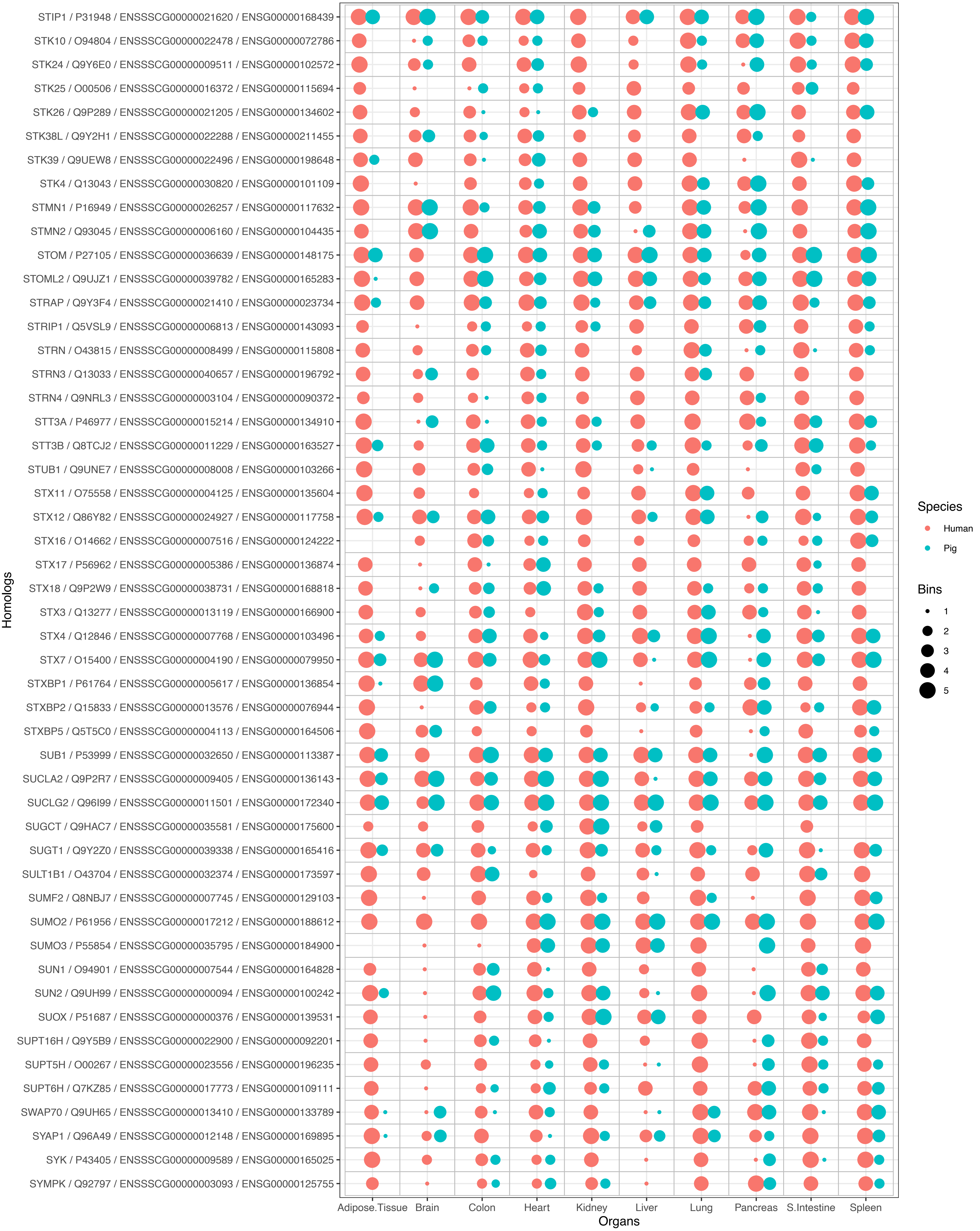

STIP1 / P31948 / ENSSSCG00000021620 / ENSG00000168439
STK10 / O94804 / ENSSSCG00000022478 / ENSG00000072786
STK24 / Q9Y6E0 / ENSSSCG00000009511 / ENSG00000102572
STK25 / O00506 / ENSSSCG00000016372 / ENSG00000115694
STK26 / Q9P289 / ENSSSCG00000021205 / ENSG00000134602
STK38L / Q9Y2H1 / ENSSSCG00000022288 / ENSG00000211455
STK39 / Q9UEW8 / ENSSSCG00000022496 / ENSG00000198648
STK4 / Q13043 / ENSSSCG00000030820 / ENSG00000101109
STMN1 / P16949 / ENSSSCG00000026257 / ENSG00000117632
STMN2 / Q93045 / ENSSSCG00000006160 / ENSG00000104435
STOM / P27105 / ENSSSCG00000036639 / ENSG00000148175
STOML2 / Q9UJZ1 / ENSSSCG00000039782 / ENSG00000165283
STRAP / Q9Y3F4 / ENSSSCG00000021410 / ENSG00000023734
STRIP1 / Q5VSL9 / ENSSSCG00000006813 / ENSG00000143093
STRN / O43815 / ENSSSCG00000008499 / ENSG00000115808
STRN3 / Q13033 / ENSSSCG00000040657 / ENSG00000196792
STRN4 / Q9NRL3 / ENSSSCG00000003104 / ENSG00000090372
STT3A / P46977 / ENSSSCG00000015214 / ENSG00000134910
STT3B / Q8TCJ2 / ENSSSCG00000011229 / ENSG00000163527
STUB1 / Q9UNE7 / ENSSSCG00000008008 / ENSG00000103266
STX11 / O75558 / ENSSSCG00000004125 / ENSG00000135604
STX12 / Q86Y82 / ENSSSCG00000024927 / ENSG00000117758
STX16 / O14662 / ENSSSCG00000007516 / ENSG00000124222
STX17 / P56962 / ENSSSCG00000005386 / ENSG00000136874
STX18 / Q9P2W9 / ENSSSCG00000038731 / ENSG00000168818
STX3 / Q13277 / ENSSSCG00000013119 / ENSG00000166900
STX4 / Q12846 / ENSSSCG00000007768 / ENSG00000103496
STX7 / O15400 / ENSSSCG00000004190 / ENSG00000079950
STXBP1 / P61764 / ENSSSCG00000005617 / ENSG00000136854
STXBP2 / Q15833 / ENSSSCG00000013576 / ENSG00000076944
STXBP5 / Q5T5C0 / ENSSSCG00000004113 / ENSG00000164506
SUB1 / P53999 / ENSSSCG00000032650 / ENSG00000113387
SUCLA2 / Q9P2R7 / ENSSSCG00000009405 / ENSG00000136143
SUCLG2 / Q96I99 / ENSSSCG00000011501 / ENSG00000172340
SUGCT / Q9HAC7 / ENSSSCG00000035581 / ENSG00000175600
SUGT1 / Q9Y2Z0 / ENSSSCG00000039338 / ENSG00000165416
SULT1B1 / O43704 / ENSSSCG00000032374 / ENSG00000173597
SUMF2 / Q8NBJ7 / ENSSSCG00000007745 / ENSG00000129103
SUMO2 / P61956 / ENSSSCG00000017212 / ENSG00000188612
SUMO3 / P55854 / ENSSSCG00000035795 / ENSG00000184900
SUN1 / O94901 / ENSSSCG00000007544 / ENSG00000164828
SUN2 / Q9UH99 / ENSSSCG00000000094 / ENSG00000100242
SUOX / P51687 / ENSSSCG00000000376 / ENSG00000139531
SUPT16H / Q9Y5B9 / ENSSSCG00000022900 / ENSG00000092201
SUPT5H / O00267 / ENSSSCG00000023556 / ENSG00000196235
SUPT6H / Q7KZ85 / ENSSSCG00000017773 / ENSG00000109111
SWAP70 / Q9UH65 / ENSSSCG00000013410 / ENSG00000133789
SYAP1 / Q96A49 / ENSSSCG00000012148 / ENSG00000169895
SYK / P43405 / ENSSSCG00000009589 / ENSG00000165025
SYMPK / Q92797 / ENSSSCG00000003093 / ENSG00000125755
Homologs
Adipose.Tissue
Brain
Colon
Heart
Kidney
Liver
Lung
Pancreas
S.Intestine
Spleen
Organs
Species
Human
Pig
Bins
1
2
3
4
5

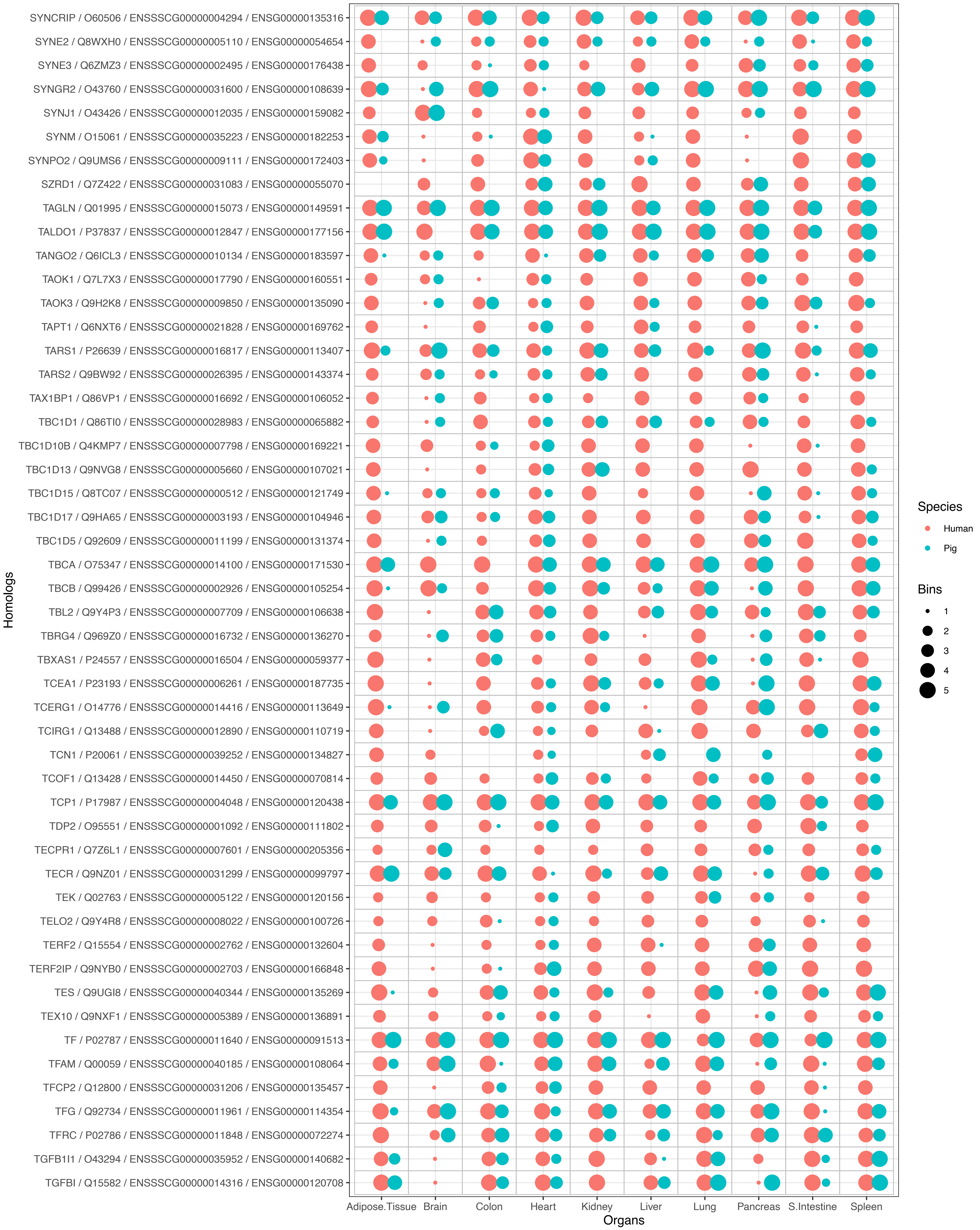
SYNCRIP / O60506 / ENSSSCG00000004294 / ENSG00000135316
SYNE2 / Q8WXH0 / ENSSSCG00000005110 / ENSG00000054654
SYNE3 / Q6ZMZ3 / ENSSSCG00000002495 / ENSG00000176438
SYNGR2 / O43760 / ENSSSCG00000031600 / ENSG00000108639
SYNJ1 / O43426 / ENSSSCG00000012035 / ENSG00000159082
SYNM / O15061 / ENSSSCG00000035223 / ENSG00000182253
SYNPO2 / Q9UMS6 / ENSSSCG00000009111 / ENSG00000172403
SZRD1 / Q7Z422 / ENSSSCG00000031083 / ENSG00000055070
TAGLN / Q01995 / ENSSSCG00000015073 / ENSG00000149591
TALDO1 / P37837 / ENSSSCG00000012847 / ENSG00000177156
TANGO2 / Q6ICL3 / ENSSSCG00000010134 / ENSG00000183597
TAOK1 / Q7L7X3 / ENSSSCG00000017790 / ENSG00000160551
TAOK3 / Q9H2K8 / ENSSSCG00000009850 / ENSG00000135090
TAPT1 / Q6NXT6 / ENSSSCG00000021828 / ENSG00000169762
TARS1 / P26639 / ENSSSCG00000016817 / ENSG00000113407
TARS2 / Q9BW92 / ENSSSCG00000026395 / ENSG00000143374
TAX1BP1 / Q86VP1 / ENSSSCG00000016692 / ENSG00000106052
TBC1D1 / Q86TI0 / ENSSSCG00000028983 / ENSG00000065882
TBC1D10B / Q4KMP7 / ENSSSCG00000007798 / ENSG00000169221
TBC1D13 / Q9NVG8 / ENSSSCG00000005660 / ENSG00000107021
TBC1D15 / Q8TC07 / ENSSSCG00000000512 / ENSG00000121749
TBC1D17 / Q9HA65 / ENSSSCG00000003193 / ENSG00000104946
TBC1D5 / Q92609 / ENSSSCG00000011199 / ENSG00000131374
TBCA / O75347 / ENSSSCG00000014100 / ENSG00000171530
TBCB / Q99426 / ENSSSCG00000002926 / ENSG00000105254
TBL2 / Q9Y4P3 / ENSSSCG00000007709 / ENSG00000106638
TBRG4 / Q969Z0 / ENSSSCG00000016732 / ENSG00000136270
TBXAS1 / P24557 / ENSSSCG00000016504 / ENSG00000059377
TCEA1 / P23193 / ENSSSCG00000006261 / ENSG00000187735
TCERG1 / O14776 / ENSSSCG00000014416 / ENSG00000113649
TCIRG1 / Q13488 / ENSSSCG00000012890 / ENSG00000110719
TCN1 / P20061 / ENSSSCG00000039252 / ENSG00000134827
TCOF1 / Q13428 / ENSSSCG00000014450 / ENSG00000070814
TCP1 / P17987 / ENSSSCG00000004048 / ENSG00000120438
TDP2 / O95551 / ENSSSCG00000001092 / ENSG00000111802
TECPR1 / Q7Z6L1 / ENSSSCG00000007601 / ENSG00000205356
TECR / Q9NZ01 / ENSSSCG00000031299 / ENSG00000099797
TEK / Q02763 / ENSSSCG00000005122 / ENSG00000120156
TELO2 / Q9Y4R8 / ENSSSCG00000008022 / ENSG00000100726
TERF2 / Q15554 / ENSSSCG00000002762 / ENSG00000132604
TERF2IP / Q9NYB0 / ENSSSCG00000002703 / ENSG00000166848
TES / Q9UGI8 / ENSSSCG00000040344 / ENSG00000135269
TEX10 / Q9NXF1 / ENSSSCG00000005389 / ENSG00000136891
TF / P02787 / ENSSSCG00000011640 / ENSG00000091513
TFAM / Q00059 / ENSSSCG00000040185 / ENSG00000108064
TFCP2 / Q12800 / ENSSSCG00000031206 / ENSG00000135457
TFG / Q92734 / ENSSSCG00000011961 / ENSG00000114354
TFRC / P02786 / ENSSSCG00000011848 / ENSG00000072274
TGFB1I1 / O43294 / ENSSSCG00000035952 / ENSG00000140682
TGFBI / Q15582 / ENSSSCG00000014316 / ENSG00000120708
Homologs
Adipose.Tissue
Brain
Colon
Heart
Kidney
Liver
Lung
Pancreas
S.Intestine
Spleen
Organs
Species
Human
Pig
Bins
1
2
3
4
5

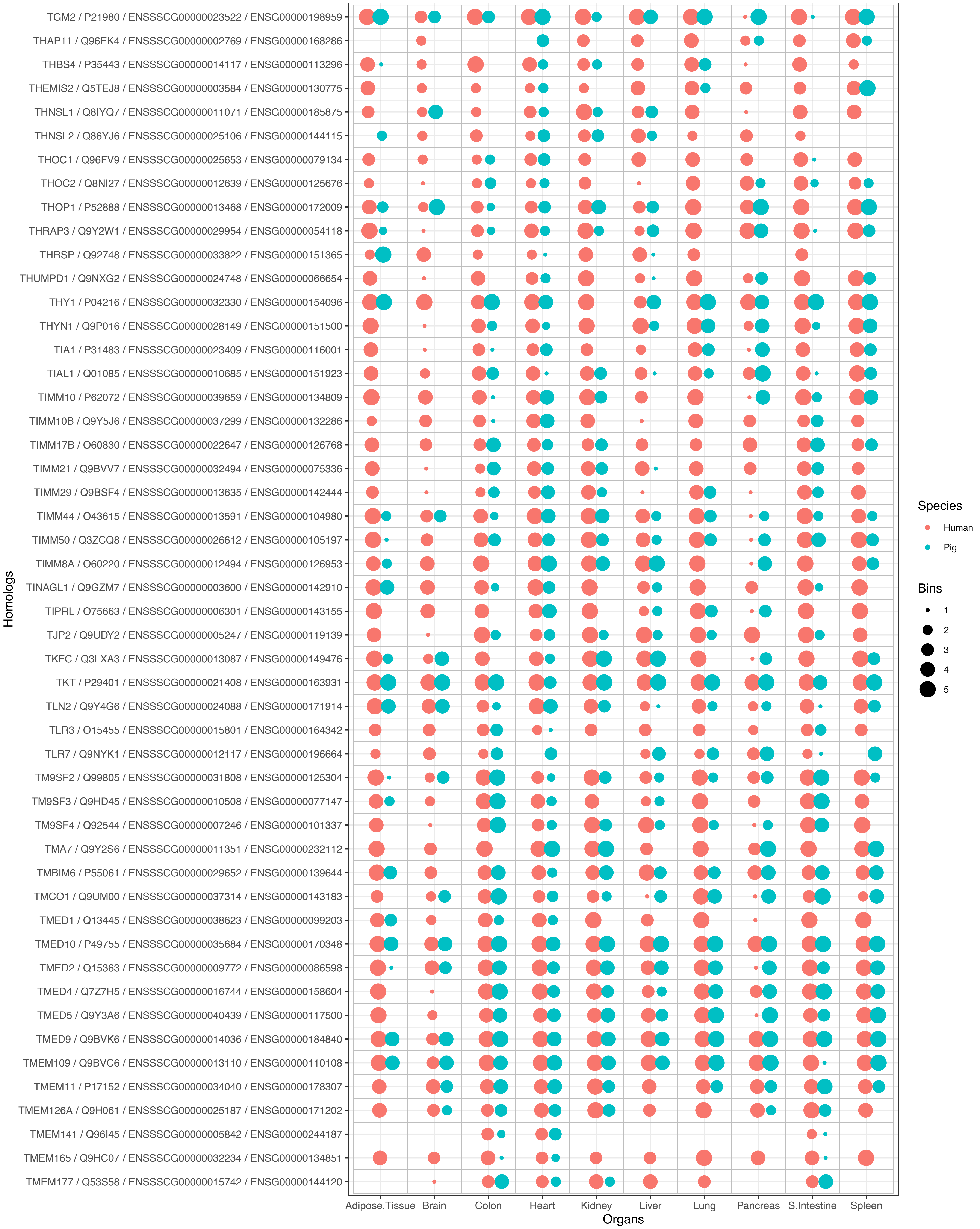
TGM2 / P21980 / ENSSSCG00000023522 / ENSG00000198959
THAP11 / Q96EK4 / ENSSSCG00000002769 / ENSG00000168286
THBS4 / P35443 / ENSSSCG00000014117 / ENSG00000113296
THEMIS2 / Q5TEJ8 / ENSSSCG00000003584 / ENSG00000130775
THNSL1 / Q8IYQ7 / ENSSSCG00000011071 / ENSG00000185875
THNSL2 / Q86YJ6 / ENSSSCG00000025106 / ENSG00000144115
THOC1 / Q96FV9 / ENSSSCG00000025653 / ENSG00000079134
THOC2 / Q8NI27 / ENSSSCG00000012639 / ENSG00000125676
THOP1 / P52888 / ENSSSCG00000013468 / ENSG00000172009
THRAP3 / Q9Y2W1 / ENSSSCG00000029954 / ENSG00000054118
THRSP / Q92748 / ENSSSCG00000033822 / ENSG00000151365
THUMPD1 / Q9NXG2 / ENSSSCG00000024748 / ENSG00000066654
THY1 / P04216 / ENSSSCG00000032330 / ENSG00000154096
THYN1 / Q9P016 / ENSSSCG00000028149 / ENSG00000151500
TIA1 / P31483 / ENSSSCG00000023409 / ENSG00000116001
TIAL1 / Q01085 / ENSSSCG00000010685 / ENSG00000151923
TIMM10 / P62072 / ENSSSCG00000039659 / ENSG00000134809
TIMM10B / Q9Y5J6 / ENSSSCG00000037299 / ENSG00000132286
TIMM17B / O60830 / ENSSSCG00000022647 / ENSG00000126768
TIMM21 / Q9BVV7 / ENSSSCG00000032494 / ENSG00000075336
TIMM29 / Q9BSF4 / ENSSSCG00000013635 / ENSG00000142444
TIMM44 / O43615 / ENSSSCG00000013591 / ENSG00000104980
TIMM50 / Q3ZCQ8 / ENSSSCG00000026612 / ENSG00000105197
TIMM8A / O60220 / ENSSSCG00000012494 / ENSG00000126953
TINAGL1 / Q9GZM7 / ENSSSCG00000003600 / ENSG00000142910
TIPRL / O75663 / ENSSSCG00000006301 / ENSG00000143155
TJP2 / Q9UDY2 / ENSSSCG00000005247 / ENSG00000119139
TKFC / Q3LXA3 / ENSSSCG00000013087 / ENSG00000149476
TKT / P29401 / ENSSSCG00000021408 / ENSG00000163931
TLN2 / Q9Y4G6 / ENSSSCG00000024088 / ENSG00000171914
TLR3 / O15455 / ENSSSCG00000015801 / ENSG00000164342
TLR7 / Q9NYK1 / ENSSSCG00000012117 / ENSG00000196664
TM9SF2 / Q99805 / ENSSSCG00000031808 / ENSG00000125304
TM9SF3 / Q9HD45 / ENSSSCG00000010508 / ENSG00000077147
TM9SF4 / Q92544 / ENSSSCG00000007246 / ENSG00000101337
TMA7 / Q9Y2S6 / ENSSSCG00000011351 / ENSG00000232112
TMBIM6 / P55061 / ENSSSCG00000029652 / ENSG00000139644
TMCO1 / Q9UM00 / ENSSSCG00000037314 / ENSG00000143183
TMED1 / Q13445 / ENSSSCG00000038623 / ENSG00000099203
TMED10 / P49755 / ENSSSCG00000035684 / ENSG00000170348
TMED2 / Q15363 / ENSSSCG00000009772 / ENSG00000086598
TMED4 / Q7Z7H5 / ENSSSCG00000016744 / ENSG00000158604
TMED5 / Q9Y3A6 / ENSSSCG00000040439 / ENSG00000117500
TMED9 / Q9BVK6 / ENSSSCG00000014036 / ENSG00000184840
TMEM109 / Q9BVC6 / ENSSSCG00000013110 / ENSG00000110108
TMEM11 / P17152 / ENSSSCG00000034040 / ENSG00000178307
TMEM126A / Q9H061 / ENSSSCG00000025187 / ENSG00000171202
TMEM141 / Q96I45 / ENSSSCG00000005842 / ENSG00000244187
TMEM165 / Q9HC07 / ENSSSCG00000032234 / ENSG00000134851
TMEM177 / Q53S58 / ENSSSCG00000015742 / ENSG00000144120
Homologs
Adipose.Tissue
Brain
Colon
Heart
Kidney
Liver
Lung
Pancreas
S.Intestine
Spleen
Organs
Species
Human
Pig
Bins
1
2
3
4
5

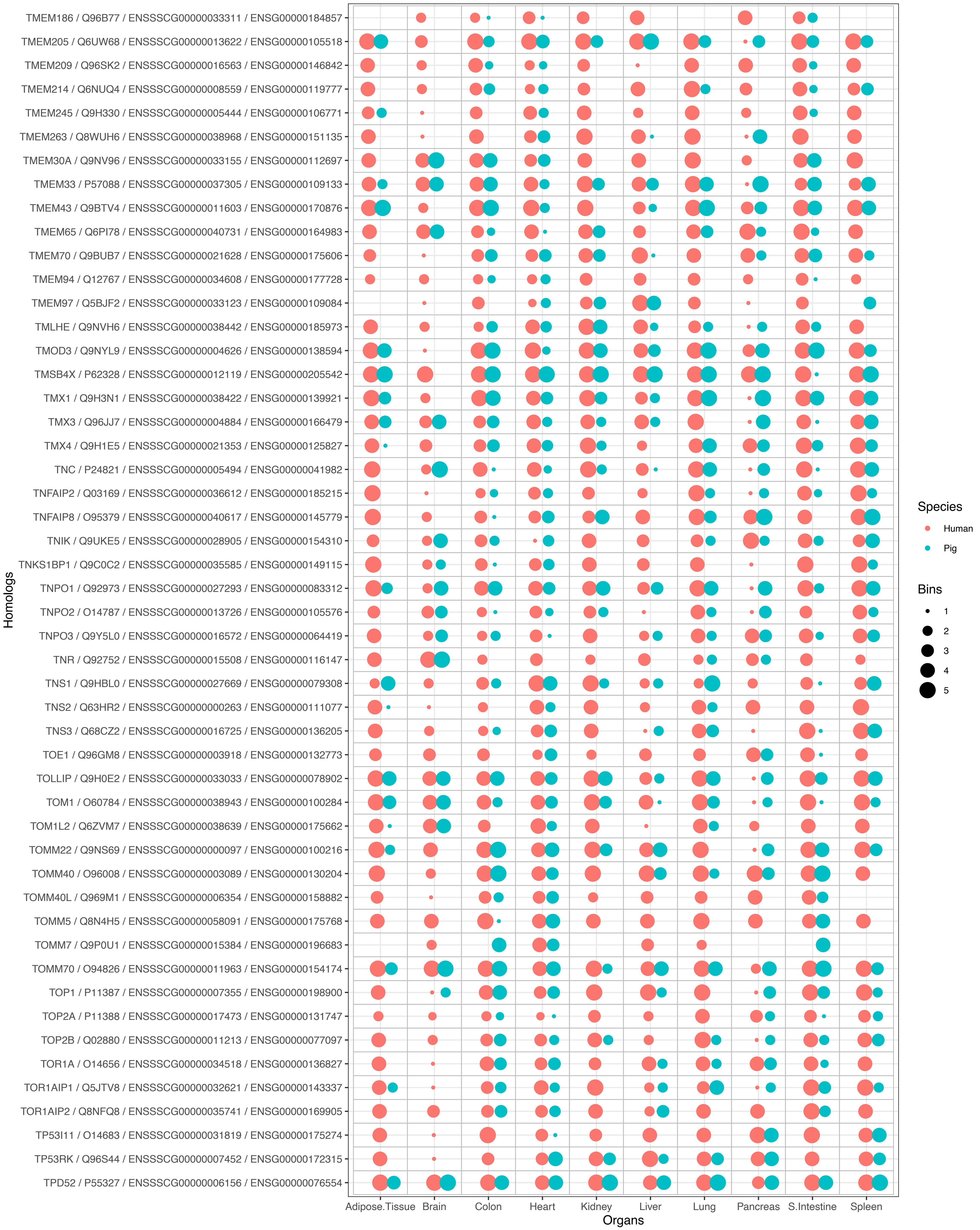
TMEM186 / Q96B77 / ENSSSCG00000033311 / ENSG00000184857
TMEM205 / Q6UW68 / ENSSSCG00000013622 / ENSG00000105518
TMEM209 / Q96SK2 / ENSSSCG00000016563 / ENSG00000146842
TMEM214 / Q6NUQ4 / ENSSSCG00000008559 / ENSG00000119777
TMEM245 / Q9H330 / ENSSSCG00000005444 / ENSG00000106771
TMEM263 / Q8WUH6 / ENSSSCG00000038968 / ENSG00000151135
TMEM30A / Q9NV96 / ENSSSCG00000033155 / ENSG00000112697
TMEM33 / P57088 / ENSSSCG00000037305 / ENSG00000109133
TMEM43 / Q9BTV4 / ENSSSCG00000011603 / ENSG00000170876
TMEM65 / Q6PI78 / ENSSSCG00000040731 / ENSG00000164983
TMEM70 / Q9BUB7 / ENSSSCG00000021628 / ENSG00000175606
TMEM94 / Q12767 / ENSSSCG00000034608 / ENSG00000177728
TMEM97 / Q5BJF2 / ENSSSCG00000033123 / ENSG00000109084
TMLHE / Q9NVH6 / ENSSSCG00000038442 / ENSG00000185973
TMOD3 / Q9NYL9 / ENSSSCG00000004626 / ENSG00000138594
TMSB4X / P62328 / ENSSSCG00000012119 / ENSG00000205542
TMX1 / Q9H3N1 / ENSSSCG00000038422 / ENSG00000139921
TMX3 / Q96JJ7 / ENSSSCG00000004884 / ENSG00000166479
TMX4 / Q9H1E5 / ENSSSCG00000021353 / ENSG00000125827
TNC / P24821 / ENSSSCG00000005494 / ENSG00000041982
TNFAIP2 / Q03169 / ENSSSCG00000036612 / ENSG00000185215
TNFAIP8 / O95379 / ENSSSCG00000040617 / ENSG00000145779
TNIK / Q9UKE5 / ENSSSCG00000028905 / ENSG00000154310
TNKS1BP1 / Q9C0C2 / ENSSSCG00000035585 / ENSG00000149115
TNPO1 / Q92973 / ENSSSCG00000027293 / ENSG00000083312
TNPO2 / O14787 / ENSSSCG00000013726 / ENSG00000105576
TNPO3 / Q9Y5L0 / ENSSSCG00000016572 / ENSG00000064419
TNR / Q92752 / ENSSSCG00000015508 / ENSG00000116147
TNS1 / Q9HBL0 / ENSSSCG00000027669 / ENSG00000079308
TNS2 / Q63HR2 / ENSSSCG00000000263 / ENSG00000111077
TNS3 / Q68CZ2 / ENSSSCG00000016725 / ENSG00000136205
TOE1 / Q96GM8 / ENSSSCG00000003918 / ENSG00000132773
TOLLIP / Q9H0E2 / ENSSSCG00000033033 / ENSG00000078902
TOM1 / O60784 / ENSSSCG00000038943 / ENSG00000100284
TOM1L2 / Q6ZVM7 / ENSSSCG00000038639 / ENSG00000175662
TOMM22 / Q9NS69 / ENSSSCG00000000097 / ENSG00000100216
TOMM40 / O96008 / ENSSSCG00000003089 / ENSG00000130204
TOMM40L / Q969M1 / ENSSSCG00000006354 / ENSG00000158882
TOMM5 / Q8N4H5 / ENSSSCG00000058091 / ENSG00000175768
TOMM7 / Q9P0U1 / ENSSSCG00000015384 / ENSG00000196683
TOMM70 / O94826 / ENSSSCG00000011963 / ENSG00000154174
TOP1 / P11387 / ENSSSCG00000007355 / ENSG00000198900
TOP2A / P11388 / ENSSSCG00000017473 / ENSG00000131747
TOP2B / Q02880 / ENSSSCG00000011213 / ENSG00000077097
TOR1A / O14656 / ENSSSCG00000034518 / ENSG00000136827
TOR1AIP1 / Q5JTV8 / ENSSSCG00000032621 / ENSG00000143337
TOR1AIP2 / Q8NFQ8 / ENSSSCG00000035741 / ENSG00000169905
TP53I11 / O14683 / ENSSSCG00000031819 / ENSG00000175274
TP53RK / Q96S44 / ENSSSCG00000007452 / ENSG00000172315
TPD52 / P55327 / ENSSSCG00000006156 / ENSG00000076554
Homologs
Adipose.Tissue
Brain
Colon
Heart
Kidney
Liver
Lung
Pancreas
S.Intestine
Spleen
Organs
Species
Human
Pig
Bins
1
2
3
4
5

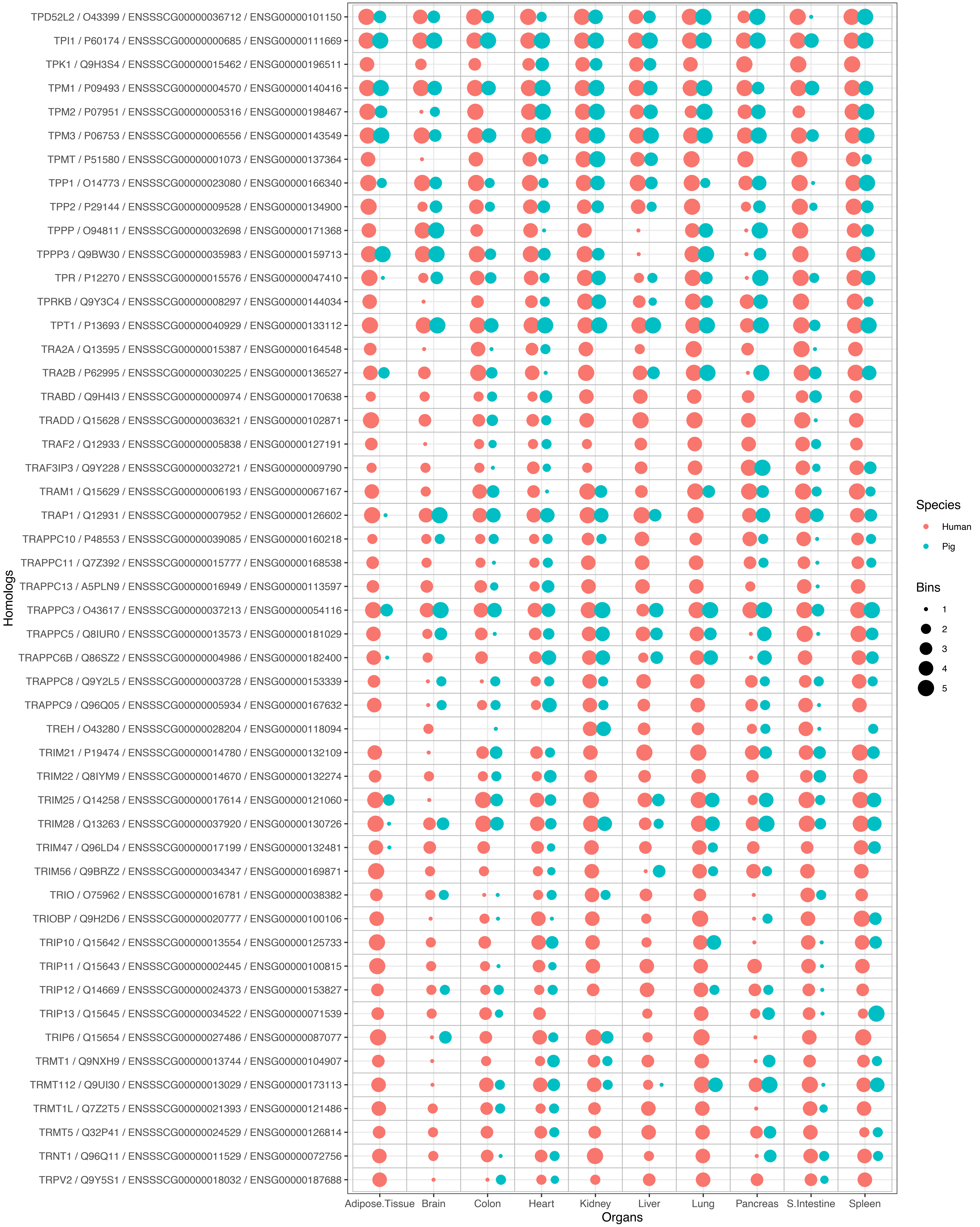
TPD52L2 / O43399 / ENSSSCG00000036712 / ENSG00000101150
TPI1 / P60174 / ENSSSCG00000000685 / ENSG00000111669
TPK1 / Q9H3S4 / ENSSSCG00000015462 / ENSG00000196511
TPM1 / P09493 / ENSSSCG00000004570 / ENSG00000140416
TPM2 / P07951 / ENSSSCG00000005316 / ENSG00000198467
TPM3 / P06753 / ENSSSCG00000006556 / ENSG00000143549
TPMT / P51580 / ENSSSCG00000001073 / ENSG00000137364
TPP1 / O14773 / ENSSSCG00000023080 / ENSG00000166340
TPP2 / P29144 / ENSSSCG00000009528 / ENSG00000134900
TPPP / O94811 / ENSSSCG00000032698 / ENSG00000171368
TPPP3 / Q9BW30 / ENSSSCG00000035983 / ENSG00000159713
TPR / P12270 / ENSSSCG00000015576 / ENSG00000047410
TPRKB / Q9Y3C4 / ENSSSCG00000008297 / ENSG00000144034
TPT1 / P13693 / ENSSSCG00000040929 / ENSG00000133112
TRA2A / Q13595 / ENSSSCG00000015387 / ENSG00000164548
TRA2B / P62995 / ENSSSCG00000030225 / ENSG00000136527
TRABD / Q9H4I3 / ENSSSCG00000000974 / ENSG00000170638
TRADD / Q15628 / ENSSSCG00000036321 / ENSG00000102871
TRAF2 / Q12933 / ENSSSCG00000005838 / ENSG00000127191
TRAF3IP3 / Q9Y228 / ENSSSCG00000032721 / ENSG00000009790
TRAM1 / Q15629 / ENSSSCG00000006193 / ENSG00000067167
TRAP1 / Q12931 / ENSSSCG00000007952 / ENSG00000126602
TRAPPC10 / P48553 / ENSSSCG00000039085 / ENSG00000160218
TRAPPC11 / Q7Z392 / ENSSSCG00000015777 / ENSG00000168538
TRAPPC13 / A5PLN9 / ENSSSCG00000016949 / ENSG00000113597
TRAPPC3 / O43617 / ENSSSCG00000037213 / ENSG00000054116
TRAPPC5 / Q8IUR0 / ENSSSCG00000013573 / ENSG00000181029
TRAPPC6B / Q86SZ2 / ENSSSCG00000004986 / ENSG00000182400
TRAPPC8 / Q9Y2L5 / ENSSSCG00000003728 / ENSG00000153339
TRAPPC9 / Q96Q05 / ENSSSCG00000005934 / ENSG00000167632
TREH / O43280 / ENSSSCG00000028204 / ENSG00000118094
TRIM21 / P19474 / ENSSSCG00000014780 / ENSG00000132109
TRIM22 / Q8IYM9 / ENSSSCG00000014670 / ENSG00000132274
TRIM25 / Q14258 / ENSSSCG00000017614 / ENSG00000121060
TRIM28 / Q13263 / ENSSSCG00000037920 / ENSG00000130726
TRIM47 / Q96LD4 / ENSSSCG00000017199 / ENSG00000132481
TRIM56 / Q9BRZ2 / ENSSSCG00000034347 / ENSG00000169871
TRIO / O75962 / ENSSSCG00000016781 / ENSG00000038382
TRIOBP / Q9H2D6 / ENSSSCG00000020777 / ENSG00000100106
TRIP10 / Q15642 / ENSSSCG00000013554 / ENSG00000125733
TRIP11 / Q15643 / ENSSSCG00000002445 / ENSG00000100815
TRIP12 / Q14669 / ENSSSCG00000024373 / ENSG00000153827
TRIP13 / Q15645 / ENSSSCG00000034522 / ENSG00000071539
TRIP6 / Q15654 / ENSSSCG00000027486 / ENSG00000087077
TRMT1 / Q9NXH9 / ENSSSCG00000013744 / ENSG00000104907
TRMT112 / Q9UI30 / ENSSSCG00000013029 / ENSG00000173113
TRMT1L / Q7Z2T5 / ENSSSCG00000021393 / ENSG00000121486
TRMT5 / Q32P41 / ENSSSCG00000024529 / ENSG00000126814
TRNT1 / Q96Q11 / ENSSSCG00000011529 / ENSG00000072756
TRPV2 / Q9Y5S1 / ENSSSCG00000018032 / ENSG00000187688
Homologs
Adipose.Tissue
Brain
Colon
Heart
Kidney
Liver
Lung
Pancreas
S.Intestine
Spleen
Organs
Species
Human
Pig
Bins
1
2
3
4
5

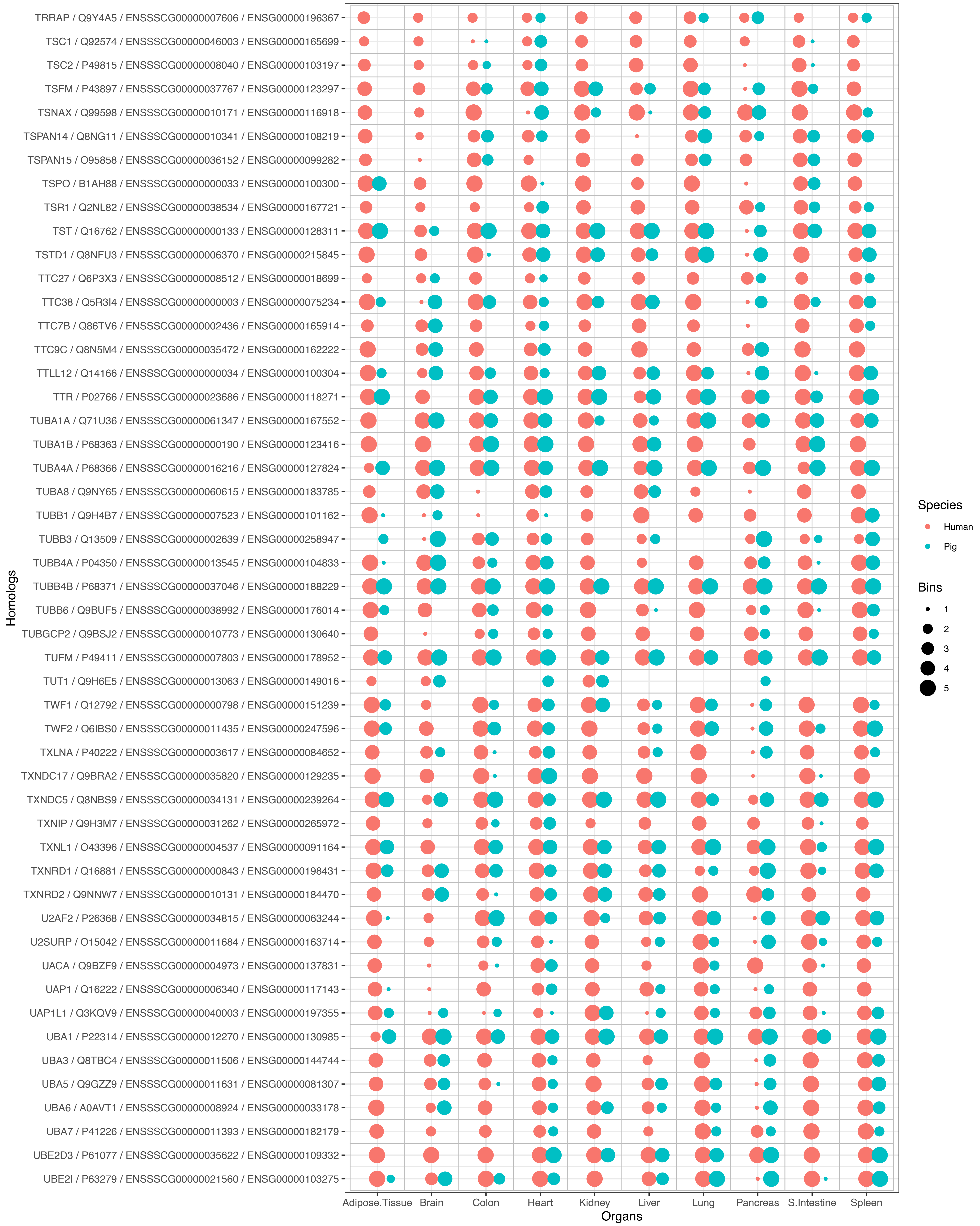
TRRAP / Q9Y4A5 / ENSSSCG00000007606 / ENSG00000196367
TSC1 / Q92574 / ENSSSCG00000046003 / ENSG00000165699
TSC2 / P49815 / ENSSSCG00000008040 / ENSG00000103197
TSFM / P43897 / ENSSSCG00000037767 / ENSG00000123297
TSNAX / Q99598 / ENSSSCG00000010171 / ENSG00000116918
TSPAN14 / Q8NG11 / ENSSSCG00000010341 / ENSG00000108219
TSPAN15 / O95858 / ENSSSCG00000036152 / ENSG00000099282
TSPO / B1AH88 / ENSSSCG00000000033 / ENSG00000100300
TSR1 / Q2NL82 / ENSSSCG00000038534 / ENSG00000167721
TST / Q16762 / ENSSSCG00000000133 / ENSG00000128311
TSTD1 / Q8NFU3 / ENSSSCG00000006370 / ENSG00000215845
TTC27 / Q6P3X3 / ENSSSCG00000008512 / ENSG00000018699
TTC38 / Q5R3I4 / ENSSSCG00000000003 / ENSG00000075234
TTC7B / Q86TV6 / ENSSSCG00000002436 / ENSG00000165914
TTC9C / Q8N5M4 / ENSSSCG00000035472 / ENSG00000162222
TTLL12 / Q14166 / ENSSSCG00000000034 / ENSG00000100304
TTR / P02766 / ENSSSCG00000023686 / ENSG00000118271
TUBA1A / Q71U36 / ENSSSCG00000061347 / ENSG00000167552
TUBA1B / P68363 / ENSSSCG00000000190 / ENSG00000123416
TUBA4A / P68366 / ENSSSCG00000016216 / ENSG00000127824
TUBA8 / Q9NY65 / ENSSSCG00000060615 / ENSG00000183785
TUBB1 / Q9H4B7 / ENSSSCG00000007523 / ENSG00000101162
TUBB3 / Q13509 / ENSSSCG00000002639 / ENSG00000258947
TUBB4A / P04350 / ENSSSCG00000013545 / ENSG00000104833
TUBB4B / P68371 / ENSSSCG00000037046 / ENSG00000188229
TUBB6 / Q9BUF5 / ENSSSCG00000038992 / ENSG00000176014
TUBGCP2 / Q9BSJ2 / ENSSSCG00000010773 / ENSG00000130640
TUFM / P49411 / ENSSSCG00000007803 / ENSG00000178952
TUT1 / Q9H6E5 / ENSSSCG00000013063 / ENSG00000149016
TWF1 / Q12792 / ENSSSCG00000000798 / ENSG00000151239
TWF2 / Q6IBS0 / ENSSSCG00000011435 / ENSG00000247596
TXLNA / P40222 / ENSSSCG00000003617 / ENSG00000084652
TXNDC17 / Q9BRA2 / ENSSSCG00000035820 / ENSG00000129235
TXNDC5 / Q8NBS9 / ENSSSCG00000034131 / ENSG00000239264
TXNIP / Q9H3M7 / ENSSSCG00000031262 / ENSG00000265972
TXNL1 / O43396 / ENSSSCG00000004537 / ENSG00000091164
TXNRD1 / Q16881 / ENSSSCG00000000843 / ENSG00000198431
TXNRD2 / Q9NNW7 / ENSSSCG00000010131 / ENSG00000184470
U2AF2 / P26368 / ENSSSCG00000034815 / ENSG00000063244
U2SURP / O15042 / ENSSSCG00000011684 / ENSG00000163714
UACA / Q9BZF9 / ENSSSCG00000004973 / ENSG00000137831
UAP1 / Q16222 / ENSSSCG00000006340 / ENSG00000117143
UAP1L1 / Q3KQV9 / ENSSSCG00000040003 / ENSG00000197355
UBA1 / P22314 / ENSSSCG00000012270 / ENSG00000130985
UBA3 / Q8TBC4 / ENSSSCG00000011506 / ENSG00000144744
UBA5 / Q9GZZ9 / ENSSSCG00000011631 / ENSG00000081307
UBA6 / A0AVT1 / ENSSSCG00000008924 / ENSG00000033178
UBA7 / P41226 / ENSSSCG00000011393 / ENSG00000182179
UBE2D3 / P61077 / ENSSSCG00000035622 / ENSG00000109332
UBE2I / P63279 / ENSSSCG00000021560 / ENSG00000103275
Homologs
Adipose.Tissue
Brain
Colon
Heart
Kidney
Liver
Lung
Pancreas
S.Intestine
Spleen
Organs
Species
Human
Pig
Bins
1
2
3
4
5

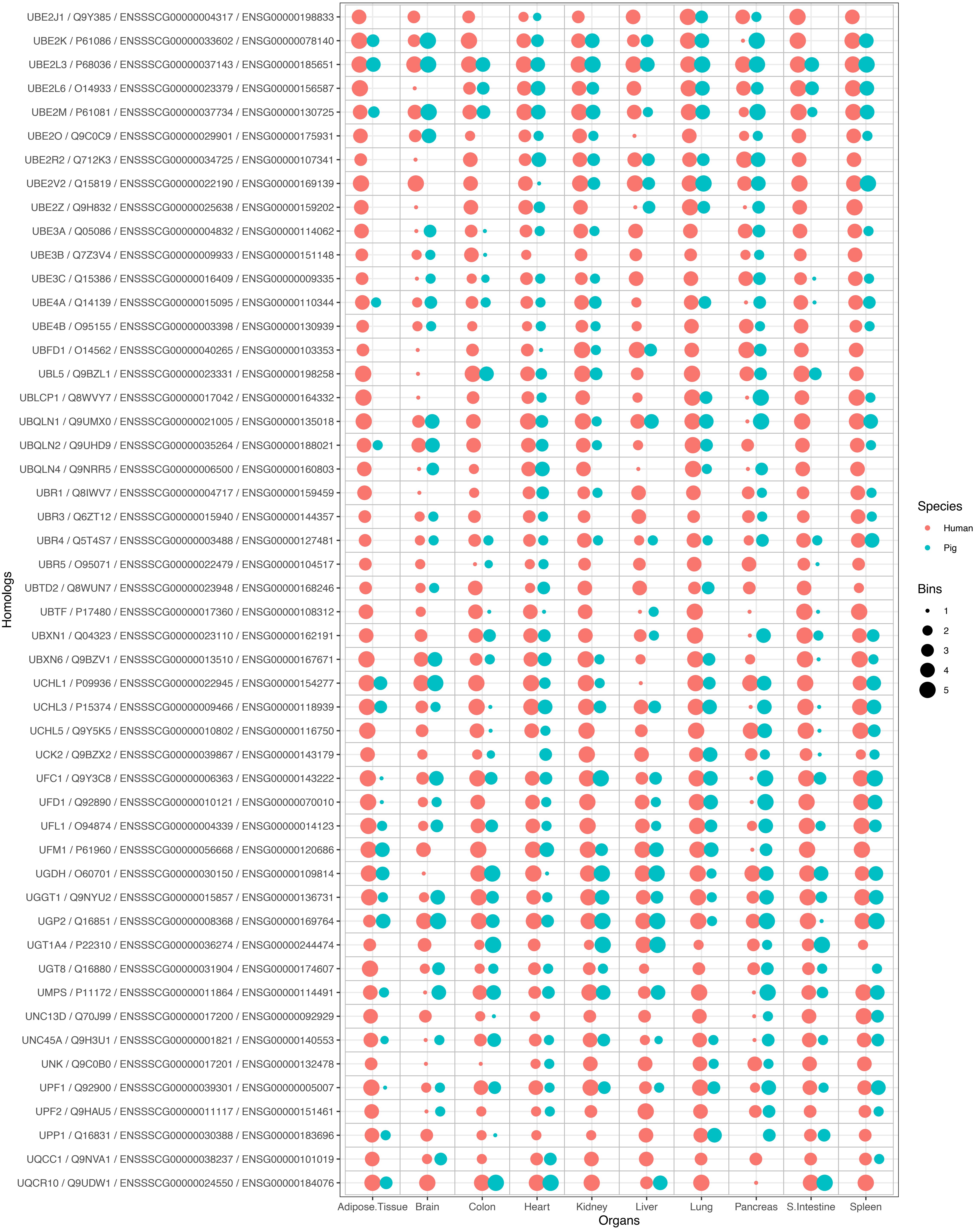
UBE2J1 / Q9Y385 / ENSSSCG00000004317 / ENSG00000198833
UBE2K / P61086 / ENSSSCG00000033602 / ENSG00000078140
UBE2L3 / P68036 / ENSSSCG00000037143 / ENSG00000185651
UBE2L6 / O14933 / ENSSSCG00000023379 / ENSG00000156587
UBE2M / P61081 / ENSSSCG00000037734 / ENSG00000130725
UBE2O / Q9C0C9 / ENSSSCG00000029901 / ENSG00000175931
UBE2R2 / Q712K3 / ENSSSCG00000034725 / ENSG00000107341
UBE2V2 / Q15819 / ENSSSCG00000022190 / ENSG00000169139
UBE2Z / Q9H832 / ENSSSCG00000025638 / ENSG00000159202
UBE3A / Q05086 / ENSSSCG00000004832 / ENSG00000114062
UBE3B / Q7Z3V4 / ENSSSCG00000009933 / ENSG00000151148
UBE3C / Q15386 / ENSSSCG00000016409 / ENSG00000009335
UBE4A / Q14139 / ENSSSCG00000015095 / ENSG00000110344
UBE4B / O95155 / ENSSSCG00000003398 / ENSG00000130939
UBFD1 / O14562 / ENSSSCG00000040265 / ENSG00000103353
UBL5 / Q9BZL1 / ENSSSCG00000023331 / ENSG00000198258
UBLCP1 / Q8WVY7 / ENSSSCG00000017042 / ENSG00000164332
UBQLN1 / Q9UMX0 / ENSSSCG00000021005 / ENSG00000135018
UBQLN2 / Q9UHD9 / ENSSSCG00000035264 / ENSG00000188021
UBQLN4 / Q9NRR5 / ENSSSCG00000006500 / ENSG00000160803
UBR1 / Q8IWV7 / ENSSSCG00000004717 / ENSG00000159459
UBR3 / Q6ZT12 / ENSSSCG00000015940 / ENSG00000144357
UBR4 / Q5T4S7 / ENSSSCG00000003488 / ENSG00000127481
UBR5 / O95071 / ENSSSCG00000022479 / ENSG00000104517
UBTD2 / Q8WUN7 / ENSSSCG00000023948 / ENSG00000168246
UBTF / P17480 / ENSSSCG00000017360 / ENSG00000108312
UBXN1 / Q04323 / ENSSSCG00000023110 / ENSG00000162191
UBXN6 / Q9BZV1 / ENSSSCG00000013510 / ENSG00000167671
UCHL1 / P09936 / ENSSSCG00000022945 / ENSG00000154277
UCHL3 / P15374 / ENSSSCG00000009466 / ENSG00000118939
UCHL5 / Q9Y5K5 / ENSSSCG00000010802 / ENSG00000116750
UCK2 / Q9BZX2 / ENSSSCG00000039867 / ENSG00000143179
UFC1 / Q9Y3C8 / ENSSSCG00000006363 / ENSG00000143222
UFD1 / Q92890 / ENSSSCG00000010121 / ENSG00000070010
UFL1 / O94874 / ENSSSCG00000004339 / ENSG00000014123
UFM1 / P61960 / ENSSSCG00000056668 / ENSG00000120686
UGDH / O60701 / ENSSSCG00000030150 / ENSG00000109814
UGGT1 / Q9NYU2 / ENSSSCG00000015857 / ENSG00000136731
UGP2 / Q16851 / ENSSSCG00000008368 / ENSG00000169764
UGT1A4 / P22310 / ENSSSCG00000036274 / ENSG00000244474
UGT8 / Q16880 / ENSSSCG00000031904 / ENSG00000174607
UMPS / P11172 / ENSSSCG00000011864 / ENSG00000114491
UNC13D / Q70J99 / ENSSSCG00000017200 / ENSG00000092929
UNC45A / Q9H3U1 / ENSSSCG00000001821 / ENSG00000140553
UNK / Q9C0B0 / ENSSSCG00000017201 / ENSG00000132478
UPF1 / Q92900 / ENSSSCG00000039301 / ENSG00000005007
UPF2 / Q9HAU5 / ENSSSCG00000011117 / ENSG00000151461
UPP1 / Q16831 / ENSSSCG00000030388 / ENSG00000183696
UQCC1 / Q9NVA1 / ENSSSCG00000038237 / ENSG00000101019
UQCR10 / Q9UDW1 / ENSSSCG00000024550 / ENSG00000184076
Homologs
Adipose.Tissue
Brain
Colon
Heart
Kidney
Liver
Lung
Pancreas
S.Intestine
Spleen
Organs
Species
Human
Pig
Bins
1
2
3
4
5

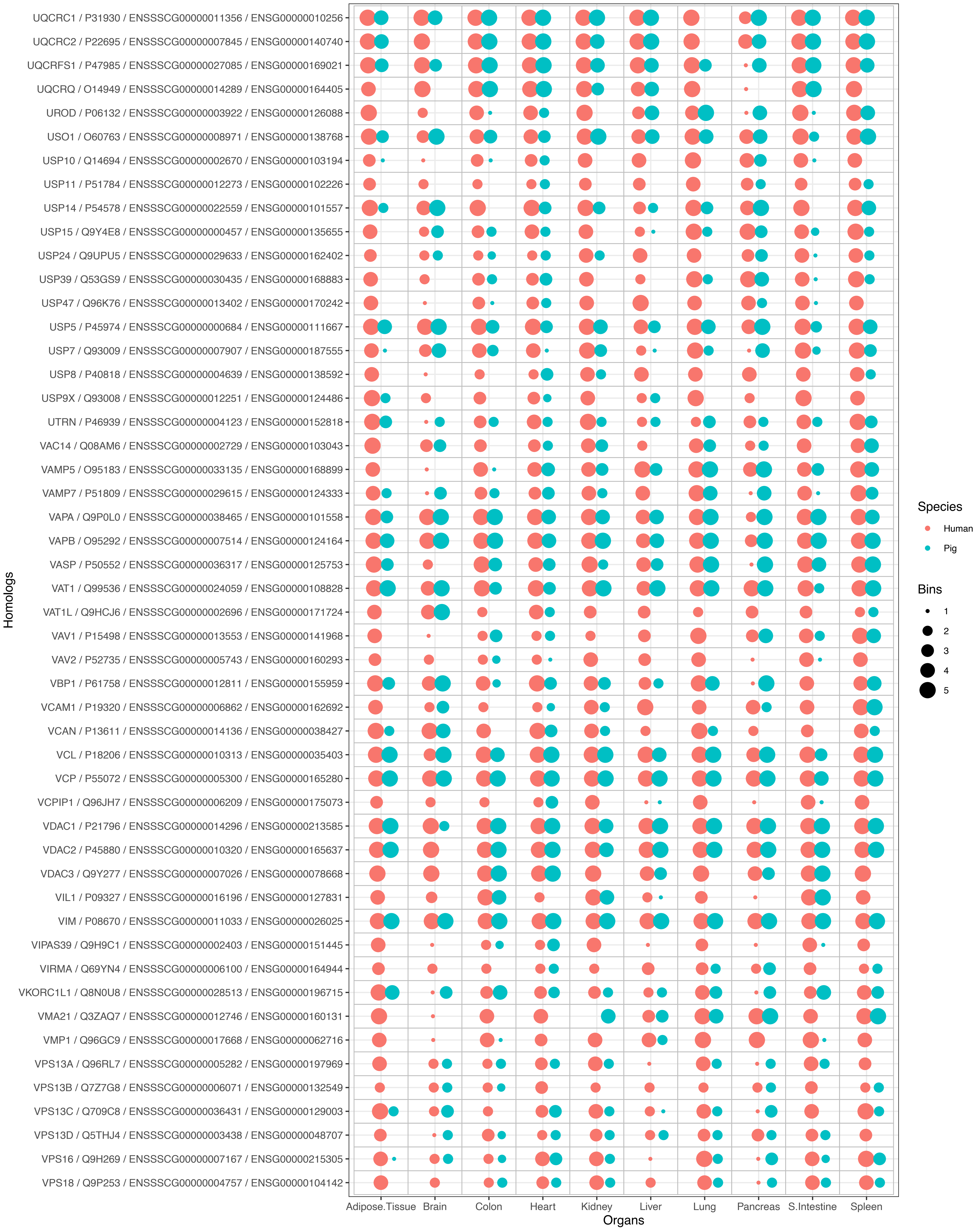
UQCRC1 / P31930 / ENSSSCG00000011356 / ENSG00000010256
UQCRC2 / P22695 / ENSSSCG00000007845 / ENSG00000140740
UQCRFS1 / P47985 / ENSSSCG00000027085 / ENSG00000169021
UQCRQ / O14949 / ENSSSCG00000014289 / ENSG00000164405
UROD / P06132 / ENSSSCG00000003922 / ENSG00000126088
USO1 / O60763 / ENSSSCG00000008971 / ENSG00000138768
USP10 / Q14694 / ENSSSCG00000002670 / ENSG00000103194
USP11 / P51784 / ENSSSCG00000012273 / ENSG00000102226
USP14 / P54578 / ENSSSCG00000022559 / ENSG00000101557
USP15 / Q9Y4E8 / ENSSSCG00000000457 / ENSG00000135655
USP24 / Q9UPU5 / ENSSSCG00000029633 / ENSG00000162402
USP39 / Q53GS9 / ENSSSCG00000030435 / ENSG00000168883
USP47 / Q96K76 / ENSSSCG00000013402 / ENSG00000170242
USP5 / P45974 / ENSSSCG00000000684 / ENSG00000111667
USP7 / Q93009 / ENSSSCG00000007907 / ENSG00000187555
USP8 / P40818 / ENSSSCG00000004639 / ENSG00000138592
USP9X / Q93008 / ENSSSCG00000012251 / ENSG00000124486
UTRN / P46939 / ENSSSCG00000004123 / ENSG00000152818
VAC14 / Q08AM6 / ENSSSCG00000002729 / ENSG00000103043
VAMP5 / O95183 / ENSSSCG00000033135 / ENSG00000168899
VAMP7 / P51809 / ENSSSCG00000029615 / ENSG00000124333
VAPA / Q9P0L0 / ENSSSCG00000038465 / ENSG00000101558
VAPB / O95292 / ENSSSCG00000007514 / ENSG00000124164
VASP / P50552 / ENSSSCG00000036317 / ENSG00000125753
VAT1 / Q99536 / ENSSSCG00000024059 / ENSG00000108828
VAT1L / Q9HCJ6 / ENSSSCG00000002696 / ENSG00000171724
VAV1 / P15498 / ENSSSCG00000013553 / ENSG00000141968
VAV2 / P52735 / ENSSSCG00000005743 / ENSG00000160293
VBP1 / P61758 / ENSSSCG00000012811 / ENSG00000155959
VCAM1 / P19320 / ENSSSCG00000006862 / ENSG00000162692
VCAN / P13611 / ENSSSCG00000014136 / ENSG00000038427
VCL / P18206 / ENSSSCG00000010313 / ENSG00000035403
VCP / P55072 / ENSSSCG00000005300 / ENSG00000165280
VCPIP1 / Q96JH7 / ENSSSCG00000006209 / ENSG00000175073
VDAC1 / P21796 / ENSSSCG00000014296 / ENSG00000213585
VDAC2 / P45880 / ENSSSCG00000010320 / ENSG00000165637
VDAC3 / Q9Y277 / ENSSSCG00000007026 / ENSG00000078668
VIL1 / P09327 / ENSSSCG00000016196 / ENSG00000127831
VIM / P08670 / ENSSSCG00000011033 / ENSG00000026025
VIPAS39 / Q9H9C1 / ENSSSCG00000002403 / ENSG00000151445
VIRMA / Q69YN4 / ENSSSCG00000006100 / ENSG00000164944
VKORC1L1 / Q8N0U8 / ENSSSCG00000028513 / ENSG00000196715
VMA21 / Q3ZAQ7 / ENSSSCG00000012746 / ENSG00000160131
VMP1 / Q96GC9 / ENSSSCG00000017668 / ENSG00000062716
VPS13A / Q96RL7 / ENSSSCG00000005282 / ENSG00000197969
VPS13B / Q7Z7G8 / ENSSSCG00000006071 / ENSG00000132549
VPS13C / Q709C8 / ENSSSCG00000036431 / ENSG00000129003
VPS13D / Q5THJ4 / ENSSSCG00000003438 / ENSG00000048707
VPS16 / Q9H269 / ENSSSCG00000007167 / ENSG00000215305
VPS18 / Q9P253 / ENSSSCG00000004757 / ENSG00000104142
Homologs
Adipose.Tissue
Brain
Colon
Heart
Kidney
Liver
Lung
Pancreas
S.Intestine
Spleen
Organs
Species
Human
Pig
Bins
1
2
3
4
5

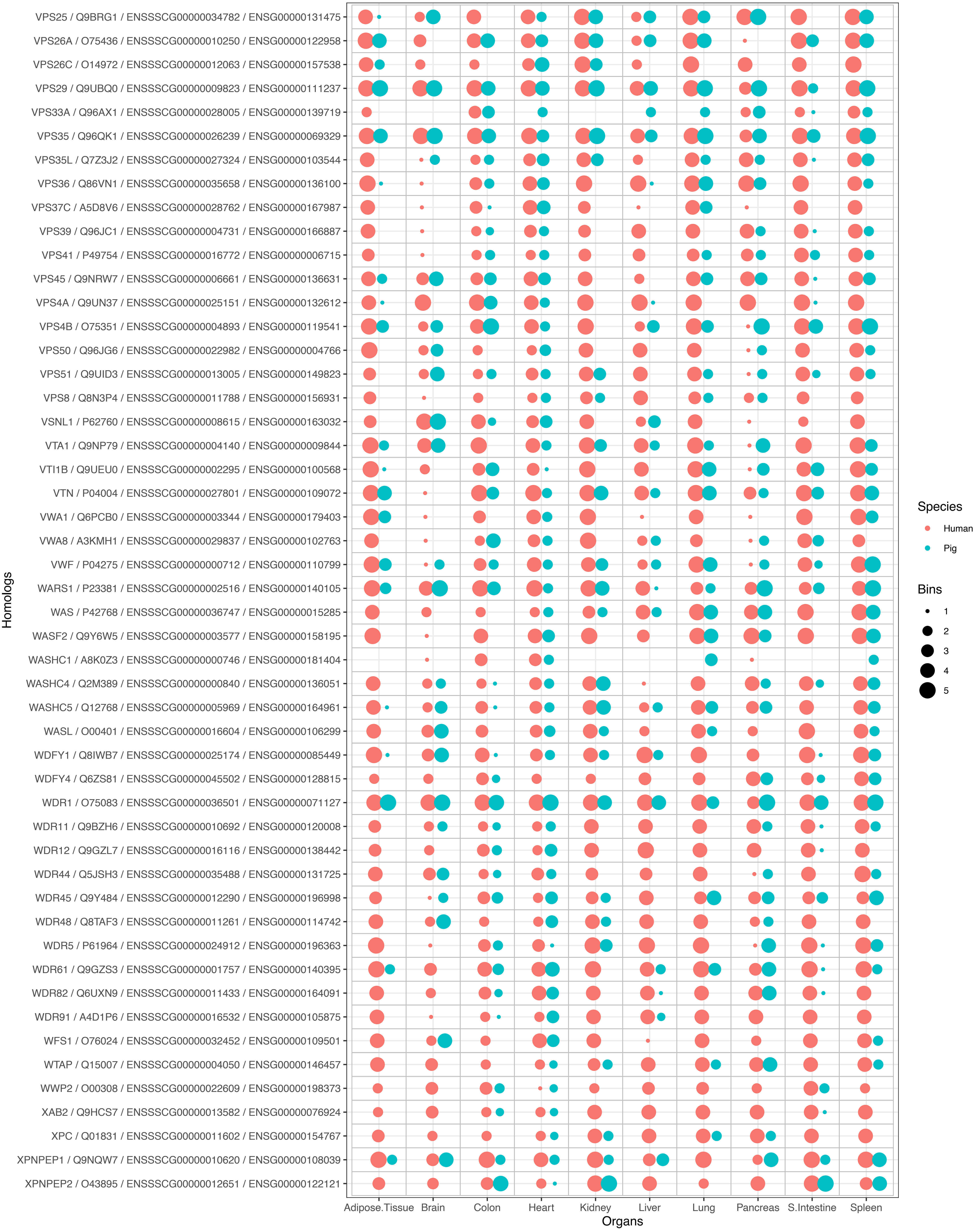
VPS25 / Q9BRG1 / ENSSSCG00000034782 / ENSG00000131475
VPS26A / O75436 / ENSSSCG00000010250 / ENSG00000122958
VPS26C / O14972 / ENSSSCG00000012063 / ENSG00000157538
VPS29 / Q9UBQ0 / ENSSSCG00000009823 / ENSG00000111237
VPS33A / Q96AX1 / ENSSSCG00000028005 / ENSG00000139719
VPS35 / Q96QK1 / ENSSSCG00000026239 / ENSG00000069329
VPS35L / Q7Z3J2 / ENSSSCG00000027324 / ENSG00000103544
VPS36 / Q86VN1 / ENSSSCG00000035658 / ENSG00000136100
VPS37C / A5D8V6 / ENSSSCG00000028762 / ENSG00000167987
VPS39 / Q96JC1 / ENSSSCG00000004731 / ENSG00000166887
VPS41 / P49754 / ENSSSCG00000016772 / ENSG00000006715
VPS45 / Q9NRW7 / ENSSSCG00000006661 / ENSG00000136631
VPS4A / Q9UN37 / ENSSSCG00000025151 / ENSG00000132612
VPS4B / O75351 / ENSSSCG00000004893 / ENSG00000119541
VPS50 / Q96JG6 / ENSSSCG00000022982 / ENSG00000004766
VPS51 / Q9UID3 / ENSSSCG00000013005 / ENSG00000149823
VPS8 / Q8N3P4 / ENSSSCG00000011788 / ENSG00000156931
VSNL1 / P62760 / ENSSSCG00000008615 / ENSG00000163032
VTA1 / Q9NP79 / ENSSSCG00000004140 / ENSG00000009844
VTI1B / Q9UEU0 / ENSSSCG00000002295 / ENSG00000100568
VTN / P04004 / ENSSSCG00000027801 / ENSG00000109072
VWA1 / Q6PCB0 / ENSSSCG00000003344 / ENSG00000179403
VWA8 / A3KMH1 / ENSSSCG00000029837 / ENSG00000102763
VWF / P04275 / ENSSSCG00000000712 / ENSG00000110799
WARS1 / P23381 / ENSSSCG00000002516 / ENSG00000140105
WAS / P42768 / ENSSSCG00000036747 / ENSG00000015285
WASF2 / Q9Y6W5 / ENSSSCG00000003577 / ENSG00000158195
WASHC1 / A8K0Z3 / ENSSSCG00000000746 / ENSG00000181404
WASHC4 / Q2M389 / ENSSSCG00000000840 / ENSG00000136051
WASHC5 / Q12768 / ENSSSCG00000005969 / ENSG00000164961
WASL / O00401 / ENSSSCG00000016604 / ENSG00000106299
WDFY1 / Q8IWB7 / ENSSSCG00000025174 / ENSG00000085449
WDFY4 / Q6ZS81 / ENSSSCG00000045502 / ENSG00000128815
WDR1 / O75083 / ENSSSCG00000036501 / ENSG00000071127
WDR11 / Q9BZH6 / ENSSSCG00000010692 / ENSG00000120008
WDR12 / Q9GZL7 / ENSSSCG00000016116 / ENSG00000138442
WDR44 / Q5JSH3 / ENSSSCG00000035488 / ENSG00000131725
WDR45 / Q9Y484 / ENSSSCG00000012290 / ENSG00000196998
WDR48 / Q8TAF3 / ENSSSCG00000011261 / ENSG00000114742
WDR5 / P61964 / ENSSSCG00000024912 / ENSG00000196363
WDR61 / Q9GZS3 / ENSSSCG00000001757 / ENSG00000140395
WDR82 / Q6UXN9 / ENSSSCG00000011433 / ENSG00000164091
WDR91 / A4D1P6 / ENSSSCG00000016532 / ENSG00000105875
WFS1 / O76024 / ENSSSCG00000032452 / ENSG00000109501
WTAP / Q15007 / ENSSSCG00000004050 / ENSG00000146457
WWP2 / O00308 / ENSSSCG00000022609 / ENSG00000198373
XAB2 / Q9HCS7 / ENSSSCG00000013582 / ENSG00000076924
XPC / Q01831 / ENSSSCG00000011602 / ENSG00000154767
XPNPEP1 / Q9NQW7 / ENSSSCG00000010620 / ENSG00000108039
XPNPEP2 / O43895 / ENSSSCG00000012651 / ENSG00000122121
Homologs
Adipose.Tissue
Brain
Colon
Heart
Kidney
Liver
Lung
Pancreas
S.Intestine
Spleen
Organs
Species
Human
Pig
Bins
1
2
3
4
5

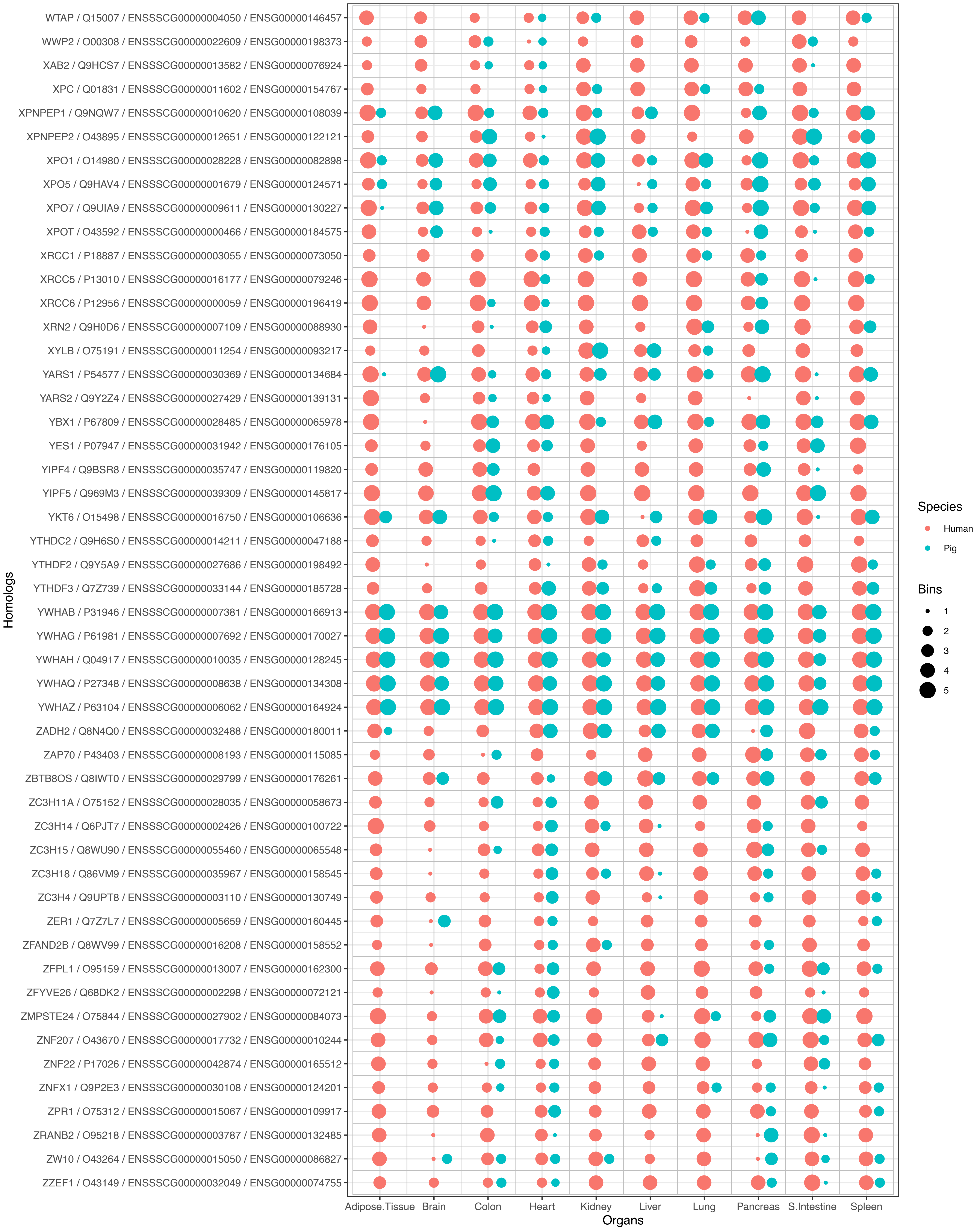
WTAP / Q15007 / ENSSSCG00000004050 / ENSG00000146457
WWP2 / O00308 / ENSSSCG00000022609 / ENSG00000198373
XAB2 / Q9HCS7 / ENSSSCG00000013582 / ENSG00000076924
XPC / Q01831 / ENSSSCG00000011602 / ENSG00000154767
XPNPEP1 / Q9NQW7 / ENSSSCG00000010620 / ENSG00000108039
XPNPEP2 / O43895 / ENSSSCG00000012651 / ENSG00000122121
XPO1 / O14980 / ENSSSCG00000028228 / ENSG00000082898
XPO5 / Q9HAV4 / ENSSSCG00000001679 / ENSG00000124571
XPO7 / Q9UIA9 / ENSSSCG00000009611 / ENSG00000130227
XPOT / O43592 / ENSSSCG00000000466 / ENSG00000184575
XRCC1 / P18887 / ENSSSCG00000003055 / ENSG00000073050
XRCC5 / P13010 / ENSSSCG00000016177 / ENSG00000079246
XRCC6 / P12956 / ENSSSCG00000000059 / ENSG00000196419
XRN2 / Q9H0D6 / ENSSSCG00000007109 / ENSG00000088930
XYLB / O75191 / ENSSSCG00000011254 / ENSG00000093217
YARS1 / P54577 / ENSSSCG00000030369 / ENSG00000134684
YARS2 / Q9Y2Z4 / ENSSSCG00000027429 / ENSG00000139131
YBX1 / P67809 / ENSSSCG00000028485 / ENSG00000065978
YES1 / P07947 / ENSSSCG00000031942 / ENSG00000176105
YIPF4 / Q9BSR8 / ENSSSCG00000035747 / ENSG00000119820
YIPF5 / Q969M3 / ENSSSCG00000039309 / ENSG00000145817
YKT6 / O15498 / ENSSSCG00000016750 / ENSG00000106636
YTHDC2 / Q9H6S0 / ENSSSCG00000014211 / ENSG00000047188
YTHDF2 / Q9Y5A9 / ENSSSCG00000027686 / ENSG00000198492
YTHDF3 / Q7Z739 / ENSSSCG00000033144 / ENSG00000185728
YWHAB / P31946 / ENSSSCG00000007381 / ENSG00000166913
YWHAG / P61981 / ENSSSCG00000007692 / ENSG00000170027
YWHAH / Q04917 / ENSSSCG00000010035 / ENSG00000128245
YWHAQ / P27348 / ENSSSCG00000008638 / ENSG00000134308
YWHAZ / P63104 / ENSSSCG00000006062 / ENSG00000164924
ZADH2 / Q8N4Q0 / ENSSSCG00000032488 / ENSG00000180011
ZAP70 / P43403 / ENSSSCG00000008193 / ENSG00000115085
ZBTB8OS / Q8IWT0 / ENSSSCG00000029799 / ENSG00000176261
ZC3H11A / O75152 / ENSSSCG00000028035 / ENSG00000058673
ZC3H14 / Q6PJT7 / ENSSSCG00000002426 / ENSG00000100722
ZC3H15 / Q8WU90 / ENSSSCG00000055460 / ENSG00000065548
ZC3H18 / Q86VM9 / ENSSSCG00000035967 / ENSG00000158545
ZC3H4 / Q9UPT8 / ENSSSCG00000003110 / ENSG00000130749
ZER1 / Q7Z7L7 / ENSSSCG00000005659 / ENSG00000160445
ZFAND2B / Q8WV99 / ENSSSCG00000016208 / ENSG00000158552
ZFPL1 / O95159 / ENSSSCG00000013007 / ENSG00000162300
ZFYVE26 / Q68DK2 / ENSSSCG00000002298 / ENSG00000072121
ZMPSTE24 / O75844 / ENSSSCG00000027902 / ENSG00000084073
ZNF207 / O43670 / ENSSSCG00000017732 / ENSG00000010244
ZNF22 / P17026 / ENSSSCG00000042874 / ENSG00000165512
ZNFX1 / Q9P2E3 / ENSSSCG00000030108 / ENSG00000124201
ZPR1 / O75312 / ENSSSCG00000015067 / ENSG00000109917
ZRANB2 / O95218 / ENSSSCG00000003787 / ENSG00000132485
ZW10 / O43264 / ENSSSCG00000015050 / ENSG00000086827
ZZEF1 / O43149 / ENSSSCG00000032049 / ENSG00000074755
Homologs
Adipose.Tissue
Brain
Colon
Heart
Kidney
Liver
Lung
Pancreas
S.Intestine
Spleen
Organs
Species
Human
Pig
Bins
1
2
3
4
5
